# Parental monitoring challenges and recommendation for managing excessive screen use among students aged 12–14 years in Dhaka, Bangladesh: A qualitative exploration

**DOI:** 10.1371/journal.pone.0359518

**Published:** 2026-10-05

**Authors:** Shahria Hafiz Kakon, Nahian Sultana, Bidhan Krishna Sarker, Tanjir Rashid Soron, Rashidul Haque, Fahmida Tofail

**Affiliations:** 1 Infectious Diseases Division, International Centre for Diarrheal Disease Research, Dhaka, Bangladesh; 2 Nutrition Research Division, International Centre for Diarrheal Disease Research, Dhaka, Bangladesh; 3 Maternal and Child Health Division, International Centre for Diarrheal Disease Research, Dhaka, Bangladesh; 4 Telepsychiatry Research and Innovation Network Ltd, Dhaka, Bangladesh; Dilla university, ETHIOPIA

## Abstract

**Background:**

The pervasive use of social media and recreational internet platforms among students has become increasingly evident. The proper use of devices for students is critical; excessive use leads to a negative impact on students in many areas of their lives. This study aimed to explore the challenges parents face when their children engage in excessive use of electronic digital screens. Additionally, it sought to capture parents, teachers, and student’s,’ recommendations for the best use of digital devices among students.

**Methods:**

An exploratory qualitative study design was employed to understand parental experience in regulating screen use and strategies adopted to manage its duration and quality. Data was collected between January and April 2024. Students aged between 12 and 14 who attend class six to ten English and Bangla medium schools in Dhaka, and their parents and teachers, were the participants in this study. Data were collected through focus group discussions and in-depth interviews, which were conducted with parents, teachers, and students to capture the perspectives of each stakeholder group. A thematic analysis was conducted in which themes were initially identified inductively from the data and subsequently organized and interpreted using the socio-ecological model as an analytical framework.

**Results:**

We included 25 students who attended classes six and ten, of whom 56% were male. The key parental challenges identified in this study, including monitoring children’s screen viewing, supervising age appropriate online content, balancing educational and recreational device use, promoting alternative offline activities and lack of digital literacy among parents. Participants’ recommendations included school-based awareness. interventions, national-level guidelines regarding the use of digital devices, development of self-regulatory behavior in students, provision of secure recreational infrastructures, creating scope for outdoor recreational activities among students, enhancing the parent-child bond, and offering interesting options to divert the students from screens.

**Conclusions:**

The study findings provide a better understanding of the parental challenges of the overuse of devices and recommendations among the population. Effective guidelines for managing students’ screen use require participation from schools, parents, legislators, and students themselves. Further research is required to determine the most effective interventions.

## Introduction

The presence of technology in everyday life has become inescapable. The boundaries between digital and real worlds have blurred, and children are increasingly immersed in virtual media, gaming, and online communication [1,2]. While digital tools can provide educational and recreational benefits, excessive screen viewing (SV) has been associated with adverse impacts on children’s health and development [3]. Existing evidence highlights the influential role of socio-economic status (SES) in shaping parenting practices related to children’s technology use [4]. Four key factors influenced screen time practices: parenting practices, digital skill expectations, access to digital resources, and opportunities for alternative recreational activities. For example, higher-SES parents often weighed concerns about screen-related harms against the importance of developing children’s digital skill [5]. Parenting styles also influence children’s technology habits, with some parents permitting more device use as part of an individualized parenting approach, even when this exceeds pediatric guidelines [4]. A relevant quantitative study on children about screen time reported that the class year was positively and significantly associated with the average time spent on electronic devices during school days, weekends, and holidays [6].

Evidence links prolonged SV with negative outcomes such as poorer mental health, sleep disturbance, reduced academic performance, and behavioral problems, although in some cases, screen use has also been associated with improved peer relationships in adolescents [7,8]. Another recent paper on SV and Adolescent Brain Cognitive Development (ABCD) further indicated associations between high screen time and depressive symptoms, conduct disorders, and attention-deficit/hyperactivity disorder [8,9]. The type of screen activity is important, as texting, video chatting, video streaming, and gaming are more strongly linked to mental health problems than other types of screen use [9]. Excessive SV is commonly defined as recreational screen use exceeding 2 hours per day, which the WHO considers part of prolonged sedentary behaviour that should be limited and replaced with physical activity for optimal health [10].

In Bangladesh, a recent study reported that recreational SV is rising rapidly among adolescents. In today’s modern world, children engage in using a wide range of digital devices such as smartphones, tablets, laptops, and game consoles in addition to watching television [11]. A study found that approximately 79% of adolescents exceeded the WHO recommended recreational screen viewing [11]. The WHO and the American Academy of Pediatrics (AAP) both recommend limiting screen exposure in early childhood. The WHO recommends no screen time for children under 2 years and no more than one hour per day for those aged 2–5 years, while the AAP recommends avoiding digital media before 18 months (except video chatting) and limiting high-quality, parent-supervised screen use to no more than one hour per day for children aged 2–5 years [2,12]. Median reported recreational screen time was found to be 4.0 hours/day, with boys having longer times (4.3 hours/day) compared to girls (3.6 hours/day). Further, the study reported that high SV was associated with unhealthy behaviors, including frequent fast-food consumption and difficulty falling asleep [11]. The trend of using more than one device simultaneously is even observed nowadays [2]. It’s popular to use multiple digital gadgets with the use of internet media for a range of objectives, including leisure, social communication, and academic duties [12]. However, no such tailored guidelines currently exist in the Bangladesh context.

Parents across both high-income countries (HICs) and low- and middle-income countries (LMICs), including Bangladesh, face similar challenges in managing adolescents’ screen viewing, despite differences in social and cultural contexts. Appropriate parental regulation of screen viewing is essential, as excessive restrictions may reduce opportunities for learning and development, while inadequate restrictions may increase the risk of harmful screen use. However, these key challenges may be further influenced by context-specific factors such as socioeconomic conditions, digital access, parental digital literacy, and cultural norms. Together, these findings highlight the complexity of managing adolescents’ SV across diverse settings, particularly in low- and middle-income countries, including Bangladesh, where digital technology is rapidly expanding [13]. In such circumstances, parents’ and caregivers’ demanding schedules often limit their ability to monitor both the amount of time children spend on screens and the nature of the content to which they are exposed [13].

Parents face the key challenge of balancing the need to protect children from internet addiction and online risks, including cybercrime, while also fostering the digital skills needed for education and future opportunities [14]. Research in a variety of settings emphasizes the issue. For instance, even though they were aware of the detrimental effects of screen time, some Canadian parents (85% of mothers) claimed that they valued screen viewing as a behavior management technique or by employing SV as a “electronic babysitter” to handle their household obligations [15]. The previous studies conducted in Bangladesh, restrictive schedules and safety concerns limited children’s opportunities for physical activity, especially for girls [16,17]. In the developed country context, self-interests such as doing tasks undisturbed, creating me-time, regulating children’s behavior, avoiding arguments, and being able to use devices themselves were the core findings [18].

In Kuwait, parents use family-based strategies to manage sedentary behavior, including encouraging children to join health clubs, using reward techniques, arranging regular sports competitions, and establishing entertainment centers in each region [19]. In the context of UK, Parents identified that school-based interventions, alongside the parental role, as important for preventing excessive use [20].

Despite the growing body of evidence, there remains a lack of research exploring how parents in low-and middle-income countries (LMICs) navigate these challenges. In Bangladesh, where digital access is rapidly expanding but parental knowledge, guidelines, and protective structures for the limitation of use of devices are limited, understanding these dynamics is particularly important This study aimed to explore the challenges parents face in supervising and restricting children’s SV from the perspectives of parents, teachers, and adolescents aged 12–14 years in urban Dhaka, Bangladesh, and to identify strategies that may help achieve a healthier balance between digital engagement and children’s overall well-being.

## Materials and methods

### Study design

This study employed an exploratory qualitative design to explore the perspectives and experiences of parents, teachers, and adolescents regarding children’s SV. This exploratory qualitative design as a follow-up to our previous quantitative study to provide a deeper understanding of the findings within the local context [21].

The qualitative component explored the perspectives of parents, teachers, and adolescents on the challenges of managing children’s SV and identified context-specific factors and potential strategies to promote healthier screen use.

### Data collection method

Data were collected using multiple qualitative data collection methods. In-depth interviews (IDIs) were conducted with parents, teachers, and adolescents to explore their experiences and perceptions of children’s screen viewing. Focus group discussions (FGDs) were conducted with parents and adolescents to capture shared experiences, challenges, and recommendations for healthier screen use. The use of both IDIs and FGDs enabled the collection of complementary individual and group perspectives.

### Study settings

The study was conducted in Dhaka, the capital and largest metropolitan city of Bangladesh, characterized by high population density, rapid urbanization, widespread internet connectivity, and extensive access to digital devices. Dhaka North and Dhaka South City Corporations represent urban areas with comparatively higher socioeconomic status, greater availability of private and public educational institutions, and high penetration of smartphones, tablets, and internet services.

### Study participants and sampling

During the school selection, we also considered, with the kind permission of the school authorities, the study included three participant groups: parents, students, and teachers. Students aged between 12–14 years, who were in classes 6–10, and who used more than two hours of screen time each day as the qualifying criteria for sample inclusion [10]. Smartphones, tablets, laptops, personal computers (PCs), televisions, and portable gaming devices were considered acceptable gadgets. However, students who might have pre-existing conditions, such as physical or psychological impairment, were excluded from the study. Parents were eligible if they were the primary caregivers of a school-going child with excessive digital device use and were responsible for setting household rules and boundaries regarding screen time. establishing household screen-time rules. Students were recruited separately from the selected schools based on the study inclusion criteria. This non-dyadic approach allowed the collection of individual perspectives from parents and students without linking responses to specific parent–child pairs. Teachers from the selected schools were recruited to provide their perspectives on students’ screen use and potential management strategies. Teachers were interviewed independently to develop potential strategies for managing screen time, as they play a significant role in student development and education.

Parents, teachers and students were required and interviewed independently. Parents and students were not enrolled as parent-child dyads, allowing individual perspectives to be explored without linking responses between family members.

A heterogeneous sampling technique was utilized for IDIs, while a homogeneous technique was applied for FGDs [22]. IDIs with students and parents were conducted separately after school hours to ensure sufficient time and minimize distractions. FGDs were also organized separately with students and parents, considering diverse classes, ages, and sexes to capture a wide range of perspectives. Participants were seated in a circle to encourage interaction, and all discussions were audio recorded. Nobody was allowed in the interview room during the sessions, ensuring confidentiality. All interviews were conducted in Bangla, the national language of Bangladesh. and privacy was maintained throughout the interviews. A total of 12 IDIs and six FGDs were conducted across six schools, which was sufficient to reach thematic saturation. Table 1 provides detailed information about the study participants below.

**Table 1:** Interview method and sampling.

Method	Type of schools		Type and number of participants		
	Bangla	English	Children	Parents	Teacher
In-depth interviews (IDIs)	6	6	5	3	4
Focus Group Discussions (FGDs)	3	3	20	24	–

### Operational definitions

**Screen Viewing (SV):** The time students spend using digital devices for any purpose, including educational, recreational, or social activities.

**Excessive screen time:** Excessive screen time is commonly defined as recreational screen use exceeding 2 hours per day, which the WHO considers part of prolonged sedentary behaviour that should be limited and replaced with physical activity for optimal health [10]

**Digital Device:** Any electronic device that allows access to digital content, such as smartphones, tablets, laptops, televisions, computers, and game consoles.

**Digital Platform:** Online services or applications accessed through digital devices, including websites, social media, educational tools, and entertainment platforms.

**Digital Literacy:** The ability to effectively and safely use digital devices and platforms, including understanding content, managing screen time, and navigating online resources.

**Outdoor Activity:** Any physical or recreational activity performed outside the home, including sports, games, creative play, and other forms of physical exercise.

**Peer Interaction:** Social engagement and communication with other students, either in-person or virtually, fostering social skills and relationships.

**Inappropriate Content:** Digital material that is harmful, age-inappropriate, or culturally unacceptable, such as violent, sexual, or drug-related content.

**Social Safety:** The provision of a secure and supervised environment for students to engage in activities, reducing exposure to harm and promoting physical, emotional, and social well-being.

### Data collection and quality checking

Data collection was carried out between January 2024 and April 2024. Six schools were purposively selected, including three Bangla-medium and three English-medium schools, to capture variation in educational systems and student backgrounds. English-medium schools in Dhaka generally cater to higher-income families, while Bangla-medium schools include students from diverse socioeconomic strata. The selected schools were located in residential and mixed-use urban neighborhoods where children are frequently exposed to digital screens for both educational and recreational purposes. Data were collected using a combination of audio recording and detailed field note-taking to ensure accurate documentation of participants’ responses and contextual observations during interviews and focus group discussions. Three separate semi-structured interview guides were developed for students, parents, and teachers based on the study objectives and a review of the relevant literature [6–8,15,19]. The guides explored participants’ experiences, challenges, and recommendations related to monitoring students’ screen time [9]. All study tools were translated from English into Bangla before data collection. All tools were pre-tested with 6 non-study participants to ensure clarity and appropriateness.

Each in-depth interview (IDI) lasted approximately 40–60 minutes, while each focus group discussion (FGD) lasted up to 90 minutes. The interviews were conducted in the teachers’ common room after school hours, providing a quiet and comfortable environment where participants could speak openly about the study topics. After informed written consent, the FGDs were conducted on the respective school premises in a quiet and private setting approved by school authorities. Interviews were conducted by a Senior Research Officer with more than five years of qualitative research experience, assisted by a Field Research Assistant with over three years of experience who took field notes. Both received training before data collection. A mental health expert monitored the overall interview process to ensure appropriate interviewing techniques, ethical conduct, and neutrality, but did not participate in the interviews or observe individual participants directly.

### Data analysis

Data analysis began by organizing the interview transcripts and field notes. To maintain confidentiality, all audio recordings were assigned unique identifiers before transcription. Interviews and FGDs were transcribed verbatim in Bangla and reviewed by the principal investigator for accuracy. Field notes and observations recorded during data collection were also incorporated into the analysis.

The transcripts were read repeatedly to achieve data familiarization. An inductive thematic analysis approach was used to allow themes to emerge directly from the data rather than being predetermined [23]. Initial codes were developed independently from the transcripts and then discussed among the research team. Through regular meetings, the team reviewed, refined, merged, and redefined the codes until consensus was reached, resulting in the development of a shared codebook. Related codes were grouped into categories and subsequently organized into themes and sub-themes that reflected participants’ experiences and recommendations regarding SV.

To enhance reflexivity, the research team regularly discussed their assumptions, interpretations, and potential biases throughout the coding and theme development process. Differences in interpretation were resolved through discussion and consensus, ensuring that the findings were grounded in participants’ accounts rather than individual researchers’ perspectives. This reflexive process was particularly important because an inductive approach was used.

The identified themes were then interpreted using a modified socioecological model [24–26] with findings organized into three interconnected levels: individual and family, institutional and community, and national policy. Four themes were identified for the challenges, while recommendations were presented according to these three socioecological levels.

To enhance the rigor and trustworthiness of the findings, we used multiple strategies, including investigator triangulation through collaborative coding, methodological triangulation across IDIs and FGDs, and data source triangulation involving parents, students, and teachers [27].

### Ethical approval

The study protocol was approved by the Institutional Review Board (IRB) of icddr,b (Protocol No: PR-22002). Informed written consent was obtained from parents and teachers. Assent was obtained from the students themselves, as they were aged above 11 years, prior to the interviews. All the respondents were informed in Bengali about their rights related to their voluntary participation in the study, and they could withdraw from the interview at any time during the interview. Interviewers of this study clearly conveyed to the respondents the objectives of the study, reasons for their participation in the study, their role, risks, benefits, anonymity, and confidentiality, including the rights not to participate or withdraw. After getting acquainted with all these issues, the respondents decided whether to participate in this study. Confidentiality during and after data collection was ensured, and no identifiable information was used that may expose the identity of the participants. Only the research team members have access to the collected data, which was stored securely. After the interview, each participant received compensation equivalent to BDT 370 (USD 3) to reimburse their time and transportation expenses.

## Results

### Background characteristics of participants

The demographic characteristics of the participants are illustrated in Table 2. All the students were between 12 and 14 years of age, and 56% of them were males. The majority of them (48%) were students of class 8, hailed from Bangla-medium schools (64%), and belonged to nuclear-type families (64%). Most of the fathers of students are involved in business (64%), whereas mothers are housewives (92%). The majority of the students (60%) had at least one device, and a substantial percentage (92%) were Wi-Fi users. They were found to be using multiple devices, meaning 100% used smartphones, followed by 76% had access to smart TVs.

**Table 2:** Participant demographics (N = 25).

Variable	Frequency (n)	Percentage (%)
**Sex**		
Male	14	56.0
Female	11	44.0
**Age (in years)**		
12	12	48
13	10	40
14	3	12
**Studying class/ grade**		
6th	2	8.0
7th	2	8.0
8th	12	48.0
9th	8	32.0
10th	1	4.00
**School type**		
Bangla Medium	16	64.0
English Medium	9	36.0
**Occupation of father**		
Business	16	64.0
Service holder	3	12.0
Labor	2	8.0
Skilled worker	1	4.0
Others	3	12.0
**Occupation of Mother**		
Housewife	23	92.0
Skilled worker	2	8.0
**Type of family**		
Nuclear	16	64.0
Extended	9	36.0
**Device owned by participant**		
Yes	15	60.0
No	10	40.0
**Type of internet access**		
Wi-Fi or Broadband	23	92.0
Mobile data	2	8.0
**Type of device used by the participant [multiple response]**		
Smart phone	25	100.0
Smart television	19	76.0
Laptop	11	44.0
Tablet	9	36.0
Computer	8	32.0

The thematic analysis identified two major themes emerging from the perspectives of parents, students, and teachers. Theme 1 focused on “Challenges faced by parents in monitoring children’s screen use”, describes the barriers encountered at the individual and family and institutional and community levels. Theme 2, “Recommendations to reduce excessive screen time among children”, presents participants’ recommendations at the individual and family, institutional and community, and national policy levels. Integrating the perspectives of parents, students, and teachers provided a comprehensive understanding of the challenges associated with excessive screen use and the strategies recommended to address them. Illustrative quotations are presented to support each theme. As no substantial differences were observed between data obtained from IDIs and FGDs, the findings are presented collectively. Including multiple stakeholder perspectives allowed triangulation of findings and provided a comprehensive understanding of excessive digital device use within the school and family context. An overview of the challenges identified and the recommendations proposed is presented using illustrative verbatim quotes. As no substantial differences were observed between responses from IDIs and FGDs, the findings are reported collectively without specific participant identifiers.

## Challenges faced by parents in monitoring children’s screen use

### Individual and family level

#### Reduced opportunities for offline social interaction and family engagement.

Parents expressed concerns about the safety of outdoor spaces, particularly for girls, which limited opportunities for students to interact with peers. Parents prevented their children from going outside or socializing with lesser-known peers out of fear of sexual abuse, even teasing, and misguidance by strangers. Consequently, students had few friends and spent most of their leisure time on digital devices.

*“Where will our children play in Dhaka city? As we live in an apartment, sometimes there are rules or restrictions on going to the rooftop. We are primarily a home-centered family and do not allow our children to mix with others due to concerns about social safety. We do not even know our next-door neighbor due to our lack of time and security. I think there is no other reason than these.” (* ***FGD; Parent; Bangla medium school)***

Most parents reported that very few children had siblings to play with and that both working and non-working parents struggled to spend sufficient time with their children due to busy schedules. Some parents noted that children felt more comfortable communicating with classmates than with family members.

*“sometimes we also cannot give enough time, as her mom stays occupied, and I also stay busy with school activities. What will she do? There is no access to play outside. She might spend her leisure time gossiping with others if we lived in a joint family, but as we live in a nuclear family, there is no scope to do that, either. That’s why they are addicted to mobile phones. We also cannot say anything considering all of these issues.”* ***(IDI; Parents; Bangla medium school)***

Students also highlighted similar issues about a lack of interaction with their parents owing to their tedious schedules. One student conveyed,

*“My father sat with me to spend time, but within two hours, he checked his E-mail numerous times. During that time, I also felt a similar urge to use my device as my father checked his inbox messages.” (****FGD; Student; Bangla medium school***)

Both parents and students reported restricted access to outdoor play due to the lack of playgrounds and concerns over social insecurity, including kidnapping, sexual harassment in public spaces, or sexual abuse. In nuclear families, children often had limited opportunities to interact and engaged with their cousins or age-matched peers, making digital devices their primary source of leisure and recreation.

*“Even when cousins are together, they’re all on their devices. Each cousin has one in hand. Instead of chatting, they just sit with their devices. We, on the other hand, can talk for hours after the end. But our kids can’t. After talking for a little while, they’ll say they need a break, which means they want to check their phone, and then again, they go back to the phone. Managing them is much tougher.” (* ***FGD; parents; English medium school*** *)*

### Limited parental capacity for effective monitoring and digital literacy

Teachers reported that some parents lacked the knowledge and skills to effectively monitor their children’s digital device use. They believed that this limited monitoring contributed to children’s exposure to age-inappropriate online content, which could negatively affect behavior, communication, and emotional well-being.

*“Due to digital device usage, children are accessing inappropriate content and becoming addicted to it, with younger students watching adult material and mimicking adult behaviors. This leads to a lack of communication, with interactions moving to platforms like Facebook. When breakups occur, they struggle to cope, often resulting in depression, as they are too young to handle such situations”. (* ***(IDI; Teacher; English medium school)***

Students stressed that supervision could prevent access to unsafe websites, pornography, gambling, and the use of abusive language. They indicated that awareness of parental monitoring would discourage engagement in risky or inappropriate online behaviors.

*“Children would be conscious if they knew that their activities on devices would be supervised or monitored by their parents, and then they would be discouraged from doing any wrong activities using those devices.” (****FGD; Student; English medium school***)

Parents acknowledged that limited digital literacy constrained their ability to monitor children effectively. Children were often more technologically skilled and could bypass restrictions or age limits, leaving parents feeling unable to enforce boundaries.

*“He is expert on device use than me. He is an expert in IT. He can hack our software to open it, that’s why I left to use age limit apps.”* ***(FGD; Parent; English medium school)***

Students also reported creating fake accounts to access games or social media apps restricted by age settings.

*“There are many children who lie by increasing their age using fake profile accounts.” **(FGD; Parent; English medium school)***

### Institutional and community level

#### Educational reliance on digital devices without structured guidance.

Parents highlighted the lack of sufficient guidance in the newly introduced education system, which increased students’ reliance on mobile phones and the internet for academic purposes. They reported that teachers often did not provide adequate explanations in class, pushing students to depend on online resources. While parents provided mobile phones to support their students’ learning, they observed that students frequently misused the devices for non-academic purposes, such as playing games, under the pretext of studying.

*“The books that are provided as per the new education system, there are no guidelines. So, students need to depend on using a mobile phone to get help from the internet. Because teachers nowadays do not explain the topics in the classroom, they tell their students to complete them at home. So, when students are given mobile phones for study, it is seen that sometimes they start to play games as an excuse for study.”* ***(FGD; Parent; Bangla medium school)***

Students reported that teachers frequently encouraged them to use Google and other online resources to complete classroom assignments and obtain additional information. Many participants explained that their parents were often unable to help them with the updated curriculum, which increased their reliance on internet-based resources for academic learning. They also described creating online groups with classmates to discuss lessons, share study materials, and collaborate on assignments through digital communication platforms. These online resources and communication platforms were considered important tools for supporting their learning and completing school-related tasks.

*“There are many topics in the books that I do not know how to solve, and even my parents cannot help me with that, so they suggested I search Google. Actually, it often happens that I take permission from my mom to use the mobile phone for study purposes, but I do not use it for study purposes. I usually watch short videos. Short videos or reels are more fun than studying”.* ***(FGD; Student; Bangla medium school)***

All parents emphasized that as a result of excessive device use, their students’ academic performance is directly impacted, resulting in lower grades.

*“My son’s roll number is dropping from 20 to 50. He only spends one hour on coaching and private tutoring. The rest of the day, he’s glued to his mobile.” (FGD; Parents; Bangla medium school)*

Teachers expressed concerns about the increasing dependence on digital platforms from grade six onwards. While acknowledging the benefits of technology for learning, they noted that many students lacked the maturity to use devices responsibly. Teachers also observed that group work often created inequalities, where a small number of students bore the entire burden, while others misused devices for unrelated activities. They emphasized the importance of parental supervision and awareness to ensure productive screen use.

*“If we ask students to create content in groups, say we form a group of ten, usually only two out of the ten end up doing the work. The remaining eight, instead of contributing, often get busy with unrelated activities on their phones or laptops, under the pretense of preparing content. This puts all the pressure on the two active members. As a result, those two, who are often the group leaders and active students across most subjects, bear the entire burden, leading to increased stress on them.”* ***(IDI; Teacher; English medium school)***

### Risks related to unsafe and unsupervised digital exposure

Parents reported that students were frequently exposed to unwanted sexual content through advertisements on platforms such as TikTok, YouTube, Facebook, and online games. Many parents found it challenging to monitor students’ device use due to time constraints and discomfort discussing sexual topics, despite recognizing the importance of guidance during childhood. Exposure to such content caused worry and anxiety, as parents feared it might trigger curiosity or inappropriate behaviors.

*“I was astonished after seeing what my son was viewing; when I asked my son about that obscene video, my son replied that he always plays games, but such obscene videos sometimes appeared on his screen as advertisements. But after that incident, I can’t stop thinking negatively about him.”* ***(FGD; Parents; Bangla medium school)***

Students confirmed that sexual or inappropriate content sometimes appeared unintentionally while using digital platforms, and they were unable to skip certain advertisements. They reported that exposure increased curiosity and encouraged further exploration, underscoring the risks of unsupervised screen time.

*“Sometimes a bad video pops up, and once you watch it, you feel like watching more. Later, it causes physical problems… the body feels weak. I mean, in the middle of a video, like for 30 seconds, these kinds of clips appear. Actually, they’re just 3-second videos that come up automatically. I was playing a game when those ads popped up, and my father saw it. After that, he didn’t give me the phone for two or three days.” (****FGD; Parents; English medium school***)

### Recommendations to reduce excessive screen time among children

The recommendations from parents, teachers, and students were organized using a socio-ecological framework, which recognizes that excessive screen use is influenced by multiple, interconnected factors at the individual, family, institutional, community, and national policy levels. Using this framework, the study examined screen-use behaviors across different layers of influence rather than focusing on a single level. After thematic analysis, the recommendations were grouped into three broad and interconnected levels: (1) individual and family, (2) institutional and community, and (3) national policy. Participants emphasized that reducing excessive screen time in a meaningful and sustainable way requires coordinated actions across all of these levels.

### Individual and family level

At the individual and family level, participants strongly emphasized the role of parent–child relationships in shaping children’s screen use behaviors. Parents highlighted the importance of spending quality time with children through shared activities such as conversations, morning walks, picnics, interactive games, and cooking together. These interactions were viewed as essential for strengthening emotional bonds and naturally reducing children’s dependence on digital devices. Parents’ own screen habits influence children’s behavior, reinforcing the need for positive family engagement.

*“My father sat with me to spend time, but within two hours, he checked his e-mail numerous times. During that time, I also felt a similar urge to use my device as my father checked his inbox messages.”* ***(FGD; Student; Bangla medium school)***

Developing self-regulation skills in children was another key recommendation. Rather than relying solely on strict parental control, parents and teachers suggested encouraging children to understand and manage their own screen habits. Emphasis was placed on fostering self-discipline, awareness of health consequences, and balanced daily routines, enabling children to voluntarily limit prolonged screen use. Counseling was repeatedly identified as a supportive strategy for both children and parents. Participants felt that counseling sessions could help address screen-related issues alongside academic stress, sleep deprivation, and behavioral concerns.

*“The time that is being spent on the mobile means they are not getting enough rest. Of course, that has an effect. That’s why I think counseling is necessary. Because through counseling, whether it’s about mobile use or something else, the issue will come up. They will hear things like, ‘Don’t do this,’ or, ‘It’s better if you avoid that,’ and then the topic of studies will also come up. One way or another, some advice will be given.” (* ***FGD; Parent; English medium school)***

Parents noted that overreactive or punitive approaches often backfired, increasing children’s curiosity and resistance.

*“Children would be conscious if they knew that their activities on devices were being supervised by their parents. Then they would be discouraged from doing inappropriate activities using those devices.”* ***(FGD; Student; English medium school*)**

Instead, logical guidance, consistent communication, and parental counseling on how to support children’s digital habits were seen as more effective. Parental should supervise their children with logical guidance. Participants also recommended promoting alternative, engaging activities to replace screen viewing. These included outdoor play, sports, reading, creative arts and crafts, and other hobbies that provide enjoyment and cognitive stimulation. Such alternatives were perceived as essential to shift children’s attention away from devices while supporting holistic development.

*“I have the same opinion as the others. One more recommendation I would make is that, since study time is spent on mobile devices, children then look for suggestions on how to excel in exams in a short amount of time. Because of this, they become more concerned with just performing well in exams rather than actually learning. That’s a major harm. Another point is that devices will be used anyway, so along with the children, the parents also need to be made aware. If we overreact, then whatever is forbidden is exactly what they will want to do. In that case, if parents behave logically, it will help, because sometimes their overreaction can have a negative impact. That’s why it would be good to have a counseling session for parents as well, on how to guide their children. At the school level, too, parents could be made more aware of how to behave with their children.” (* ***FGD; Parent; English medium school)***

### Institutional and community level

At the institutional level, schools were viewed as critical settings for reducing excessive screen exposure. Participants recommended minimizing unnecessary online activities within the curriculum and replacing them with offline, interactive teaching approaches wherever possible. When digital tools were required, parents and teachers suggested that device-based tasks should be completed under direct teacher supervision, with schools providing the necessary devices rather than relying on students’ personal phones.

“The books under the new education system do not provide enough guidance. Students have to depend on mobile phones to search the internet, and once they get access to a phone for studying, they often end up playing games instead.” **(FGD; Parent; Bangla medium school)**

Schools were also encouraged to play a proactive role in awareness building, through joint programs involving parents, teachers, and students that address the harmful physical, psychological, and academic consequences of excessive screen use. Increasing extracurricular opportunities such as sports, physical exercise, creative games, and cultural activities was seen as an effective way to divert students’ attention from screens. Several participants emphasized the need for school-based psychological support, recommending the appointment of a mentor or psychologist to address device overuse, provide counseling, and support students’ mental well-being. Additionally, limiting ICT and computer studies to supervised use within school premises was suggested to prevent unsupervised and excessive exposure.

*“Due to digital device usage, children are accessing inappropriate content and becoming addicted to it. They become isolated, and when problems arise, they often become depressed because they are too young to cope.”* ***(IDI; Teacher; English medium school)***

At the community level, participants highlighted the importance of improving access to safe outdoor spaces. Students and parents emphasized the need for playgrounds in every neighborhood, along with security measures such as guards and surveillance cameras, to ensure children’s safety.

*“Every area needs to have a playground. There should be guards around the field and cameras installed along the area’s borders.” (* ***FGD; Students; English medium school)***

Creating secure and supervised environments was seen as essential for encouraging outdoor play, physical activity, social interaction, and the development of life skills, all of which contribute to reduced screen time. Community-based awareness initiatives, including programs led by schools, NGOs, and local organizations, were also recommended. These initiatives could educate parents about the mental health risks associated with excessive mobile phone use and promote healthier digital practices at home.

*“Schools could arrange programs to make parents aware of how harmful mobile phones can be. Such initiatives could also be taken through different NGOs. These programs could highlight the mental health problems associated with mobile phone use and explain them sincerely to parents. Various awareness sessions could also be conducted.” (* ***FGD; student; Bangla medium school)***

### National policy level

At the national level, participants described excessive screen use as a growing public health concern requiring government leadership and policy action. Parents recommended the development of digital restriction mechanisms in collaboration with the ICT Department of Bangladesh to prevent children’s exposure to age-inappropriate content. Parents alone cannot ensure safe digital environments, so national technological safeguards are needed.

*“He is more expert in using devices than I am. He can bypass our software restrictions, so I eventually stopped using age-limit applications.”* ***(FGD; Parent; English medium school)***

Suggested strategies included web and content filtering systems, child-friendly digital platforms, and software or applications that offer age-appropriate content while enabling parental monitoring. “There are many children who lie about their age by creating fake profile accounts.” (FGD; Parent; English medium school)

National digital regulations, age-verification mechanisms and Government-led awareness campaigns were also strongly emphasized. Participants suggested using widely accessible platforms such as television, social media, and child-focused media to disseminate messages about the risks of excessive screen time. Integrating these messages into popular educational cartoons and programs was viewed as particularly effective for reaching children.

Incorporating digital health education into the formal school curriculum was another major recommendation. Participants proposed adding dedicated chapters to ICT textbooks that address the negative impacts of excessive screen use and promote healthy digital habits. Teachers further suggested including age-specific screen time guidelines, such as standard use practices within textbooks to formalize recommended limits, aligning educational content with international and national regulatory guidance. Overall, these recommendations reflect a collective call from parents, teachers, and students for multi-level, coordinated interventions that combine family engagement, school-based strategies, community support, and national policies to address the growing challenge of excessive screen use among children.

*“This has actually become a national issue now since almost all children are facing this problem. If the government includes these issues in the curriculum, it carries more importance than just us parents saying it. This could be done through NGOs or schools as well. The media we have could also be used to raise awareness. For example, we were taught many things through the Meena cartoon. In the same way, cartoons could be made to show the negative sides of mobile use, so that children themselves would be attracted to watch and learn.”* ***(FGD; Parents; English medium school))***

## Discussion

This study explored the challenges of monitoring excessive screen use among school-going children and identified multi-level recommendations from parents, teachers, and students in urban Bangladesh. Parents and students highlighted that the new education system encourages independent learning and reliance on digital platforms. Many parents reported that teachers provide insufficient guidance in classrooms, pushing students to seek help online. While digital devices facilitate learning, parents observed frequent misuse, such as playing games under the guise of studying. Students’ dependence on online resources and group assignments via social media further exacerbated the challenge, as distractions from short videos, reels, and other entertainment content were commonly reported. Study found digital media negatively impacts education by causing interaction issues, academic failure, and miscommunication [28]. Another study conducted in Nepal shows that online video games can develop independent study skills and autonomous learning abilities in secondary students, but parents perceive potential negative health impacts if not educationally designed [29]. Teachers noted that younger students often lack the maturity to use devices responsibly, emphasizing the importance of parental supervision to ensure productive and safe digital learning.

Parents’ concerns about social safety, especially for girls, limited children’s outdoor play and peer interactions. This finding has been incorporated into how environmental and social safety concerns constrain children’s participation in alternative recreational and social activities, potentially increasing reliance on digital screens. Several studies conducted in Bangladesh focused on outdoor play have consistently reported similar findings, highlighting social safety, particularly for girls, as a major concern [16,17]. Fear of eve-teasing, sexual abuse, or misguidance led parents to limit their children’s opportunities for outdoor activities and physical play. In nuclear families, the scarcity of siblings or cousins further reduced opportunities for interaction, leaving digital devices as the main avenue for leisure. Limited family time and communication gaps were also evident, as parents’ busy schedules hindered meaningful engagement with their children. Children reported feeling more comfortable interacting with peers than with parents, a phenomenon that may contribute to increased screen dependency. These findings align with previous research conducted in Bangladesh and similar contexts, e.g., India, indicating that urban children often face constrained outdoor play due to safety concerns and that parental availability plays a critical role in managing SV [30,31].

Parents and teachers reported concerns about children’s exposure to inappropriate content on popular digital platforms, particularly through advertisements. While this did not directly constitute a challenge in managing the duration of screen use, it influenced parental vigilance and monitoring practices, shaping the strategies they employed to guide children’s digital media engagement. Previous quantitative research suggests that parent-child relationships, socio-economic conditions, parents being unaware of children’s SV, and children owning devices by themselves were major contributive elements for viewing violent or pornographic content [32]. Students confirmed that exposure to sexual content often increased curiosity and encouraged further exploration, reinforcing the risks of increase digital use. A relevant qualitative paper explored the concerns of parents to exposure to harmful content [33]. Teachers highlighted that unsupervised access could lead to behavioral and emotional consequences, including imitation of adult behaviors, reduced communication skills, and vulnerability to online risks. Students emphasized that awareness of parental monitoring could discourage engagement in unsafe or inappropriate activities.

A significant barrier to effective supervision was parents’ limited digital literacy. The home technology environment is imperative in this context. Various studies report that children of this generation are exposed to a wide range of toys, multimedia, devices, and gadgets from a very early age, which enable and enhance them to efficiently operate them [34,35]. In contrast, many parents reported being less technologically skilled than their children, who could bypass restrictions or manipulate age verification systems. Students also reported creating fake accounts to access games or social media apps restricted by age. These results reflect global evidence showing that parents’ digital skills are critical for establishing boundaries and guiding safe online behaviors [36].

Furthermore, this study focused on some recommendations that the caregivers suggested in order to reduce SV in the young ones. They mentioned that schools should take some core initiatives. A cluster-randomized controlled trial to evaluate the effectiveness of school-based intervention on SV in Bangladesh was conducted between two groups, reporting that there were promising changes in children when physical activity was encouraged and SV was reduced [37].

Points such as implementation of national guidelines, offering alternatives to SV, increased family time, development of self-regulation in children, and ensuring a safer environment were also discussed extensively. Relevant qualitative exploration enumerated some solutions for the government to implement, such as monitoring inappropriate programs for the young ones and blocking them; using social media to explain the detrimental impacts of overuse of devices; establishing more sports clubs; ensuring computer classes at schools, which would include lessons regarding optimum SV [19]. Another paper mentioned that strong bonding between children and parents beneficially impacts the SV of children [24]. Furthermore, similar research recommended that parents need to enhance their knowledge about social media and associated risks to protect their children [25]. In addition, a systematic review revealed that weaker parent-child bonding and an unhealthy familial environment were linked to gaming addiction in children [26]. Also, single-parent families were detected to have greater internet abuse in young ones.

It is essential to consider the deleterious impacts of excessive SV on children. The implications of this paper are based on the challenges encountered by the caregivers and their thoughtful proposals to mitigate this condition. The balance between the real and virtual worlds is difficult to maintain, but it is mandatory. While it is necessary to maintain the guidelines of children’s SV flexible in order to go with the flow of the rapidly changing technological ambience, certain guidelines need to be implemented to make sure caregivers understand how much SV is excessive. It can be encouraged to develop specific policies and programs on multiple levels to eliminate the difficulties faced with regard to monitoring SV and promote alternative activities and options to foster a healthier life for children.

## Conclusion

Urban digitalization creates many challenges for caregivers in managing children’s screen use. These include the increasing use of digital devices for schoolwork and limited digital knowledge among caregivers. Safety concerns in outdoor environments also reduce opportunities for children to play outside, while selecting age-appropriate digital content remains difficult. This study aimed to understand the challenges parents face when their children use electronic screens excessively and to gather parents’ suggestions on how digital devices can be used more healthily and effectively for students. The findings highlight the need for clear policy guidelines that involve schools and are supported by national-level strategies. Creating a supportive home environment and providing safe, child-friendly spaces are also important. Future research should focus on long-term and intervention-based studies to assess school-based digital literacy programs, caregiver training, and improvements in urban environments to support balanced screen use and healthy child development. This recommendation approach facilitated a structured interpretation of participants’ perspectives and highlighted the need for coordinated interventions rather than isolated actions. Organizing the findings within this framework ensured clarity, coherence, and relevance for policy and program development.
