# Supplementary material for: Parental monitoring challenges and recommendation for managing excessive screen use among students aged 12–14 years in Dhaka, Bangladesh: A qualitative exploration
Source: PLoS One. 2026 Oct 5;21(10):e0359518. doi: 10.1371/journal.pone.0359518 (PMC13637862; doi:10.1371/journal.pone.0359518)
Supplement: Supplementary file 1 [file pone.0359518.s001.pdf]

PLOS ONE

# Parental monitoring challenges and recommendation for managing excessive screen use among adolescents aged 12–14 years in Dhaka, Bangladesh: a qualitative exploration

--Manuscript Draft--

| | |
|---|---|
| **Manuscript Number:** | PONE-D-25-02782 |
| **Article Type:** | Research Article |
| **Full Title:** | Parental monitoring challenges and recommendation for managing excessive screen use among adolescents aged 12–14 years in Dhaka, Bangladesh: a qualitative exploration |
| **Short Title:** | Challenges and recommendations for screen use in adolescents: a qualitative study |
| **Corresponding Author:** | Shahria Hafiz Kakon, MBBS,MPH,PhD fellow<br>ICDDR B: International Centre for Diarrhoeal Disease Research Bangladesh<br>Dhaka, BANGLADESH |
| **Keywords:** | Screen viewing; device; Adolescents; gadgets; social media; Challenges; recommendations; focus group discussion; in-depth interviews and key informant interviews. |
| **Abstract:** | Background : Of late, social media and recreational internet use are undeniable. The proper use of devices for children is critical; overindulgence leads to a negative impact on children in many areas of their lives. Objectives: This study investigated the challenges that parents face when their children use electronic digital screens excessively. It also consists of recommendations from the participants for the best use of digital devices by adolescent children. Methods: The study used a qualitative approach and revealed the obstacles parents encountered to ensure the quality and duration of screen use, as well as some strategies for regulating this element. Data were collected from January 2024 to April 2024. Adolescents aged between 12 and 14 attended six different English and Bangla medium schools in Dhaka; and their parents and teachers were the participants in this study. The data collection methods were focus group discussions, in-depth interviews, and key informant interviews. Data were analyzed using the inductive method and presented in a narrative qualitative manner. Results: Results displayed 25 children between classes 6-10, where 56% were males. The core challenges were the recent excessive use of devices for social media and recreational internet besides learning and academics, ensuring safe and proper environments in the neighborhoods for outdoor play, confirming the viewing of age-appropriate content and lastly, a deficit of digital literacy in the parents. Recommendations received were school-based interventions, national-level policies, development of self-regulatory behavior in children, provision of secured infrastructures, enhancing the parent-child bond and offering interesting options to divert the children from screens. Conclusions: The study findings provide a better understanding of the challenges of overuse of devices and recommendations among the population. Effective guidelines for managing students' screen use require the participation from schools, parents, legislators and students themselves. Further research is required to determine the most effective interventions. |
| **Order of Authors:** | Shahria Hafiz Kakon, MBBS,MPH,PhD fellow |
| | Nahian Sultana |
| | Bidhan Krishna Sarker |
| | Tanjir Rashid Soron |
| | Rashidul Hoque |
| | Fahmida Tofail |
| **Additional Information:** | |
| **Question** | **Response** |
| **Financial Disclosure** | Yes |

Powered by Editorial Manager® and ProduXion Manager® from Aries Systems Corporation

| | |
|---|---|
| Enter a financial disclosure statement that describes the sources of funding for the work included in this submission. Review the submission guidelines for detailed requirements. View published research articles from *PLOS ONE* for specific examples.<br><br>This statement is required for submission and **will appear in the published article** if the submission is accepted. Please make sure it is accurate.<br><br>**Funded studies**<br>Enter a statement with the following details:<br>• Initials of the authors who received each award<br>• Grant numbers awarded to each author<br>• The full name of each funder<br>• URL of each funder website<br>• Did the sponsors or funders play any role in the study design, data collection and analysis, decision to publish, or preparation of the manuscript?<br><br>Did you receive funding for this work? | |
| Please add funding details.<br>as follow-up to "**Financial Disclosure**<br><br>Enter a financial disclosure statement that describes the sources of funding for the work included in this submission. Review the submission guidelines for detailed requirements. View published research articles from *PLOS ONE* for specific examples.<br><br>This statement is required for submission and **will appear in the published article** if the submission is accepted. Please make sure it is accurate. | Bangladesh government awarded project named Mujib 100 research Grant for women. I have received this award on 2022. Funded was $ 43,250 for 2 years project |

| | |
|---|---|
| **Funded studies**<br>Enter a statement with the following details:<br>• Initials of the authors who received each award<br>• Grant numbers awarded to each author<br>• The full name of each funder<br>• URL of each funder website<br>• Did the sponsors or funders play any role in the study design, data collection and analysis, decision to publish, or preparation of the manuscript?<br><br>Did you receive funding for this work?" | |
| Please select the country of your main research funder (please select carefully as in some cases this is used in fee calculation).<br>as follow-up to "**Financial Disclosure**<br><br>Enter a financial disclosure statement that describes the sources of funding for the work included in this submission. Review the submission guidelines for detailed requirements. View published research articles from *PLOS ONE* for specific examples.<br><br>This statement is required for submission and **will appear in the published article** if the submission is accepted. Please make sure it is accurate.<br><br>**Funded studies**<br>Enter a statement with the following details:<br>• Initials of the authors who received each award<br>• Grant numbers awarded to each author<br>• The full name of each funder<br>• URL of each funder website<br>• Did the sponsors or funders play any role in the study design, data collection and analysis, decision to publish, or preparation of the manuscript?<br><br>Did you receive funding for this work?" | BANGLADESH - BD |
| **Competing Interests** | The authors have declared that no competing interests exist. |

| | |
|---|---|
| Use the instructions below to enter a competing interest statement for this submission. On behalf of all authors, disclose any competing interests that could be perceived to bias this work—acknowledging all financial support and any other relevant financial or non-financial competing interests.<br><br>This statement is required for submission and **will appear in the published article** if the submission is accepted. Please make sure it is accurate and that any funding sources listed in your Funding Information later in the submission form are also declared in your Financial Disclosure statement.<br><br>View published research articles from *PLOS ONE* for specific examples.<br><br>**NO authors have competing interests**<br><br>Enter: *The authors have declared that no competing interests exist.*<br><br>**Authors with competing interests**<br><br>Enter competing interest details beginning with this statement:<br><br>*I have read the journal's policy and the authors of this manuscript have the following competing interests: [insert competing interests here]*<br><br>* typeset | |
| **Ethics Statement**<br><br>Enter an ethics statement for this submission. This statement is required if the study involved:<br><br>• Human participants<br>• Human specimens or tissue<br>• Vertebrate animals or cephalopods<br>• Vertebrate embryos or tissues | IRB approval letters (RRC and ERC), informed written consent and assent forms and human subject research checklist have been attached |

Powered by Editorial Manager® and ProduXion Manager® from Aries Systems Corporation

| | |
|---|---|
| • Field research<br><br>Write "N/A" if the submission does not require an ethics statement.<br><br>General guidance is provided below. Consult the submission guidelines for detailed instructions. **Make sure that all information entered here is included in the Methods section of the manuscript.** | |

| | |
|---|---|
| **Format for specific study types**<br><br>**Human Subject Research (involving human participants and/or tissue)**<br>• Give the name of the institutional review board or ethics committee that approved the study<br>• Include the approval number and/or a statement indicating approval of this research<br>• Indicate the form of consent obtained (written/oral) or the reason that consent was not obtained (e.g. the data were analyzed anonymously)<br><br>**Animal Research (involving vertebrate animals, embryos or tissues)**<br>• Provide the name of the Institutional Animal Care and Use Committee (IACUC) or other relevant ethics board that reviewed the study protocol, and indicate whether they approved this research or granted a formal waiver of ethical approval<br>• Include an approval number if one was obtained<br>• If the study involved *non-human primates*, add *additional details* about animal welfare and steps taken to ameliorate suffering<br>• If anesthesia, euthanasia, or any kind of animal sacrifice is part of the study, include briefly which substances and/or methods were applied<br><br>**Field Research**<br><br>Include the following details if this study involves the collection of plant, animal, or other materials from a natural setting:<br>• Field permit number<br>• Name of the institution or relevant body that granted permission | |
| **Data Availability**<br><br>Authors are required to make all data underlying the findings described fully available, without restriction, and from the time of publication. PLOS allows rare exceptions to address legal and ethical concerns. See the PLOS Data Policy and FAQ for detailed information. | No - some restrictions will apply |

Powered by Editorial Manager® and ProduXion Manager® from Aries Systems Corporation

| | |
|---|---|
| A Data Availability Statement describing where the data can be found is required at submission. Your answers to this question constitute the Data Availability Statement and **will be published in the article**, if accepted.<br><br>**Important:** Stating 'data available on request from the author' is not sufficient. If your data are only available upon request, select 'No' for the first question and explain your exceptional situation in the text box.<br><br>Do the authors confirm that all data underlying the findings described in their manuscript are fully available without restriction? | |
| **Describe where the data may be found in full sentences. If you are copying our sample text, replace any instances of XXX with the appropriate details.**<br><br>• If the data are **held or will be held in a public repository**, include URLs, accession numbers or DOIs. If this information will only be available after acceptance, indicate this by ticking the box below. For example: *All XXX files are available from the XXX database (accession number(s) XXX, XXX.*).<br>• If the data are all contained **within the manuscript and/or Supporting Information files**, enter the following: *All relevant data are within the manuscript and its Supporting Information files.*<br>• If neither of these applies but you are able to provide **details of access elsewhere**, with or without limitations, please do so. For example:<br><br>*Data cannot be shared publicly because of [XXX]. Data are available from the XXX Institutional Data Access / Ethics Committee (contact via XXX) for researchers who meet the criteria for access to confidential data.*<br><br>*The data underlying the results presented in the study are available from (include the name of the third party* | N/A |

| | |
|---|---|
| *and contact information or URL).*<br>• This text is appropriate if the data are owned by a third party and authors do not have permission to share the data.<br><br>* typeset | |
| Additional data availability information: | Tick here if the URLs/accession numbers/DOIs will be available only after acceptance of the manuscript for publication so that we can ensure their inclusion before publication.; Tick here if your circumstances are not covered by the questions above and you need the journal's help to make your data available. |

Manuscript Click here to access/download;Manuscript;Manuscript_SHK.docx

# Full title: Parental monitoring challenges and recommendation for managing excessive screen use among adolescents aged 12–14 years in Dhaka, Bangladesh: a qualitative exploration

**Short title: Challenges and recommendations for screen use in adolescents: a qualitative study**

**Authors:**

Shahria Hafiz Kakon[1*], Nahian Sultana[2], Bidhan Krishna Sarker[3], Tanjir Rashid Soron[4], Rashidul Haque[1], Fahmida Tofail[2]

**Affiliations:**

[1]Infectious Diseases Division, International Centre for Diarrheal Disease Research, Bangladesh, Dhaka, Bangladesh

[2]Maternal and Child Nutrition, Nutrition Research Division, International Centre for Diarrheal Disease Research, Bangladesh, Dhaka, Bangladesh

[3]Maternal and Child Health Division, International Centre for Diarrheal Disease Research, Bangladesh, Dhaka, Bangladesh

[4]Telepsychiatry Research and Innovation Network Ltd, Dhaka, Bangladesh

***Corresponding Author:**

Dr. Shahria Hafiz Kakon
Project Research Manager,
Infectious Diseases Division
International Centre for Diarrheal Disease Research, Bangladesh (icddr,b)
68, Shaheed Tajuddin Ahmed Sarani
Mohakhali, Dhaka 1212
Bangladesh
Phone: +8801726428760
E-mail: shahriahafiz@icddrb.org

**Abstract (300 words)**

## Background

Of late, social media and recreational internet use are undeniable. The proper use of devices for children is critical; overindulgence leads to a negative impact on children in many areas of their lives.

## Objectives

This study investigated the challenges that parents face when their children use electronic digital screens excessively. It also consists of recommendations from the participants for the best use of digital devices by adolescent children.

## Methods

The study used a qualitative approach and revealed the obstacles parents encountered to ensure the quality and duration of screen use, as well as some strategies for regulating this element. Data were collected from January 2024 to April 2024. Adolescents aged between 12 and 14 attended six different English and Bangla medium schools in Dhaka; and their parents and teachers were the participants in this study. The data collection methods were focus group discussions, in-depth interviews, and key informant interviews. Data were analyzed using the inductive method and presented in a narrative qualitative manner.

## Results

Results displayed 25 children between classes 6-10, where 56% were males. The core challenges were the recent excessive use of devices for social media and recreational internet besides learning and academics, ensuring safe and proper environments in the neighborhoods for outdoor play, confirming the viewing of age-appropriate content and lastly, a deficit of digital literacy in the parents. Recommendations received were school-based interventions, national-level policies, development of self-regulatory behavior in children, provision of secured infrastructures, enhancing the parent-child bond and offering interesting options to divert the children from screens.

## Conclusions

The study findings provide a better understanding of the challenges of overuse of devices and recommendations among the population. Effective guidelines for managing students' screen use

require the participation from schools, parents, legislators and students themselves. Further research is required to determine the most effective interventions.

**Keywords**

*Screen viewing; device; adolescents; gadgets; social media; challenges; recommendations; focus group discussion; in-depth interviews and key informant interviews*.

## Introduction

The reign of technology is undeniable. In today's age, the blend of the real and digital world has become inevitable. [1] The alloy of virtual media, gaming and recreation has collectively integrated into our daily lives. [2] Despite various confounding factors playing a role in determining the adverse impacts of excessive screen viewing (SV), popular opinion remains that unrestricted screen time has tremendous adverse effects on the health and development of children. [3] Research suggests that socio-economic status (SES) is a highly influential element in the parenting process. Four factors were depicted in this regard. Firstly, higher-SES parents were indecisive about technology use, concerned about its harmful impacts on children its limitations, and also imploring technological skills' potential to mitigate predicted career deficits of children. Secondly, these parents perceived that there were discrepancies in the established recommendations. [4] Thirdly, parenting style where parents intentionally structure their children's lives to help them succeed in the future. which emphasized children's dealings with adults, resulting in children creating situations where parents permitted them to use devices more than they would naturally prefer. Lastly, these processes motivated higher-SES parents to build individualist parenting approaches that typically resulted in children's technology use far exceeding pediatric guidelines. [5] A study controlling the socio-economic factors and ethnicity done on adolescent children about the impacts of excessive screen viewing revealed that more screen time was moderately linked to worse mental health, raised behavioral problems, lowered academic performance and poorer quality of sleep, but increased quality of peer relationships. [6] In addition, a recent paper on-screen viewing and Adolescent Brain Cognitive Development (ABCD) reported that screen time on the higher end was linked to all sorts of mental problems in adolescents; the commonest problems detected being depressive moods, somatic disorders, conduct disorders and attention deficit hyperactivity disorders disorder (ADHD). [7] Also, the particular screen types with the highest associations with depressive symptoms were video chat,

texting, videos and video games. [7] A relevant quantitative study on children about screen time reported that the class year was positively and significantly associated with the average time spent on electronic devices during school days, weekends, and holidays. [8]

In the context of Bangladesh, a relevant paper [9] on adolescents reported that around 79% of the adolescents had increased recreational screen time. Median reported recreational screen time was found to be 4.0 hours/day; with boys having longer times (4.3 hours/day) compared to girls (3.6 hours/day). Multivariable analyses revealed that higher screen time was more common among boys than girls and was positively associated with travelling to school by car, intake of fast food ≥3 times/week, having trouble falling asleep and higher SES. In the recent cutting-edge and modernized era, apart from regular television viewing, children are engaged in a widespread array of devices such as tablets, smartphones, game consoles, laptops and so on. The trend of using more than one device simultaneously is even observed nowadays. [2] Combining online platforms and using Internet media for a number of purposes, such as academic assignments, social communication, and leisure, is a common phenomenon. [10] Due to widespread internet and digital media use lately, previous screen viewing recommendations by the American Association of Pediatrics (AAP) have undergone significant revisions. A "Family Media Use Plan" is now recognized, which helps families to establish an equilibrium between screen time and other activities with no set boundary for screen viewing. [2,10]

Parents, being the primary guardians responsible for providing a healthy environment and shaping the children's future, face a lot of obstacles. With the rapid pace of innovation, particularly in middle-income countries, the restrictions on screen viewing tend to be less effective as they undermine opportunities and developments. [11] Needless to mention, with the hectic schedules of parents and caregivers, it is challenging to keep an eye on the duration of screen time and the type of content that children are exposed to. [11] The debate of concern between protecting children from internet addiction, cybercrime and empowering children for the future with enhanced digital expertise is the sole challenge. [12] A Canadian study conducted focus groups with parents (85% mothers) of children reported that, despite knowing of the negative impacts of screen time, some parents valued screen viewing as a behavior management strategy or by using SV as an 'electronic babysitter' so that parents could complete their household chores. [13] A study in Spain revealed that the principal barrier to physical activity

participation in children, particularly girls, was creating more restrictive and sedentary schedules. The paper suggested enhancing the safety of recreational parks as a solution, emphasizing the gender-sensitive perspective. [14] In a Dutch qualitative analysis, self-interests such as doing tasks undisturbed, creating me-time, regulating children's behavior, avoiding arguments and being able to use devices themselves were the core findings. [15] A recent study conducted by the Indian Academy of Pediatrics suggests that not only SV but other involvements such as outdoor physical activities, sleep, family and peer interaction, education and skill building, which are vital for overall health and development, should all form a part of the daily lives of the children and adolescents. [16] Families should provide an encouraging and fun-filled environment at home, as well as supervise their children's SV to ensure that the content being watched is educational, age-appropriate and non-violent. [16] A healthy Family Media Use Plan, as previously mentioned, as recommended by AAP, can create an optimum balance between SV and other daily activities, encourage age-appropriate critical thought processing digital literacy and lastly, implement elemental rules about the usage of media. [10] A qualitative study done in Kuwait in 2021 on parents regarding children's usage of digital gadgets suggested some as follows: increase familial socialization, encourage children to join health clubs, use reward techniques, arrange regular sports competitions, establish entertainment centers at each region and so on. [17] Another study in the UK in 2021 reported that, school-based interventions could play a vital role alongside parents to prevent excessive use, enhance parent-child interaction and aid in lowering inter-familial conflicts. According to those parents, on thematic analysis it was deducted that, schools were "prevention hubs". [18]

To date, there is a lack of research focusing on the conflicts faced by parents in regulating SV in children. It is imperative to attain a "digital balance" within families to achieve symmetry between the need to remain technologically skilled and the minimization of the chances of internet addiction in children. The aim of this qualitative narrative was to examine the parental challenges for supervising and restricting SV of children as well as gather specific feedback on how to effectively utilize SV within urban Bangladesh context.

## Material and Methods

### Study Design and methods of data collection

This qualitative cross-sectional study employed multiple qualitative methods to achieve its research objectives. Data collection methods included in-depth interviews (IDIs) and focus group discussions (FGDs) with parents and children, as well as key informant interviews (KIIs) with teachers. These methods were employed to comprehensively explore the challenges and gather recommendations for effectively monitoring children's screen time.

**Study settings, participants and the sampling procedure**

Data collection was carried out between January 2024 to April 2024 involved six purposively selected schools—three Bangla medium and three English medium—from North and South Dhaka City Corporations to ensure representation of diverse educational systems, socioeconomic backgrounds and geographical contexts aligning with the study's objective to explore variations in educational experiences.

Participants in this study included three groups: 1) Parents whose children exhibit excessive use of digital devices were interviewed as they are the primary caregivers and often the ones who set rules and boundaries regarding screen time to gain insight into their experiences of challenges in managing their children's screen time. 2) Children with experience of excessive digital device were interviewed to understand their experiences in which they engage with digital devices and how they perceive their screen time limits. 3) Teachers were interviewed to develop potential solutions for managing screen time as they play a significant role in children's development and education.

A diversified group of respondents was purposively selected to ensure a comprehensive understanding of the research objects, incorporating a wide range of perspectives and experiences. Children along with their parents and teachers, were purposively selected from the class register book. For selecting IDI participants, we chose children and parents for separate individual interviews in the school after school hours to ensure they had sufficient time and could participate without distractions. Similarly, we conducted separate Focus Group Discussions (FGDs) with children and parents, considering diverse class, age, and gender to capture a wide range of perspectives and experiences which ensured the discussions reflected the varied backgrounds of participants and encouraged richer insights through different viewpoints.

We selected class teachers for Key Informant Interviews (KIIs) as they are directly involved in the academic environment and can provide valuable insights into challenges and opportunities within the school system, helping inform policy decisions.

There are no known guidelines to determine non-probabilistic sample sizes. Point of saturation or point of redundancy has been done and still is the "intellectual guiding principle" in terms of deciding how many interviews to conduct for a qualitative study with the purposive sampling as its basis. As mentioned, it is only an intellectual guiding principle with little or no on-the-ground guide to sample size estimation that will be required to reach this saturation. [19] To comprehend the challenges and recommendations for monitoring screen use, total 18 interviews were conducted; including 8 IDIs, 4 KIIs and 6 FGDs sessions was conducted. The table 1 below provides detailed information about the sessions and the participants in six schools.

**Table 1: The sample distribution of each school.**

| SL. No. | School Types | In Depth Interviews/ Key Informant Interviews | | | Focus Group Discussions | |
|---|---|---|---|---|---|---|
| | | **Students** | **Teachers** | **Parents** | **Parents** | **Students** |
| 1. | English | 1 | 1 | | 11 | |
| 2. | English | 1 | | 1 | 6 | |
| 3. | English | 1 | 1 | | | 6 |
| 4. | Bangla | 1 | 1 | | | 7 |
| 5. | Bangla | | 1 | 1 | | 7 |
| 6. | Bangla | 1 | | 1 | 7 | |
| | Total participants | 5 | 4 | 3 | 24 | 20 |

## Data collection procedure and quality checking

The data collection process involved a combination of audio recording and note-taking. Based on the study objectives and a review of the existing literature, we developed three separate guidelines: one for students, one for parents and another for teachers. FGDs and IDIs highlighted the challenges and recommendations of supervising screen time of children. After drafting, the

tools were translated from English to Bengali. Data collection was carried out by a field research assistant and a senior research officer, who had more than three and five years of experiences on qualitative data collections methods.

Separate guidelines were prepared and pre-tested to conduct IDI, FGD and KII with 5-6 non-study participants prior to data collection. Semi-structured guidelines were followed to conduct in-depth interviews, limited to approximately 1.5 hours. On the other hand, focus group discussion lasted a maximum of 2 hours, and key informant interviews were open-ended designed, which lasted approximately one hour. The interviews were conducted in the teacher's common room during an off period where the participants could speak freely about the topic. The participants were sitting on chairs arranged in a circle. All interviews were audio recorded. One researcher acted as the facilitator, and the other used the recorder. Privacy was maintained when administering all data procedures. All interviews were carried out in Bangla, the national language of Bangladesh. The facilitator encouraged the discussion and involvement without being placed in the role of expert. She also kept the time distribution equal for each topic to maintain interest. Data collectors were responsible for transcribing, coding as well as summarizing the interviews. A senior research officer and a mental health expert closely monitored the data collection team and provided necessary feedback during data collection to discuss the findings and modification of instruments.

To ensure data reliability, we explored different types informants including parents, children and teachers by conducting interviews in order to gather diverse experiences shared by participants. Data validity was maintained by cross-referencing specific concepts and confirming information with multiple sources. To determining the validity of this qualitative inquiry, we employed three types of data collection tools to achieve methodological, investigator, and data source triangulation. For methodological triangulation, we used IDI, FGD and KII to cross-check information and ensure consistency. Multiple supervisors and investigators were involved to minimize bias and ensure a comprehensive interpretation of the data. Additionally, to validate the findings across various contexts, we collected data from different participants, settings, and time periods.

**Data analysis**

The first stage of qualitative data analysis began by organizing and preparing the field data, including interviews and field notes. The recorded interviews were re-named with symbolic identifiers to maintain confidentiality. Then, the interview data were transcribed verbatim into Bangla and reviewed by the principal investigator during the study. While conducting interviews, relevant notes were collected. All the field notes and interviews (with or without recording) were considered for the data analysis process and transcribed similarly. The transcripts were then carefully examined for data familiarization, leading to the development of a codebook for data coding, which was done using inductive approaches. Coding of interviews KII, IDI and FGD data were coded in matrixes. The analysis team discussed, defined, and applied the codes to the data. Through thematic analysis, common themes were identified to explore the challenges and recommendation as per research objectives. A summary was created for analyzing the themes and sub-themes to identify challenges and recommendation. The findings were then compiled and summarized for all the sites. The use of data and methodological triangulation enabled comprehensive analysis across different research sites and methods. Comparisons between key informants, parents, and students enhanced the validity of the findings.

**Ethical approval**

The study protocol was approved by the Institutional Review Board (IRB) of icddr,b (Protocol No: PR-22002). Written informed consent was taken from each respondent prior to the interview. Interviewers of this study clearly conveyed to the respondents the objectives of the study, reasons for their participation in the study, their role, risks, benefits, anonymity and confidentiality, including the rights not to participate or withdraw. After getting acquainted with all these issues, the respondents decided whether to participate in this study. Confidentiality during and after data collection was ensured, and no identifiable information was used that may expose the identity of the participants. Only the research team members have access to the collected data, which were stored securely. After the interview, each participant received a gift as a token of appreciation for their valuable time.

**Results**

The demographic characteristics of the participants are illustrated in Table 2. All the children were between 12 and 14 years of age; and 56.0% of them were males. The majority of them

(48.0%) were students of class 8, hailed from Bangla medium schools (64.0%) and belonged to nuclear type of families (64.0%). Most of the fathers were doing business (64.0%), whereas mothers were housewives (92.0%). The majority of the children (60.0%) had at least one device and were WiFi users (92.0%). They were found to be using multiple devices, meaning 100.0% used smartphones followed by 76.0% had access to smart TVs.

**Table 2: Participant demographics (n=25).**

| Variables | Attributes | Frequency (f) | Percentage (%) |
|---|---|---|---|
| Gender | Male | 14 | 56.0 |
| | Female | 11 | 44.0 |
| Age (in years) | 12 to 14 | 25 | 100.0 |
| Class | 6th | 2 | 8.0 |
| | 7th | 2 | 8.0 |
| | 8th | 12 | 48.0 |
| | 9th | 8 | 32.0 |
| | 10th | 1 | 4.00 |
| Medium of education | Bangla Medium | 16 | 64.0 |
| | English Medium | 9 | 36.0 |
| Occupation of father | Business | 16 | 64.0 |
| | Service holder | 3 | 12.0 |
| | Others | 3 | 12.0 |
| | Labor | 2 | 8.0 |
| | Skilled worker | 1 | 4.0 |
| Occupation of Mother | Housewife | 23 | 92.0 |
| | Skilled worker | 2 | 8.0 |
| Type of family | Nuclear | 16 | 64.0 |
| | Extended | 9 | 36.0 |
| Device owned by participant | Yes | 15 | 60.0 |
| | No | 10 | 40.0 |
| Type of internet | WiFi or Broadband | 23 | 92.0 |

| | | | |
|---|---|---|---|
| Access | Mobile data | 2 | 8.0 |
| Type of device* used by the participant | Smart phone | 25 | 100.0 |
| | Smart television | 19 | 76.0 |
| | Laptop | 11 | 44.0 |
| | Tablet | 9 | 36.0 |
| | Computer | 8 | 32.0 |

***Indicates multiple responses.**

The holistic concept of screen viewing involving the duration, content, and safety issues was a concern for the parents. Meanwhile, students also had their own explanations and thoughts regarding the outcomes of excessive device use and teachers made their own observations and remarks. The line between digital skill development and internet addiction is razor-thin; it is difficult for parents and caregivers to strike this balance and maintain a harmonious and contented home environment.

An overview of the challenges faced and recommendations provided are discussed, with illustrative verbatim quotes. A lack of marked differences between the interviews and discussion responses was found, so the responses are presented here collectively, with no particular identifiers.

## Challenges faced by parents for monitoring screen use

### 1. Students use digital device for education

Parents reported their limitations to restrict the overuse of gadgets and devices. They revealed their challenges in monitoring the children's screen time because various educational content is available on the digital platform. As a result, parents are bound to provide digital devices like computers and mobile phones, although they doubt whether children were utilizing those devices for education. They assume that children at this age can deviate from studies easily since they are not mature enough to have a grip on themselves. They added that, currently, the teachers do not explain the topics in classrooms; rather, they encourage the students to find the solutions themselves at home, even mentioning that they should search "Google" to solve their queries. Consequently, most of the screen time takes place unsupervised as they cannot take away the devices for the *excuse* of using the device for academic needs. The situation turns more

complicated for working parents as they cannot be available at home for a long time, so they are compelled to permit their children to use "WhatsApp" and "Messenger" as certain school notifications are often distributed through these *social media*. One parent from a Bangla medium school said,

*"The books which are provided as per the new education system, there are no guidelines. So, students need to depend on using mobile phone to take help from the internet. Because teachers nowadays do not explain the topics in the classroom, they say their students to complete them at home. So, when students are given mobile phones for study, it is seen that sometimes they start to play games as an excuse for study."* ***(FGD; Parent; Bangla medium school)***

Students exposed similar findings in the sense that they claimed that they needed to depend on the internet to find information as their class teachers did not explain many topics in class properly as per the instructions in the books. Hence, their class teachers suggest them to take help by searching Google as well as their parents are unable to guide them well on certain topics due to the updated education protocols. They added that they form a group with their classmates and connect online through social networking services as the class teachers give them assignments that they complete together using social networks. Consequently, they are bound to use the internet, which leads to difficulties concentrating on their studies since it distracts them from their studies due to various persuasive elements like notifications and eye-catching interfaces.

*"There are many topics in the books that I do not know how to solve, and even my parents cannot help me with that, so they suggested I search Google. Actually, it often happens that I take permission from my mom to use the mobile phone for study purposes but I do not use it for study purposes. I usually view short videos. Short videos or reels are more fun than study".* ***(FGD; Student; Bangla medium school)***

Echoing same voice, teachers showed concerns about the new curriculum's encouragement of online learning, starting from grade six, which increases students' reliance on mobile devices. Many students lack access to smartphones or the internet, leading to incomplete assignments, while others face distractions when using digital platforms. They added that younger students often lack the maturity to use devices solely for educational purposes. They mentioned that parental supervision is crucial to ensure productive screen time and prevent misuse. The need for

awareness and parental involvement is emphasized to balance the benefits of technology with its risks. One teacher said,

*"If we ask students to create content in groups, say we form a group of ten, usually only two out of the ten ends up doing the work. The remaining eight, instead of contributing, often get busy with unrelated activities on their phones or laptops, under the pretense of preparing content. This puts all the pressure on the two active members. As a result, those two, who are often the group leaders and active students across most subjects, bear the entire burden, leading to increased stress on them."* ***(IDI; Teacher; English medium school)***

**2. Adolescents have become more dependent on devices owing to restrictions in outdoor activities and interaction**

Additionally, parents reported that if children are restricted from using devices, they would try to go out of their homes, which would essentially not be safe and secured (particularly for the girls), with added fears of misguidance by strangers along with encountering eve teasing or, the worst, sexual abuse. Under these circumstances, parents mentioned that they do not allow their children to interact with lesser-known peers for fear of them going astray, which results in fewer friends and acquaintances. As a consequence, children do not discover any opportunities to play outdoor games; rather, they pass their leisure by engaging in devices only. One parent of a Bangla medium school said,

*"Where will our children play in Dhaka city? As we live in apartment, sometimes there is rules or restrictions on going to the rooftop. We are primarily a home-centered family and do not allow our children to mix with others due to concerns about social safety. We do not even know our next-door neighbor due to our lack of time and security. I think there is no other reason than these."* ***(FGD; Parent; Bangla medium school)***

Echoing the same voice, most parents mentioned that very few children have siblings with whom they can play. Simultaneously, both working and non-working parents expressed regret about their inability to spend sufficient time with their children to manage their hectic schedules on a daily basis. In contrast, some parents revealed that children do not feel comfortable communicating with their parents compared to their comfort level with their classmates. One parent of a Bangla medium school added,

*"As they do not have access to playing outdoor games, sometimes we also cannot give enough time as her mom stays occupied and I also stay busy with school activities. What will she do? There is no access to play outside. She might spend her leisure time gossiping with others if we lived in a joint family, but as we live in a nuclear family, there is no scope to do that, either. That's why they are addicted to mobile phones. We also cannot say anything considering all of these issues."* **(IDI; Parents; Bangla medium school)**

Common to their parents, students also highlighted similar issues about lack of interaction with their parents owing to their tedious schedules. One student conveyed,

*"My father sat with me to spend time, but within two hours, he checked his E-mail numerous times. During that time, I also felt a similar urge to use my device as my father checked his inbox messages."* **(FGD; Student; Bangla medium school)**

Additionally, they revealed that they did not have access to play outside due to the scarcity of playgrounds. Many students mentioned that they cannot go out even if there is a playground due to social insecurity concerns and risks such as kidnapping, eve teasing or sexual abuse. In addition, the nuclear family structure was such that there were no cousins of similar age to interact with. Hence, they said there were no alternatives for doing recreational activities other than using devices.

### 3. Unwanted exposure to age-inappropriate contents

Parents stated that unwanted advertisement was yet another obstacle to supervising or restricting the overuse of children's devices as unwanted advertising of sexual content appeared on screen automatically when children viewed "TikTok", "YouTube", "Facebook" and online games. Parents referred to this as a principal challenge as they are unable to suffice and provide ample time to monitor their children. Consequently, they feel perturbed about viewing age-inappropriate videos. Moreover, some parents conveyed that they do not feel comfortable to converse with their children regarding sexual matters. If truth be told, they are not aware how to break the ice about certain physical changes of development in adolescence, even though they are well aware this is the crucial age to accustom them as their children will enter this phase of life. In addition, some parents shared their unpleasant experiences with children viewing inappropriate content, which, according to their perception, occurred due to undesired

advertisements. They included that, after finding out their young ones are exposed to indecent content, negative thoughts frequently appeared in their minds; for instance, children might be curious towards sexual elements in the formative years of life. One parent of a Bangla medium school said,

*"I was astonished after seeing what my son was viewing; when I asked my son about that obscene video, my son replied that he always plays games, but such obscene videos sometimes appeared on his screen as advertisements. But after that incident, I can't stop thinking negatively about him."* ***(FGD; Parents; Bangla medium school)***

Similarly, children revealed that, sometimes, some undesirable advertisements of sexual content appear unintentionally on screens when they engage in social networks. They added that they couldn't skip the advertisements if they wanted to continue watching the content they were viewing. Some of them also notified that watching these unwanted contents further develops their inquisitiveness and provokes them to explore more.

Another point that needs to be emphasized in the context of age-appropriate content is inability to supervise their screen viewing. Parents added that children who use digital technology are *not safe* if they are left unsupervised and have too much screen time. One teacher at an English medium school said,

*"You are leaving your children in the middle of the ocean as you permit them to use Google, YouTube, etc. So, you should view the content before permitting your children to view it. Parents should guide children so that the device used can be beneficial for them."* ***(KII; Teacher; English medium school)***

Similar findings were revealed from the discussion with students where most of the students discussed that children at their age should be kept under supervision because- there are many dark websites of pornography and gambling where children at their age enter just out of inquisitiveness, unaware of the risks and harms of online use. They added that many children use abusive words without knowing the meaning of those words; some fall prey to unsafe relations as well as drug addiction and gambling. For these reasons, most of the students expressed the necessity of supervising children's digital device use. Students emphasized that children would be conscious if they knew that their activities on devices would be supervised or monitored by

their parents, and then they would be discouraged from doing any wrong activities using those devices.

Similar findings revealed from the discussion of teachers whereas they reported that the lack of internet restrictions allows students to access various sites and learn things beyond their age, leading to unregulated usage. This is reflected in the classroom, where students use inappropriate, vulgar, or adult language—words that were unknown to previous generations at such a young age. Teachers often learn about these words for the first time from their students. One teacher said,

Due to digital device usage, children are accessing inappropriate content and becoming addicted to it, with younger students watching adult material and mimicking adult behaviors. This leads to a lack of communication, with interactions moving to platforms like Facebook. When breakups occur, they struggle to cope, often resulting in depression, as they are too young to handle such situations. (***(KII; Teacher; English medium school)***

### 4. Lack of parental digital literacy

Parents reported that the lack of digital literacy was a major challenge in monitoring their children. They mentioned that their children are more accustomed to using gadgets and devices than their parents are, as most of them are unaware of the proper operation of the devices. They added that children are well versed on internet safety strategies; hence, many parents do not set any age limit app by creating an E-mail account to restrict inappropriate content since their young ones can often figure out how to do away with such *technical* tricks. Moreover, some parents mentioned that they are not even habituated to these technical strategies, which they consider to be another huge challenge. One parent of an English medium school said,

*"He is expert on device use than me. He is an expert in IT. He can hack our software to open it, that's why I left to use age limit apps."* ***(FGD; Parent; English medium school)***

An interesting finding was revealed from the discussion of students, who mentioned that students of their age make *fake accounts* in order to access certain video games and social media apps. One student in this context reported,

*"There are many children who lie by increasing their age using fake profile accounts."* ***(FGD; Parent; English medium school)***

**Recommendations from study participants to reduce excessive screen time:**

1. **Initiatives to be taken by schools**
    - Participants, including parents and students, emphasized the significance of school efforts to implement steps to supervise and lower device usage. Most parents suggested replacing online activities in the teaching curriculum with innovative offline activities.
    - They also proposed that students should complete their device-related tasks at school under the supervision of teachers rather than bringing them home. In this context, parents advocated for school authorities to provide the required devices for such purposes.
    - Both parents and students drew attention to the need to raise awareness from the school's end regarding the harmful effects of excessive device use on children. They urged the implementation of collaborative programs involving parents, students, and teachers on school premises to pursue the objective of reducing screen use this objective in a holistic manner.
    - Students and parents also discussed the value of increasing extracurricular activities at school. Some parents have suggested allocating specific times regularly for children to be involved in physical activities and innovative games. They believe that this approach will help divert their attention from using devices.
    - Teachers and parents have accentuated recruiting a mentor, specifically a psychologist, within the school. This mentor would particularly address concerns regarding excessive device use among children and offer guidelines to overcome addiction along with other psychological support.
    - Meanwhile, teachers highlighted using all devices for academic purposes under the supervision of teachers at school premises because using digital devices in education is indispensable in today's world. Subjects like ICT and computer studies are impossible to comprehend without the aid of digital devices.

2. **Alternative options instead of screen viewing**
    - All participants, including parents, teachers, and students, recommended offering other choices instead of screen viewing. Preferable activities like playing outdoor games, indulging in creative arts and crafts, engaging in an exciting book, or participating in sports or hobbies they would enjoy, thus providing a range of options that would enrapture their attention beyond the screen.
    - Students suggested reducing screen time by removing the barriers and enhancing accessibility for outdoor playing and activities in the surrounding neighborhood.
3. **Parents should spend quality time with their children**
    - All participants stressed the significance of parents spending quality time with their children to reduce screen time. They should prioritize quality time together, offering a practical approach to managing screen time and nurturing healthy relationships within the family.
    - Students said that parents can actively engage in meaningful interactions, such as conversations and combined activities like morning walks, picnics or outings, interactive games, cooking something together and so on, all of which would strengthen the parent-child bonding, culminating in decreased screen time.
4. **To develop self-regulation in children**
    - Most students and parents refer to the importance of building self-regulation to reduce the excessive use of digital devices. They believe that learning to refrain from prolonged use of screens is more effective than enforcing strict rules and regulations by parents.
5. **Ensuring social security**
    - Parents recommended engaging children in outdoor activities to reduce screen time. They emphasized the importance of creating safe and supervised environments. They believed that promoting outdoor play reduces screen time and offers the benefits of physical exercise, social interaction and the development of essential life skills and resilience.

6. **Policies to be implemented on the national level**

- Parents have proposed numerous ideas for the government to implement in order to reduce children's screen viewing and create safer digital platforms. They suggested developing digital restriction mechanisms with the help of the ICT (Information & Communication Technology) Department of Bangladesh to avoid age-inappropriate content such as drug abuse or indecent videos, which can prove to be harmful to them.

- Based on their views, content filtering or web filtering means using software or hardware to block access to specific websites or online content categories based on predefined criteria. They added that by such filtering, children can be prevented from accessing detrimental or undesirable materials while browsing the internet.

- Some parents have suggested the development of specialized software or applications that offer children-friendly content with specific age categories, ensuring that they are exposed to age-appropriate materials while using digital devices. Additionally, parents could utilize these apps to monitor their children's screen time activities directly, ensuring their children indulged in content that aligned with their respective cultural values and traditions. This approach combines content filtering with parental monitoring features to create a safer and more integrated digital environment for children.

- Additionally, parents highlighted that the government should utilize various media platforms, such as TV channels and social media, where children spend most of their time, to raise awareness about the deleterious effects of excessive digital device use. They said that advertising campaigns can effectively reach children and parents, alerting them about the potential risks associated with prolonged screen time.

- Some parents stress on including a chapter in every year's ICT textbook, specifically focusing on the negative impacts of excessive screen

viewing. They argue that while the subject teaches students how to use digital devices and familiarizes them with various features, it often neglects to address the potential negative consequences of unrestrained usage.

- Teachers also emphasize governmental interventions to formalize rules and regulations for children's digital device usage. For instance, a suggestion has been made that the screen time duration should be mentioned in their textbooks according to age, such as children in the second grade being limited to one hour of screen time per day as per recommendations of regulatory bodies.

**Discussion**

The data on this paper demonstrates the challenges faced by parents to regulate screen viewing as well as suggestions with the means to reduce use of digital media and gadgets. To begin with, devices nowadays are an indispensable source of learning and acquiring knowledge. Our data revealed that, parents believed SV can be a useful educational tool, showing consent to previous research conducted by Solomon Moore et al [2]. The study highlighted four main points in the challenges section, one of which being “positive aspects of SV”, where parents found restricting SV could turn into an obstacle for them in terms of their technological know-hows. Another domain was “not all screens are equal” where the parents actively encouraged educational SV mentioning their children enjoyed watching documentaries and researching topics on the internet. The greater challenge would appear when children used these devices mentioning they were indulged in academics however operating it for non-academic purposes such as gaming and recreation. There is immense scope for future research to implore this concept.

A common challenge reported by parents was their inability to provide outdoor access to children. These issues were raised throughout the current study as safety was a major barrier for outdoor activities. A qualitative case study [20] done on Spanish immigrant children highlighted similar findings. Factors such as importance of physical activities, gender disparities, academic load, scarcity of social acquaintances, familial socio-economic limitations, absence of proper surroundings, time constraints and feelings of insecurity were the key-points which were exposed in the paper. Owing to rising crimes and chances for children being kidnapped or sexually harassed, the challenge was inevitable. Another qualitative research [21] on barriers and

facilitators of physical activity among schoolchildren identified children preferring sedentary activities such as video games, influence of older siblings, lack of family mediation and deficit of sufficient infrastructure as the main findings. It was elicited that, lack of spaces such as courtyards and terraces (particularly those who lived in smaller homes), parks or gardens were a major hurdle for active play.

According to our findings, unwanted or inappropriate content was a significant barrier that parents wanted to avoid.

Previous quantitative research [22] suggests that, parent-child relationship, socio-economic conditions, parents being unaware of children's SV and children owning devices by themselves were major contributive elements for viewing violent or pornographic content. Such exposure to adult content at the wrong time implicate multiple risks and can lead to harmful outcomes. There could be increased curiosity about these elements resulting in lack of attention to academic contents and deviating to watching more adult videos which could have a deleterious impact on the future of the children. In this case, the familial understanding is supposed to play a vital role. Relevant qualitative paper [23] explored the concerns of parents to exposure to harmful content. The elements they thought were important in case of girl child were impacts of social media, cyberbullying, grooming and early sexualization; whereas addictive behaviour, gaming and pornography viewing for boys.

Our findings demonstrate that a lack of digital literacy was a significant factor.

The home technology environment is imperative in this context. Various studies [24-25] report that, children of this generation are exposed to a wide range of toys, multimedia, devices and gadgets from a very early age which enable and enhance them to efficiently operate them. This, in contrast, is not the scenario for the parents of this generation. Their attitudes, preferences and concerns are different owing to lack of digital knowledge in them. Digital literacy is an essential skill of late. A paper by Romero [24] mentioned, there are 4 subsets of skills for parents namely basic digital parenting skills, communication skills, creativity and life-long learning skills. Digital knowledge imparts an understanding of online safety. [26] In addition, they need to adapt to the changing times which is engulfed in the digital environment. Lack of digital literacy leads to familial disputes, impacting the peace and harmony of the home; on the contrary, learning these skills can protect the children and strengthen family bonds. Only when parents are capable

to use such devices with ease, they can take charge of children's screen viewing duration and most importantly, screen content.

Furthermore, this study focused on some recommendations which the caregivers suggested in order to reduce SV in the young ones. They mentioned that schools should take some core initiatives. A cluster-randomized controlled trial [27] to evaluate the effectiveness of school-based intervention on screen time in Bangladesh was done between two groups reported that, there were promising changes in children when physical activity was encouraged and screen time was reduced.

Points such as implementation of national guidelines, offering alternatives to screen viewing, increased family time, development of self-regulation in children and ensuring safer environment were also discussed extensively. Relevant qualitative exploration [17] enumerated some solutions for the government to imply such as monitor inappropriate programs for the young ones and block them; use social media to explain the detrimental impacts of overuse of devices; establish more sports clubs; ensure computer classes at schools which would include lessons regarding optimum SV. Another paper [28] mentioned that, strong bonding between children and parents impacts the SV of children in a beneficial manner. Furthermore, similar research [29] recommended that, parents need to enhance their knowledge about social media and associated risks to protect their children. In addition, a systematic review [30] revealed that, weaker parent-child bonding and unhealthy familial environment was linked to gaming addiction in children. Also, single-parent families were detected to have greater amount of internet abuse in the young ones.

It is essential to take into account the deleterious impacts of excessive SV on children. The implications of this paper are based on the challenges encountered by the caregivers and their thoughtful proposals to mitigate this condition The balance between the real and virtual world is difficult to maintain, but it is mandatory. While it is necessary to maintain the guidelines of children's SV flexible in order to go with the flow of the rapidly-changing technological ambience, certain guidelines need to be implemented to make sure caregivers understand how much SV is excessive. It can be encouraged to develop specific policies and programs on multiple levels to eliminate the difficulties faced with regards to monitoring SV and promote alternative activities and options to foster a healthier life for the children.

## Limitations

This study has a number of limitations. There were only three KIIs with teachers, and two of the respondents were men and one was a woman. This could have influenced the results in terms of viewpoints on the topics covered in the interviews. If we had more time, we could have expanded the sample sizes and added focus groups with mothers and fathers separately, as well as with boys and girls, to triangulate the findings from the KIIs and IDIs. The urban setting of Dhaka, Bangladesh, where we conducted this study, may have affected the findings and made them different from those of other rural areas of the country. Lastly, such qualitative research in a particular region with a small sample size is not representative of the entire population.

## Conclusion

Urban digitalization has its own set of drawbacks. The hurdles caregivers face in regulating screen use of children highlight the contribution of devices for academic purposes and their digital illiteracy. The inability to ensure safety for outdoor activities is a major drawback. In addition, ensuring age-appropriate content for children turns out to be a matter of concern.

It is essential to implement policy guidelines that emphasize the active involvement of schools, complemented by national-level strategies. Furthermore, a warm home environment and safe and suitable infrastructure are compulsory.

## Acknowledgment

The author would like to acknowledge their affiliation, with the icddr, b and its core donors who provide unrestricted support to icddr,b for its operations and research, including the Governments of Bangladesh and Canada. We gratefully acknowledge our core donors for their support and commitment to icddr,b's research efforts. The authors are also grateful to the school authorities for their unconditional support.

## Data availability

The data sets generated or analyzed during this study are available from the corresponding author upon reasonable request.

**Authors' contributions**

SHK and NS contributed to conceptualizing, analyzing, writing, revising, and finalizing the manuscript with the support of BKS, TRS, RH, and FT. All the authors have read, revised, and approved the final version of the manuscript.

**Conflict of interests**

None declared.

**Funding statement**

The current study was financially supported by the Bangladesh government's Mujib100 Research Grant for Women award, in collaboration with icddr, b, grant no. 1945.

## References

1. Ashton JJ, Beattie RM. Screen time in children and adolescents: is there evidence to guide parents and policy? The Lancet Child & Adolescent Health. 2019 May 1;3(5):292-4.
2. Solomon-Moore E, Matthews J, Reid T, Toumpakari Z, Sebire SJ, Thompson JL, et al. Examining the challenges posed to parents by the contemporary screen environments of children: a qualitative investigation. BMC pediatrics. 2018 Dec; 18:1-2.
3. Wilkinson C, Low F, Gluckman P. Screen time: The effects on children's emotional, social, and cognitive development.
4. Lareau A. Unequal childhoods: Class, race, and family life. Inequality in the 21st Century 2018 May 15 (pp. 444-451). Routledge.
5. Mollborn S, Limburg A, Pace J, Fomby P. Family socioeconomic status and children's screen time. Journal of Marriage and Family. 2022 Aug;84(4):1129-51.
6. Paulich KN, Ross JM, Lessem JM, Hewitt JK. Screen time and early adolescent mental health, academic, and social outcomes in 9-and 10-year-old children: Utilizing the Adolescent Brain Cognitive Development℠(ABCD) Study. PloS one. 2021 Sep 8;16(9):e0256591.
7. Nagata JM, Al-Shoaibi AA, Leong AW, Zamora G, Testa A, Ganson KT, Baker FC. Screen time and mental health: a prospective analysis of the Adolescent Brain Cognitive Development (ABCD) Study. BMC Public Health. 2024 Oct 7;24(1):2686.

8. Tsang SM, Cheing GL, Lam AK, Siu AM, Pang PC, Yip KC, et al. Excessive use of electronic devices among children and adolescents is associated with musculoskeletal symptoms, visual symptoms, psychosocial health, and quality of life: a cross-sectional study. Frontiers in Public Health. 2023 Jun 29;11:1178769.
9. Khan A, Burton NW. Screen-based behaviors of adolescents in Bangladesh. Journal of Physical Activity and Health. 2016 Nov 1;13(11):1156-63.
10. Reid Chassiakos YL, Radesky J, Christakis D, Moreno MA, Cross C, Hill D, Ameenuddin N, Hutchinson J, Levine A, Boyd R, Mendelson R. Children and adolescents and digital media. Pediatrics. 2016 Nov 1;138(5).
11. Livingstone S, Byrne J. Parenting in the digital age: The challenges of parental responsibility in comparative perspective.
12. Blum-Ross A, Livingstone S. "Sharenting," parent blogging, and the boundaries of the digital self. InSelf-(re) presentation now 2020 May 18 (pp. 70-85). Routledge.
13. Carson V, Clark M, Berry T, Holt NL, Latimer-Cheung AE. A qualitative examination of the perceptions of parents on the Canadian Sedentary Behaviour Guidelines for the early years. International Journal of Behavioral Nutrition and Physical Activity. 2014 Dec;11:1-8.
14. Martínez-Andrés M, Bartolomé-Gutiérrez R, Rodríguez-Martín B, Pardo-Guijarro MJ, Garrido-Miguel M, Martínez-Vizcaíno V. Barriers and facilitators to leisure physical activity in children: a qualitative approach using the socio-ecological model. International journal of environmental research and public health. 2020 May;17(9):3033.
15. Geurts SM, Koning IM, Vossen H, Van den Eijnden RJ. A qualitative study on children's digital media use and parents' self-interest. Journal of Child and Family Studies. 2022 Jul 1:1-2.
16. Gupta P, Shah D, Bedi N, Galagali P, Dalwai S, Agrawal S, John JJ, Mahajan V, Meena P, Mittal HG, Narmada S. Indian Academy of Pediatrics Guidelines on screen time and digital wellness in infants, children and adolescents. Indian pediatrics. 2022 Mar;59(3):235-44.
17. Buabbas A, Hasan H, Shehab AA. Parents' attitudes toward school students' overuse of smartphones and its detrimental health impacts: Qualitative study. JMIR Pediatrics and Parenting. 2021 May 20;4(2):e24196.

18. Throuvala MA, Griffiths MD, Rennoldson M, Kuss DJ. Policy recommendations for preventing problematic internet use in schools: a qualitative study of parental perspectives. International journal of environmental research and public health. 2021 Apr 24;18(9):4522.
19. Guest G, Namey E, Chen M. A simple method to assess and report thematic saturation in qualitative research. PloS one. 2020 May 5;15(5):e0232076.
20. Marconnot R, Marín-Rojas AL, Delfa-de-la-Morena JM, Pérez-Corrales J, Gueita-Rodríguez J, Fernández-de-Las-Peñas C, et al. Recognition of barriers to physical activity promotion in immigrant children in Spain: a qualitative case study. International journal of environmental research and public health. 2019 Feb;16(3):431.
21. Alcántara-Porcuna V, Sánchez-López M, Martínez-Vizcaíno V, Martínez-Andrés M, Ruiz-Hermosa A, Rodríguez-Martín B. Parents’ perceptions on barriers and facilitators of physical activity among schoolchildren: A qualitative study. International Journal of Environmental Research and Public Health. 2021 Mar 17;18(6):3086.
22. Liu S, Chang FC, Chiu CH, Li F, Chen PH, Chen CY, et al. Parent–Child Discrepancies in Reports of Exposure to Violence/Pornography on Mobile Devices and the Impact on Children's Psychosocial Adjustment. Cyberpsychology, Behavior, and Social Networking. 2021 Jun 1;24(6):390-8.
23. Marsh S, Chu JT, Calder AJ. ‘I tried to take my phone off my daughter, and i got hit in the face’: a qualitative study of parents’ challenges with adolescents’ screen use and a toolbox of their tips. BMC public health. 2024 Jan 18;24(1):217.
24. Romero M. Digital literacy for parents of the 21st century children. Elearning Papers. 2014 May;38:32-40.
25. Terras MM, Ramsay J. Family digital literacy practices and children’s mobile phone use. Frontiers in psychology. 2016 Dec 23;7:1957.
26. Sulistyo PB, Umarella FH, Mahmudah SM, Iza N. Digital Literacy Competence of Parents in Supervising Their Children Using Digital Media. International Journal of Social Science and Human Research. 2022 Feb:636-42.
27. Ahmed KR, Kolbe-Alexander T, Khan A. Effectiveness of a school-based intervention on physical activity and screen time among adolescents. Journal of science and medicine in sport. 2022 Mar 1;25(3):242-8.

28. Nwankwo F, Shin HD, Al-Habaibeh A, Massoud H. Evaluation of children's screen viewing time and parental role in household context. Global pediatric health. 2019 Sep;6:2333794X19878062.
29. Moawad GE, Ebrahem GG. The Relationship between Use of Technology and Parent-Adolescents Social Relationship. Journal of Education and Practice. 2016;7(14):168-78.4
30. Schneider LA, King DL, Delfabbro PH. Family factors in adolescent problematic Internet gaming: A systematic review. Journal of behavioral addictions. 2017 Sep;6(3):321-33.
