# Supplementary material for: Parental monitoring challenges and recommendation for managing excessive screen use among students aged 12–14 years in Dhaka, Bangladesh: A qualitative exploration
Source: PLoS One. 2026 Oct 5;21(10):e0359518. doi: 10.1371/journal.pone.0359518 (PMC13637862; doi:10.1371/journal.pone.0359518)
Supplement: Supplementary file 2 [file pone.0359518.s004.pdf]

PLOS ONE

Parental monitoring challenges and recommendation for managing excessive screen use among adolescents aged 12–14 years in Dhaka, Bangladesh: a qualitative exploration

--Manuscript Draft--

**Manuscript Number:**	PONE-D-25-02782R1
**Article Type:**	Research Article
**Full Title:**	Parental monitoring challenges and recommendation for managing excessive screen use among adolescents aged 12–14 years in Dhaka, Bangladesh: a qualitative exploration
**Short Title:**	Challenges and recommendations for screen use in adolescents: a qualitative study
**Corresponding Author:**	Shahria Hafiz Kakon, MBBS,MPH,PhD fellow<br>ICDDR B: International Centre for Diarrhoeal Disease Research Bangladesh<br>Dhaka, BANGLADESH
**Keywords:**	Digital Device; Students; Screen viewing; Use of social media; Bangladesh
**Abstract:**	Background: The pervasive use of social media and recreational internet platforms among students has become increasingly evident. The proper use of devices for students is critical; overindulgence leads to a negative impact on students in many areas of their lives. This study aimed to explore the challenges parents face when their students engage in excessive use of electronic digital screens. Additionally, it sought to capture participants' recommendations for the best use of digital devices among students.<br>Methods: An exploratory qualitative study design was employed to understand parental experience in regulating screen use and strategies adopted to manage its duration and quality. Data was collected between January and April 2024. Students aged between 12 and 14 who attending class six to ten English and Bangla medium schools in Dhaka and their parents and teachers were the participants in this study. Data was collected through focus group discussions and different type of in-depth interviews. A thematic analysis was performed using inductive approach.<br>Results: We included 25 students who attending between classes six and ten, of whom 56% were male. Key challenges identified by parents included excessive use of devices for social media and recreational purposes beyond academic purposes, ensuring safe and conductive outdoor environments, monitoring age-appropriate content, and a lack of digital literacy among parents. Participants' recommendations comprehended school-based awareness interventions, national level guideline regarding the use of digital devices, development of self-regulatory behavior in students, provision of secured recreational infrastructures, create scope outdoor recreational activities among students, enhancing the parent-child bond and offering interesting options to divert the students from screens.<br>Conclusions: The study findings provide a better understanding of the challenges of overuse of devices and recommendations among the population. Effective guidelines for managing students' screen use require participation from schools, parents, legislators and students themselves. Further research is required to determine the most effective interventions.
**Order of Authors:**	Shahria Hafiz Kakon, MBBS,MPH,PhD fellow
	Nahian Sultana
	Bidhan Krishna Sarker
	Tanjir Rashid Soron
	Rashidul Hoque
	Fahmida Tofail
**Response to Reviewers:**	NA
**Additional Information:**	

Powered by Editorial Manager® and ProduXion Manager® from Aries Systems Corporation

Question	Response
**Financial Disclosure**<br><br>Enter a financial disclosure statement that describes the sources of funding for the work included in this submission. Review the submission guidelines for detailed requirements. View published research articles from *PLOS ONE* for specific examples.<br><br>This statement is required for submission and **will appear in the published article** if the submission is accepted. Please make sure it is accurate.<br><br>**Funded studies**<br>Enter a statement with the following details:<br>• Initials of the authors who received each award<br>• Grant numbers awarded to each author<br>• The full name of each funder<br>• URL of each funder website<br>• Did the sponsors or funders play any role in the study design, data collection and analysis, decision to publish, or preparation of the manuscript?<br><br>Did you receive funding for this work?	Yes
Please add funding details.<br>as follow-up to "**Financial Disclosure**<br><br>Enter a financial disclosure statement that describes the sources of funding for the work included in this submission. Review the submission guidelines for detailed requirements. View published research articles from *PLOS ONE* for specific examples.<br><br>This statement is required for submission and **will appear in the published article** if the submission is accepted. Please make sure it is accurate.	Bangladesh government awarded project named Mujib 100 research Grant for women. I have received this award on 2022. Funded was $ 43,250 for 2 years project. The funders had no role in study design, data collection and analysis, decision to publish, or preparation of the manuscript.

**Funded studies**<br>Enter a statement with the following details:<br>• Initials of the authors who received each award<br>• Grant numbers awarded to each author<br>• The full name of each funder<br>• URL of each funder website<br>• Did the sponsors or funders play any role in the study design, data collection and analysis, decision to publish, or preparation of the manuscript?<br><br>Did you receive funding for this work?"	
Please select the country of your main research funder (please select carefully as in some cases this is used in fee calculation).<br>as follow-up to "**Financial Disclosure**<br><br>Enter a financial disclosure statement that describes the sources of funding for the work included in this submission. Review the submission guidelines for detailed requirements. View published research articles from *PLOS ONE* for specific examples.<br><br>This statement is required for submission and **will appear in the published article** if the submission is accepted. Please make sure it is accurate.<br><br>**Funded studies**<br>Enter a statement with the following details:<br>• Initials of the authors who received each award<br>• Grant numbers awarded to each author<br>• The full name of each funder<br>• URL of each funder website<br>• Did the sponsors or funders play any role in the study design, data collection and analysis, decision to publish, or preparation of the manuscript?<br><br>Did you receive funding for this work?"	BANGLADESH - BD
**Competing Interests**	The authors have declared that no competing interests exist.

Use the instructions below to enter a competing interest statement for this submission. On behalf of all authors, disclose any competing interests that could be perceived to bias this work—acknowledging all financial support and any other relevant financial or non-financial competing interests.<br><br>This statement is required for submission and **will appear in the published article** if the submission is accepted. Please make sure it is accurate and that any funding sources listed in your Funding Information later in the submission form are also declared in your Financial Disclosure statement.<br><br>View published research articles from *PLOS ONE* for specific examples.<br><br>**NO authors have competing interests**<br><br>Enter: *The authors have declared that no competing interests exist.*<br><br>**Authors with competing interests**<br><br>Enter competing interest details beginning with this statement:<br><br>*I have read the journal's policy and the authors of this manuscript have the following competing interests: [insert competing interests here]*<br><br>* typeset	
**Ethics Statement**<br><br>Enter an ethics statement for this submission. This statement is required if the study involved:<br><br>• Human participants<br>• Human specimens or tissue<br>• Vertebrate animals or cephalopods<br>• Vertebrate embryos or tissues	The study protocol was approved by the Institutional Review Board (IRB) of icddr,b (Protocol No: PR-22002). Informed written consent was taken from each respondent (parents, teacher) and students were taken consent from their parents as well as assent was taken as their age was above 11 years prior to the interview. All the respondents were informed in Bengali about their rights related to their voluntary participation in the study and withdrew from the interview at any time during the interview. Interviewers of this study clearly conveyed to the respondents the objectives of the study, reasons for their participation in the study, their role, risks, benefits, anonymity and confidentiality, including the rights not to participate or withdraw. After getting acquainted with all these issues, the respondents decided whether to

• Field research<br><br>Write "N/A" if the submission does not require an ethics statement.<br><br>General guidance is provided below. Consult the submission guidelines for detailed instructions. **Make sure that all information entered here is included in the Methods section of the manuscript.**	participate in this study. Confidentiality during and after data collection was ensured, and no identifiable information was used that may expose the identity of the participants. Only the research team members have access to the collected data, which were stored securely. After the interview, each participant received a gift (equivalent to BDT370 [USD3] as a token of appreciation for their valuable time.

Powered by Editorial Manager® and ProduXion Manager® from Aries Systems Corporation

**Format for specific study types**<br><br>**Human Subject Research (involving human participants and/or tissue)**<br>• Give the name of the institutional review board or ethics committee that approved the study<br>• Include the approval number and/or a statement indicating approval of this research<br>• Indicate the form of consent obtained (written/oral) or the reason that consent was not obtained (e.g. the data were analyzed anonymously)<br><br>**Animal Research (involving vertebrate animals, embryos or tissues)**<br>• Provide the name of the Institutional Animal Care and Use Committee (IACUC) or other relevant ethics board that reviewed the study protocol, and indicate whether they approved this research or granted a formal waiver of ethical approval<br>• Include an approval number if one was obtained<br>• If the study involved *non-human primates*, add *additional details* about animal welfare and steps taken to ameliorate suffering<br>• If anesthesia, euthanasia, or any kind of animal sacrifice is part of the study, include briefly which substances and/or methods were applied<br><br>**Field Research**<br><br>Include the following details if this study involves the collection of plant, animal, or other materials from a natural setting:<br>• Field permit number<br>• Name of the institution or relevant body that granted permission	
**Data Availability**<br><br>Authors are required to make all data underlying the findings described fully available, without restriction, and from the time of publication. PLOS allows rare exceptions to address legal and ethical concerns. See the PLOS Data Policy and FAQ for detailed information.	No - some restrictions will apply

A Data Availability Statement describing where the data can be found is required at submission. Your answers to this question constitute the Data Availability Statement and **will be published in the article**, if accepted.<br><br>**Important:** Stating 'data available on request from the author' is not sufficient. If your data are only available upon request, select 'No' for the first question and explain your exceptional situation in the text box.<br><br>Do the authors confirm that all data underlying the findings described in their manuscript are fully available without restriction?	
**Describe where the data may be found in full sentences. If you are copying our sample text, replace any instances of XXX with the appropriate details.**<br><br>• If the data are **held or will be held in a public repository**, include URLs, accession numbers or DOIs. If this information will only be available after acceptance, indicate this by ticking the box below. For example: *All XXX files are available from the XXX database (accession number(s) XXX, XXX.*).<br>• If the data are all contained **within the manuscript and/or Supporting Information files**, enter the following: *All relevant data are within the manuscript and its Supporting Information files.*<br>• If neither of these applies but you are able to provide **details of access elsewhere**, with or without limitations, please do so. For example:<br><br>*Data cannot be shared publicly because of [XXX]. Data are available from the XXX Institutional Data Access / Ethics Committee (contact via XXX) for researchers who meet the criteria for access to confidential data.*<br><br>*The data underlying the results presented in the study are available from (include the name of the third party*	The data from this study are not publicly available due to ethical restrictions and icddr,b's data sharing policy. The study involved in-depth interviews and focus group discussions. Although the data were anonymized and participants provided consent after being informed about confidentiality safeguards, there remains a risk of identification because of the level of detail in the responses. The data are held by icddr,b. For any requests during peer review, please contact Sabina Shahnaz (sabina.shahnaz@icddrb.org). Data may also be made available upon reasonable request for research purposes through Shiblee Sayeed (shiblee_s@icddrb.org), Head of Research Administration at icddr,b, in accordance with the institution's data policy.

Powered by Editorial Manager® and ProduXion Manager® from Aries Systems Corporation

and contact information or URL).<br>• This text is appropriate if the data are owned by a third party and authors do not have permission to share the data.<br><br>* typeset	
Additional data availability information:	

Manuscript Click here to access/download;Manuscript;Revised Manuscript.docx

Title: Parental monitoring challenges and recommendation for managing excessive screen use among students aged 12–14 years in Dhaka, Bangladesh: a qualitative exploration

Authors:

Shahria Hafiz Kakon[1*], Nahian Sultana[2], Bidhan Krishna Sarker[3], Tanjir Rashid Soron[4], Rashidul Haque[1], Fahmida Tofail[2]

Affiliations:

[1]Infectious Diseases Division, International Centre for Diarrheal Disease Research, Bangladesh, Dhaka, Bangladesh

[2]Maternal and Child Nutrition, Nutrition Research Division, International Centre for Diarrheal Disease Research, Bangladesh, Dhaka, Bangladesh

[3]Maternal and Child Health Division, International Centre for Diarrheal Disease Research, Bangladesh, Dhaka, Bangladesh

[4]Telepsychiatry Research and Innovation Network Ltd, Dhaka, Bangladesh

***Corresponding Author:**

Dr. Shahria Hafiz Kakon

Project Research Manager,

Infectious Diseases Division,

International Centre for Diarrheal Disease Research, Bangladesh (icddr,b)

68, Shaheed Tajuddin Ahmed Sarani, Mohakhali, Dhaka 1212, Bangladesh

Phone: +8801726428760; E-mail: shahriahafiz@icddrb.org

Abstract

Background: The pervasive use of social media and recreational internet platforms among students has become increasingly evident. The proper use of devices for students is critical; overindulgence leads to a negative impact on students in many areas of their lives. This study aimed to explore the challenges parents face when their students engage in excessive use of electronic digital screens. Additionally, it sought to capture participants' recommendations for the best use of digital devices among students.

Methods: An exploratory qualitative study design was employed to understand parental experience in regulating screen use and strategies adopted to manage its duration and quality. Data was collected between January and April 2024. Students aged between 12 and 14 who attending class six to ten English and Bangla medium schools in Dhaka and their parents and teachers were the participants in this study. Data was collected through focus group discussions and different type of in-depth interviews. A thematic analysis was performed using inductive approach.

Results: We included 25 students who attending between classes six and ten, of whom 56% were male. Key challenges identified by parents included excessive use of devices for social media and recreational purposes beyond academic purposes, ensuring safe and conductive outdoor environments, monitoring age-appropriate content, and a lack of digital literacy among parents. Participants' recommendations comprehended school-based awareness interventions, national level guideline regarding the use of digital devices, development of self-regulatory behavior in students, provision of secured recreational infrastructures, create scope outdoor recreational activities among students, enhancing the parent-child bond and offering interesting options to divert the students from screens.

Conclusions: The study findings provide a better understanding of the challenges of overuse of devices and recommendations among the population. Effective guidelines for managing students' screen use require participation from schools, parents, legislators and students themselves. Further research is required to determine the most effective interventions.

Keywords: Digital Device; Students; Screen viewing; Use of social media; Bangladesh

Introduction

The presence of technology in everyday life is undeniable. The boundaries between digital and reals worlds have blurred, and children are increasingly immersed in virtual media, gaming, and online communication [1] [2]. While digital tools can provide educational and recreational benefits, excessive screen viewing (SV) has been associated with adverse impacts on children's health and development [3]. Evidence reported the role of socio-economic status (SES) in shaping parenting practices related to technological use. is a highly influential element in the parenting process. Four factors were depicted in this regard. For example, higher-SES parents often struggle to reconcile concerns about harmful impacts with the perceived need for children to develop digital skills [4]. Parenting styles also influence children's technology habits, with some parents permitting more device use as part of an individualized parenting approach, even when this exceeds pediatric guidelines [5].

Evidence links prolonged SV with negative outcomes such as poorer mental health, sleep disturbance, reduced academic performance, and behavioral problems, although in some cases, screen use has also been associated with improved peer relationships [6]. Another recent paper on-screen viewing and Adolescent Brain Cognitive Development (ABCD) further indicated

associations between high screen time and depressive symptoms, conduct disorders, and attention-deficit/hyperactivity disorder [7]. In addition, the type of screen activity matters: texting, video, chats, video streaming, and gaming show stronger association with mental health challenges compared to other forms of use [7]. A relevant quantitative study on children about screen time reported that the class year was positively and significantly associated with the average time spent on electronic devices during school days, weekends, and holidays [8].

In Bangladesh, a recent study reported that recreational SV is rising rapidly among adolescents. The study found that around 79% of the adolescents had increased recreational screen time [9]. Median reported recreational screen time was found to be 4.0 hours/day; with boys having longer times (4.3 hours/day) compared to girls (3.6 hours/day). Further the study reported that high SV was associated with unhealthy behaviors, such as frequent fast-food consumption, difficulty falling asleep, and higher SES [9]. In the recent cutting-edge and modernized era, apart from regular television viewing, children are engaged in a widespread array of devices such as tablets, smartphones, game consoles, laptops and so on [9]. The trend of using more than one device simultaneously is even observed nowadays [2]. Combining online platforms and using Internet media for a number of purposes, such as academic assignments, social communication, and leisure, is a common phenomenon [10]. Due to widespread internet and digital media use ~~lately~~, previous screen viewing recommendations by the American Association of Pediatrics (AAP) have undergone significant revisions. A "Family Media Use Plan" is now recognized, which helps families to establish an equilibrium between screen time and other activities with no set boundary for screen viewing [2,10]. However, no such tailored guidelines currently exist in the Bangladesh context.

Parents, being the primary guardians responsible for providing a healthy environment and shaping the children's future, face a lot of obstacles. With the rapid pace of innovation, particularly in middle-income countries, the restrictions on screen viewing tend to be less effective as they undermine opportunities and developments [11]. Needless to mention, with the hectic schedules of parents and caregivers, it is challenging to keep an eye on the duration of screen time and the type of content that children are exposed to [11]. The debate of concern between protecting children from internet addiction, cybercrime and empowering children for the future with enhanced digital expertise is the sole challenge [12]. Studies across diverse contexts highlights the concern. For example, Canadian parents (85% mothers) of children reported that, despite knowing of the negative impacts of screen time, some parents valued screen viewing as a behavior management strategy or by using SV as an 'electronic babysitter' to manage their household demands [13]. In Spain, restrictive schedules and safety concerns limited children's opportunities for the physical activity, especially for girls [14]. In a Dutch study, self-interests such as doing tasks undisturbed, creating me-time, regulating children's behavior, avoiding arguments and being able to use devices themselves were the core findings [15]. A recent study conducted by the Indian Academy of Pediatrics suggests that not only SV but other involvements such as outdoor physical activities, sleep, family and peer interaction, education and skill building, which are vital for overall health and development, should all form a part of the daily lives of the children and adolescents [16]. whereas, parents in Kuwait family-based strategies, including encourage children to join health clubs, use reward techniques, arrange regular sports competitions, establish entertainment centers at each region to manage SV [17] and parents in UK identified school-based interventions alongside the parent's role to prevent excessive use [18].

Despite the growing body of evidence, there remains a lack of research exploring how parents in low-and middle-income countries (LMICs) navigate these challenges. In Bangladesh, where digital access is rapidly expanding but parental guidance and protecting structure are limited, understanding these dynamics is particularly important. This study therefore aimed to explore the challenges parents face in supervising and restricting children's SV in urban Dhaka, Bangladesh, and to gather their feedback on strategies that may help achieve a healthier balance between digital engagement and overall child well-being.

Material and Methods

Study design

This study employed an exploratory qualitative design with standard qualitative tools being used to gather information on our research interest. We conducted qualitative interview from the same schools where previously quantitative study was performed. We collected data from parents, school going students and teachers which provided a holistic understanding of the phenomena and a detailed contextual explanation for quantitative result. Methods were applied with purposive sampling of different types of informants. A combination of multiple tools, including in-depth interviews (IDIs) with parents, students and teachers and focus group discussions (FGDs) with students and their parents, was utilized to understand experiences, perceptions, and recommendations regarding the use of screen time. The use of multiple methods facilitated the collection of both individual and collective perspective. Such methodological triangulation is recognized for enhancing the trustworthiness of qualitative findings.

Study settings

We conducted this study in Dhaka city. We purposively selected three Bangla-medium and three English-medium schools from Dhaka North and Dhaka South city corporations. Dhaka has been selected as the center of the country with dominant presence of high socioeconomic community considering over exposure to digital screens. Both Bangla and English-medium schools have been chosen in order to ensure the equal distribution of the Bangla and English medium schools for the representation of diverse educational systems, socioeconomic status, and geographical context. During the school selection, we also considered with the kind permission of the school authorities, students aged between 12 to 14 years, who were in class 6 to class 10, and who used more than two hours of screen time each day as the qualifying criteria for sample inclusion. Smartphones, tablets, laptops, PCs, gaming consoles, televisions, and portable gaming devices were among the acceptable gadgets. whereas, students who might have affected the results due to pre-existing conditions, such as physical or psychological impairment, were excluded from the study.

Study participants and sampling

We have included three types of participants: 1) parents whose students exhibit excessive use of digital devices were interviewed as they are primary caregivers and who set rules and boundaries regarding screen time. 2) students with experience of excessive digital device used were interviewed to understand their experiences of engagement pattern and perceptions of screen time limits. 3) teachers were interviewed to develop potential strategies for managing screen time as they play a significant role in student development and education.

A heterogeneous sampling technique was utilized for IDIs, while a homogeneous technique was applied for FGDs [19]. IDIs with students and parents were conducted separately after school hours

to ensure sufficient time and minimize distractions. FGDs were also organized separately with students and parents, considering diverse class, age, sex to capture diverse perspectives. A total of 12 IDIs and six FGDs were conducted across six schools, which was sufficient to reach thematic saturation. Table 1 provides detailed information about the study participants below.

[Table 1 to be inserted here]

Data collection method and quality checking

Data collection was carried out between January 2024, and April 2024 using a combination of audio recording and note taking. Three separate semi-structured guidelines were developed based on the study focus and literature review [6,7,13,17,20–28]: one each for students, parents and teacher. The guidelines focused on the challenges and recommendations for monitoring screen time for students. The tools were translated from English to Bengali prior to use. All tools were pre-tested with 6 non-study participants to ensure clarity and appropriateness.

Each IDI lasted approximately 40-60 minutes and FGD up to maximum 90 minutes. The interviews were conducted in teachers' common room at the end of the class, allowing the participants to speak freely about the topic. For FGDs, the participants were seated in a circle, and all interviews were audio recorded. Nobody allows the interview room during sessions and maintained confidentiality. All the interviews were conducted in Bangla, the national language of Bangladesh and privacy was maintained throughout the interview. Interviews were conducted by a Senior Research Officer who has more than five years of qualitative research experience. On the other hand, interview note has been taken by a Field Research Assistant who has more than three years of experience in qualitative research. Both of whom received rigorous training prior to data

collection. There is also a Mental Health Expert who has observed the interviews team and provided feedback where necessary. Interviewers were responsible for transcribing, coding, and summarizing the interviews. Further, to ensure data credibility and dependability, we engaged multiple types of informants including parents, students and teacher to gather diverse experience shared by the participants.

Operational definitions

Screen Viewing (SV): The time students spend using digital devices for any purpose, including educational, recreational, or social activities.

Digital Device: Any electronic device that allows access to digital content, such as smartphones, tablets, laptops, Television, computers, and game consoles.

Digital Platform: Online services or applications accessed through digital devices, including websites, social media, educational tools, and entertainment platforms.

Digital Literacy: The ability to effectively and safely use digital devices and platforms, including understanding content, managing screen time, and navigating online resources.

Outdoor Activity: Any physical or recreational activity performed outside the home, including sports, games, creative play, and other forms of physical exercise.

Peer Interaction: Social engagement and communication with other students, either in-person or virtually, fostering social skills and relationships.

Inappropriate Content: Digital material that is harmful, age-inappropriate, or culturally unacceptable, such as violent, sexual, or drug-related content.

Social Safety: The provision of a secure and supervised environment for students to engage in activities, reducing exposure to harm and promoting physical, emotional, and social well-being.

Data analysis

Data analysis began with organizing and preparing the field data, including interviews and field notes. To maintain confidentiality recorded interviews were assigned symbolic identifiers. All the interviews were transcribed verbatim in Bangla and reviewed by the principal investigator. Relevant notes were collected during interviews and field observation were also incorporated into the analysis.

The transcripts were read thoroughly for data familiarization, which guided the development of a codebook using the inductive approach. IDI and FGD data were coded in the appropriate codes. The analysis team collaboratively defines and re-defines codes. Thematic analysis then conducted to identify common themes and sub-themes related to research objectives [29]. The findings were then compiled and summarized for all the sites.

To enhance rigor, data triangulation was applied at multiple levels: methodological (IDI and FGD), researchers (multiple researchers), and data sources (different type of participants). These strategies enhance trustworthiness, minimize potential bias, and ensure that the findings reflected the richness and diversity of different perspectives [30].

Ethical approval

The study protocol was approved by the Institutional Review Board (IRB) of icddr,b (Protocol No: PR-22002). Informed written consent was taken from each respondent (parents, teacher) and students were taken consent from their parents as well as assent was taken as their age was above 11 years prior to the interview. All the respondents were informed in Bengali about their rights related to their voluntary participation in the study and withdrew from the interview at any time

during the interview. Interviewers of this study clearly conveyed to the respondents the objectives of the study, reasons for their participation in the study, their role, risks, benefits, anonymity and confidentiality, including the rights not to participate or withdraw. After getting acquainted with all these issues, the respondents decided whether to participate in this study. Confidentiality during and after data collection was ensured, and no identifiable information was used that may expose the identity of the participants. Only the research team members have access to the collected data, which were stored securely. After the interview, each participant received a gift (equivalent to BDT370 [USD3] as a token of appreciation for their valuable time.

Results

Background Characteristics of participants

The demographic characteristics of the participants are illustrated in Table 2. All the students were between 12 and 14 years of age; and 56% of them were males. The majority of them (48%) were students of class 8, hailed from Bangla-medium schools (64%) and belonged to nuclear type families (64%). Most of the fathers of students are involved in business (64%), whereas mothers were housewives (92%). The majority of the students (60%) had at least one device and a substantial percentage (92%) was Wi-Fi users. They were found to be using multiple devices, meaning 100% used smartphones followed by 76% had access to smart TVs.

[Table 2 to be inserted here]

The holistic concept of screen viewing involving the duration, content, and safety issues was a concern for the parents. Meanwhile, students also had their own explanations and thoughts

regarding the outcomes of excessive device use and teachers made their own observations and remarks. The line between digital skill development and internet addiction is razor-thin; it is difficult for parents and caregivers to strike this balance and maintain a harmonious and contented home environment.

An overview of the challenges faced, and recommendations provided are discussed, with illustrative verbatim quotes. A lack of marked differences between the interviews and discussion responses was found, so the responses are presented here collectively, with no particular identifiers.

Challenges faced by parents for monitoring screen use

Students' use digital device for education

Parents highlighted the lack of sufficient guidance in the newly introduced education system, which increased student's reliance on mobile phones and the internet for academic purposes. They reported that teachers often did not provide adequate explanations in class, pushing students to depend on online resources. While parents provided mobile phones to support their students' learning, they observed that students frequently misused the devices for non-academic purposes, such as playing games, under the pretext of studying.

> *"The books which are provided as per the new education system, there are no guidelines. So, students need to depend on using mobile phone to take help from the internet. Because teachers nowadays do not explain the topics in the classroom, they tell their students to complete them at home. So, when students are given mobile phones for study, it is seen that sometimes they start to play games as an excuse for study."* ***(FGD; Parent; Bangla medium school)***

Student's reliance on the internet for learning support

Students reported that they were often directed by teachers to search Google or other online sources for solving classroom tasks. Many students mentioned that parents could not provide guidance on the updated curriculum, which further increased their dependence on the internet. They frequently formed online groups with classmates to complete assignments, using social networking platforms for communication. However, they admitted that such academic use of devices often led to distractions, particularly from short videos, reels, and other entertainment content.

> *"There are many topics in the books that I do not know how to solve, and even my parents cannot help me with that, so they suggested I search Google. Actually, it often happens that I take permission from my mom to use the mobile phone for study purposes, but I do not use it for study purposes. I usually watch short videos. Short videos or reels are more fun than studying".* ***(FGD; Student; Bangla medium school)***

> *"We often have group work, and for that we need to discuss things. Now if we don't have a phone, how will we discuss? That's why the phone is necessary."* ***(FGD; Student; Bangla medium school)***

Parents' perspectives on digital learning and supervision

All parents emphasized that as a result of excessive device use, their student's academic performance is directly impacted, resulting in lower grades.

> *"My son's roll number is dropping from 20 to 50. He only spends one hour on coaching and private tutoring. The rest of the day, he's glued to his mobile." (FGD; Parents; Bangla medium school)*

Teachers' perspectives on digital learning and supervision

Teachers expressed concerns about the increasing dependence on digital platforms from grade six onwards. While acknowledging the benefits of technology for learning, they noted that many students lacked the maturity to use devices responsibly. Teachers also observed that group work often created inequalities, where a small number of students bore the entire burden, while others misused devices for unrelated activities. They emphasized the importance of parental supervision and awareness to ensure productive screen use.

> *"If we ask students to create content in groups, say we form a group of ten, usually only two out of the ten ends up doing the work. The remaining eight, instead of contributing, often get busy with unrelated activities on their phones or laptops, under the pretense of preparing content. This puts all the pressure on the two active members. As a result, those two, who are often the group leaders and active students across most subjects, bear the entire burden, leading to increased stress on them."* ***(IDI; Teacher; English medium school)***

Safety concerns and limited peer interaction

Parents expressed concerns about the safety of outdoor spaces, particularly for girls, which limited opportunities for students to interact with peers. Fear of misguidance by strangers, eve teasing, or sexual abuse led parents to restrict their students from going outside or interacting with lesser-known peers. Consequently, students had few friends and spent most of their leisure time on digital devices.

> *"Where will our children play in Dhaka city? As we live in apartment, sometimes there are rules or restrictions on going to the rooftop. We are primarily a home-*

centered family and do not allow our children to mix with others due to concerns about social safety. We do not even know our next-door neighbor due to our lack of time and security. I think there is no other reason than these." ***(FGD; Parent; Bangla medium school)***

Limited family time and communication

Most parents reported that very few children had siblings to play with and that both working and non-working parents struggled to spend sufficient time with their children due to busy schedules. Some parents noted that children felt more comfortable communicating with classmates than with family members.

> *"...sometimes we also cannot give enough time as her mom stays occupied and I also stay busy with school activities. What will she do? There is no access to play outside. She might spend her leisure time gossiping with others if we lived in a joint family, but as we live in a nuclear family, there is no scope to do that, either. That's why they are addicted to mobile phones. We also cannot say anything considering all of these issues."* ***(IDI; Parents; Bangla medium school)***

Students also highlighted similar issues about lack of interaction with their parents owing to their tedious schedules. One student conveyed,

> *"My father sat with me to spend time, but within two hours, he checked his E-mail numerous times. During that time, I also felt a similar urge to use my device as my father checked his inbox messages."* ***(FGD; Student; Bangla medium school)***

Scarcity of outdoor play and recreational opportunities

Both parents and students reported restricted access to outdoor play due to the lack of playgrounds and concerns over social insecurity, including kidnapping, eve teasing, or sexual abuse. In nuclear families, children lacked cousins or age-matched peers to interact with, leaving digital devices as the main alternative for leisure and recreation.

> *"In my home, it's just a nuclear family. Some families might have home support, but kids don't really listen to them, and they can't provide real company either. So, the child ends up spending that time mostly with electronic devices. Since both of us work, that's what keeps the child occupied." (**FGD; parents; English medium school**)*

> "*Even when cousins are together, they're all on their devices. Each cousin has one in hand. Instead of chatting, they just sit with their devices. We, on the other hand, can talk for hours after hours. But our kids can't. After talking for a little while, they'll say they need a break, which means they want to check their phone and then again, they go back to the phone. Managing them is much tougher.*" ***(FGD; parents; English medium school)***

Unwanted exposure to age-inappropriate contents

Parents reported that students were frequently exposed to unwanted sexual content through advertisements on platforms such as TikTok, YouTube, Facebook, and online games. Many parents found it challenging to monitor student's device use due to time constraints and discomfort discussing sexual topics, despite recognizing the importance of guidance during childhood. Exposure to such content caused worry and anxiety, as parents feared it might trigger curiosity or inappropriate behaviors.

> *"I was astonished after seeing what my son was viewing; when I asked my son about that obscene video, my son replied that he always plays games, but such obscene videos sometimes appeared on his screen as advertisements. But after that incident, I can't stop thinking negatively about him."* ***(FGD; Parents; Bangla medium school)***

Socioeconomic status and screen time

We identified a positive association between higher SES and higher possibility of the use of screen time among students.

> *"Families with higher income often provide multiple devices to their children, which makes it harder to control screen time and can affect their sleep and concentration."* ***(IDI; Parents; Bangla medium school)***

> *"Some of my friends don't have personal devices at home, so they play outside more, and I think they stay healthier than those who spend all day on phones or tablets."* *(**FGD; Student; Bangla medium school**)*

These quotes illustrate how SES shapes access to technology and, consequently, children's screen habits and associated health outcomes.

Student's perspective on unwanted content

Students confirmed that sexual or inappropriate content sometimes appeared unintentionally while using digital platforms, and they were unable to skip certain advertisements. They reported that exposure increased curiosity and encouraged further exploration, underscoring the risks of unsupervised screen time.

"Sometimes a bad video pops up, and once you watch it, you feel like watching more. Later it causes physical problems… the body feels weak. I mean, in the middle of a video, like for 30 seconds, these kinds of clips appear. Actually, they're just 3-second videos that come up automatically. I was playing a game when those ads popped up, and my father saw it. After that, he didn't give me the phone for two or three days." **(FGD; Parents; English medium school)**

"After watching it, I keep thinking, 'Did I really watch that?' It keeps coming to my mind all the time, and it feels like mental pressure. So, I can't focus on anything else. And once I've seen it, I want to watch it again; if I don't, it doesn't feel good." **(FGD; Parents; English medium school)**

Teachers' perspective on supervision

Teachers emphasized the importance of parental supervision to ensure safe and productive use of digital devices. They noted that unsupervised access allowed students to engage with age-inappropriate content, which could negatively affect behavior, communication, and emotional development.

"You are leaving your children in the middle of the ocean as you permit them to use Google, YouTube, etc. So, you should view the content before permitting your children to view it. Parents should guide children so that the device used can be beneficial for them." **(IDI; Teacher; English medium school)**

"Due to digital device usage, children are accessing inappropriate content and becoming addicted to it, with younger students watching adult material and mimicking adult behaviors. This leads to a lack of communication, with

*interactions moving to platforms like Facebook. When breakups occur, they struggle to cope, often resulting in depression, as they are too young to handle such situations". (***(IDI; Teacher; English medium school)***

Students' views on supervision

Students stressed that supervision could prevent access to unsafe websites, pornography, gambling, and the use of abusive language. They indicated that awareness of parental monitoring would discourage engagement in risky or inappropriate online behaviors.

*"Children would be conscious if they knew that their activities on devices would be supervised or monitored by their parents, and then they would be discouraged from doing any wrong activities using those devices." (***FGD; Student; English medium school***)*

Lack of parental digital literacy

Parents acknowledged that limited digital literacy constrained their ability to monitor children effectively. Children were often more technologically skilled and could bypass restrictions or age limits, leaving parents feeling unable to enforce boundaries.

"He is expert on device use than me. He is an expert in IT. He can hack our software to open it, that's why I left to use age limit apps." ***(FGD; Parent; English medium school)***

Students also reported creating fake accounts to access games or social media apps restricted by age settings.

"There are many children who lie by increasing their age using fake profile accounts." ***(FGD; Parent; English medium school)***

Recommendations from study participants to reduce excessive screen time

Individual and Family Level

- Parents should spend quality time with their children: Parents should engage in meaningful interactions such as conversations, morning walks, picnics, interactive games, or cooking together to strengthen parent–child bonding and naturally reduce reliance on digital devices.
- Developing self-regulation in children: Encourage children to manage their own screen habits and refrain from prolonged use, emphasizing self-discipline over strict parental rules.

> *"The time that is being spent on the mobile means they are not getting enough rest. Of course, that has an effect. That's why I think counseling is necessary. Because through counseling, whether it's about mobile use or something else, the issue will come up. They will hear things like, 'Don't do this,' or, 'It's better if you avoid that,' and then the topic of studies will also come up. One way or another, some advice will be given."* ***(FGD_Parents_ACADEMIA)***

- Alternative options instead of screen viewing: Promote hobbies like outdoor games, creative arts and crafts, reading, sports, and other enjoyable activities to capture children's attention beyond digital devices.

> *"I have the same opinion as the others. One more recommendation I would make is that, since study time is spent on mobile devices, children then look for suggestions on how to excel in exams in a short amount of time. Because of this, they become more concerned with just performing well in exams rather than actually learning. That's a major harm. Another point is that devices will be used anyway, so along with the children, the parents also need to be made aware. If we overreact, then whatever is forbidden is exactly what they will want*

to do. In that case, if parents behave logically, it will help, because sometimes their overreaction can have a negative impact. That's why it would be good to have a counseling session for parents as well, on how to guide their children. At the school level, too, parents could be made more aware of how to behave with their children." ***(FGD_Parents_ACADEMIA)***

Institutional and Community Level

- Initiatives to be taken by schools:
    - Replace online activities in the curriculum with offline approaches.
    - Ensure device-related tasks are completed under teacher supervision, with schools providing necessary devices.
    - Raise awareness about the harmful effects of excessive screen use through collaborative programs involving parents, teachers, and students.
    - Increase extracurricular opportunities, such as physical activities and creative games, to divert children's attention from devices.
    - Appoint a school-based mentor, preferably a psychologist, to address device overuse concerns and provide psychological support.
    - Limit digital tools like ICT and computer studies to supervised use within school premises.
- Alternative options instead of screen viewing (institutional support): Reduce barriers and improve access to outdoor spaces and neighborhood activities to encourage participation in non-screen pursuits.

"Every area needs to have a playground. There should be guards around the field and cameras installed along the area's borders." ***(FGD_Baridhara_students)***

- Ensuring social security: Create safe and supervised environments for outdoor activities, encouraging physical exercise, social interaction, and development of life skills while reducing screen time.

  "Schools could arrange *programs to make parents aware of how harmful mobile phones can be. Such initiatives could also be taken through different NGOs. These programs could highlight the mental health problems associated with mobile phone use and explain them sincerely to parents. Various awareness sessions could also be conducted."* ***(B2_FGD_Students_ DAM)***

Policies to be implemented on the national level

- Parents proposed a range of government-led initiatives to reduce children's screen viewing and promote safer digital platforms. They suggested developing digital restriction mechanisms in collaboration with the ICT Department of Bangladesh to prevent exposure to age-inappropriate content, using content or web filtering tools to block harmful websites and online categories. Additionally, parents recommended creating specialized software or applications offering age-appropriate content while enabling parental monitoring, combining safety with culturally relevant oversight.
- Government-driven awareness campaigns were also emphasized, utilizing widely accessed platforms such as television and social media to inform parents and children about the risks of excessive screen time. Integrating discussions of digital risks into formal education was another key recommendation, including dedicated chapters in ICT textbooks addressing the

negative impacts of device overuse. Teachers further suggested incorporating age-specific screen time guidelines in textbooks, formalizing limits such as restricting second-grade students to one hour of screen use per day in line with regulatory recommendations.

> *"This has actually become a national issue now since almost all children are facing this problem. If the government includes these issues in the curriculum, it carries more importance than just us parents saying it. This could be done through NGOs or schools as well. The media we have could also be used to raise awareness. For example, we were taught many things through the Meena cartoon. In the same way, cartoons could be made to show the negative sides of mobile use, so that children themselves would be attracted to watch and learn."*
>
> ***(FGD_Parents_ACADEMIA)***

Overall, these recommendations reflect a collective call from parents and teachers for national - level policies that combine regulation, education, awareness, and technology-based solutions to address the growing challenges of children's screen use.

Discussion

This study explored the challenges parents face in monitoring their school going children's screen use in the context of the evolving educational and social environment in urban Bangladesh. Parents and students highlighted that the new education system encourages independent learning and reliance on digital platforms. Many parents reported that teachers provide insufficient guidance in classrooms, pushing students to seek help online. While digital devices facilitate learning, parents observed frequent misuse, such as playing games under the guise of studying. Students' dependence on online resources and group assignments via social media further exacerbated the

challenge, as distractions from short videos, reels, and other entertainment content were commonly reported. Studies found that the barriers and facilitators of physical activity among schoolchildren identified children preferring sedentary activities such as video games, influence of older siblings, lack of family mediation and deficit of sufficient infrastructure as the main findings [31]. Teachers noted that younger students often lack the maturity to use devices responsibly, emphasizing the importance of parental supervision to ensure productive and safe digital learning.

The study identified several challenges, one of which was the “positive aspects of SV”, where parents found restricting SV could turn into an obstacle for them in terms of their technological know-hows. Another domain, “not all screens are equal” where the parents actively encouraged educational SV mentioning their children enjoyed watching documentaries and researching topics on the internet. These findings suggest considerable scope for future research to further explore the nuanced impacts of different types of screen use.

Parents’ concerns about social safety, particularly for girls, restricted children’s outdoor activities and peer interactions. A qualitative case study done on Spanish immigrant children highlighted similar findings [32]. Fear of eve teasing, sexual abuse, or misguidance led parents to limit their children’s opportunities for outdoor activities and physical play. In nuclear families, the scarcity of siblings or cousins further reduced opportunities for interaction, leaving digital devices as the main avenue for leisure. Limited family time and communication gaps were also evident, as parents’ busy schedules hindered meaningful engagement with their children. Children reported feeling more comfortable interacting with peers than with parents, a phenomenon that may contribute to increased screen dependency. These findings align with previous research indicating that urban children often face constrained outdoor play due to safety concerns and that parental availability plays a critical role in managing screen time [33].

Parents and teachers expressed concern about children’s exposure to inappropriate content, often appearing through advertisements on popular digital platforms. Previous quantitative research suggests that parent-child relationship, socio-economic conditions, parents being unaware of children’s SV and children owning devices by themselves were major contributive elements for viewing violent or pornographic content [34]. Many parents reported feeling unprepared to discuss sexual development, which compounded the challenges of supervision. Students confirmed that exposure to such content often increased curiosity and encouraged further exploration, reinforcing the risks of unsupervised digital use. Relevant qualitative paper explored the concerns of parents to exposure to harmful content [21]. Teachers highlighted that unsupervised access could lead to behavioral and emotional consequences, including imitation of adult behaviors, reduced communication skills, and vulnerability to online risks. Students emphasized that awareness of parental monitoring could discourage engagement in unsafe or inappropriate activities.

A significant barrier to effective supervision was parents limited digital literacy. The home technology environment is imperative in this context. Various studies report that children of this generation are exposed to a wide range of toys, multimedia, devices and gadgets from a very early age which enable and enhance them to efficiently operate them [35,36]. In contrast, many parents reported being less technologically skilled than their children, who could bypass restrictions or manipulate age verification systems. Students also reported creating fake accounts to access games or social media apps restricted by age. These results reflect global evidence showing that parents’ digital skills are critical for establishing boundaries and guiding safe online behaviors [37].

Furthermore, this study focused on some recommendations which the caregivers suggested in order to reduce SV in the young ones. They mentioned that schools should take some core initiatives. A cluster-randomized controlled trial to evaluate the effectiveness of school-based

intervention on screen time in Bangladesh was done between two groups reported that, there were promising changes in children when physical activity was encouraged and screen time was reduced [28].

Points such as implementation of national guidelines, offering alternatives to screen viewing, increased family time, development of self-regulation in children and ensuring safer environment were also discussed extensively. Relevant qualitative exploration enumerated some solutions for the government to imply such as monitor inappropriate programs for the young ones and block them; use social media to explain the detrimental impacts of overuse of devices; establish more sports clubs; ensure computer classes at schools which would include lessons regarding optimum SV [17]. Another paper mentioned that, strong bonding between children and parents impacts the SV of children in a beneficial manner [20]. Furthermore, similar research recommended that parents need to enhance their knowledge about social media and associated risks to protect their children [38]. In addition, a systematic review revealed that, weaker parent-child bonding and unhealthy familial environment was linked to gaming addiction in children [39]. Also, single-parent families were detected to have greater internet abuse in young ones.

It is essential to consider the deleterious impacts of excessive SV on children. The implications of this paper are based on the challenges encountered by the caregivers and their thoughtful proposals to mitigate this condition The balance between the real and virtual world is difficult to maintain, but it is mandatory. While it is necessary to maintain the guidelines of children's SV flexible in order to go with the flow of the rapidly changing technological ambience, certain guidelines need to be implemented to make sure caregivers understand how much SV is excessive. It can be encouraged to develop specific policies and programs on multiple levels to eliminate the

difficulties faced with regards to monitoring SV and promote alternative activities and options to foster a healthier life for children.

Conclusion

Urban digitalization has its own set of drawbacks. The hurdles caregivers face in regulating screen use of children highlight the contribution of devices for academic purposes and their digital illiteracy. The inability to ensure safety for outdoor activities is a major drawback. In addition, ensuring age-appropriate content for children turns out to be a matter of concern. It is essential to implement policy guidelines that emphasize the active involvement of schools, complemented by national-level strategies. Furthermore, a warm home environment and safe and suitable infrastructure are compulsory.

Declarations

Acknowledgment

The author would like to acknowledge their affiliation, with the icddr, b and its core donors who provide unrestricted support to icddr,b for its operations and research, including the Governments of Bangladesh and Canada. We gratefully acknowledge our core donors for their support and commitment to icddr,b's research efforts. The authors are also grateful to the school authorities for their unconditional support.

Authors' contributions

SHK and NS contributed to conceptualizing, analyzing, writing, revising, and finalizing the manuscript with the support of BKS, TRS, RH, and FT. All the authors have read, revised, and approved the final version of the manuscript.

Data Availability

The data from this study are not publicly available due to ethical restrictions and icddr,b's data sharing policy. The study involved in-depth interviews and focus group discussions. Although the data were anonymized and participants provided consent after being informed about confidentiality safeguards, there remains a risk of identification because of the level of detail in the responses. The data are held by icddr,b. For any requests during peer review, please contact Sabina Shahnaz (sabina.shahnaz@icddrb.org). Data may also be made available upon reasonable request for research purposes through Shiblee Sayeed (shiblee_s@icddrb.org), Head of Research Administration at icddr,b, in accordance with the institution's data policy.

Conflict of interests

None declared.

Reference

1. Ashton JJ, Beattie RM. Screen time in children and adolescents: is there evidence to guide parents and policy? Lancet Child Adolesc Heal. 2019;3: 292–294. doi:10.1016/S2352-4642(19)30062-8
2. Solomon-Moore E, Matthews J, Reid T, Toumpakari Z, Sebire SJ, Thompson JL, et al. Examining the challenges posed to parents by the contemporary screen environments of children: a qualitative investigation. BMC Pediatr. 2018;18: 129. doi:10.1186/s12887-018-1106-y
3. Wilkinson C, Low F, Gluckman P. Screen time: The effects on children's emotional, social, and cognitive development. Univ Auckl Cent Inf Futur. 2021; 1–11.
4. Lareau A. Unequal Childhoods: Class, Race, and Family Life. 2018 pp. 444–451.

Available: https://www.taylorfrancis.com/chapters/edit/10.4324/9780429499821-75/unequal-childhoods-class-race-family-life-annette-lareau

5. Mollborn S, Limburg A, Pace J, Fomby P. Family Socioeconomic Status and Children's Screen Time. J Marriage Fam. 2022;84: 1129–1151. doi:10.1111/jomf.12834

6. Paulich KN, Ross JM, Lessem JM, Hewitt JK. Screen time and early adolescent mental health, academic, and social outcomes in 9- and 10- year old children: Utilizing the Adolescent Brain Cognitive Development SM (ABCD) Study. PLoS One. 2021;16: e0256591. doi:10.1371/journal.pone.0256591

7. Nagata JM, Al-Shoaibi AAA, Leong AW, Zamora G, Testa A, Ganson KT, et al. Screen time and mental health: a prospective analysis of the Adolescent Brain Cognitive Development (ABCD) Study. BMC Public Health. 2024;24: 2686. doi:10.1186/s12889-024-20102-x

8. Tsang SMH, Cheing GLY, Lam AKC, Siu AMH, Pang PCK, Yip K-C, et al. Excessive use of electronic devices among children and adolescents is associated with musculoskeletal symptoms, visual symptoms, psychosocial health, and quality of life: a cross-sectional study. Front public Heal. 2023;11: 1178769. doi:10.3389/fpubh.2023.1178769

9. Khan A, Burton NW. Screen-Based Behaviors of Adolescents in Bangladesh. J Phys Act Health. 2016;13: 1156–1163. doi:10.1123/jpah.2015-0514

10. Reid Chassiakos YL, Radesky J, Christakis D, Moreno MA, Cross C. Children and Adolescents and Digital Media. Pediatrics. 2016;138. doi:10.1542/peds.2016-2593

11. Livingstone S BJ. Parenting in the digital age: The challenges of parental responsibility in comparative perspective. diva-portal.org. 2018. Available:

https://scholar.google.com/scholar?q=Parenting+in+the+digital+age:+The+challenges+of+parental+responsibility+in+comparative+perspective&hl=en&as_sdt=0&as_vis=1&oi=scholart

12. Blum-Ross A, Livingstone S. "Sharenting," parent blogging, and the boundaries of the digital self. Pop Commun. 2017;15: 110–125. doi:10.1080/15405702.2016.1223300

13. Carson V, Clark M, Berry T, Holt NL, Latimer-Cheung AE. A qualitative examination of the perceptions of parents on the Canadian Sedentary Behaviour Guidelines for the early years. Int J Behav Nutr Phys Act. 2014;11: 65. doi:10.1186/1479-5868-11-65

14. Martínez-Andrés M, Bartolomé-Gutiérrez R, Rodríguez-Martín B, Pardo-Guijarro MJ, Garrido-Miguel M, Martínez-Vizcaíno V. Barriers and Facilitators to Leisure Physical Activity in Children: A Qualitative Approach Using the Socio-Ecological Model. Int J Environ Res Public Health. 2020;17. doi:10.3390/ijerph17093033

15. Geurts SM, Koning IM, Vossen H, Van den Eijnden RJJM. A Qualitative Study on Children's Digital Media Use and Parents' Self-interest. J Child Fam Stud. 2022;31: 2015–2026. doi:10.1007/s10826-021-02074-3

16. Gupta P, Shah D, Bedi N, Galagali P, Dalwai S, Agrawal S, et al. Indian Academy of Pediatrics Guidelines on Screen Time and Digital Wellness in Infants, Children and Adolescents. Indian Pediatr. 2022;59: 235–244.

17. Buabbas A, Hasan H, Shehab AA. Parents' Attitudes Toward School Students' Overuse of Smartphones and Its Detrimental Health Impacts: Qualitative Study. JMIR Pediatr Parent. 2021;4: e24196. doi:10.2196/24196

18. Throuvala MA, Griffiths MD, Rennoldson M, Kuss DJ. Policy recommendations for preventing problematic internet use in schools: A qualitative study of parental

perspectives. Int J Environ Res Public Health. 2021;18. doi:10.3390/ijerph18094522

19. Sharp CA. Qualitative Research and Evaluation Methods (3rd ed.). Evaluation Journal of Australasia. 2003. pp. 60–61. doi:10.1177/1035719X0300300213

20. Nwankwo F, Shin HD, Al-Habaibeh A, Massoud H. Evaluation of Children's Screen Viewing Time and Parental Role in Household Context. Glob Pediatr Heal. 2019;6: 2333794X19878062. doi:10.1177/2333794X19878062

21. Marsh S, Chu JTW, Calder AJ. "I tried to take my phone off my daughter, and i got hit in the face": a qualitative study of parents' challenges with adolescents' screen use and a toolbox of their tips. BMC Public Health. 2024;24: 217. doi:10.1186/s12889-024-17690-z

22. Muppalla SK, Vuppalapati S, Reddy Pulliahgaru A, Sreenivasulu H. Effects of Excessive Screen Time on Child Development: An Updated Review and Strategies for Management. Cureus. 2023;15: e40608. doi:10.7759/cureus.40608

23. Raj D, Zulkefli NABM, Minhat HS, Ahmad N. Parental Intervention Strategies to Reduce Screen Time Among Preschool-aged Children: A Systematic Review. Malaysian J Med Heal Sci. 2022;18: 295–304. doi:10.47836/mjmhs18.6.38

24. Sharma H, Ahuja S. Mitigating factors of screen time on children: a systematic review. Int J Contemp Pediatr. 2024;11: 1637–1643. doi:10.18203/2349-3291.ijcp20243092

25. Qi J, Yan Y, Yin H. Screen time among school-aged children of aged 6–14: a systematic review. Glob Heal Res Policy. 2023;8: 12. doi:10.1186/s41256-023-00297-z

26. Choi EJ, King GKC, Duerden EG. Screen time in children and youth during the pandemic: A systematic review and meta-analysis. Glob Pediatr. 2023;6: 100080. doi:https://doi.org/10.1016/j.gpeds.2023.100080

27. Sulistyo PB. Digital Literacy Competence of Parents in Supervising Their Children Using

Digital Media. Int J Soc Sci Hum Res. 2022;05: 636–642. doi:10.47191/ijsshr/v5-i2-32
28. Ahmed KR, Kolbe-Alexander T, Khan A. Effectiveness of a school-based intervention on physical activity and screen time among adolescents. J Sci Med Sport. 2022;25: 242–248. doi:10.1016/j.jsams.2021.10.007
29. Braun V, Clarke V. Using thematic analysis in psychology. Qual Res Psychol. 2006;3: 77–101. doi:10.1191/1478088706qp063oa
30. Nowell LS, Norris JM, White DE, Moules NJ. Thematic Analysis: Striving to Meet the Trustworthiness Criteria. Int J Qual Methods. 2017;16: 1–13. doi:10.1177/1609406917733847
31. Alcántara-Porcuna V, Sánchez-López M, Martínez-Vizcaíno V, Martínez-Andrés M, Ruiz-Hermosa A, Rodríguez-Martín B. Parents' Perceptions on Barriers and Facilitators of Physical Activity among Schoolchildren: A Qualitative Study. Int J Environ Res Public Health. 2021;18. doi:10.3390/ijerph18063086
32. Marconnot R, Marín-Rojas AL, Delfa-de-la-Morena JM, Pérez-Corrales J, Gueita-Rodríguez J, Fernández-de-Las-Peñas C, et al. Recognition of Barriers to Physical Activity Promotion in Immigrant Children in Spain: A Qualitative Case Study. Int J Environ Res Public Health. 2019;16. doi:10.3390/ijerph16030431
33. Charan Gopal Singh, Kalia Raman, Khurana Mandeep Singh, Narang Gursharan Singh. From Screens to Sunshine: Rescuing Children's Outdoor Playtime in the Digital Era. J Indian Assoc Child Adolesc Ment Heal. 2024;20: 11–17. doi:10.1177/09731342241229845
34. Liu S, Chang F-C, Chiu C-H, Li F, Chen P-H, Chen C-Y, et al. Parent-Child Discrepancies in Reports of Exposure to Violence/Pornography on Mobile Devices and

the Impact on Children’s Psychosocial Adjustment. Cyberpsychol Behav Soc Netw. 2021;24: 390–398. doi:10.1089/cyber.2020.0120

35. Romero M. Digital literacy for parents of the 21st century children. 2014; 32–40.

36. Terras MM, Ramsay J. Family Digital Literacy Practices and Children’s Mobile Phone Use. Front Psychol. 2016;7: 1957. doi:10.3389/fpsyg.2016.01957

37. Teke B, Ayar D. The Impact of Parents’ Digital Awareness on their Digital Attitude Behaviors. Compr Child Adolesc Nurs. 2025;48: 139–151. doi:10.1080/24694193.2025.2511111

38. El G, Moawad NA, Gad G, Ebrahem S. The Relationship between Use of Technology and Parent-Adolescents Social Relationship. J Educ Pract. 2016;7: 168–178. Available: www.iiste.org

39. Schneider LA, King DL, Delfabbro PH. Family factors in adolescent problematic Internet gaming: A systematic review. J Behav Addict. 2017;6: 321–333. doi:10.1556/2006.6.2017.035

List of tables:

Table 1. Interview method and sampling

Method	Type of schools		Type and number of participants		
	Bangla	English	Children	Parents	Teacher
In-depth interviews (IDIs)	6	6	5	3	4
Focus Group Discussions (FGDs)	3	3	20	24	-

Table 2: Participant demographics (N=25).

Variable	Frequency (n)	Percentage (%)
**Sex**		
Male	14	56.0
Female	11	44.0
**Age (in years)**		
12	12	48
13	10	40
14	3	12
**Studying class/ grade**		
6th	2	8.0
7th	2	8.0
8th	12	48.0
9th	8	32.0
10th	1	4.00
**School type**		
Bangla Medium	16	64.0
English Medium	9	36.0
**Occupation of father**		
Business	16	64.0
Service holder	3	12.0
Labor	2	8.0

Skilled worker	1	4.0
Others	3	12.0
**Occupation of Mother**		
Housewife	23	92.0
Skilled worker	2	8.0
**Type of family**		
Nuclear	16	64.0
Extended	9	36.0
**Device owned by participant**		
Yes	15	60.0
No	10	40.0
**Type of internet access**		
Wi-Fi or Broadband	23	92.0
Mobile data	2	8.0
**Type of device used by the participant [multiple response]**		
Smart phone	25	100.0
Smart television	19	76.0
Laptop	11	44.0
Tablet	9	36.0
Computer	8	32.0

T~~Full t~~itle: Parental monitoring challenges and recommendation for managing excessive screen use among ~~adolescents~~ students aged 12–14 years in Dhaka, Bangladesh: a qualitative exploration

~~**Short title: Challenges and recommendations for screen use in adolescents: a qualitative study**~~

Authors:

Shahria Hafiz Kakon[1*], Nahian Sultana[2], Bidhan Krishna Sarker[3], Tanjir Rashid Soron[4], Rashidul Haque[1], Fahmida Tofail[2]

Affiliations:

[1]Infectious Diseases Division, International Centre for Diarrheal Disease Research, Bangladesh, Dhaka, Bangladesh

[2]Maternal and Child Nutrition, Nutrition Research Division, International Centre for Diarrheal Disease Research, Bangladesh, Dhaka, Bangladesh

[3]Maternal and Child Health Division, International Centre for Diarrheal Disease Research, Bangladesh, Dhaka, Bangladesh

[4]Telepsychiatry Research and Innovation Network Ltd, Dhaka, Bangladesh

***Corresponding Author:**

Dr. Shahria Hafiz Kakon

Project Research Manager,

Infectious Diseases Division,

Style Definition: Heading 1: Font color: Text 1, Space Before: 0 pt, Line spacing: 1.5 lines

Style Definition: Heading 2: Font: Times New Roman, Bold, Indent: Left: 0"

Style Definition: Heading 3: Font: Bold

Style Definition: Heading 4: Font: Times New Roman, Bold

Style Definition: Quote: Justified, Line spacing: 1.5 lines

Formatted: Space After: 0 pt, Line spacing: Double

Formatted: Font: Not Bold

Formatted: Space After: 0 pt, Line spacing: Double

Formatted: Line spacing: Double

Formatted: Line spacing: Double

International Centre for Diarrheal Disease Research, Bangladesh (icddr,b)
68, Shaheed Tajuddin Ahmed Sarani,
Mohakhali, Dhaka 1212,
Bangladesh
Phone: +8801726428760;
E-mail: shahriahafiz@icddrb.org

Abstract ~~(300 words)~~

Background

The pervasive use of ~~Of late,~~ social media and recreational internet platforms among children has become increasingly evident ~~use are undeniable~~. The proper use of devices for children is critical; overindulgence leads to a negative impact on children in many areas of their lives.

~~Objectives~~

This study aimed to explore ~~investigated~~ the challenges ~~that~~ parents face when their children engage in excessive use electronic digital screens~~ excessively~~. Additionally, ~~I~~it ~~also consists of~~ sought to capture participants' recommendations ~~from the participants~~ for the best use of digital devices among ~~by adolescent~~ children.

Methods

~~The study used~~ An~~a~~ exploratory qualitative study design was employed to understand parental experience in regulating screen use and ~~approach and revealed the obstacles parents encountered to ensure the quality and duration of screen use, as well as some~~ strategies adopted to manage its duration and quality~~for regulating this element~~. Data were collected ~~from~~ between January and

Formatted: Font: 12 pt
Formatted: Space After: 0 pt, Line spacing: Double
Formatted: Heading 1, Left, Line spacing: Double
Formatted: Font: 12 pt, Character scale: 100%
Formatted: Normal, Space Before: 0 pt, Line spacing: Double
Formatted: Font: 12 pt, Character scale: 100%, Not Expanded by / Condensed by
Formatted: Font: Character scale: 100%, Not Expanded by / Condensed by
Formatted: Font: (Default) Times New Roman, 12 pt
Formatted: Line spacing: Double
Formatted: Font: (Default) Times New Roman, 12 pt, Bold
Formatted: Font: 12 pt, Character scale: 100%
Formatted: Font: 12 pt, Not Bold, Character scale: 100%
Formatted: Font:
Formatted: Font: (Default) Times New Roman, 12 pt
Formatted: Space After: 0 pt, Line spacing: Double

~~2024 to~~ April 2024. ~~Adolescents~~ Children aged between 12 and 14 who attending~~ed~~ class six to ten ~~different~~ English and Bangla medium schools in Dhaka~~;~~ and their parents and teachers were the participants in this study. D~~The d~~ata was collected~~ion~~ ~~methods~~ through ~~were~~ focus group discussions and different type of ~~,~~ in-depth interviews~~, and key informant interviews~~. A thematic analysis was performed ~~Data were analyzed~~ using ~~the~~ inductive ~~method~~ approach~~and presented in a narrative qualitative manner.~~.

Results

We included ~~Results displayed~~ 25 children who attending between classes six~~6~~ and ~~10~~ten, of whom ~~, where~~ 56%~~-~~ were male~~s~~. Key challenges identified by parents included excessive use of devices for social media and recreational purposes beyond academic purposes, ensuring safe and conductive outdoor environments, monitoring age-appropriate content, and a lack of digital literacy among parents.~~The core challenges were the recent excessive use of devices for social media and recreational internet besides learning and academics, ensuring safe and proper environments in the neighborhoods for outdoor play, confirming the viewing of age-appropriate content and lastly, a deficit of digital literacy in the parents.~~ Participants' recommendations comprehended school-based awareness interventions, national level guideline regarding the use of digital devices, ~~Recommendations received were school-based interventions, national level policies,~~ development of self-regulatory behavior in children, provision of secured recreational infrastructures, create scope outdoor recreational activities among children, enhancing the parent-child bond and offering interesting options to divert the children from screens.

Conclusions

The study findings provide a better understanding of the challenges of overuse of devices and recommendations among the population. Effective guidelines for managing students' screen use

Formatted: Font: (Default) Times New Roman, 12 pt, Bold

Formatted: Font: 12 pt, Character scale: 100%

Formatted: Font:

Formatted: Normal, Space Before: 0 pt, Line spacing: Double

Formatted: Font: (Default) Times New Roman, 12 pt, Bold

Formatted: Space After: 0 pt, Line spacing: Double

Formatted: Font: (Default) Times New Roman, 12 pt

Formatted: Font: 12 pt, Character scale: 100%

Formatted: Normal, Line spacing: Double

Formatted: Font: Character scale: 100%

Formatted: Font: (Default) Times New Roman, 12 pt

Formatted: Space After: 0 pt, Line spacing: Double

require ~~the participation~~participation from schools, parents, legislators and students themselves. Further research is required to determine the most effective interventions.

Keywords Digital Device; Children;

Screen viewing;~~ device; adolescents;~~ Use of ~~gadgets; s~~social media; Bangladesh

~~challenges; recommendations;~~

~~focus group discussion; in-depth interviews and key informant interviews.~~

Introduction

The presence~~reign~~ of technology in everyday life is undeniable. The boundaries between digital and reals worlds have blurred, and children are increasingly immersed in virtual media, gaming, and online communication ~~In today's age, the blend of the real and digital world has become inevitable~~ [1]~~. The alloy of virtual media, gaming and recreation has collectively integrated into our daily lives~~ [2]. While digital tools can provide educational and recreational benefits, excessive screen viewing (SV) has been associated with adverse impacts on children's health and development ~~Despite various confounding factors playing a role in determining the adverse impacts of excessive screen viewing (SV), popular opinion remains that unrestricted screen time has tremendous adverse effects on the health and development of children~~ [3]. Evidence reported ~~Research suggests~~ that the role of socio-economic status (SES) in shaping parenting practices related to technological use. is a highly influential element in the parenting process. Four factors were depicted in this regard. For example, higher-SES parents often struggle to reconcile concerns about harmful impacts with the perceived need for children to develop digital skills ~~Firstly, higher-~~

Formatted: Font: (Default) Times New Roman, 12 pt

Formatted: Space After: 0 pt, Line spacing: Double

Formatted: Font: Not Bold

Formatted: Font: Not Italic

Formatted: Font: Not Italic

Formatted: Font: Not Italic

Formatted: Font: Not Italic

Formatted: Justified, Space After: 0 pt, Line spacing: Double

Formatted: Font: Not Italic

Formatted: Line spacing: Double

Formatted: Heading 1, Left, Line spacing: Double

Formatted: Space After: 0 pt

~~SES parents were indecisive about technology use, concerned about its harmful impacts on children its limitations, and also imploring technological skills' potential to mitigate predicted career deficits of children. Secondly, these parents perceived that there were discrepancies in the established recommendations~~ [4]. Parenting styles also influence children's technology habits, with some parents permitting more device use as part of an individualized parenting approach, even when this exceeds pediatric guidelines ~~Thirdly, parenting style where parents intentionally structure their children's lives to help them succeed in the future. which emphasized children's dealings with adults, resulting in children creating situations where parents permitted them to use devices more than they would naturally prefer. Lastly, these processes motivated higher SES parents to build individualist parenting approaches that typically resulted in children's technology use far exceeding pediatric guidelines~~ [5].

Evidence links prolonged SV with negative outcomes such as poorer mental health, sleep disturbance, reduced academic performance, and behavioral problems, although in some cases, screen use has also been associated with improved ~~A study controlling the socio-economic factors and ethnicity done on adolescent children about the impacts of excessive screen viewing revealed that more screen time was moderately linked to worse mental health, raised behavioral problems, lowered academic performance and poorer quality of sleep, but increased quality of~~ peer relationships [6]. ~~In addition, a~~Another recent paper on-screen viewing and Adolescent Brain Cognitive Development (ABCD) ~~reported~~ further indicated associations between ~~that screen time on the~~ high screen time and depressive symptomse, conduct disorders, and attention-deficit/hyperactivity disorder~~,~~ ~~end was linked to all sorts of mental problems in adolescents; the commonest problems detected being depressive moods, somatic disorders, conduct disorders and attention deficit hyperactivity disorders disorder (ADHD)~~ [7]. In addition, the type of screen

Formatted: Space After: 0 pt, Line spacing: Double

activity matters: texting, video, chats, video streaming, and gaming show stronger association with mental health challenges compared to other forms of use ~~Also, the particular screen types with the highest associations with depressive symptoms were video chat, texting, videos and video games~~ [7]. A relevant quantitative study on children about screen time reported that the class year was positively and significantly associated with the average time spent on electronic devices during school days, weekends, and holidays [8].

In ~~the context of~~ Bangladesh, a recent study ~~relevant paper on adolescents~~ reported that recreational SV is rising rapidly among adolescents. The study found that around 79% of the adolescents had increased recreational screen time [9]. Median reported recreational screen time was found to be 4.0 hours/day; with boys having longer times (4.3 hours/day) compared to girls (3.6 hours/day). Further the study reported that high SV was associated with unhealthy behaviors, such as frequent fast-food consumption, difficulty falling asleep, and higher SES [9]. ~~Multivariable analyses revealed that higher screen time was more common among boys than girls and was positively associated with travelling to school by car, intake of fast food ≥3 times/week, having trouble falling asleep and higher SES.~~ In the recent cutting-edge and modernized era, apart from regular television viewing, children are engaged in a widespread array of devices such as tablets, smartphones, game consoles, laptops and so on [9]. The trend of using more than one device simultaneously is even observed nowadays [2]. Combining online platforms and using Internet media for a number of purposes, such as academic assignments, social communication, and leisure, is a common phenomenon [10]. Due to widespread internet and digital media use lately, previous screen viewing recommendations by the American Association of Pediatrics (AAP) have undergone significant revisions. A "Family Media Use Plan" is now recognized, which helps families to establish an equilibrium between screen time and other activities with no set boundary

for screen viewing [2,10]. However, no such tailored guidelines currently exist in the Bangladesh context.

Parents, being the primary guardians responsible for providing a healthy environment and shaping the children's future, face a lot of obstacles. With the rapid pace of innovation, particularly in middle-income countries, the restrictions on screen viewing tend to be less effective as they undermine opportunities and developments [11]. Needless to mention, with the hectic schedules of parents and caregivers, it is challenging to keep an eye on the duration of screen time and the type of content that children are exposed to [11]. The debate of concern between protecting children from internet addiction, cybercrime and empowering children for the future with enhanced digital expertise is the sole challenge [12]. Studies across diverse contexts highlights the concern. For example, ~~A~~ Canadian ~~study conducted focus groups with~~ parents (85% mothers) of children reported that, despite knowing of the negative impacts of screen time, some parents valued screen viewing as a behavior management strategy or by using SV as an 'electronic babysitter' to manage ~~so that parents could complete~~ their household ~~chores~~ demands [13]. I~~A study i~~n Spain, restrictive schedules and safety concerns limited children’s opportunities for the physical activity, especially for girls ~~revealed that the principal barrier to physical activity participation in children, particularly girls, was creating more restrictive and sedentary schedules. The paper suggested enhancing the safety of recreational parks as a solution, emphasizing the gender-sensitive perspective~~ [14]. In a Dutch ~~qualitative analysis~~study, self-interests such as doing tasks undisturbed, creating me-time, regulating children's behavior, avoiding arguments and being able to use devices themselves were the core findings [15]. A recent study conducted by the Indian Academy of Pediatrics suggests that not only SV but other involvements such as outdoor physical activities, sleep, family and peer interaction, education and skill building, which are vital for

overall health and development, should all form a part of the daily lives of the children and adolescents [16]. Families should provide an encouraging and fun-filled environment at home, as well as supervise their children's SV to ensure that the content being watched is educational, age-appropriate and non-violent [16]. A healthy Family Media Use Plan, as previously mentioned, as recommended by AAP, can create an optimum balance between SV and other daily activities, encourage age-appropriate critical thought processing digital literacy and lastly, implement elemental rules about the usage of media [10]. whereas, parents in Kuwait family-based strategies, including A qualitative study done in Kuwait in 2021 on parents regarding children's usage of digital gadgets suggested some as follows: increase familial socialization, encourage children to join health clubs, use reward techniques, arrange regular sports competitions, establish entertainment centers at each region to manage SV and so on [17] and . Another study in the parents in UK in 2021 reported that,identified school-based interventions could play a vital role alongside the parent's roles to prevent excessive use , enhance parent-child interaction and aid in lowering inter-familial conflicts. According to those parents, on thematic analysis it was deducted that, schools were "prevention hubs" [18].

Despite the growing body of evidence, there remains a lack of research exploring how parents in low-and middle-income countries (LMICs) navigate these challenges. In Bangladesh, where digital access is rapidly expanding but parental guidance and protecting structure are limited, understanding these dynamics is particularly important. This study therefore aimed to explore To date, there is a lack of research focusing on the conflicts faced by parents in regulating SV in children. It is imperative to attain a "digital balance" within families to achieve symmetry between the need to remain technologically skilled and the minimization of the chances of internet addiction in children. The aim of this qualitative narrative was to examine the parental challenges parents

Formatted: Space After: 0 pt

face in for supervising and restricting children's SV in urban Dhaka, Bangladesh, and to gather their feedback on strategies that may help achieve a healthier balance between digital engagement and overall child well-being.of children as well as gather specific feedback on how to effectively utilize SV within urban Bangladesh context.

Formatted: Space After: 0 pt, Line spacing: Double

Material and Methods

Formatted: Heading 1, Left, Line spacing: Double

Study dDesign and methods of data collection

This study employed an exploratory qualitative design with standard qualitative tools being used to gather information on our research interest. We conducted qualitative interview from the same schools where previously quantitative study was performed. We collected data from parents, students and teachers which provided a holistic understanding of the phenomena and a detailed contextual explanation for quantitative result. Methods were applied with purposive sampling of different types of informants. cross-sectional study employed multiple qualitative methods to achieve its research objectives.A combination of multiple tools, including Data collection methods included in-depth interviews (IDIs) with parents, children and teachers and focus group discussions (FGDs) with children and their parents, was utilized to understand experiences, perceptions, and recommendations- regarding the use of screen timeand children., as well as key informant interviews (KIIs) with teachers. The use of multiple methods facilitated the collection of both individual and collective perspective. Such methodological triangulation is recognized for enhancing the trustworthiness of qualitative findings. These methods were employed to comprehensively explore the challenges and gather recommendations for effectively monitoring children's screen time.

Study settings~~, participants and the sampling procedure~~

We conducted this study in Dhaka city. We purposively selected three Bangla-medium and three English-medium schools from Dhaka North and Dhaka South city corporations. Dhaka has been selected as the center of the country with dominant presence of high socioeconomic community considering over exposure to digital screens. Both Bangla and English-medium schools have been chosen in order to ensure the equal distribution of the Bangla and English medium schools for the representation of diverse educational systems, socioeconomic status, and geographical context. During the school selection, we also considered with the kind permission of the school authorities, children aged between 12 to 14 years, who were in class 6 to class 10, and who used more than two hours of screen time each day as the qualifying criteria for sample inclusion. Smartphones, tablets, laptops, PCs, gaming consoles, televisions, and portable gaming devices were among the acceptable gadgets. whereas, children who might have affected the results due to pre-existing conditions, such as physical or psychological impairment, were excluded from the study.

Study participants and sampling

We have included three types of ~~Data collection was carried out between January 2024 to April 2024 involved six purposively selected schools - three Bangla medium and three English medium - from North and South Dhaka City Corporations to ensure representation of diverse educational systems, socioeconomic backgrounds and geographical contexts aligning with the study's objective to explore variations in educational experiences.~~

~~P~~participants~~ in this study included three groups~~: 1) ~~p~~Parents whose children exhibit excessive use of digital devices were interviewed as they are the primary caregivers and ~~often the ones~~ who set

Formatted: Space After: 0 pt, Line spacing: Double

Formatted: Heading 2, Left, Line spacing: Double

Formatted: Space After: 0 pt, Line spacing: Double

Formatted: Heading 2, Left, Line spacing: Double

Formatted: Space After: 0 pt, Line spacing: Double

Formatted: Font: (Default) Times New Roman, 12 pt

rules and boundaries regarding screen time~~ to gain insight into their experiences of challenges in managing their children's screen time~~. 2) c~~C~~hildren with experience of excessive digital device ~~were ~~used were interviewed to understand their experiences ~~in which they ~~of engagement pattern and ~~with digital devices and how they ~~perceptions of~~ive their~~ screen time limits. 3) t~~T~~eachers were interviewed to develop potential ~~solutions ~~strategies for managing screen time as they play a significant role in children's development and education.

A heterogeneous sampling technique ~~diversified group of respondents ~~was utilized for IDIs, while a homogeneous technique was applied for FGDs [19]. IDIs with children and parents were conducted separately after school hours to ensure sufficient time and minimize distractions. FGDs were also organized separately with children and parents, considering diverse class, age, sex to capture diverse perspectives. A total of 12 IDIs and six FGDs were conducted across six schools, which was sufficient to reach thematic saturation. Table 1 provides detailed information about the study participants below.

Formatted: Space After: 0 pt, Line spacing: Double

[Table 1 to be inserted here]

Formatted: Centered

~~was purposively selected to ensure a comprehensive understanding of the research objects, incorporating a wide range of perspectives and experiences. Children along with their parents and teachers, were purposively selected from the class register book. For selecting IDI participants, we chose children and parents for separate individual interviews in the school after school hours to ensure they had sufficient time and could participate without distractions. Similarly, we conducted separate Focus Group Discussions (FGDs) with children and parents, considering diverse class, age, and gender to capture a wide range of perspectives and experiences which ensured the~~

Formatted: Space After: 0 pt, Line spacing: Double

discussions reflected the varied backgrounds of participants and encouraged richer insights through different viewpoints.

We selected class teachers for Key Informant Interviews (KIIs) as they are directly involved in the academic environment and can provide valuable insights into challenges and opportunities within the school system, helping inform policy decisions.

Formatted: Line spacing: Double

There are no known guidelines to determine non-probabilistic sample sizes. Point of saturation or point of redundancy has been done and still is the "intellectual guiding principle" in terms of deciding how many interviews to conduct for a qualitative study with the purposive sampling as its basis. As mentioned, it is only an intellectual guiding principle with little or no on-the-ground guide to sample size estimation that will be required to reach this saturation [19]. To comprehend the challenges and recommendations for monitoring screen use, total 18 interviews were conducted; including 8 IDIs, 4 KIIs and 6 FGDs sessions was conducted. The table 1 below provides detailed information about the sessions and the participants in six schools.

Table 1: The sample distribution of each school.

**SL. No.**	**School Types**	**In Depth Interviews/ Key Informant Interviews**			**Focus Group Discussions**	
		**Students**	**Teachers**	**Parents**	**Parents**	**Students**
1.	English	1	1		11	
2.	English	1		1	6	
3.	English	1	1			6
4.	Bangla	1	1			7
5.	Bangla		1	1		7

Formatted: Line spacing: Double

Formatted: Line spacing: Double

Formatted: Line spacing: Double

Formatted: Line spacing: Double

Formatted: Line spacing: Double

Formatted: Line spacing: Double

~~6.~~	~~Bangla~~	~~1~~		~~1~~	~~7~~	
	~~Total participants~~	~~5~~	~~4~~	~~3~~	~~24~~	~~20~~

Data collection ~~procedure~~ method and quality checking

Data collection was carried out between January 2024, and April 2024 using a combination of audio recording and note taking. Three separate semi-structured guidelines were developed based on the study focus and literature review [6,7,13,17,20–28]: one each for children, parents and teacher. The guidelines focused on the challenges and recommendations for monitoring screen time for children. The tools were translated from English to Bengali prior to use. All tools were pre-tested with 6 non-study participants to ensure clarity and appropriateness.

Each IDI lasted approximately 40-60 minutes and FGD up to maximum 90 minutes. The interviews were conducted in teachers' common room at the end of the class, allowing the participants to speak freely about the topic. For FGDs, the participants were seated in a circle, and all interviews were audio recorded. Nobody allows the interview room during sessions and maintained confidentiality. All the interviews were conducted in Bangla, the national language of Bangladesh and privacy was maintained throughout the interview. Interviews were conducted by a Senior Research Officer who has more than five years of qualitative research experience. On the other hand, interview note has been taken by a Field Research Assistant who has more than three years of experience in qualitative research. Both of whom received rigorous training prior to data collection. There is also a Mental Health Expert who has observed the interviews team and provided feedback where necessary. Interviewers were responsible for transcribing, coding, and summarizing the interviews. Further, to ensure data credibility and dependability, we engaged

Formatted: Line spacing: Double

Formatted: Line spacing: Double

Formatted: Line spacing: Double

Formatted: Heading 2, Left, Line spacing: Double

Formatted: Space After: 0 pt, Line spacing: Double

Formatted: Not Highlight

Formatted: Not Highlight

multiple types of informants including parents, children and teacher to gather diverse experience shared by the participants.

Operational definitions~~The data collection process involved a combination of audio recording and note-taking. Based on the study objectives and a review of the existing literature, we developed three separate guidelines: one for students, one for parents and another for teachers. FGDs and IDIs highlighted the challenges and recommendations of supervising screen time of children. After drafting, the tools were translated from English to Bengali. Data collection was carried out by a field research assistant and a senior research officer, who had more than three and five years of experiences on qualitative data collections methods.~~

Screen Viewing (SV): The time children spend using digital devices for any purpose, including educational, recreational, or social activities.

Digital Device: Any electronic device that allows access to digital content, such as smartphones, tablets, laptops, Television, computers, and game consoles.

Digital Platform: Online services or applications accessed through digital devices, including websites, social media, educational tools, and entertainment platforms.

Digital Literacy: The ability to effectively and safely use digital devices and platforms, including understanding content, managing screen time, and navigating online resources.

Outdoor Activity: Any physical or recreational activity performed outside the home, including sports, games, creative play, and other forms of physical exercise.

Formatted: Line spacing: Double

Formatted: Font: 12 pt

Formatted: Font: 12 pt, Not Bold

Formatted: Normal, Justified

Formatted: Font:

Formatted: Font: 12 pt

Formatted: Font: 12 pt, Not Bold

Formatted: Font: 12 pt, Not Bold

Formatted: Font:

Formatted: Font: 12 pt

Formatted: Font: 12 pt, Not Bold

Formatted: Font:

Formatted: Font: 12 pt

Formatted: Font: 12 pt, Not Bold

Formatted: Font:

Formatted: Font: 12 pt

Formatted: Font: 12 pt, Not Bold

Formatted: Font:

Peer Interaction: Social engagement and communication with other children or adolescents, either in-person or virtually, fostering social skills and relationships.

Inappropriate Content: Digital material that is harmful, age-inappropriate, or culturally unacceptable, such as violent, sexual, or drug-related content.

Social Safety: The provision of a secure and supervised environment for children to engage in activities, reducing exposure to harm and promoting physical, emotional, and social well-being.

~~**Separate guidelines were prepared and pre-tested to conduct IDI, FGD and KII with 5-6 non-study participants prior to data collection. Semi-structured guidelines were followed to conduct in-depth interviews, limited to approximately 1.5 hours. On the other hand, focus group discussion lasted a maximum of 2 hours, and key informant interviews were open-ended designed, which lasted approximately one hour. The interviews** were conducted in the teacher's common room during an off period where the participants could speak freely about the topic. The participants were sitting on chairs arranged in a circle. **All interviews were audio recorded.** One researcher acted as the facilitator, and the other used the recorder. **Privacy was maintained when administering all data procedures. All interviews were carried out in Bangla, the national language of Bangladesh.** The facilitator encouraged the discussion and involvement without being placed in the role of expert. She also kept the time distribution equal for each topic to maintain interest. **Data collectors were responsible for transcribing, coding as well as summarizing the interviews. A senior research officer and a mental health expert closely monitored the data collection team and provided necessary feedback during data collection to discuss the findings and modification of instruments.**~~

Formatted: Font: 12 pt

Formatted: Font: 12 pt, Not Bold

Formatted: Font:

Formatted: Font: 12 pt

Formatted: Font: 12 pt, Not Bold

Formatted: Font:

Formatted: Font: Not Bold

Formatted: Font: Not Bold

Formatted: Font: Not Bold, Not Expanded by / Condensed by

Formatted: Font: Not Bold

Formatted: Font: Not Bold, Not Expanded by / Condensed by

Formatted: Font: Not Bold

Formatted: Font: Not Bold, Not Expanded by / Condensed by

Formatted: Font: Not Bold

Formatted: Font: Not Bold, Not Expanded by / Condensed by

Formatted: Font: Not Bold

Formatted: Font: Not Bold, Not Expanded by / Condensed by

Formatted: Font: Not Bold

Formatted: Font: Not Bold, Not Expanded by / Condensed by

Formatted: Font: Not Bold

Formatted: Font: Not Bold, Not Expanded by / Condensed by

Formatted: Font: Not Bold

Formatted: Font: Not Bold, Not Expanded by / Condensed by

Formatted: Font: Not Bold

Formatted: Font: Not Bold

Formatted: Font: Not Bold

Formatted: Font: Not Bold, Not Expanded by / Condensed by

Formatted: Font: Not Bold

Formatted: Font: Not Bold, Not Expanded by / Condensed by

Formatted: Font: Not Bold

Formatted: Font:

Formatted: Space After: 0 pt, Line spacing: Double

~~**To ensure data reliability, we explored different types informants including parents, children and teachers by conducting interviews in order to gather diverse experiences shared by participants. Data validity was maintained by cross-referencing specific concepts and confirming information with multiple sources. To determining the validity of this qualitative inquiry, we employed three types of data collection tools to achieve methodological, investigator, and data source triangulation. For methodological triangulation, we used IDI, FGD and KII to cross-check information and ensure consistency. Multiple supervisors and investigators were involved to minimize bias and ensure a comprehensive interpretation of the data. Additionally, to validate the findings across various contexts, we collected data from different participants, settings, and time periods.**~~

Formatted: Heading 2, Left, Space Before: 0 pt, Line spacing: Double

Data analysis

Formatted: Heading 2, Left, Line spacing: Double

~~The first stage of qualitative d~~Data analysis began with~~by~~ organizing and preparing the field data, including interviews and field notes. To ~~he~~ maintain confidentiality recorded interviews were assigned ~~re-named with~~ symbolic identifiers. ~~to maintain confidentiality.~~ All the~~Then, the~~ interviews~~data~~ were transcribed verbatim in~~to~~ Bangla and reviewed by the principal investigator ~~during the study~~. ~~While conducting interviews,~~ ~~r~~Relevant notes were collected during interviews and field observation were also incorporated into the analysis.

Formatted: Line spacing: Double

~~All the field notes and interviews (with or without recording) were considered for the data analysis process and transcribed similarly.~~ The transcripts were read thoroughly ~~then carefully examined~~ for data familiarization, which guided ~~leading to~~ the development of a codebook ~~for data coding, which was done~~ using the inductive approach~~es~~. ~~Coding of interviews KII,~~ IDI and FGD data were coded in the appropriate code~~matrixes~~s. The analysis team collaboratively defines and re-defines

codes. discussed, defined, and applied the codes to the data. ThroughT thematic analysis then conducted to identify , common themes and sub-themes related towere identified to explore the challenges and recommendation as per research objectives [29]. A summary was created for analyzing the themes and sub-themes to identify challenges and recommendation. The findings were then compiled and summarized for all the sites.

To enhance rigor, The use of data and methodological data triangulation was applied at multiple levels: methodological (IDI and FGD), researchers (multiple researchers), and data sources (different type of participants). These strategies enhance trustworthiness, minimize potential bias, and ensure that the findings reflected the richness and diversity of different perspectives [30]. enabled comprehensive analysis across different research sites and methods. Comparisons between key informants, parents, and students enhanced the validity of the findings.

Ethical approval

The study protocol was approved by the Institutional Review Board (IRB) of icddr,b (Protocol No: PR-22002). Informed wWritten informed consent was taken from each respondent (parents, teacher) and children was taken consent from their parents as well as assent was taken as their age was above 11 years prior to the interview. All the respondents were informed in Bengali about their rights related to their voluntary participation in the study and withdrew from the interview at any time during the interview. Interviewers of this study clearly conveyed to the respondents the objectives of the study, reasons for their participation in the study, their role, risks, benefits, anonymity and confidentiality, including the rights not to participate or withdraw. After getting acquainted with all these issues, the respondents decided whether to participate in this study. Confidentiality during and after data collection was ensured, and no identifiable information was

Formatted: Heading 2, Left, Line spacing: Double

Formatted: Line spacing: Double

used that may expose the identity of the participants. Only the research team members have access to the collected data, which were stored securely. After the interview, each participant received a gift (equivalent to BDT370 [USD3] as a token of appreciation for their valuable time.

Results

Background Characteristics of participants

The demographic characteristics of the participants are illustrated in Table 2. All the children were between 12 and 14 years of age; and 56~~.0~~% of them were males. The majority of them (48~~.0~~%) were students of class 8, hailed from Bangla-~~-~~medium schools (64~~.0~~%) and belonged to nuclear type of families (64~~.0~~%). Most of the fathers of children are involved in ~~were doing~~ business (64~~.0~~%), whereas mothers were housewives (92~~.0~~%). The majority of the children (60~~.0~~%) had at least one device and a substantial percentage (92%) was ~~were WiFi~~Wi-Fi users. ~~(92.0%).~~ They were found to be using multiple devices, meaning 100~~.0~~% used smartphones followed by 76~~.0~~% had access to smart TVs.

[Table 2 to be inserted here]

~~**Table 2: Participant demographics (n=25).**~~

~~**Variables**~~	~~**Attributes**~~	~~**Frequency (f)**~~	~~**Percentage (%)**~~
~~Gender~~	~~Male~~	~~14~~	~~56.0~~
	~~Female~~	~~11~~	~~44.0~~
~~Age (in years)~~	~~12 to 14~~	~~25~~	~~100.0~~
~~Class~~	~~6th~~	~~2~~	~~8.0~~

Formatted: Not Highlight
Formatted: Heading 1, Left, Line spacing: Double
Formatted: Line spacing: Double
Formatted: Font: Bold
Formatted: Font: Bold
Formatted: Centered, Line spacing: Double
Formatted: Line spacing: Double
Formatted Table
Formatted: Line spacing: Double
Formatted: Line spacing: Double
Formatted: Line spacing: Double

	7th	2	8.0
	8th	12	48.0
	9th	8	32.0
	10th	1	4.00
Medium of education	Bangla Medium	16	64.0
	English Medium	9	36.0
Occupation of father	Business	16	64.0
	Service holder	3	12.0
	Others	3	12.0
	Labor	2	8.0
	Skilled worker	1	4.0
Occupation of Mother	Housewife	23	92.0
	Skilled worker	2	8.0
Type of family	Nuclear	16	64.0
	Extended	9	36.0
Device owned by participant	Yes	15	60.0
	No	10	40.0
Type of internet Access	WiFi or Broadband	23	92.0
	Mobile data	2	8.0
Type of device* used by the participant	Smart phone	25	100.0
	Smart television	19	76.0
	Laptop	11	44.0
	Tablet	9	36.0

Formatted: Line spacing: Double

Formatted: Line spacing: Double

Formatted: Line spacing: Double

Formatted: Line spacing: Double

Formatted: Line spacing: Double

Formatted: Line spacing: Double

Formatted: Line spacing: Double

	Computer	8	32.0

*Indicates multiple responses.

The holistic concept of screen viewing involving the duration, content, and safety issues was a concern for the parents. Meanwhile, students also had their own explanations and thoughts regarding the outcomes of excessive device use and teachers made their own observations and remarks. The line between digital skill development and internet addiction is razor-thin; it is difficult for parents and caregivers to strike this balance and maintain a harmonious and contented home environment.

An overview of the challenges facedfaced, and recommendations provided are discussed, with illustrative verbatim quotes. A lack of marked differences between the interviews and discussion responses was found, so the responses are presented here collectively, with no particular identifiers.

Challenges faced by parents for monitoring screen use

Students use digital device for education

Students use digital device for education

Parents highlighted the lack of sufficient guidance in the newly introduced education system, which increased children's reliance on mobile phones and the internet for academic purposes. They reported that teachers often did not provide adequate explanations in class, pushing students to depend on online resources. While parents provided mobile phones to support their children's learning, they observed that children frequently misused the devices for non-academic purposes, such as playing games, under the pretext of studying.Parents reported their limitations to restrict the overuse of gadgets and devices. They revealed their challenges in monitoring the children's screen time because various educational content is available on the digital platform. As a result,

Formatted: Line spacing: Double

Formatted: Space After: 0 pt, Line spacing: Double

Formatted: No underline, Font color: Auto

Formatted: Heading 2, Left, Line spacing: Double

Formatted: No underline

Formatted: Line spacing: Double

Formatted: Heading 3, Left, Line spacing: Double, No bullets or numbering

Formatted: Font: Times New Roman

Formatted: Space After: 0 pt, Line spacing: Double

~~parents are bound to provide digital devices like computers and mobile phones, although they doubt whether children were utilizing those devices for education. They assume that children at this age can deviate from studies easily since they are not mature enough to have a grip on themselves. They added that, currently, the teachers do not explain the topics in classrooms; rather, they encourage the students to find the solutions themselves at home, even mentioning that they should search "Google" to solve their queries. Consequently, most of the screen time takes place unsupervised as they cannot take away the devices for the excuse of using the device for academic needs. The situation turns more complicated for working parents as they cannot be available at home for a long time, so they are compelled to permit their children to use "WhatsApp" and "Messenger" as certain school notifications are often distributed through these social media. One parent from a Bangla medium school said,~~

> *"The books which are provided as per the new education system, there are no guidelines. So, students need to depend on using mobile phone to take help from the internet. Because teachers nowadays do not explain the topics in the classroom, they say their students to complete them at home. So, when students are given mobile phones for study, it is seen that sometimes they start to play games as an excuse for study."* ***(FGD; Parent; Bangla medium school)***

Students' reliance on the internet for learning support

Students reported that they were often directed by teachers to search Google or other online sources for solving classroom tasks. Many students mentioned that parents could not provide guidance on the updated curriculum, which further increased their dependence on the internet. They frequently formed online groups with classmates to complete assignments, using social networking platforms for communication. However, they admitted that such academic use of devices often led to

Formatted: Font: Not Italic

Formatted: Font: Not Italic

Formatted: Font: 12 pt, Italic

Formatted: Normal, Justified, Line spacing: Double

Formatted: Font: 12 pt

Formatted: Quote, Left, Line spacing: Double

Formatted: Font: 12 pt, Pattern: Clear

Formatted: Font: 12 pt

Formatted: Font color: Text 1

distractions, particularly from short videos, reels, and other entertainment content.Students exposed similar findings in the sense that they claimed that they needed to depend on the internet to find information as their class teachers did not explain many topics in class properly as per the instructions in the books. Hence, their class teachers suggest them to take help by searching Google as well as their parents are unable to guide them well on certain topics due to the updated education protocols. They added that they form a group with their classmates and connect online through social networking services as the class teachers give them assignments that they complete together using social networks. Consequently, they are bound to use the internet, which leads to difficulties concentrating on their studies since it distracts them from their studies due to various persuasive elements like notifications and eye-catching interfaces.

> *"There are many topics in the books that I do not know how to solve, and even my parents cannot help me with that, so they suggested I search Google. Actually, it often happens that I take permission from my mom to use the mobile phone for study purposes but I do not use it for study purposes. I usually view short videos. Short videos or reels are more fun than study".* ***(FGD; Student; Bangla medium school)***

> *"We often have group work, and for that we need to discuss things. Now if we don't have a phone, how will we discuss? That's why the phone is necessary."* ***(FGD; Student; Bangla medium school)***

Parents' perspectives on digital learning and supervision

All parents emphasized that as a result of excessive device use, their children's academic performance is directly impacted, resulting in lower grades.

> *"My son's roll number is dropping from 20 to 50. He only spends one hour on coaching and private tutoring. The rest of the day, he's glued to his mobile." (FGD; Parents; Bangla medium school)*

Formatted: Font: Times New Roman, 12 pt, Font color: Text 1

Formatted: Font: Times New Roman, 12 pt, Italic, Font color: Text 1

Formatted: Font: (Default) Times New Roman, 12 pt, Font color: Custom Color(RGB(13,13,13)), Pattern: Clear (White)

Formatted: Quote, Line spacing: Double

Formatted: Font: 12 pt

Formatted: Font: 12 pt

Formatted: Font color: Text 1, Pattern: Clear

Teachers’ perspectives on digital learning and supervision

Teachers expressed concerns about the increasing dependence on digital platforms from grade six onwards. While acknowledging the benefits of technology for learning, they noted that many students lacked the maturity to use devices responsibly. Teachers also observed that group work often created inequalities, where a small number of students bore the entire burden, while others misused devices for unrelated activities. They emphasized the importance of parental supervision and awareness to ensure productive screen use. ~~Echoing same voice, teachers showed concerns about the new curriculum's encouragement of online learning, starting from grade six, which increases students' reliance on mobile devices. Many students lack access to smartphones or the internet, leading to incomplete assignments, while others face distractions when using digital platforms. They added that younger students often lack the maturity to use devices solely for educational purposes. They mentioned that parental supervision is crucial to ensure productive screen time and prevent misuse. The need for awareness and parental involvement is emphasized to balance the benefits of technology with its risks. One teacher said,~~

> *“If we ask students to create content in groups, say we form a group of ten, usually only two out of the ten ends up doing the work. The remaining eight, instead of contributing, often get busy with unrelated activities on their phones or laptops, under the pretense of preparing content. This puts all the pressure on the two active members. As a result, those two, who are often the group leaders and active students across most subjects, bear the entire burden, leading to increased stress on them.~~.~~”* ***(IDI; Teacher; English medium school)***

Formatted: Left, Line spacing: Multiple 1.08 li

Formatted: Heading 3, Left, Line spacing: Double

Formatted: Space After: 0 pt, Line spacing: Double

Formatted: Font: 12 pt

Formatted: Quote, Left, Line spacing: Double

~~2.~~ Safety concerns and limited peer interaction~~Adolescents have become more dependent on devices owing to restrictions in outdoor activities and interaction~~

~~Additionally, p~~Parents expressed concerns about the safety of outdoor spaces, particularly for girls, which limited opportunities for children to interact with peers. Fear of misguidance by strangers, eve teasing, or sexual abuse led parents to restrict their children from going outside or interacting with lesser-known peers. Consequently, children had few friends and spent most of their leisure time on digital devices.~~arents reported that if children are restricted from using devices, they would try to go out of their homes, which would essentially not be safe and secured (particularly for the girls), with added fears of misguidance by strangers along with encountering eve teasing or, the worst, sexual abuse. Under these circumstances, parents mentioned that they do not allow their children to interact with lesser-known peers for fear of them going astray, which results in fewer friends and acquaintances. As a consequence, children do not discover any opportunities to play outdoor games; rather, they pass their leisure by engaging in devices only. One parent of a Bangla medium school said,~~

> *"Where will our children play in Dhaka city? As we live in apartment, sometimes there is rules or restrictions on going to the rooftop. We are primarily a home-centered family and do not allow our children to mix with others due to concerns about social safety. We do not even know our next-door neighbor due to our lack of time and security. I think there is no other reason than these."* ***(FGD; Parent; Bangla medium school)***

Limited family time and communication

~~Echoing the same voice, m~~Most parents reported that very few children had siblings to play with and that both working and non-working parents struggled to spend sufficient time with their

Formatted: Space After: 0 pt, Line spacing: Double

Formatted: Font: 12 pt

Formatted: Quote, Left, Line spacing: Double

Formatted: Font color: Text 1, Pattern: Clear

Formatted: Font color: Text 1, Pattern: Clear

Formatted: Font color: Text 1, Pattern: Clear

Formatted: Font color: Text 1, Pattern: Clear

Formatted: Space After: 0 pt, Line spacing: Double

children due to busy schedules. Some parents noted that children felt more comfortable communicating with classmates than with family members. ~~ost parents mentioned that very few children have siblings with whom they can play. Simultaneously, both working and non-working parents expressed regret about their inability to spend sufficient time with their children to manage their hectic schedules on a daily basis. In contrast, some parents revealed that children do not feel comfortable communicating with their parents compared to their comfort level with their classmates. One parent of a Bangla medium school added,~~

> *"~~As they do not have access to playing outdoor games,~~… sometimes we also cannot give enough time as her mom stays occupied and I also stay busy with school activities. What will she do? There is no access to play outside. She might spend her leisure time gossiping with others if we lived in a joint family, but as we live in a nuclear family, there is no scope to do that, either. That's why they are addicted to mobile phones. We also cannot say anything considering all of these issues."* ***(IDI; Parents; Bangla medium school)***

S~~Common to their parents, s~~tudents also highlighted similar issues about lack of interaction with their parents owing to their tedious schedules. One student conveyed,

> *"My father sat with me to spend time, but within two hours, he checked his E-mail numerous times. During that time, I also felt a similar urge to use my device as my father checked his inbox messages."* ***(FGD; Student; Bangla medium school)***

Formatted: Font: 12 pt

Formatted: Quote, Left, Line spacing: Double

Formatted: Space After: 0 pt, Line spacing: Double

Formatted: Quote, Left, Line spacing: Double

Formatted: Font: Not Bold, Italic

Formatted: Font: Not Bold, Italic

Formatted: Font: 12 pt, Not Bold, Font color: Text 1, Pattern: Clear

Scarcity of outdoor play and recreational opportunities

Both parents and students reported restricted access to outdoor play due to the lack of playgrounds and concerns over social insecurity, including kidnapping, eve teasing, or sexual abuse. In nuclear families, children lacked cousins or age-matched peers to interact with, leaving digital devices as the main alternative for leisure and recreation.

> *"In my home, it's just a nuclear family. Some families might have home support, but kids don't really listen to them, and they can't provide real company either. So, the child ends up spending that time mostly with electronic devices. Since both of us work, that's what keeps the child occupied."* ***(FGD; parents; English medium school)***

> "Even when cousins are together, they're all on their devices. Each cousin has one in hand. Instead of chatting, they just sit with their devices. We, on the other hand, can talk for hours after hours. But our kids can't. After talking for a little while, they'll say they need a break which means they want to check their phone and then *again*, they go back to the phone. Managing them is much tougher." ***(FGD; parents; English medium school)***

Additionally, they revealed that they did not have access to play outside due to the scarcity of playgrounds. Many students mentioned that they cannot go out even if there is a playground due to social insecurity concerns and risks such as kidnapping, eve teasing or sexual abuse. In addition, the nuclear family structure was such that there were no cousins of similar age to interact with. Hence, they said there were no alternatives for doing recreational activities other than using devices.

Formatted: Font color: Text 1

Formatted: Emphasis, Font: 11 pt, Font color: Text 1, Pattern: Clear

Formatted: Emphasis, Font: 12 pt

Formatted: Quote, Left, Line spacing: Double

Formatted: Emphasis, Font: 12 pt

Formatted: Emphasis, Font: 12 pt

Formatted: Emphasis, Font: 11 pt, Font color: Text 1, Pattern: Clear

Formatted: Line spacing: Double

~~3.~~ Unwanted exposure to age-inappropriate contents

Parents reported that children were frequently exposed to unwanted sexual content through advertisements on platforms such as TikTok, YouTube, Facebook, and online games. Many parents found it challenging to monitor children’s device use due to time constraints and discomfort discussing sexual topics, despite recognizing the importance of guidance during adolescence. Exposure to such content caused worry and anxiety, as parents feared it might trigger curiosity or inappropriate behaviors.~~Parents stated that unwanted advertisement was yet another obstacle to supervising or restricting the overuse of children’s devices as unwanted advertising of sexual content appeared on screen automatically when children viewed “TikTok”, “YouTube”, “Facebook” and online games. Parents referred to this as a principal challenge as they are unable to suffice and provide ample time to monitor their children. Consequently, they feel perturbed about viewing age-inappropriate videos. Moreover, some parents conveyed that they do not feel comfortable to converse with their children regarding sexual matters. If truth be told, they are not aware how to break the ice about certain physical changes of development in adolescence, even though they are well aware this is the crucial age to accustom them as their children will enter this phase of life. In addition, some parents shared their unpleasant experiences with children viewing inappropriate content, which, according to their perception, occurred due to undesired advertisements. They included that, after finding out their young ones are exposed to indecent content, negative thoughts frequently appeared in their minds; for instance, children might be curious towards sexual elements in the formative years of life. One parent of a Bangla medium school said,~~

> *“I was astonished after seeing what my son was viewing; when I asked my son about that obscene video, my son replied that he always plays games, but such*

Formatted: Heading 3, Left, Line spacing: Double, No bullets or numbering

Formatted: Font: Not Bold

Formatted: Space After: 0 pt, Line spacing: Double

Formatted: Font: 12 pt

Formatted: Quote, Left, Line spacing: Double

obscene videos sometimes appeared on his screen as advertisements. But after that incident, I can't stop thinking negatively about him." ***(FGD; Parents; Bangla medium school)***

Student's perspective on unwanted content

Students confirmed that sexual or inappropriate content sometimes appeared unintentionally while using digital platforms, and they were unable to skip certain advertisements. They reported that exposure increased curiosity and encouraged further exploration, underscoring the risks of unsupervised screen time.~~Similarly, children revealed that, sometimes, some undesirable advertisements of sexual content appear unintentionally on screens when they engage in social networks. They added that they couldn't skip the advertisements if they wanted to continue watching the content they were viewing. Some of them also notified that watching these unwanted contents further develops their inquisitiveness and provokes them to explore more.~~

> *"Sometimes a bad video pops up, and once you watch it, you feel like watching more. Later it causes physical problems… the body feels weak. I mean, in the middle of a video, like for 30 seconds, these kinds of clips appear. Actually, they're just 3-second videos that come up automatically. I was playing a game when those ads popped up, and my father saw it. After that, he didn't give me the phone for two or three days."* ***(FGD; Parents; English medium school)***

> *"After watching it, I keep thinking, 'Did I really watch that?' It keeps coming to my mind all the time, and it feels like mental pressure. So, I can't focus on anything else. And once I've seen it, I want to watch it again; if I don't, it doesn't feel good."* ***(FGD; Parents; English medium school)***

Formatted: Font color: Text 1
Formatted: Font color: Text 1
Formatted: Font color: Text 1
Formatted: Font color: Text 1
Formatted: Heading 4, Line spacing: Double
Formatted: Font: Not Bold, Font color: Text 1
Formatted: Font color: Text 1
Formatted: Quote, Left
Formatted: Font color: Text 1
Formatted: Font: Times New Roman, Font color: Text 1
Formatted: Font: Times New Roman, Font color: Text 1

Socioeconomic status and screen time

We identified a positive association between higher SES and higher possibility of the use of screen time among students.

> *"Families with higher income often provide multiple devices to their children, which makes it harder to control screen time and can affect their sleep and concentration."* ***(IDI; Parents; Bangla medium school)***

> *"Some of my friends don't have personal devices at home, so they play outside more, and I think they stay healthier than those who spend all day on phones or tablets."* *(**FGD; Student; Bangla medium school**)*

These quotes illustrate how SES shapes access to technology and, consequently, children's screen habits and associated health outcomes.

Teachers' perspective on supervision

Teachers emphasized the importance of parental supervision to ensure safe and productive use of digital devices. They noted that unsupervised access allowed students to engage with age-inappropriate content, which could negatively affect behavior, communication, and emotional development.~~Another point that needs to be emphasized in the context of age-appropriate content is inability to supervise their screen viewing. Parents added that children who use digital technology are not safe if they are left unsupervised and have too much screen time. One teacher at an English medium school said,~~

Formatted: Font: (Default) +Body (Calibri), 11 pt, Font color: Auto, Pattern: Clear

Formatted: Left, Line spacing: Multiple 1.08 li

Formatted: Font color: Text 1

Formatted: Heading 4, Left, Line spacing: Double

Formatted: Line spacing: Double

> *“You are leaving your children in the middle of the ocean as you permit them to use Google, YouTube, etc. So, you should view the content before permitting your children to view it. Parents should guide children so that the device used can be beneficial for them~~.~~.”* ***(~~K~~IDI; Teacher; English medium school)***

~~Similar findings were revealed from the discussion with students where most of the students discussed that children at their age should be kept under supervision because there are many dark websites of pornography and gambling where children at their age enter just out of inquisitiveness, unaware of the risks and harms of online use. They added that many children use abusive words without knowing the meaning of those words; some fall prey to unsafe relations as well as drug addiction and gambling. For these reasons, most of the students expressed the necessity of supervising children's digital device use. Students emphasized that children would be conscious if they knew that their activities on devices would be supervised or monitored by their parents, and then they would be discouraged from doing any wrong activities using those devices.~~

~~Similar findings revealed from the discussion of teachers whereas they reported that the lack of internet restrictions allows students to access various sites and learn things beyond their age, leading to unregulated usage. This is reflected in the classroom, where students use inappropriate, vulgar, or adult language—words that were unknown to previous generations at such a young age. Teachers often learn about these words for the first time from their students. One teacher said,~~

> *“Due to digital device usage, children are accessing inappropriate content and becoming addicted to it, with younger students watching adult material and mimicking adult behaviors. This leads to a lack of communication, with*

Formatted: Font color: Auto, Pattern: Clear
Formatted: Space After: 0 pt, Line spacing: Double
Formatted: Font: 12 pt
Formatted: Quote, Left, Line spacing: Double
Formatted: Space After: 0 pt, Line spacing: Double
Formatted: Font: 12 pt
Formatted: Line spacing: Double

interactions moving to platforms like Facebook. When breakups occur, they struggle to cope, often resulting in depression, as they are too young to handle such situations”. ((~~K~~IDI; Teacher; English medium school)

Students’ views on supervision

Students stressed that supervision could prevent access to unsafe websites, pornography, gambling, and the use of abusive language. They indicated that awareness of parental monitoring would discourage engagement in risky or inappropriate online behaviors.

> *“Children would be conscious if they knew that their activities on devices would be supervised or monitored by their parents, and then they would be discouraged from doing any wrong activities using those devices.”* ***(FGD; Student; English medium school)***

~~4.~~ Lack of parental digital literacy

Parents acknowledged that limited digital literacy constrained their ability to monitor children effectively. Children were often more technologically skilled and could bypass restrictions or age limits, leaving parents feeling unable to enforce boundaries. ~~Parents reported that the lack of digital literacy was a major challenge in monitoring their children. They mentioned that their children are more accustomed to using gadgets and devices than their parents are, as most of them are unaware of the proper operation of the devices. They added that children are well versed on internet safety strategies; hence, many parents do not set any age limit app by creating an E-mail account to restrict inappropriate content since their young ones can often figure out how to do away with such *technical* tricks. Moreover, some parents mentioned that they are not even habituated to these technical strategies, which they consider to be another huge challenge. One parent of an English medium school said,~~

Formatted: Font: 12 pt, Pattern: Clear (White)
Formatted: Font: 12 pt, Not Bold, Pattern: Clear (White)
Formatted: Justified, Space After: 0 pt, Line spacing: Double
Formatted: Font: 12 pt, Pattern: Clear (White)
Formatted: Quote, Left, Line spacing: Double
Formatted: Font: 12 pt, Bold, Pattern: Clear (White)
Formatted: Font: 12 pt, Pattern: Clear (White)
Formatted: Font: 12 pt
Formatted: Heading 3, Left, Line spacing: Double, No bullets or numbering
Formatted: Space After: 0 pt, Line spacing: Double

> *"He is expert on device use than me. He is an expert in IT. He can hack our software to open it, that's why I left to use age limit apps."* ***(FGD; Parent; English medium school)***

Students also reported creating fake accounts to access games or social media apps restricted by age settings. ~~An interesting finding was revealed from the discussion of students, who mentioned that students of their age make *fake accounts* in order to access certain video games and social media apps. One student in this context reported,~~

> *"There are many children who lie by increasing their age using fake profile accounts."* ***(FGD; Parent; English medium school)***

Recommendations from study participants to reduce excessive screen time~~:~~

~~1. **Initiatives to be taken by schools**~~

- ~~Participants, including parents and students, emphasized the significance of school efforts to implement steps to supervise and lower device usage. Most parents suggested replacing online activities in the teaching curriculum with innovative offline activities.~~
- ~~They also proposed that students should complete their device-related tasks at school under the supervision of teachers rather than bringing them home. In this context, parents advocated for school authorities to provide the required devices for such purposes.~~
- ~~Both parents and students drew attention to the need to raise awareness from the school's end regarding the harmful effects of excessive device use on children. They urged the implementation of collaborative programs involving parents, students, and teachers on school premises to pursue the objective of reducing screen use this objective in a holistic manner.~~

Formatted: Font: 12 pt
Formatted: Quote, Left, Line spacing: Double
Formatted: Space After: 0 pt, Line spacing: Double
Formatted: Font: 12 pt
Formatted: Quote, Left, Line spacing: Double
Formatted: No underline
Formatted: No underline
Formatted: Space After: 0 pt, Line spacing: Double
Formatted: Font: (Default) Times New Roman, 12 pt, Bold
Formatted: Normal, Line spacing: Double, No bullets or numbering
Formatted: Font: (Default) Times New Roman, 12 pt
Formatted: Space After: 0 pt, Line spacing: Double

- Students and parents also discussed the value of increasing extracurricular activities at school. Some parents have suggested allocating specific times regularly for children to be involved in physical activities and innovative games. They believe that this approach will help divert their attention from using devices.
- Teachers and parents have accentuated recruiting a mentor, specifically a psychologist, within the school. This mentor would particularly address concerns regarding excessive device use among children and offer guidelines to overcome addiction along with other psychological support.
- Meanwhile, teachers highlighted using all devices for academic purposes under the supervision of teachers at school premises because using digital devices in education is indispensable in today's world. Subjects like ICT and computer studies are impossible to comprehend without the aid of digital devices.

2. Alternative options instead of screen viewing

All participants, including parents, teachers, and students, recommended offering other choices instead of screen viewing. Preferable activities like playing outdoor games, indulging in creative arts and crafts, engaging in an exciting book, or participating in sports or hobbies they would enjoy, thus providing a range of options that would enrapture their attention beyond the screen.

- Students suggested reducing screen time by removing the barriers and enhancing accessibility for outdoor playing and activities in the surrounding neighborhood.

3. Parents should spend quality time with their children

All participants stressed the significance of parents spending quality time with their children to reduce screen time. They should prioritize quality time together, offering a practical approach to managing screen time and nurturing healthy relationships within the family.

Formatted: Font: (Default) Times New Roman, 12 pt, Bold

Formatted: Normal, Line spacing: Double, No bullets or numbering

Formatted: Font: (Default) Times New Roman, 12 pt

Formatted: Justified, Space After: 0 pt, Line spacing: Double

Formatted: Font: (Default) Times New Roman, 12 pt

Formatted: Normal, Left, Line spacing: Double, No bullets or numbering

Formatted: Justified, Space After: 0 pt, Line spacing: Double

- Students said that parents can actively engage in meaningful interactions, such as conversations and combined activities like morning walks, picnics or outings, interactive games, cooking something together and so on, all of which would strengthen the parent-child bonding, culminating in decreased screen time.

4. **To develop self-regulation in children**

- Most students and parents refer to the importance of building self-regulation to reduce the excessive use of digital devices. They believe that learning to refrain from prolonged use of screens is more effective than enforcing strict rules and regulations by parents.

5. **Ensuring social security**

- Parents recommended engaging children in outdoor activities to reduce screen time. They emphasized the importance of creating safe and supervised environments. They believed that promoting outdoor play reduces screen time and offers the benefits of physical exercise, social interaction and the development of essential life skills and resilience.

6. **Policies to be implemented on the national level**

Individual and Family Level

- Parents should spend quality time with their children: Parents should engage in meaningful interactions such as conversations, morning walks, picnics, interactive games, or cooking together to strengthen parent–child bonding and naturally reduce reliance on digital devices.
- Developing self-regulation in children: Encourage children to manage their own screen habits and refrain from prolonged use, emphasizing self-discipline over strict parental rules.

> *"The time that is being spent on the mobile means they are not getting enough rest. Of course, that has an effect. That's why I think counseling is necessary. Because through counseling, whether it's about mobile use or something else,*

Formatted: Font: (Default) Times New Roman, 12 pt

Formatted: Normal, Left, Line spacing: Double, No bullets or numbering

Formatted: Font: (Default) Times New Roman, 12 pt, Bold

Formatted: Normal, Line spacing: Double, No bullets or numbering

Formatted: Font: (Default) Times New Roman, 12 pt

Formatted: Font: (Default) Times New Roman, 12 pt, Bold

Formatted: Font: (Default) Times New Roman, 12 pt

Formatted: Font: (Default) Times New Roman, 12 pt, Bold

the issue will come up. They will hear things like, 'Don't do this,' or, 'It's better if you avoid that,' and then the topic of studies will also come up. One way or another, some advice will be given." ***(FGD_Parents_ACADEMIA)***

- Alternative options instead of screen viewing: Promote hobbies like outdoor games, creative arts and crafts, reading, sports, and other enjoyable activities to capture children's attention beyond digital devices.

"I have the same opinion as the others. One more recommendation I would make is that, since study time is spent on mobile devices, children then look for suggestions on how to excel in exams in a short amount of time. Because of this, they become more concerned with just performing well in exams rather than actually learning. That's a major harm. Another point is that devices will be used anyway, so along with the children, the parents also need to be made aware. If we overreact, then whatever is forbidden is exactly what they will want to do. In that case, if parents behave logically, it will help, because sometimes their overreaction can have a negative impact. That's why it would be good to have a counseling session for parents as well, on how to guide their children. At the school level, too, parents could be made more aware of how to behave with their children." ***(FGD_Parents_ACADEMIA)***

Institutional and Community Level

- Initiatives to be taken by schools:
    - Replace online activities in the curriculum with offline approaches.

- Ensure device-related tasks are completed under teacher supervision, with schools providing necessary devices.
- Raise awareness about the harmful effects of excessive screen use through collaborative programs involving parents, teachers, and students.
- Increase extracurricular opportunities, such as physical activities and creative games, to divert children's attention from devices.
- Appoint a school-based mentor, preferably a psychologist, to address device overuse concerns and provide psychological support.
- Limit digital tools like ICT and computer studies to supervised use within school premises.

- Alternative options instead of screen viewing (institutional support): Reduce barriers and improve access to outdoor spaces and neighborhood activities to encourage participation in non-screen pursuits.

> *"Every area needs to have a playground. There should be guards around the field and cameras installed along the area's borders."* ***(FGD_Baridhara_students)***

- Ensuring social security: Create safe and supervised environments for outdoor activities, encouraging physical exercise, social interaction, and development of life skills while reducing screen time.

> *"Schools could arrange programs to make parents aware of how harmful mobile phones can be. Such initiatives could also be taken through different NGOs. These programs could highlight the mental health problems associated with mobile phone use and explain them sincerely to parents. Various awareness sessions could also be conducted."* ***(B2_FGD_Students_ DAM)***

Policies to be implemented on the national level

- Parents proposed a range of government-led initiatives to reduce children's screen viewing and promote safer digital platforms. They suggested developing digital restriction mechanisms in collaboration with the ICT Department of Bangladesh to prevent exposure to age-inappropriate content, using content or web filtering tools to block harmful websites and online categories. Additionally, parents recommended creating specialized software or applications offering age-appropriate content while enabling parental monitoring, combining safety with culturally relevant oversight.
- Government-driven awareness campaigns were also emphasized, utilizing widely accessed platforms such as television and social media to inform parents and children about the risks of excessive screen time. Integrating discussions of digital risks into formal education was another key recommendation, including dedicated chapters in ICT textbooks addressing the negative impacts of device overuse. Teachers further suggested incorporating age-specific screen time guidelines in textbooks, formalizing limits such as restricting second-grade students to one hour of screen use per day in line with regulatory recommendations.

> *"This has actually become a national issue now since almost all children are facing this problem. If the government includes these issues in the curriculum, it carries more importance than just us parents saying it. This could be done through NGOs or schools as well. The media we have could also be used to raise awareness. For example, we were taught many things through the Meena cartoon. In the same way, cartoons could be made to show the negative sides of mobile use, so that children themselves would be attracted to watch and learn."*
>
> ***(FGD_Parents_ACADEMIA)***

Overall, these recommendations reflect a collective call from parents and teachers for national - level policies that combine regulation, education, awareness, and technology-based solutions to address the growing challenges of children’s screen use.

~~Parents have proposed numerous ideas for the government to implement in order to reduce children's screen viewing and create safer digital platforms. They suggested developing digital restriction mechanisms with the help of the ICT (Information & Communication Technology) Department of Bangladesh to avoid age-inappropriate content such as drug abuse or indecent videos, which can prove to be harmful to them.~~

- ~~Based on their views, content filtering or web filtering means using software or hardware to block access to specific websites or online content categories based on predefined criteria. They added that by such filtering, children can be prevented from accessing detrimental or undesirable materials while browsing the internet.~~
- ~~Some parents have suggested the development of specialized software or applications that offer children-friendly content with specific age categories, ensuring that they are exposed to age-appropriate materials while using digital devices. Additionally, parents could utilize these apps to monitor their children's screen time activities directly, ensuring their children indulged in content that aligned with their respective cultural values and traditions. This approach combines content filtering with parental monitoring features to create a safer and more integrated digital environment for children.~~

Formatted: Space After: 0 pt, Line spacing: Double

Formatted: Space After: 0 pt, Line spacing: Double, No bullets or numbering

Formatted: Space After: 0 pt, Line spacing: Double

- Additionally, parents highlighted that the government should utilize various media platforms, such as TV channels and social media, where children spend most of their time, to raise awareness about the deleterious effects of excessive digital device use. They said that advertising campaigns can effectively reach children and parents, alerting them about the potential risks associated with prolonged screen time.
- Some parents stress on including a chapter in every year's ICT textbook, specifically focusing on the negative impacts of excessive screen viewing. They argue that while the subject teaches students how to use digital devices and familiarizes them with various features, it often neglects to address the potential negative consequences of unrestrained usage.
- Teachers also emphasize governmental interventions to formalize rules and regulations for children's digital device usage. For instance, a suggestion has been made that the screen time duration should be mentioned in their textbooks according to age, such as children in the second grade being limited to one hour of screen time per day as per recommendations of regulatory bodies.

Discussion

This study explored the challenges parents face in monitoring their children’s screen use in the context of the evolving educational and social environment in urban Bangladesh. Parents and students highlighted that the new education system encourages independent learning and reliance on digital platforms. Many parents reported that teachers provide insufficient guidance in classrooms, pushing students to seek help online. While digital devices facilitate learning, parents

observed frequent misuse, such as playing games under the guise of studying. Students' dependence on online resources and group assignments via social media further exacerbated the challenge, as distractions from short videos, reels, and other entertainment content were commonly reported. Studies found that the barriers and facilitators of physical activity among schoolchildren identified children preferring sedentary activities such as video games, influence of older siblings, lack of family mediation and deficit of sufficient infrastructure as the main findings [31]. Teachers noted that younger students often lack the maturity to use devices responsibly, emphasizing the importance of parental supervision to ensure productive and safe digital learning.

The data on this paper demonstrates the challenges faced by parents to regulate screen viewing as well as suggestions with the means to reduce use of digital media and gadgets. To begin with, devices nowadays are an indispensable source of learning and acquiring knowledge. Our data revealed that, parents believed SV can be a useful educational tool, showing consent to previous research conducted by Solomon Moore et al [2]. The study highlighted identified several four main points in the challenges section, , one of which was being the "positive aspects of SV", where parents found restricting SV could turn into an obstacle for them in terms of their technological know-hows. Another domain, was "not all screens are equal" where the parents actively encouraged educational SV mentioning their children enjoyed watching documentaries and researching topics on the internet. The greater challenge would appear when children used these devices mentioning they were indulged in academics however operating it for non-academic purposes such as gaming and recreation. These findings suggest considerable scope for future research to further explore the nuanced impacts of different types of screen use. re is immense scope for future research to implore this concept.

Formatted: Not Highlight

Formatted: Highlight

Parents' concerns about social safety, particularly for girls, restricted children's outdoor activities and peer interactions.A common challenge reported by parents was their inability to provide outdoor access to children. These issues were raised throughout the current study as safety was a major barrier for outdoor activities. A qualitative case study done on Spanish immigrant children highlighted similar findings [32]. Fear of eve teasing, sexual abuse, or misguidance led parents to limit their children's opportunities for outdoor activities and physical play. In nuclear families, the scarcity of siblings or cousins further reduced opportunities for interaction, leaving digital devices as the main avenue for leisure. Limited family time and communication gaps were also evident, as parents' busy schedules hindered meaningful engagement with their children. Children reported feeling more comfortable interacting with peers than with parents, a phenomenon that may contribute to increased screen dependency. Factors such as importance of physical activities, gender disparities, academic load, scarcity of social acquaintances, familial socio-economic limitations, absence of proper surroundings, time constraints and feelings of insecurity were the key-points which were exposed in the paper. Owing to rising crimes and chances for children being kidnapped or sexually harassed, the challenge was inevitable. Another qualitative research on barriers and facilitators of physical activity among schoolchildren identified children preferring sedentary activities such as video games, influence of older siblings, lack of family mediation and deficit of sufficient infrastructure as the main findings [32]. These findings align with previous research indicating that urban children often face constrained outdoor play due to safety concerns and that parental availability plays a critical role in managing screen time [33].

Formatted: Font: (Default) Times New Roman, 12 pt

It was elicited that, lack of spaces such as courtyards and terraces (particularly those who lived in smaller homes), parks or gardens were a major hurdle for active play.

Parents and teachers expressed concern about children's exposure to inappropriate content, often appearing through advertisements on popular digital platforms. ~~According to our findings, unwanted or inappropriate content was a significant barrier that parents wanted to avoid.~~

Formatted: Font: (Default) Times New Roman, 12 pt

Previous quantitative research suggests that parent-child relationship, socio-economic conditions, parents being unaware of children's SV and children owning devices by themselves were major contributive elements for viewing violent or pornographic content [34]. Many parents reported feeling unprepared to discuss sexual development, which compounded the challenges of supervision. Students confirmed that exposure to such content often increased curiosity and encouraged further exploration, reinforcing the risks of unsupervised digital use. ~~Such exposure to adult content at the wrong time implicate multiple risks and can lead to harmful outcomes. There could be increased curiosity about these elements resulting in lack of attention to academic contents and deviating to watching more adult videos which could have a deleterious impact on the future of the children. In this case, the familial understanding is supposed to play a vital role.~~ Relevant qualitative paper explored the concerns of parents to exposure to harmful content [21]. Teachers highlighted that unsupervised access could lead to behavioral and emotional consequences, including imitation of adult behaviors, reduced communication skills, and vulnerability to online risks. Students emphasized that awareness of parental monitoring could discourage engagement in unsafe or inappropriate activities.

A significant barrier to effective supervision was parents' limited ~~The elements they thought were important in case of girl child were impacts of social media, cyberbullying, grooming and early sexualization; whereas addictive behaviour, gaming and pornography viewing for boys.~~

~~Our findings demonstrate that a lack of~~ digital literacy ~~was a significant factor.~~.

The home technology environment is imperative in this context. Various studies report that children of this generation are exposed to a wide range of toys, multimedia, devices and gadgets from a very early age which enable and enhance them to efficiently operate them [35,36]. This, iIn contrast, is not the scenario for the parents of this generation. many parents reported being less technologically skilled than their children, who could bypass restrictions or manipulate age verification systems. Students also reported creating fake accounts to access games or social media apps restricted by age. These results reflect global evidence showing that parents' digital skills are critical for establishing boundaries and guiding safe online behaviors [37].

Their attitudes, preferences and concerns are different owing to lack of digital knowledge in them. Digital literacy is an essential skill of late. A paper by Romero mentioned, there are 4 subsets of skills for parents namely basic digital parenting skills, communication skills, creativity and life-long learning skills [34]. Digital knowledge imparts an understanding of online safety [24]. In addition, they need to adapt to the changing times which are engulfed in the digital environment. Lack of digital literacy leads to familial disputes, impacting the peace and harmony of the home; on the contrary, learning these skills can protect the children and strengthen family bonds. Only when parents are capable to use such devices with ease, they can take charge of children's screen viewing duration and most importantly, screen content.

Furthermore, this study focused on some recommendations which the caregivers suggested in order to reduce SV in the young ones. They mentioned that schools should take some core initiatives. A cluster-randomized controlled trial to evaluate the effectiveness of school-based intervention on screen time in Bangladesh was done between two groups reported that, there were promising changes in children when physical activity was encouraged and screen time was reduced [28].

Points such as implementation of national guidelines, offering alternatives to screen viewing, increased family time, development of self-regulation in children and ensuring safer environment were also discussed extensively. Relevant qualitative exploration enumerated some solutions for the government to imply such as monitor inappropriate programs for the young ones and block them; use social media to explain the detrimental impacts of overuse of devices; establish more sports clubs; ensure computer classes at schools which would include lessons regarding optimum SV [17]. Another paper mentioned that, strong bonding between children and parents impacts the SV of children in a beneficial manner [20]. Furthermore, similar research recommended that parents need to enhance their knowledge about social media and associated risks to protect their children [38]. In addition, a systematic review revealed that, weaker parent-child bonding and unhealthy familial environment was linked to gaming addiction in children [39]. Also, single-parent families were detected to have greater ~~amount of internet~~internet abuse in the young ones. It is essential to ~~take into account~~consider the deleterious impacts of excessive SV on children. The implications of this paper are based on the challenges encountered by the caregivers and their thoughtful proposals to mitigate this condition The balance between the real and virtual world is difficult to maintain, but it is mandatory. While it is necessary to maintain the guidelines of children's SV flexible in order to go with the flow of the rapidly ~~-~~changing technological ambience, certain guidelines need to be implemented to make sure caregivers understand how much SV is excessive. It can be encouraged to develop specific policies and programs on multiple levels to eliminate the difficulties faced with regards to monitoring SV and promote alternative activities and options to foster a healthier life for ~~the~~ children.

~~**Limitations**~~

~~This study has a number of limitations. There were only three KIIs with teachers, and two of the respondents were men and one was a woman. This could have influenced the results in terms of viewpoints on the topics covered in the interviews. If we had more time, we could have expanded the sample sizes and added focus groups with mothers and fathers separately, as well as with boys and girls, to triangulate the findings from the KIIs and IDIs. The urban setting of Dhaka, Bangladesh, where we conducted this study, may have affected the findings and made them different from those of other rural areas of the country. Lastly, such qualitative research in a particular region with a small sample size is not representative of the entire population.~~

Conclusion

Urban digitalization has its own set of drawbacks. The hurdles caregivers face in regulating screen use of children highlight the contribution of devices for academic purposes and their digital illiteracy. The inability to ensure safety for outdoor activities is a major drawback. In addition, ensuring age-appropriate content for children turns out to be a matter of concern.

Formatted: Line spacing: Double

It is essential to implement policy guidelines that emphasize the active involvement of schools, complemented by national-level strategies. Furthermore, a warm home environment and safe and suitable infrastructure are compulsory.

Formatted: Space After: 0 pt, Line spacing: Double

Acknowledgment

Formatted: Line spacing: Double

The author would like to acknowledge their affiliation, with the icddr, b and its core donors who provide unrestricted support to icddr,b for its operations and research, including the Governments of Bangladesh and Canada. We gratefully acknowledge our core donors for their support and commitment to icddr,b's research efforts. The authors are also grateful to the school authorities for their unconditional support.

Data availability

The data sets generated or analyzed during this study are available from the corresponding author upon reasonable request.

Authors' contributions

SHK and NS contributed to conceptualizing, analyzing, writing, revising, and finalizing the manuscript with the support of BKS, TRS, RH, and FT. All the authors have read, revised, and approved the final version of the manuscript.

Conflict of interests

None declared.

~~Funding statement~~

~~The current study was financially supported by the Bangladesh government's Mujib100 Research Grant for Women award, in collaboration with icddr, b, grant no. 1945.~~

References~~s~~

Formatted: No Spacing, Left, Line spacing: single

Formatted: Space After: 0 pt, Line spacing: Double

1. Ashton JJ, Beattie RM. Screen time in children and adolescents: is there evidence to guide parents and policy? Lancet Child Adolesc Heal. 2019;3: 292–294. doi:10.1016/S2352-4642(19)30062-8
2. Solomon-Moore E, Matthews J, Reid T, Toumpakari Z, Sebire SJ, Thompson JL, et al. Examining the challenges posed to parents by the contemporary screen environments of children: a qualitative investigation. BMC Pediatr. 2018;18: 129. doi:10.1186/s12887-

018-1106-y

3. Wilkinson C, Low F, Gluckman P. Screen time: The effects on children's emotional, social, and cognitive development. Univ Auckl Cent Inf Futur. 2021; 1–11.
4. Lareau A. Unequal Childhoods: Class, Race, and Family Life. 2018 pp. 444–451. Available: https://www.taylorfrancis.com/chapters/edit/10.4324/9780429499821-75/unequal-childhoods-class-race-family-life-annette-lareau
5. Mollborn S, Limburg A, Pace J, Fomby P. Family Socioeconomic Status and Children's Screen Time. J Marriage Fam. 2022;84: 1129–1151. doi:10.1111/jomf.12834
6. Paulich KN, Ross JM, Lessem JM, Hewitt JK. Screen time and early adolescent mental health, academic, and social outcomes in 9- and 10- year old children: Utilizing the Adolescent Brain Cognitive Development SM (ABCD) Study. PLoS One. 2021;16: e0256591. doi:10.1371/journal.pone.0256591
7. Nagata JM, Al-Shoaibi AAA, Leong AW, Zamora G, Testa A, Ganson KT, et al. Screen time and mental health: a prospective analysis of the Adolescent Brain Cognitive Development (ABCD) Study. BMC Public Health. 2024;24: 2686. doi:10.1186/s12889-024-20102-x
8. Tsang SMH, Cheing GLY, Lam AKC, Siu AMH, Pang PCK, Yip K-C, et al. Excessive use of electronic devices among children and adolescents is associated with musculoskeletal symptoms, visual symptoms, psychosocial health, and quality of life: a cross-sectional study. Front public Heal. 2023;11: 1178769. doi:10.3389/fpubh.2023.1178769
9. Khan A, Burton NW. Screen-Based Behaviors of Adolescents in Bangladesh. J Phys Act Health. 2016;13: 1156–1163. doi:10.1123/jpah.2015-0514

10. Reid Chassiakos YL, Radesky J, Christakis D, Moreno MA, Cross C. Children and Adolescents and Digital Media. Pediatrics. 2016;138. doi:10.1542/peds.2016-2593

11. Livingstone S BJ. Parenting in the digital age: The challenges of parental responsibility in comparative perspective. diva-portal.org. 2018. Available: https://scholar.google.com/scholar?q=Parenting+in+the+digital+age:+The+challenges+of+parental+responsibility+in+comparative+perspective&hl=en&as_sdt=0&as_vis=1&oi=scholart

12. Blum-Ross A, Livingstone S. “Sharenting,” parent blogging, and the boundaries of the digital self. Pop Commun. 2017;15: 110–125. doi:10.1080/15405702.2016.1223300

13. Carson V, Clark M, Berry T, Holt NL, Latimer-Cheung AE. A qualitative examination of the perceptions of parents on the Canadian Sedentary Behaviour Guidelines for the early years. Int J Behav Nutr Phys Act. 2014;11: 65. doi:10.1186/1479-5868-11-65

14. Martínez-Andrés M, Bartolomé-Gutiérrez R, Rodríguez-Martín B, Pardo-Guijarro MJ, Garrido-Miguel M, Martínez-Vizcaíno V. Barriers and Facilitators to Leisure Physical Activity in Children: A Qualitative Approach Using the Socio-Ecological Model. Int J Environ Res Public Health. 2020;17. doi:10.3390/ijerph17093033

15. Geurts SM, Koning IM, Vossen H, Van den Eijnden RJJM. A Qualitative Study on Children’s Digital Media Use and Parents’ Self-interest. J Child Fam Stud. 2022;31: 2015–2026. doi:10.1007/s10826-021-02074-3

16. Gupta P, Shah D, Bedi N, Galagali P, Dalwai S, Agrawal S, et al. Indian Academy of Pediatrics Guidelines on Screen Time and Digital Wellness in Infants, Children and Adolescents. Indian Pediatr. 2022;59: 235–244.

17. Buabbas A, Hasan H, Shehab AA. Parents’ Attitudes Toward School Students’ Overuse of

Smartphones and Its Detrimental Health Impacts: Qualitative Study. JMIR Pediatr Parent. 2021;4: e24196. doi:10.2196/24196

18. Throuvala MA, Griffiths MD, Rennoldson M, Kuss DJ. Policy recommendations for preventing problematic internet use in schools: A qualitative study of parental perspectives. Int J Environ Res Public Health. 2021;18. doi:10.3390/ijerph18094522

19. Sharp CA. Qualitative Research and Evaluation Methods (3rd ed.). Evaluation Journal of Australasia. 2003. pp. 60–61. doi:10.1177/1035719X0300300213

20. Nwankwo F, Shin HD, Al-Habaibeh A, Massoud H. Evaluation of Children's Screen Viewing Time and Parental Role in Household Context. Glob Pediatr Heal. 2019;6: 2333794X19878062. doi:10.1177/2333794X19878062

21. Marsh S, Chu JTW, Calder AJ. "I tried to take my phone off my daughter, and i got hit in the face": a qualitative study of parents' challenges with adolescents' screen use and a toolbox of their tips. BMC Public Health. 2024;24: 217. doi:10.1186/s12889-024-17690-z

22. Muppalla SK, Vuppalapati S, Reddy Pulliahgaru A, Sreenivasulu H. Effects of Excessive Screen Time on Child Development: An Updated Review and Strategies for Management. Cureus. 2023;15: e40608. doi:10.7759/cureus.40608

23. Raj D, Zulkefli NABM, Minhat HS, Ahmad N. Parental Intervention Strategies to Reduce Screen Time Among Preschool-aged Children: A Systematic Review. Malaysian J Med Heal Sci. 2022;18: 295–304. doi:10.47836/mjmhs18.6.38

24. Sharma H, Ahuja S. Mitigating factors of screen time on children: a systematic review. Int J Contemp Pediatr. 2024;11: 1637–1643. doi:10.18203/2349-3291.ijcp20243092

25. Qi J, Yan Y, Yin H. Screen time among school-aged children of aged 6–14: a systematic review. Glob Heal Res Policy. 2023;8: 12. doi:10.1186/s41256-023-00297-z

26. Choi EJ, King GKC, Duerden EG. Screen time in children and youth during the pandemic: A systematic review and meta-analysis. Glob Pediatr. 2023;6: 100080. doi:https://doi.org/10.1016/j.gpeds.2023.100080

27. Sulistyo PB. Digital Literacy Competence of Parents in Supervising Their Children Using Digital Media. Int J Soc Sci Hum Res. 2022;05: 636–642. doi:10.47191/ijsshr/v5-i2-32

28. Ahmed KR, Kolbe-Alexander T, Khan A. Effectiveness of a school-based intervention on physical activity and screen time among adolescents. J Sci Med Sport. 2022;25: 242–248. doi:10.1016/j.jsams.2021.10.007

29. Braun V, Clarke V. Using thematic analysis in psychology. Qual Res Psychol. 2006;3: 77–101. doi:10.1191/1478088706qp063oa

30. Nowell LS, Norris JM, White DE, Moules NJ. Thematic Analysis: Striving to Meet the Trustworthiness Criteria. Int J Qual Methods. 2017;16: 1–13. doi:10.1177/1609406917733847

31. Alcántara-Porcuna V, Sánchez-López M, Martínez-Vizcaíno V, Martínez-Andrés M, Ruiz-Hermosa A, Rodríguez-Martín B. Parents' Perceptions on Barriers and Facilitators of Physical Activity among Schoolchildren: A Qualitative Study. Int J Environ Res Public Health. 2021;18. doi:10.3390/ijerph18063086

32. Marconnot R, Marín-Rojas AL, Delfa-de-la-Morena JM, Pérez-Corrales J, Gueita-Rodríguez J, Fernández-de-Las-Peñas C, et al. Recognition of Barriers to Physical Activity Promotion in Immigrant Children in Spain: A Qualitative Case Study. Int J Environ Res Public Health. 2019;16. doi:10.3390/ijerph16030431

33. Charan Gopal Singh, Kalia Raman, Khurana Mandeep Singh, Narang Gursharan Singh. From Screens to Sunshine: Rescuing Children's Outdoor Playtime in the Digital Era. J

Indian Assoc Child Adolesc Ment Heal. 2024;20: 11–17. doi:10.1177/09731342241229845
34. Liu S, Chang F-C, Chiu C-H, Li F, Chen P-H, Chen C-Y, et al. Parent-Child Discrepancies in Reports of Exposure to Violence/Pornography on Mobile Devices and the Impact on Children's Psychosocial Adjustment. Cyberpsychol Behav Soc Netw. 2021;24: 390–398. doi:10.1089/cyber.2020.0120
35. Romero M. Digital literacy for parents of the 21st century children. 2014; 32–40.
36. Terras MM, Ramsay J. Family Digital Literacy Practices and Children's Mobile Phone Use. Front Psychol. 2016;7: 1957. doi:10.3389/fpsyg.2016.01957
37. Teke B, Ayar D. The Impact of Parents' Digital Awareness on their Digital Attitude Behaviors. Compr Child Adolesc Nurs. 2025;48: 139–151. doi:10.1080/24694193.2025.2511111
38. El G, Moawad NA, Gad G, Ebrahem S. The Relationship between Use of Technology and Parent-Adolescents Social Relationship. J Educ Pract. 2016;7: 168–178. Available: www.iiste.org
39. Schneider LA, King DL, Delfabbro PH. Family factors in adolescent problematic Internet gaming: A systematic review. J Behav Addict. 2017;6: 321–333. doi:10.1556/2006.6.2017.035

1. Ashton JJ, Beattie RM. Screen time in children and adolescents: is there evidence to guide parents and policy? The Lancet Child & Adolescent Health. 2019 May 1;3(5):292-4.

Formatted: Strikethrough

2. Solomon-Moore E, Matthews J, Reid T, Toumpakari Z, Sebire SJ, Thompson JL, et al. Examining the challenges posed to parents by the contemporary screen environments of children: a qualitative investigation. BMC pediatrics. 2018 Dec; 18:1-2.

3. Wilkinson C, Low F, Gluckman P. Screen time: The effects on children's emotional, social, and cognitive development.

4. Lareau A. Unequal childhoods: Class, race, and family life. Inequality in the 21st Century 2018 May 15 (pp. 444-451). Routledge.

5. Mollborn S, Limburg A, Pace J, Fomby P. Family socioeconomic status and children's screen time. Journal of Marriage and Family. 2022 Aug;84(4):1129-51.

6. Paulich KN, Ross JM, Lessem JM, Hewitt JK. Screen time and early adolescent mental health, academic, and social outcomes in 9-and 10-year old children: Utilizing the Adolescent Brain Cognitive Development℠(ABCD) Study. PloS one. 2021 Sep 8;16(9):e0256591.

7. Nagata JM, Al-Shoaibi AA, Leong AW, Zamora G, Testa A, Ganson KT, Baker FC. Screen time and mental health: a prospective analysis of the Adolescent Brain Cognitive Development (ABCD) Study. BMC Public Health. 2024 Oct 7;24(1):2686.

8. Tsang SM, Cheing GL, Lam AK, Siu AM, Pang PC, Yip KC, et al. Excessive use of electronic devices among children and adolescents is associated with musculoskeletal symptoms, visual symptoms, psychosocial health, and quality of life: a cross-sectional study. Frontiers in Public Health. 2023 Jun 29;11:1178769.

9. Khan A, Burton NW. Screen-based behaviors of adolescents in Bangladesh. Journal of Physical Activity and Health. 2016 Nov 1;13(11):1156-63.

10. Reid Chassiakos YL, Radesky J, Christakis D, Moreno MA, Cross C, Hill D, Ameenuddin N, Hutchinson J, Levine A, Boyd R, Mendelson R. Children and adolescents and digital media. Pediatrics. 2016 Nov 1;138(5).

11. Livingstone S, Byrne J. Parenting in the digital age: The challenges of parental responsibility in comparative perspective.

12. Blum-Ross A, Livingstone S. "Sharenting," parent blogging, and the boundaries of the digital self. InSelf-(re) presentation now 2020 May 18 (pp. 70-85). Routledge.

13. Carson V, Clark M, Berry T, Holt NL, Latimer-Cheung AE. A qualitative examination of the perceptions of parents on the Canadian Sedentary Behaviour Guidelines for the early years. International Journal of Behavioral Nutrition and Physical Activity. 2014 Dec;11:1-8.

14. Martínez-Andrés M, Bartolomé-Gutiérrez R, Rodríguez-Martín B, Pardo-Guijarro MJ, Garrido-Miguel M, Martínez-Vizcaíno V. Barriers and facilitators to leisure physical activity in children: a qualitative approach using the socio-ecological model. International journal of environmental research and public health. 2020 May;17(9):3033.

15. Geurts SM, Koning IM, Vossen H, Van den Eijnden RJ. A qualitative study on children's digital media use and parents' self-interest. Journal of Child and Family Studies. 2022 Jul 1:1-2.

16. Gupta P, Shah D, Bedi N, Galagali P, Dalwai S, Agrawal S, John JJ, Mahajan V, Meena P, Mittal HG, Narmada S. Indian Academy of Pediatrics Guidelines on screen time and digital wellness in infants, children and adolescents. Indian pediatrics. 2022 Mar;59(3):235-44.

17. Buabbas A, Hasan H, Shehab AA. Parents' attitudes toward school students' overuse of smartphones and its detrimental health impacts: Qualitative study. JMIR Pediatrics and Parenting. 2021 May 20;4(2):e24196.

18. Throuvala MA, Griffiths MD, Rennoldson M, Kuss DJ. Policy recommendations for preventing problematic internet use in schools: a qualitative study of parental perspectives. International journal of environmental research and public health. 2021 Apr 24;18(9):4522.

19. Guest G, Namey E, Chen M. A simple method to assess and report thematic saturation in qualitative research. PloS one. 2020 May 5;15(5):e0232076.

20. Marconnot R, Marín-Rojas AL, Delfa-de-la-Morena JM, Pérez-Corrales J, Gueita-Rodríguez J, Fernández-de-Las-Peñas C, et al. Recognition of barriers to physical activity promotion in immigrant children in Spain: a qualitative case study. International journal of environmental research and public health. 2019 Feb;16(3):431.

21. Alcántara-Porcuna V, Sánchez-López M, Martínez-Vizcaíno V, Martínez-Andrés M, Ruiz-Hermosa A, Rodríguez-Martín B. Parents' perceptions on barriers and facilitators of physical activity among schoolchildren: A qualitative study. International Journal of Environmental Research and Public Health. 2021 Mar 17;18(6):3086.

22. Liu S, Chang FC, Chiu CH, Li F, Chen PH, Chen CY, et al. Parent–Child Discrepancies in Reports of Exposure to Violence/Pornography on Mobile Devices and the Impact on Children's Psychosocial Adjustment. Cyberpsychology, Behavior, and Social Networking. 2021 Jun 1;24(6):390-8.

23. Marsh S, Chu JT, Calder AJ. 'I tried to take my phone off my daughter, and i got hit in the face': a qualitative study of parents' challenges with adolescents' screen use and a toolbox of their tips. BMC public health. 2024 Jan 18;24(1):217.

24. Romero M. Digital literacy for parents of the 21st century children. Elearning Papers. 2014 May;38:32-40.

25. Terras MM, Ramsay J. Family digital literacy practices and children's mobile phone use. Frontiers in psychology. 2016 Dec 23;7:1957.

26. Sulistyo PB, Umarella FH, Mahmudah SM, Iza N. Digital Literacy Competence of Parents in Supervising Their Children Using Digital Media. International Journal of Social Science and Human Research. 2022 Feb:636-42.

27. Ahmed KR, Kolbe-Alexander T, Khan A. Effectiveness of a school-based intervention on physical activity and screen time among adolescents. Journal of science and medicine in sport. 2022 Mar 1;25(3):242-8.

28. Nwankwo F, Shin HD, Al-Habaibeh A, Massoud H. Evaluation of children's screen viewing time and parental role in household context. Global pediatric health. 2019 Sep;6:2333794X19878062.

29. Moawad GE, Ebrahem GG. The Relationship between Use of Technology and Parent-Adolescents Social Relationship. Journal of Education and Practice. 2016;7(14):168-78.4

30. Schneider LA, King DL, Delfabbro PH. Family factors in adolescent problematic Internet gaming: A systematic review. Journal of behavioral addictions. 2017 Sep;6(3):321-33.

List of tables

Table. Interview method and sampling

**Method**	**Type of schools**		**Type and number of participants**		
	**Bangla**	**English**	**Children**	**Parents**	**Teacher**

Formatted: Font: Bold

Formatted: Indent: First line: 0"

In-depth interviews (IDIs)	6	6	5	3	4
Focus Group Discussions (FGDs)	3	3	20	24	-

Table 2: Participant demographics (N=25).

**Variable**	**Frequency (n)**	**Percentage (%)**
**Sex**		
Male	14	56.0
Female	11	44.0
**Age (in years)**		
12	12	48
13	10	40
14	3	12
**Studying class/ grade**		
6th	2	8.0
7th	2	8.0
8th	12	48.0
9th	8	32.0
10th	1	4.00
**School type**		
Bangla Medium	16	64.0
English Medium	9	36.0

**Occupation of father**		
Business	16	64.0
Service holder	3	12.0
Labor	2	8.0
Skilled worker	1	4.0
Others	3	12.0
**Occupation of Mother**		
Housewife	23	92.0
Skilled worker	2	8.0
**Type of family**		
Nuclear	16	64.0
Extended	9	36.0
**Device owned by participant**		
Yes	15	60.0
No	10	40.0
**Type of internet access**		
Wi-Fi or Broadband	23	92.0
Mobile data	2	8.0
**Type of device used by the participant [multiple response]**		
Smart phone	25	100.0
Smart television	19	76.0

Laptop	11	44.0
Tablet	9	36.0
Computer	8	32.0

~~30.~~

Formatted: Font: (Default) +Body (Calibri), 11 pt

Formatted: Normal, Left, Indent: First line: 0.5", Line spacing: single, No bullets or numbering

September 29, 2025

To
Derebe Madoro Bunte
Academic Editor
PLOS ONE

Subject: Responses to Editor and Reviewer comments on the manuscript [PONE-D-25-02782] - [EMID:bd74e89f4f1ba54a]

Dear Editor,

Thank you for allowing us the opportunity to improve and resubmit our manuscript titled "**Parental monitoring challenges and recommendation for managing excessive screen use among children aged 12–14 years in Dhaka, Bangladesh: a qualitative exploration"**. We would like to extend our gratitude to the editor and reviewers for their valuable feedback and comments on our research manuscript. We have carefully reviewed all the comments and have addressed them individually. Additionally, we have incorporated the suggested changes into the manuscript where applicable. Below, please finds a detailed account of our responses to the comments from the editor and reviewers.

Response to Editor's Comments:

Comment: Please ensure that your manuscript meets PLOS ONE's style requirements, including those for file naming. The PLOS ONE style templates can be found at https://journals.plos.org/plosone/s/file?id=wjVg/PLOSOne_formatting_sample_main_body.pdf and https://journals.plos.org/plosone/s/file?id=ba62/PLOSOne_formatting_sample_title_authors_affiliations.pdf

Response: Thank you for the suggestion and provide the link. As suggested and following the links, we have revised the manuscript.

Comment: You indicated that you had ethical approval for your study. In your Methods section, please ensure you have also stated whether you obtained consent from parents or guardians of the minors included in the study or whether the research ethics committee or IRB specifically waived the need for their consent.

Response: Many thanks for this comment. We have obtained written consent from each participant and mentioned it in the ethical approval section. ***Please see Lines: 223-235.***

Comment: We note that the grant information you provided in the 'Funding Information' and 'Financial Disclosure' sections do not match.

Response: Thank you for pointing this out. We apologize for the inconsistency. We have carefully reviewed the grant details and updated the Funding Information and Financial Disclosure sections to ensure they are consistent and accurately reflect the source of funding.

Comment: When you resubmit, please ensure that you provide the correct grant numbers for the awards you received for your study in the 'Funding Information' section.

Response: We have verified the grant details and ensured that the correct grant numbers are provided in the Funding Information section.

Comment:
Thank you for stating the following financial disclosure: "Bangladesh government awarded project named Mujib 100 research Grant for women. I have received this award in 2022. Funded was $ 43,250 for 2 years project"

Please state what role the funders took in the study. If the funders had no role, please state: "The funders had no role in study design, data collection and analysis, decision to publish, or preparation of the manuscript."

If this statement is not correct you must amend it as needed.

Please include this amended Role of Funder statement in your cover letter; we will change the online submission form on your behalf.

Response: Thank you for your comment. The funders had no role in study design, data collection and analysis, decision to publish, or preparation of the manuscript. We have included this amended *Role of Funder* statement in the cover letter as suggested. Thanks once again for your kind support in making the change in the online system.

Comment: Please note that funding information should not appear in any section or other areas of your manuscript. We will only publish funding information present in the Funding Statement section of the online submission form. Please remove any funding-related text from the manuscript.

Response: Thank you for this clarification. We have removed all funding-related text from the manuscript.

Comment: Please provide a complete Data Availability Statement in the submission form, ensuring you include all necessary access information or a reason for why you are unable to make your data freely accessible. If your research concerns only data provided within your submission, please write "All data are in the manuscript and/or supporting information files" as your Data Availability Statement.

Response: Thank you for your guidance. We have revised the submission form and included a complete Data Availability Statement.

Comment: When completing the data availability statement of the submission form, you indicated that you would make your data available on acceptance. We strongly recommend all authors decide on a data sharing plan before acceptance, as the process can be lengthy and hold up publication timelines. Please note that, though access restrictions are acceptable now, your entire data will need to be made freely accessible if your manuscript is accepted for publication. This policy applies to all data except where public deposits would breach compliance with the protocol approved by your research ethics board. If you are unable to adhere to our open data policy, please kindly revise your statement to explain your reasoning and we will seek the editor's input on an exemption. Please be assured that once you have provided your new statement, the assessment of your exemption will not hold up the peer review process.

Response: Thank you for your advice regarding the data availability statement. We understand and acknowledge the journal's open data policy. However, our data cannot be made freely accessible due to icddr,b's data sharing policy. Accordingly, we have revised our Data Availability Statement to clarify this restriction and have noted that the data will be available upon reasonable request through icddr,b's Research Administration.

Comment: Please include your full ethics statement in the 'Methods' section of your manuscript file. In your statement, please include the full name of the IRB or ethics committee who approved or waived your study, as well as whether or not you obtained informed written or verbal consent. If consent was waived for your study, please include this information in your statement as well.

Response: Thank you for your guidance. We have revised the Methods section of the manuscript to include the full ethics statement. Specifically, we have added the name of the IRB that approved the study and clarified that informed written was obtained.

Comment: If the reviewer comments include a recommendation to cite specific previously published works, please review and evaluate these publications to determine whether they are relevant and should be cited. There is no requirement to cite these works unless the editor has indicated otherwise.

Response: Thank you for the clarification. We have carefully reviewed the suggested publications and evaluated their relevance to our study. We have cited those that are directly relevant and have not included those that are not applicable, in line with your guidance.

Additional Editor Comments:

- The manuscript requires substantial revision before acceptance, with mandatory changes including clarification of study design and methodology (operational definitions, inclusion/exclusion criteria, recruitment procedures, role of teachers, separation of FGDs and IDIs, complete demographic reporting), detailed ethical procedures, and rigorous description of the analysis process with supporting quotes for all themes. Recommended (non-essential) changes include restructuring recommendations using an ecological model, adding SES-linked analysis, presenting exemplary quotes in tables, and improving contextual relevance with local guidelines. While all reviewers agree on the topic's importance and the value of the findings, they consistently highlight gaps in methodological transparency, thematic presentation, and language clarity, with no direct

conflicts—thus authors should follow the converging advice to strengthen rigor, synthesis, and readability.

Response: We thank the editor and reviewers for their constructive feedback. We have revised the manuscript to clarify the study design and methodology, including operational definitions, inclusion/exclusion criteria, recruitment procedures, and the role of teachers; separated FGDs and IDIs; provided complete demographic reporting; detailed ethical procedures; and strengthened the analysis with supporting quotes for all themes. Additionally, we have addressed recommended suggestions by restructuring recommendations using an ecological model, adding SES-linked analysis, presenting exemplary quotes in tables, and improving contextual relevance. We believe these revisions enhance the manuscript's clarity, rigor, and readability.

Response to Reviewer 1

Comment: Thank you for your submission. Overall, this paper addresses what seems to be a key concern. The paper appears to do what it aimed to do, but the portrayal of the results and discussion hinders its impact, and the language and construction of the paper hinders readability. Literature needs to be further synthesized and used effectively to draw conclusions and develop arguments in introduction and discussion. With reworking and refining concerns, this could be a publishable paper.

Response: Thank you for the thoughtful feedback and recognition of the paper's importance. We have revised the manuscript to improve clarity and readability, refine the presentation of results and discussion, and better synthesize and integrate the literature to strengthen arguments and conclusions.

Comment: Grammar and word use not always appropriate for an academic article or unclear. (Lines 67, 74, 83, 84, 85, 86, 89, 101, 104, 112, 115) Overall the introduction could improve in flow, conciseness and effectiveness.

Response: Thank you for pointing this out. We have revised the manuscript thoroughly including the identified lines to correct grammar and word use, and improve the overall flow, conciseness, and effectiveness of the introduction.

Comment: The review of literature felt like a side-by-side review of the different studies, rather than synthesizing the information into specific sections to build into an argument to justify this paper. Justification for this study and the gap in literature was not fully developed in the introduction, therefore difficult to ascertain. Although there was a description of limited data on the topic, this was not argued so difficult to ascertain.

Response: Thank you for this valuable comment. We have synthesized the literature thematically, highlight the research gap more clearly, and strengthen the justification for the study in the introduction. ***Please see the lines: 54-119.***

Comment: I would suggest that this section is reworked to synthesise the evidence to make a stronger case for the evidence gap and justification for this study.

Response: We reworked the section to synthesize the evidence more effectively, highlight the gap, and present a stronger justification for this study. ***Please see the lines: 54-119.***

Comment: Remember we don't examine in qualitative work, but rather explore or understand, describe etc.

Response: We revised the wording to use more appropriate terms such as "explore," and "understand," in line with qualitative research conventions.

Comment: 'gather specific feedback on how to effectively utilize SV within urban Bangladesh context' doesn't make sense - for adolescent use? to create a family plan for SV? The second statement in the methods section makes much more sense.

Response: We have revised the statement for clarity to better reflect the intended meaning and have ensured consistency with the methods section. ***Please see the lines: 116-119.***

Comment: A cross-sectional design is linked to quantitative work, not qual. This appears to be a descriptive qualitative study. Please use references.

Response: We have clarified that this is a descriptive qualitative study and have updated the manuscript to reflect appropriate references supporting this approach. ***Please see the lines: 123-124.***

Comment: Please provide a description of the setting (i.e. North and South Dhaka City Corporations in Bangladesh) so that we have an understanding of the context which is vital for rigour, specifically transferability. Sampling and recruitment: Clear justification for why specific participants were chosen and heterogenous sampling was used. However, your recruitment strategy was unclear and the eligibility criteria was not apparent.

Response: Thank you for this detailed comment. We have added a clear description of the study setting, including North and South Dhaka City Corporations, to provide context and enhance transferability. We have also clarified our recruitment strategy, eligibility criteria, and the rationale for using heterogeneous sampling. ***Please see the lines: 137-148.***

Comment: How did you identify the schools from which to sample the children from? I see that you aimed for a heterogenous sample, but what criteria specifically did you use to select and approach these schools?

Response: We have clarified the criteria and process used to select and approach schools, ensuring that our sampling strategy achieved the intended heterogeneity of participants. ***Please see the lines: 157-162.***

Comment: How did you identify children and parents (of children) with excess screen use? How did you recruit them into the study? What were the age children were you sampling and why?

Response: We have clarified how children and their parents were identified based on excess screen use, detailed the recruitment process, and specified the age range of children sampled along with the rationale for these choices. ***Please see lines: 150-162.***

Comment: Were parents and children from the same families interviewed? How did you negotiate the ethics around this?

Response: We have clarified that parents and children were from the same families and have detailed how ethical considerations, including consent and confidentiality, were addressed in the study. **Please see lines: 223-235.**

Comment: Please ensure that you separate sampling and data collection procedures as this hinders the flow of the paper and makes for confusing reading. Data saturation has to do with the number of interviews and data collection, not sample size. Please review this section. We don't need to make a case for sample size when using purposive sampling, but rather focus on the eligibility criteria which guided your sampling of the participants.

Response: We have revised the manuscript to clearly separate sampling and data collection procedures, focused on eligibility criteria guiding purposive sampling, and clarified that data saturation relates to the number of interviews rather than sample size. ***Please see the lines: 150-186.***

Comment: You clarified the number of IDIs and FGDs you used, who they were with, where they were run, the language, the facilitators and how you developed the guides.

Response: We have revised the section to clearly separate sampling and data collection procedures, removed discussion of sample size, and focused on the eligibility criteria that guided our purposive sampling of participants. ***Please see the lines: 173-186.***

Comment: Please use references to support your processes?

Response: We have added appropriate references to support the processes used in our study. ***Please see the lines: 212-220.***

Comment: Please consolidate the data collection methods under one concise section, as this is not a streamlined passage.

Response: We have revised and concise the suggested point. ***Please see lines: 166-186.***

Comment: Methods seem appropriate for design. Would have been nice to see why these methods are useful to harness participants perspectives/experiences etc. or linked to the aim.

Response: We have clarified how the chosen methods are suited to capturing participants' perspectives and linked them explicitly to the study's aim.

Comment: 1.5 hrs appears very long for the children. What other strategies did you use to ensure that the data collection was rigorous for this specific age group? Looking at power differences etc. I am wondering how having the interviews at school could have influenced data collection as it is a place where children are safe, but not 'powerful'.

Response: We understand your concern. We have checked and clarified the strategies used to ensure rigorous data collection for this age group, including managing power dynamics, and have discussed how conducting interviews at school may have influenced the children's responses. Mentioned that each IDI lasted approximately 40-60 minutes and FGD up to maximum 90 minutes.

Comment: Please refer to the sources you used to guide this process? It appears that quantitative principles are being used to guide rigor strategies for qualitative work, which is confusing from a paradigm perspective and doesn't achieve its purpose.

Response: We have revised the manuscript to reference qualitative sources that guided our rigor strategies and have clarified the alignment of these strategies with qualitative research principles. ***Please see lines: 212-220.***

Comment: Rigor in data collection needs refinement, I would strongly urge you to refer to Lincoln and Guba's four constructs. This article refers to rigour in qualitative analysis specifically, however the principles can be applied to the project as a whole: International Journal of Qualitative Methods Thematic Analysis: Striving to Meet the Trustworthiness Criteria Volume 16: 1–13 DOI: 10.1177/160940691773384.

Response: We have refined the discussion of rigor in data collection and analysis by incorporating Lincoln and Guba's four constructs and have referenced the suggested article to strengthen the trustworthiness of our study. ***Please see lines: 212-220.***

Comment: Please refer to sources used to guide this process?

Response: We have added references to the sources that guided this process. ***Please see lines: 169.***

Comment: Outline the type of analysis used and the steps upfront (with reference).

Response: We have outlined the type of analysis used, detailed the steps upfront, and included appropriate references. ***Please see lines: 212-220.***

Comment: Clarify how you used the codebook and matrixes?

Response: We have clarified how the codebook and matrices were used to organize and analyze the data systematically. ***Please see lines: 166-186.***

Comment: No mention of reflexivity, this is vital in qualitative work to make your approach clear.

Response: We have added a section on reflexivity to clarify our approach and account for researcher influence in the study. ***Please see lines: 166-186.***

Comment: Were any specific ethical approaches needed as the study was working with children? How were these approached.

Response: We have detailed the specific ethical approaches used when working with children, including consent, assent, and safeguarding measures. ***Please see lines: 224-233.***

Comment: Add reimbursement to data collection for smooth reading

Response: We have included information on participant reimbursement to improve the flow of the manuscript. ***Please see lines: 233-235.***

Comment: It is not clear that table 1 only refers to student's characteristics. Why were characteristics of the other participants not reported?

Response: Thank you for the comment. We have clarified in the manuscript that Table 1 specifically presents the characteristics of the student participants only. The characteristics of parents and teachers were not reported in the table because the primary focus of this section was to describe the student sample, who were the main participants for exploring screen time behaviors. Demographic information for parents and teachers was summarized narratively in the Methods section to provide context without overloading the table, as their role was mainly to provide perspectives rather than be the primary subjects of analysis.

Comment: An initial overview of the themes related to challenges faced by parents needs to be provided. i.e. 4 themes were identified that consisted of…

Response: We have provided an initial overview of the themes related to challenges faced by parents, specifying the four main themes and their components. ***Please see lines: 260-432.***

Comment: It is expected that the information from the IDI and FGDs is synthesized as a part of thematic analysis, not presented separately, so this doesn't need to be explained.

Response: We have revised the manuscript to synthesize information from the IDIs and FGDs within the thematic analysis rather than presenting them separately.

Comment: Why are the recommendations' themes written as bullet points and do not follow the same process as the challenges with quotes to illustrate your point. The same structure should be used? These are still themes that you identified and need evidence. This section needs to be reworked to follow a similar structure and it would seem that some of the items fall under the same theme. I would consider using the ecological model to structure this section and place recommendations under the different levels as themes (i.e. micro, meso and macro) Please see this as an example: Harper CR, Steiner RJ, Brookmeyer KA. Using the Social-Ecological Model to Improve Access to Care for Adolescents and Young Adults. J Adolesc Health. 2018 Jun;62(6):641-642. doi: 10.1016/j.jadohealth.2018.03.010. PMID: 29784107; PMCID: PMC10898617.

Response: Thank you for this valuable suggestion. We have restructured the recommendations section to align with the format used for the challenges section, providing illustrative quotes from participants for each theme. Additionally, we have organized the recommendations using the social-ecological model, grouping them under micro (individual and family), meso (school and community), and macro (national policy) levels. This approach allows readers to clearly see the thematic structure, the supporting evidence from participants, and the relationships between different recommendations. By applying this framework, overlapping items were consolidated under broader themes, making the presentation more coherent and consistent with qualitative analysis standards. ***Please see lines: 433-513.***

Comment: General clarity, sentence structure, word usage and grammar needs reworking.

Response: We have carefully revised the manuscript to improve clarity, sentence structure, word usage, and grammar throughout.

Comment: Evidence/quote provided does not always match statement that it is linked to. (i.e. line 289)
Response: We have reviewed the manuscript and ensured that all evidence and quotes are correctly linked to the corresponding statements.

Comment: In the first section (challenges), children, parents and teachers often say the same thing. It would be more impactful for me if these topics are discussed with the evidence from the participants synthesised together, with only their unique contributions/differences discussed separately in another paragraph with evidence, rather than presenting each group of participants results separately under each heading.

Response: We have revised the challenges section to synthesize evidence from children, parents, and teachers, highlighting shared perspectives together and presenting unique contributions or differences separately with supporting evidence.

Comment: Why are some words in italics? Are these direct quotes? I would suggest using quote marks to make this clear.

Response: We have revised the manuscript to use quotation marks for all direct quotes and followed the journal formats.

Comment: General clarity, sentence structure, word usage and grammar needs reworking.

Response: We have thoroughly revised the manuscript to improve clarity, sentence structure, word usage, and grammar throughout.

Comment: This section needs reworking – rather than reporting what other evidence notes, you need to use this other research to link and make recommendations from your own research. It is fine to draw the similarities between your and other research as a mechanism to situate your evidence, but rather than report what other studies found, use it to build the discussion around your

evidence.

Response: We have reworked this section to use existing research to contextualize our findings and inform recommendations, rather than merely reporting other studies, ensuring the discussion is centered on our evidence.

Comment: Limitations:

- Why didn't you have enough time to do separate FGDs?
- The context of a study is not a limitation, rather provide a think description of the setting so that results are transferable to similar settings.
- Qualitative research does not aim to be generalisable and therefore this is not a limitation of the study

Response: Thank you for these comments. We have clarified the reasons for not conducting separate FGDs and have revised the limitations (removed the specific point) section to focus on relevant methodological considerations. We have also removed references to generalizability and contextual factors as limitations, providing a detailed description of the setting to enhance transferability instead.

Response to Reviewer 2

Comment: Check typos in line 77, 204.

Response: We have checked lines and addressed the issues accordingly.

Comment: Put phrases or acronyms in uniform, line 171 IDI so line 174,177 too.

Response: We have standardized the use of phrases and acronyms throughout the manuscript, including IDI and related terms.

Comment: Line 180-183, please have a look and rephrase.

Response: We have rephrased lines 180–183 for improved clarity and readability.

Comment: Please clarify the participants and the numbers of participants in line 160 and so forth.

Response: We have clarified the participants and specified the number of participants at line 160 and other relevant sections.

Comment: Line 216: clarify multiple sources. What are the three types of data collection tools

Response: We have clarified what is meant by multiple sources and specified the three types of data collection tools used in the study. ***Please see lines: 167-186.***

Comment: What is your measure to determine "excessive use of internet or screen view in this study?"

Response: We have clarified that “excessive use of internet or screen time” in this study was defined as more than 2 hours per day.

Response to Reviewer 3

Comment: This study tackles an important and relevant topic with valuable insights and practical recommendations that could significantly contribute to the field. The authors have conducted meaningful research that addresses real challenges faced by parents in managing adolescent screen use. However, the manuscript requires substantial methodological clarification and improved presentation of results to meet publication standards. The findings and recommendations are strong, but the research would benefit from more rigorous reporting and clearer methodology to enhance its impact and reproducibility.

Response: We sincerely thank the reviewer for recognizing the relevance and value of our study. We have revised the manuscript to provide clearer methodological details, improve the presentation of results, and enhance rigor and reproducibility, addressing the reviewer’s suggestions to strengthen the overall impact of the research.

Comment: Article Summary: This qualitative study explores the challenges parents face in monitoring their adolescents' (aged 12-14) screen use in Dhaka, Bangladesh. Using focus group discussions, in-depth interviews, and key informant interviews with 25 children, parents, and teachers from six schools, the study identifies four main challenges: educational necessity of devices creating supervision difficulties, restricted outdoor activities, increasing device dependence, exposure to age-inappropriate content, and parental digital illiteracy. The study provides comprehensive recommendations including school-based interventions, national-level policies, enhanced parent-child bonding, and secured infrastructure development.

Response: We thank the reviewer for providing a clear summary of our study. We appreciate the acknowledgement of the challenges identified and the recommendations provided. We have ensured that the manuscript reflects these key findings accurately and comprehensively.

Comment: Comprehensive multi-stakeholder approach: The inclusion of children, parents, and teachers provides valuable triangulation of perspectives on screen use challenges

Response: We thank the reviewer for highlighting this strength. We have ensured that the manuscript emphasizes the value of including multiple stakeholders to provide a well-rounded understanding of challenges related to adolescent screen use.

Comment: Practical and actionable recommendations: The study offers concrete suggestions for schools, parents, and policymakers that are contextually relevant to the Bangladeshi setting

Response: We thank the reviewer for recognizing the practical relevance of our recommendations. We have ensured that these actionable suggestions for schools, parents, and policymakers are clearly presented and contextualized for the Bangladeshi setting.

Comment: Important and timely research topic: Addresses a critical public health concern regarding adolescent screen use in a rapidly digitizing society

Response: We thank the reviewer for acknowledging the importance and timeliness of our research. We have emphasized the public health significance of adolescent screen use in a rapidly digitizing society throughout the manuscript.

Comment: Rich qualitative data: The combination of FGDs, IDIs, and KIIs provides depth and breadth to understanding the phenomenon

Response: We thank the reviewer for recognizing the richness of our qualitative data. We have highlighted how the combination of FGDs and IDIs contributed to a comprehensive understanding of the challenges associated with adolescent screen use.

Comment: Cultural contextualization: Appropriately situates findings within the social and educational context of Dhaka, Bangladesh

Response: We thank the reviewer for highlighting this point. We have ensured that the findings are appropriately contextualized within the social and educational environment of Dhaka, Bangladesh.

Comment: Lack of clear operational definitions

Response: We have added clear operational definitions for all key terms in the manuscript. ***Please see lines: 188-204.***

Comment: Insufficient inclusion/exclusion criteria

Response: We have specified detailed inclusion and exclusion criteria for all participant groups, including age, technology use, and caregiver role. ***Please see lines: 151-162.***

Comment: Unclear data collection procedures

Response: We have clarified all data collection procedures, including interviews, focus groups, and key informant interviews, and the process for achieving thematic saturation. ***Please see lines: 166-186.***

Comment: Inadequate sample size justification

Response: We have clarified the rationale for the sample size, distinguishing between interviews and focus groups, and explained how saturation was determined. ***Please see lines: 151-162.***

Comment: Missing inter-rater reliability measures

Response: We have described the coding process and measures taken to ensure consistency and reliability between coders. ***Please see lines: 167-186.***

Comment: Incomplete demographic reporting.

Response: We have now provided relevant complete demographic details for all participant groups. Please see table 1 and 2.

Comment: Incomplete consent procedures

Response: We have clarified the consent and assent procedures, including ethical considerations for working with children. ***Please see lines: 223-235.***

Comment: Grammar issues

Response: We have thoroughly revised the manuscript to improve grammar, clarity, and sentence structure.

Comment: Presentation and organization

Response: We have reorganized sections for better flow, coherence, and readability, ensuring consistent structure throughout the manuscript.

Comment: Suggest using a grammar checker and proofreading – a lot of misspellings and run on sentences which make it hard to read the paper

Response: We have thoroughly proofread the manuscript and used grammar-checking tools to correct misspellings, run-on sentences, and improve overall readability.

Comment: Paragraph 1 - link between parenting and SES is established but it is unclear as to how parenting relates to health of adolescents ◊ explicitly state that parenting style is a mediator of technology use and thus, health of adolescents

Response: Parental monitoring, communication with adolescents and emotional care play a crucial role in shaping adolescents' use of technological devices and their related health outcomes. Additionally, positive parenting and strong parent-adolescent relationships are strongly linked to responsible technology use and improved mental health. Because inconsistent monitoring and supervision of parents can lead to higher risks of internet addiction, problematic usage of devices and adverse health outcomes, including anxiety, depression and attention-related problems.

Comment: Line 95 – please mention whether the screen time usage is a mean or median measure

Response: The median recreational screen time was found to be 4.0 hours/day; with boys having longer times (4.3 hours/day) compared to girls (3.6 hours/day).

Comment: Line 105 – author's mention AAP. The study context is Dhaka, Bangladesh, are there any Bangladeshi guidelines for pediatrics that should be mentioned? It will help strengthen the context and be directly applicable

Response: No. there is no Bangladeshi guideline for pediatrics which can be directly applicable.

Comment: Currently, paragraphs 1,2, 3 are not logically organized – suggestion for organization ◊ paragraph 1: clearly outline the problems on health brought forth by SV; paragraph 2: outline the literature for why parents are intentionally/unintentionally using technology to help with parenting; paragraph 3: outline existing pediatric guidelines and perspectives for healthier management of technology use among adolescents
Response: We have reorganized paragraphs 1–3 as suggested, clearly outlining the health problems related to screen viewing, literature on parental use of technology, and existing pediatric guidelines for managing adolescent technology use.

Comment: Line 160 – please quantify "excessive use" of digital devices using a threshold (for example, > 5 hours/day, etc.)

Response: We have quantified "excessive use" of digital devices as more than 2 hours per day in line 160.

Comment: Lines 160 – Lines 167 – please be specific with inclusion and exclusion criteria for the study. Please list the minimum age inclusion/exclusion for each participant, along with any other inclusion/exclusion criteria that was relevant to the study (e.g. technology use, language, family size, etc.) Please try to be specific in this section.

Response: Thank you for this comment. We have revised lines 160–167 to provide specific inclusion and exclusion criteria for all participant groups. Parents included were primary caregivers of children with excessive digital device use (>2 hours/day) who set rules regarding screen time. Adolescents included were boys and girls aged 12–14 years with excessive digital use (>2 hours/day). Teachers included were those experienced in managing students with high screen time, implementing school digital policies, and providing digital literacy education. Additional relevant criteria, such as language proficiency and engagement with technology, have also been specified.

Comment: Line 165 – while teacher's play a role in children's development and education, it is unclear as to how they would be valuable as key informants for the aim of the research as they are not providing child care; the introduction argues that SV has come on as a way of making caregiving easier. Please include some literature/citations that can help justify the inclusion of teachers as I do agree that they are an important perspective to consider.

Response: We have clarified and justified the inclusion of teachers as key informants. Teachers included were those with experience managing adolescents with excessive digital device use and implementing school policies on screen time. Their perspectives were valuable as they observe students' screen use in educational and social contexts and provide digital literacy education. We

have also added relevant literature to support the importance of including teachers in studies on adolescent screen use and caregiving dynamics.

Comment: Line 171 – please define how these "class register books" were obtained

Response: Thank you for pointing this out. We have clarified that adolescent students were purposively selected from the class register books, which were obtained with permission from the respective school authorities.

Comment: Regarding focus groups – were children and their parents both present in the same focus groups? This poses a concern for social desirability bias on the end of the children to appease to their parents. Please clarify if parents and children had separate focus groups (parents only and children only). If parents and children were present in the same focus group, please justify why this decision was made and how bias arising from potential social desirability bias was avoided.

Response: We have clarified that separate focus group discussions (FGDs) were conducted for children and parents, taking into account diverse classes, ages, and genders. This approach ensured a wide range of perspectives and experiences, minimized social desirability bias, and encouraged richer insights from participants.

Comment: Lines 180 – 184 – please condense language to reflect that interviews and focus groups were conducted until thematic saturation was reached.

Response: The point of saturation or point of redundancy was determined based on an "intellectual guiding principle" for deciding how many interviews to conduct in a qualitative study with purposive sampling as its basis. As mentioned, it is only an intellectual guiding principle with little or no on-the-ground guidance to sample size estimation that will be required to reach this saturation. To comprehend the challenges and recommendations for monitoring screen use of adolescents, a total of 18 interviews were conducted, including 12 IDIs and 6 FGD sessions till thematic saturation was achieved.

Comment: Line 185 – Please clarify the 18 interviews – I am only counting 12 interviews (5 students, 3 parents, and 4 key informant teacher interviews); please note that focus group discussions should not be included in the interview sample size and should be listed separately. E.g., "A total of 12 interviews and 6 FGDs were conducted." Additionally, please list the sample size, N for easier reading.

Response: To understand the challenges and recommendations for monitoring adolescent screen use, a total of 12 interviews (12 IDIs) and 6 FGDs were conducted until thematic saturation was achieved.

Comment: Table 1 – Please change the "School Types" column to be more reflective of categorization based on language of teaching as "school types" suggest classification based on funding – private v. public.

Response: Changed the "School Types" into "Medium of Instruction at Schools".

Comment: Line 191 – Please include/indicate how sociodemographic data was collected from participants.

Response: A section on sociodemographic-related questions was included in every guideline to gather the socio-demographic information of the participants.

Comment: Line 193 – Please include guidelines in the appendix. Alternatively, provide brief descriptions of the contents of the guidelines – did they include suggestions for how long interviews should last? Did they include any probes? How many questions were there? What themes did the interview/FGD questions explore, if any?

Response: Guidelines are included in the appendix, as suggested.

Comment: Line 197 – please update the sentence to state "three and five years of experience on qualitative data collection, respectively."

Response: We have updated the sentence. **Please see the lines: 166-186.**

Comment: Lines 200 – 203 – Which language were the interviews/FGDs conducted in? Were the interviews/FGD conducted in English at English-medium schools and in Bangla at Bangla-medium schools? Please clarify

Response: All interviews were carried out in Bangla, the national language of Bangladesh. **Please see the lines: 173-186.**

Comment: Line 201 – how long were key informant interviews?

Response: There was an understanding gap, and we have resolved this in revised version. **Please see the lines: 151-162.**

Comment: Line 208 – Please use gender neutral pronouns to describe the facilitator

Response: One researcher acted as the facilitator, and the other used the recorder to record the interviews, taking notes simultaneously. **Please see the lines: 166-186.**

Comment: Line 205 – Please clarify if the researcher with the recorder also took notes.

Response: One researcher acted as the facilitator, and the other used the recorder to record the interviews, taking notes simultaneously. **Please see the lines: 166-186.**

Comment: Lines 213- 218 – How was data validity maintained/triangulated? Were the same participants interviewed multiple times? Were parent – child dyads tracked for this triangulation? This section is very unclear – please provide more details regarding the methodology, maybe including a figure would be helpful.

Response: We have now provided a detailed explanation for it. **Please see lines: 206-220.**

Comment: Lines 219 – 220 – Data interpretation should be talked about in the analysis section, please remove this sentence.

Response: Removed

Comment: Line 221 – Please clarify validating data cross various contexts. Validity of data is usually not a concern in one-time interviews or focus group participations. There are usually limitations of self-report or social desirability biases, but it is unrealistic to have responses free of bias in qualitative studies. Who/what is this data being validated against. Is there a standard respondent? Alternatively, are the authors checking for quality of data, i.e., easy to comprehend and bug-free recordings or recordings that are easy to download/transcribe? Please clarify.

Response: Multiple supervisors and investigators were involved to minimise bias and provide a comprehensive interpretation of the data. The use of data and methodological triangulation enabled comprehensive analysis across different research sites and methods. Comparisons between key informants, parents, and students enhanced the validity of the findings. **Please see lines: 206-220.**

Comment: Line 226 – please provide an example file nomenclatures; e.g. "Participant001_English_IDI" or something along those lines that can let readers know of how files were stored in case they want to replicate.

Response: Thank you for the suggestion. We have now clarified the file nomenclature in the manuscript. For ease of organization and replication, all interview files were stored using a standardized naming convention that included the participant number, language of the interview, and interview type. For example: "Participant001_English_IDI" or "Participant012_Bangla_FGD". This approach ensured anonymity while allowing systematic identification and retrieval of files during analysis.

Comment: Line 228 – Earlier it is mentioned that interviews were recorded, but there is now a note implying that some interviews were not recorded. Please explain.

Response: Checked and edited accordingly. **Please see lines: 167-176.**

Comment: Line 231 – Please clarify if there were any consensus meetings to finalize the codebook. Consensus for codebook development is a foundational part of qualitative analysis. Additionally, please include the final codebook in the appendix as it will be helpful to other investigators conducting similar studies.

Response: The research team had a discussion on the developed codebook and finalized the codebook after addressing the critical review and feedback. **Please see lines: 167-186.**

Comment: Line 232– Please list the initials of those involved in the coding process (analysis team).

Response: The first stage of qualitative data analysis began by organising and preparing the field data, including interviews and field notes, by SHK and NS. **Please see lines: 206-220.**

Comment: Lines 236-238 – Rather than validity of data, I am more interested in the inter-rater reliability. Please clarify the exact coding methods – was a double-coding or single-coding method used. Or were a select few transcripts coded by all the analysis team members at the start to ensure consistency in coding before single-coding. Alternatively, did all the analysis team members code all the transcripts? Additionally, please provide Cohen's kappa for a measure of inter-rater reliability.

Response: Thank you for the comment. To clarify, we used a double-coding approach at the initial stage of analysis to ensure consistency and reliability in coding. This approach ensured rigor and consistency in our qualitative coding process. **Please see lines: 206-220.**

Comment: Line 241 – As children (<18) were involved in the study, please elaborate on their consent process. Was parental assent required?

Response: Revised ethical approval process *"The study protocol was approved by the Institutional Review Board (IRB) of icddr,b (Protocol No: PR-22002). Informed written consent was taken from each respondent (parents, teacher) and students were taken consent from their parents as well as assent was taken as their age was above 11 years prior to the interview. All the respondents were informed in Bengali about their rights related to their voluntary participation in the study and withdrew from the interview at any time during the interview. Interviewers of this study clearly conveyed to the respondents the objectives of the study, reasons for their participation in the study, their role, risks, benefits, anonymity and confidentiality, including the rights not to participate or withdraw. After getting acquainted with all these issues, the respondents decided whether to participate in this study. Confidentiality during and after data collection was ensured, and no identifiable information was used that may expose the identity of the participants. Only the research team members have access to the collected data, which were stored securely. After the interview, each participant received a gift (equivalent to BDT370 [USD3] as a token of appreciation for their valuable time."*

Comment: Line 249 – Where were the recordings/data stored? Please provide a location e.g. "password protected computer only accessible by the research team." Additionally, were the interviews themselves de-identified? Please clarify.

Response: Confidentiality during and after data collection was ensured, and no identifiable information was used that may expose the identity of the participants. Only the research team members have access to the collected data, which were stored securely in the password-protected central data storage of icddr,b.

Comment: Line 250 – Please specify the token of appreciation amount (in Bangladeshi currency and USD).

Response: We have specified the token of appreciation amount, provided in both Bangladeshi Taka and its equivalent in USD. **Please see lines: 219-220 as follows "*After the interview, each***

participant received a gift (equivalent to BDT370 [USD3] as a token of appreciation for their valuable time."

Comment: Line 253 – Please note that Table 2 only provides the demographics for children who participated in the study (from what I gather). Please update language to reflective of this specific demographic. As parents and teachers were also interviewed/included in the study, please add additional tables for demographic characteristics of each of these groups. Alternatively, you can create one large table and have subgroup frequencies included as columns (column 1 = children, column 2 = parents, column 3 = teachers)

Response: Thank you for this comment. We have clarified in the manuscript that Table 2 presents the demographic characteristics of the child participants only. To provide a complete overview of all study participants, we have added additional tables reporting the demographics of parents and teachers. Alternatively, we have also considered creating a combined table with subgroup frequencies in separate columns (children, parents, teachers) to allow easy comparison across participant groups. This ensures transparency and allows readers to understand the composition of all participants included in the study.

Comment: Line 263 – 268 – please provide your definition of screen viewing and then provide a summary of responses/content analysis by participant subgroup that relate to screen viewing as a concept. To strengthen this paragraph, you can also summarize any quotes or general remarks that point towards relations between screen viewing and health.

Response: We have provided a clear definition of screen viewing and summarized responses by participant subgroup. We have also included quotes and remarks highlighting the relationships between screen viewing and health to strengthen this section.

Comment: Lines 325 – 332 – please provide more context using literature or another source to contextualize the social climate in Dhaka.

Response: Revised the text: Dhaka, a densely populated megacity, has limited open and child-friendly spaces, restricting children's opportunities for outdoor play. Concerns over traffic, pollution, and safety further confine children indoors. At the same time, rapid expansion of internet and smartphone access has made screen-based activities more available and attractive. Together, these factors create a social climate that favors indoor, screen-based leisure—an important context for interpreting participants' views in this study. **Please see lines: 76-90.**

Comment: Lines 394 – 403 – please provide an exemplary quote to support the claim being made. It is interesting and I would like to read what was said.

Response: We have added an exemplary quote from participants to support the claim and provide greater insight into their perspectives.

Comment: Line 410 – please check the formatting of the quote.

Response: Tanks. Formatted accordingly

Comment: Line 431 – please include a transition paragraph that segways into the new subsection of recommendations. Also, please consider condensing these recommendations and moving them to the discussion section or including them as a table. Great work on the recommendations!

Response: The study participants did not limit themselves to sharing only the challenges that they faced with morning screen time but also provided some contextualized recommendations to address these challenges. **Please see lines: 433-513 and entire discussion part.**

Comment: Line 484 – Please rename the subheading to "ensure social safety"

Response: Changed to "Ensuring social security"

Comment: General note – I am missing quotes or analysis based on SES status as that seemed to be a big factor influencing health of adolescents and technology use according to the introduction written by the author's. Please include 1 or 2 quotes that highlight any relationships of SES, technology use, and health/concerns.

Response: Thank you for this important observation. We have now incorporated participants' perspectives highlighting the influence of socioeconomic status (SES) on screen use and related health concerns. For instance, one parent noted: "Families with higher income often provide multiple devices to their children, which makes it harder to control screen time and can affect their sleep and concentration." Similarly, a student mentioned: "Some of my friends don't have personal devices at home, so they play outside more, and I think they stay healthier than those who spend all day on phones or tablets." These quotes illustrate how SES shapes access to technology and, consequently, children's screen habits and associated health outcomes. ***Please see lines: 369-378.***

Comment: Lines 533 – 537 – I appreciate the domains/points brought up regarding SV (e.g. not all screens are equal). It would potentially be helpful to include a table in the results section listing exemplary quotes organized by these domains for easier comprehension.

Response: Thank you for this comment. We tried to incorporate in text.

Comment: Line 567 – please elaborate. The paragraph is only one sentence currently.

Response: We have elaborated on the paragraph at line 567, expanding it to provide greater detail and context.

Comment: Line 583 – what were the results of the clustered randomized controlled trial?

Response: Here is the result: Total physical activity (PA), measured in MET-min/week, increased from baseline to 8 and 12 weeks in the intervention group (IG) by 3%–5%, whereas it decreased in the control group (CG) by 5%–3%. Over time, PA improved significantly in the IG compared to the CG ($p < 0.001$). Average sedentary time (ST) in minutes per day decreased from baseline to 8 and 12 weeks in the IG by 28%–35%, while remaining largely unchanged in the CG (6%–5%). At 12 weeks, the IG had a significantly lower average ST than the CG. Additionally, average

knowledge scores on PA and sedentary behavior (SB) were significantly higher in the IG than in the CG at 12 weeks ($p < 0.001$).

Comment: Line 608 – I like the call to action being presented here. To tighten narrative, please tie it in or include how existing pediatric guidelines could be modified. Alternatively, should we consider devising new pediatric guidelines? Curious to hear your thoughts.

Response: We have tied the call to action to existing pediatric guidelines, discussing potential modifications and the possibility of developing new guidelines to better address adolescent screen use in the current context.

Comment: Under limitations, please also include the influence of social desirability biases among participants or potential selection for responses given the skew towards Bangla-medium participants in the study. Additionally, please include limitations related to potential human error (such as not recording interviews, errors in translating from Bangla to English or vice versa).

Response: Thanks for this comment and suggestion. We have reviewed our manuscript carefully and removed the limitation points. Since it’s a qualitative study and there is no generalizations issue like quantitative study, we have removed the section.

Sincerely
Dr. Shahria Hafiz Kakon
On behalf of Nahian Sultana,Bidhan Krishna Sarker, Tanjir Rashid Soron, Rashidul Hoque, Fahmida Tofail

September 29, 2025

To
Derebe Madoro Bunte
Academic Editor
PLOS ONE

Subject: Responses to Editor and Reviewer comments on the manuscript [PONE-D-25-02782] - [EMID:bd74e89f4f1ba54a]

Dear Editor,

Thank you for allowing us the opportunity to improve and resubmit our manuscript titled "**Parental monitoring challenges and recommendation for managing excessive screen use among students aged 12–14 years in Dhaka, Bangladesh: a qualitative exploration**". We would like to extend our gratitude to the editor and reviewers for their valuable feedback and comments on our research manuscript. We have carefully reviewed all the comments and have addressed them individually. Additionally, we have incorporated the suggested changes into the manuscript where applicable. Below, please finds a detailed account of our responses to the comments from the editor and reviewers.

Response to Editor's Comments:

Comment: Please ensure that your manuscript meets PLOS ONE's style requirements, including those for file naming. The PLOS ONE style templates can be found at
https://journals.plos.org/plosone/s/file?id=wjVg/PLOSOne_formatting_sample_main_body.pdf
and
https://journals.plos.org/plosone/s/file?id=ba62/PLOSOne_formatting_sample_title_authors_affiliations.pdf

Response: Thank you for the suggestion and provide the link. As suggested and following the links, we have revised the manuscript.

Comment: You indicated that you had ethical approval for your study. In your Methods section, please ensure you have also stated whether you obtained consent from parents or guardians of the minors included in the study or whether the research ethics committee or IRB specifically waived the need for their consent.

Response: Many thanks for this comment. We have obtained written consent from each participant and mentioned it in the ethical approval section. ***Please see Lines: 223-235.***

Comment: We note that the grant information you provided in the 'Funding Information' and 'Financial Disclosure' sections do not match.

Response: Thank you for pointing this out. We apologize for the inconsistency. We have carefully reviewed the grant details and updated the Funding Information and Financial Disclosure sections to ensure they are consistent and accurately reflect the source of funding.

Comment: When you resubmit, please ensure that you provide the correct grant numbers for the awards you received for your study in the 'Funding Information' section.

Response: We have verified the grant details and ensured that the correct grant numbers are provided in the Funding Information section.

Comment:
Thank you for stating the following financial disclosure: "Bangladesh government awarded project named Mujib 100 research Grant for women. I have received this award in 2022. Funded was $ 43,250 for 2 years project"

Please state what role the funders took in the study. If the funders had no role, please state: "The funders had no role in study design, data collection and analysis, decision to publish, or preparation of the manuscript."

If this statement is not correct you must amend it as needed.

Please include this amended Role of Funder statement in your cover letter; we will change the online submission form on your behalf.

Response: Thank you for your comment. The funders had no role in study design, data collection and analysis, decision to publish, or preparation of the manuscript. We have included this amended *Role of Funder* statement in the cover letter as suggested. Thanks once again for your kind support in making the change in the online system.

Comment: Please note that funding information should not appear in any section or other areas of your manuscript. We will only publish funding information present in the Funding Statement section of the online submission form. Please remove any funding-related text from the manuscript.

Response: Thank you for this clarification. We have removed all funding-related text from the manuscript.

Comment: Please provide a complete Data Availability Statement in the submission form, ensuring you include all necessary access information or a reason for why you are unable to make your data freely accessible. If your research concerns only data provided within your submission, please write "All data are in the manuscript and/or supporting information files" as your Data Availability Statement.

Response: Thank you for your guidance. We have revised the submission form and included a complete Data Availability Statement.

Comment: When completing the data availability statement of the submission form, you indicated that you would make your data available on acceptance. We strongly recommend all authors decide on a data sharing plan before acceptance, as the process can be lengthy and hold up publication timelines. Please note that, though access restrictions are acceptable now, your entire data will need to be made freely accessible if your manuscript is accepted for publication. This policy applies to all data except where public deposits would breach compliance with the protocol approved by your research ethics board. If you are unable to adhere to our open data policy, please kindly revise your statement to explain your reasoning and we will seek the editor's input on an exemption. Please be assured that once you have provided your new statement, the assessment of your exemption will not hold up the peer review process.

Response: Thank you for your advice regarding the data availability statement. We understand and acknowledge the journal's open data policy. However, our data cannot be made freely accessible due to icddr,b's data sharing policy. Accordingly, we have revised our Data Availability Statement to clarify this restriction and have noted that the data will be available upon reasonable request through icddr,b's Research Administration.

Comment: Please include your full ethics statement in the 'Methods' section of your manuscript file. In your statement, please include the full name of the IRB or ethics committee who approved or waived your study, as well as whether or not you obtained informed written or verbal consent. If consent was waived for your study, please include this information in your statement as well.

Response: Thank you for your guidance. We have revised the Methods section of the manuscript to include the full ethics statement. Specifically, we have added the name of the IRB that approved the study and clarified that informed written was obtained.

Comment: If the reviewer comments include a recommendation to cite specific previously published works, please review and evaluate these publications to determine whether they are relevant and should be cited. There is no requirement to cite these works unless the editor has indicated otherwise.

Response: Thank you for the clarification. We have carefully reviewed the suggested publications and evaluated their relevance to our study. We have cited those that are directly relevant and have not included those that are not applicable, in line with your guidance.

Additional Editor Comments:

- The manuscript requires substantial revision before acceptance, with mandatory changes including clarification of study design and methodology (operational definitions, inclusion/exclusion criteria, recruitment procedures, role of teachers, separation of FGDs and IDIs, complete demographic reporting), detailed ethical procedures, and rigorous description of the analysis process with supporting quotes for all themes. Recommended (non-essential) changes include restructuring recommendations using an ecological model, adding SES-linked analysis, presenting exemplary quotes in tables, and improving contextual relevance with local guidelines. While all reviewers agree on the topic's importance and the value of the findings, they consistently highlight gaps in methodological transparency, thematic presentation, and language clarity, with no direct

conflicts—thus authors should follow the converging advice to strengthen rigor, synthesis, and readability.

Response: We thank the editor and reviewers for their constructive feedback. We have revised the manuscript to clarify the study design and methodology, including operational definitions, inclusion/exclusion criteria, recruitment procedures, and the role of teachers; separated FGDs and IDIs; provided complete demographic reporting; detailed ethical procedures; and strengthened the analysis with supporting quotes for all themes. Additionally, we have addressed recommended suggestions by restructuring recommendations using an ecological model, adding SES-linked analysis, presenting exemplary quotes in tables, and improving contextual relevance. We believe these revisions enhance the manuscript's clarity, rigor, and readability.

<u>Response to Reviewer 1</u>

Comment: Thank you for your submission. Overall, this paper addresses what seems to be a key concern. The paper appears to do what it aimed to do, but the portrayal of the results and discussion hinders its impact, and the language and construction of the paper hinders readability. Literature needs to be further synthesized and used effectively to draw conclusions and develop arguments in introduction and discussion. With reworking and refining concerns, this could be a publishable paper.

Response: Thank you for the thoughtful feedback and recognition of the paper's importance. We have revised the manuscript to improve clarity and readability, refine the presentation of results and discussion, and better synthesize and integrate the literature to strengthen arguments and conclusions.

Comment: Grammar and word use not always appropriate for an academic article or unclear. (Lines 67, 74, 83, 84, 85, 86, 89, 101, 104, 112, 115) Overall the introduction could improve in flow, conciseness and effectiveness.

Response: Thank you for pointing this out. We have revised the manuscript thoroughly including the identified lines to correct grammar and word use, and improve the overall flow, conciseness, and effectiveness of the introduction.

Comment: The review of literature felt like a side-by-side review of the different studies, rather than synthesizing the information into specific sections to build into an argument to justify this paper. Justification for this study and the gap in literature was not fully developed in the introduction, therefore difficult to ascertain. Although there was a description of limited data on the topic, this was not argued so difficult to ascertain.

Response: Thank you for this valuable comment. We have synthesized the literature thematically, highlight the research gap more clearly, and strengthen the justification for the study in the introduction. ***Please see the lines: 54-119.***

Comment: I would suggest that this section is reworked to synthesise the evidence to make a stronger case for the evidence gap and justification for this study.

Response: We reworked the section to synthesize the evidence more effectively, highlight the gap, and present a stronger justification for this study. ***Please see the lines: 54-119.***

Comment: Remember we don't examine in qualitative work, but rather explore or understand, describe etc.

Response: We revised the wording to use more appropriate terms such as "explore," and "understand," in line with qualitative research conventions.

Comment: 'gather specific feedback on how to effectively utilize SV within urban Bangladesh context' doesn't make sense - for adolescent use? to create a family plan for SV? The second statement in the methods section makes much more sense.

Response: We have revised the statement for clarity to better reflect the intended meaning and have ensured consistency with the methods section. ***Please see the lines: 116-119.***

Comment: A cross-sectional design is linked to quantitative work, not qual. This appears to be a descriptive qualitative study. Please use references.

Response: We have clarified that this is a descriptive qualitative study and have updated the manuscript to reflect appropriate references supporting this approach. ***Please see the lines: 123-124.***

Comment: Please provide a description of the setting (i.e. North and South Dhaka City Corporations in Bangladesh) so that we have an understanding of the context which is vital for rigour, specifically transferability. Sampling and recruitment: Clear justification for why specific participants were chosen and heterogenous sampling was used. However, your recruitment strategy was unclear and the eligibility criteria was not apparent.

Response: Thank you for this detailed comment. We have added a clear description of the study setting, including North and South Dhaka City Corporations, to provide context and enhance transferability. We have also clarified our recruitment strategy, eligibility criteria, and the rationale for using heterogeneous sampling. ***Please see the lines: 137-148.***

Comment: How did you identify the schools from which to sample the children from? I see that you aimed for a heterogenous sample, but what criteria specifically did you use to select and approach these schools?

Response: We have clarified the criteria and process used to select and approach schools, ensuring that our sampling strategy achieved the intended heterogeneity of participants. ***Please see the lines: 157-162.***

Comment: How did you identify children and parents (of children) with excess screen use? How did you recruit them into the study? What were the age children were you sampling and why?

Response: We have clarified how children and their parents were identified based on excess screen use, detailed the recruitment process, and specified the age range of children sampled along with the rationale for these choices. ***Please see lines: 150-162.***

Comment: Were parents and children from the same families interviewed? How did you negotiate the ethics around this?

Response: We have clarified that parents and children were from the same families and have detailed how ethical considerations, including consent and confidentiality, were addressed in the study. **Please see lines: 223-235.**

Comment: Please ensure that you separate sampling and data collection procedures as this hinders the flow of the paper and makes for confusing reading. Data saturation has to do with the number of interviews and data collection, not sample size. Please review this section. We don't need to make a case for sample size when using purposive sampling, but rather focus on the eligibility criteria which guided your sampling of the participants.

Response: We have revised the manuscript to clearly separate sampling and data collection procedures, focused on eligibility criteria guiding purposive sampling, and clarified that data saturation relates to the number of interviews rather than sample size. ***Please see the lines: 150-186.***

Comment: You clarified the number of IDIs and FGDs you used, who they were with, where they were run, the language, the facilitators and how you developed the guides.

Response: We have revised the section to clearly separate sampling and data collection procedures, removed discussion of sample size, and focused on the eligibility criteria that guided our purposive sampling of participants. ***Please see the lines: 173-186.***

Comment: Please use references to support your processes?

Response: We have added appropriate references to support the processes used in our study. ***Please see the lines: 212-220.***

Comment: Please consolidate the data collection methods under one concise section, as this is not a streamlined passage.

Response: We have revised and concise the suggested point. ***Please see lines: 166-186.***

Comment: Methods seem appropriate for design. Would have been nice to see why these methods are useful to harness participants perspectives/experiences etc. or linked to the aim.

Response: We have clarified how the chosen methods are suited to capturing participants' perspectives and linked them explicitly to the study's aim.

Comment: 1.5 hrs appears very long for the children. What other strategies did you use to ensure that the data collection was rigorous for this specific age group? Looking at power differences etc. I am wondering how having the interviews at school could have influenced data collection as it is a place where children are safe, but not 'powerful'.

Response: We understand your concern. We have checked and clarified the strategies used to ensure rigorous data collection for this age group, including managing power dynamics, and have discussed how conducting interviews at school may have influenced the children's responses. Mentioned that each IDI lasted approximately 40-60 minutes and FGD up to maximum 90 minutes.

Comment: Please refer to the sources you used to guide this process? It appears that quantitative principles are being used to guide rigor strategies for qualitative work, which is confusing from a paradigm perspective and doesn't achieve its purpose.

Response: We have revised the manuscript to reference qualitative sources that guided our rigor strategies and have clarified the alignment of these strategies with qualitative research principles. ***Please see lines: 212-220.***

Comment: Rigor in data collection needs refinement, I would strongly urge you to refer to Lincoln and Guba's four constructs. This article refers to rigour in qualitative analysis specifically, however the principles can be applied to the project as a whole: International Journal of Qualitative Methods Thematic Analysis: Striving to Meet the Trustworthiness Criteria Volume 16: 1–13 DOI: 10.1177/160940691773384.

Response: We have refined the discussion of rigor in data collection and analysis by incorporating Lincoln and Guba's four constructs and have referenced the suggested article to strengthen the trustworthiness of our study. ***Please see lines: 212-220.***

Comment: Please refer to sources used to guide this process?

Response: We have added references to the sources that guided this process. ***Please see lines: 169.***

Comment: Outline the type of analysis used and the steps upfront (with reference).

Response: We have outlined the type of analysis used, detailed the steps upfront, and included appropriate references. ***Please see lines: 212-220.***

Comment: Clarify how you used the codebook and matrixes?

Response: We have clarified how the codebook and matrices were used to organize and analyze the data systematically. ***Please see lines: 166-186.***

Comment: No mention of reflexivity, this is vital in qualitative work to make your approach clear.

Response: We have added a section on reflexivity to clarify our approach and account for researcher influence in the study. ***Please see lines: 166-186.***

Comment: Were any specific ethical approaches needed as the study was working with children? How were these approached.

Response: We have detailed the specific ethical approaches used when working with children, including consent, assent, and safeguarding measures. ***Please see lines: 224-233.***

Comment: Add reimbursement to data collection for smooth reading

Response: We have included information on participant reimbursement to improve the flow of the manuscript. ***Please see lines: 233-235.***

Comment: It is not clear that table 1 only refers to student's characteristics. Why were characteristics of the other participants not reported?

Response: Thank you for the comment. We have clarified in the manuscript that Table 1 specifically presents the characteristics of the student participants only. The characteristics of parents and teachers were not reported in the table because the primary focus of this section was to describe the student sample, who were the main participants for exploring screen time behaviors. Demographic information for parents and teachers was summarized narratively in the Methods section to provide context without overloading the table, as their role was mainly to provide perspectives rather than be the primary subjects of analysis.

Comment: An initial overview of the themes related to challenges faced by parents needs to be provided. i.e. 4 themes were identified that consisted of…

Response: We have provided an initial overview of the themes related to challenges faced by parents, specifying the four main themes and their components. ***Please see lines: 260-432.***

Comment: It is expected that the information from the IDI and FGDs is synthesized as a part of thematic analysis, not presented separately, so this doesn't need to be explained.

Response: We have revised the manuscript to synthesize information from the IDIs and FGDs within the thematic analysis rather than presenting them separately.

Comment: Why are the recommendations' themes written as bullet points and do not follow the same process as the challenges with quotes to illustrate your point. The same structure should be used? These are still themes that you identified and need evidence. This section needs to be reworked to follow a similar structure and it would seem that some of the items fall under the same theme. I would consider using the ecological model to structure this section and place recommendations under the different levels as themes (i.e. micro, meso and macro) Please see this as an example: Harper CR, Steiner RJ, Brookmeyer KA. Using the Social-Ecological Model to Improve Access to Care for Adolescents and Young Adults. J Adolesc Health. 2018 Jun;62(6):641-642. doi: 10.1016/j.jadohealth.2018.03.010. PMID: 29784107; PMCID: PMC10898617.

Response: Thank you for this valuable suggestion. We have restructured the recommendations section to align with the format used for the challenges section, providing illustrative quotes from participants for each theme. Additionally, we have organized the recommendations using the social-ecological model, grouping them under micro (individual and family), meso (school and community), and macro (national policy) levels. This approach allows readers to clearly see the thematic structure, the supporting evidence from participants, and the relationships between different recommendations. By applying this framework, overlapping items were consolidated under broader themes, making the presentation more coherent and consistent with qualitative analysis standards. ***Please see lines: 433-513.***

Comment: General clarity, sentence structure, word usage and grammar needs reworking.

Response: We have carefully revised the manuscript to improve clarity, sentence structure, word usage, and grammar throughout.

Comment: Evidence/quote provided does not always match statement that it is linked to. (i.e. line 289)
Response: We have reviewed the manuscript and ensured that all evidence and quotes are correctly linked to the corresponding statements.

Comment: In the first section (challenges), children, parents and teachers often say the same thing. It would be more impactful for me if these topics are discussed with the evidence from the participants synthesised together, with only their unique contributions/differences discussed separately in another paragraph with evidence, rather than presenting each group of participants results separately under each heading.

Response: We have revised the challenges section to synthesize evidence from children, parents, and teachers, highlighting shared perspectives together and presenting unique contributions or differences separately with supporting evidence.

Comment: Why are some words in italics? Are these direct quotes? I would suggest using quote marks to make this clear.

Response: We have revised the manuscript to use quotation marks for all direct quotes and followed the journal formats.

Comment: General clarity, sentence structure, word usage and grammar needs reworking.

Response: We have thoroughly revised the manuscript to improve clarity, sentence structure, word usage, and grammar throughout.

Comment: This section needs reworking – rather than reporting what other evidence notes, you need to use this other research to link and make recommendations from your own research. It is fine to draw the similarities between your and other research as a mechanism to situate your evidence, but rather than report what other studies found, use it to build the discussion around your

evidence.

Response: We have reworked this section to use existing research to contextualize our findings and inform recommendations, rather than merely reporting other studies, ensuring the discussion is centered on our evidence.

Comment: Limitations:

- Why didn't you have enough time to do separate FGDs?
- The context of a study is not a limitation, rather provide a think description of the setting so that results are transferable to similar settings.
- Qualitative research does not aim to be generalisable and therefore this is not a limitation of the study

Response: Thank you for these comments. We have clarified the reasons for not conducting separate FGDs and have revised the limitations (removed the specific point) section to focus on relevant methodological considerations. We have also removed references to generalizability and contextual factors as limitations, providing a detailed description of the setting to enhance transferability instead.

Response to Reviewer 2

Comment: Check typos in line 77, 204.

Response: We have checked lines and addressed the issues accordingly.

Comment: Put phrases or acronyms in uniform, line 171 IDI so line 174,177 too.

Response: We have standardized the use of phrases and acronyms throughout the manuscript, including IDI and related terms.

Comment: Line 180-183, please have a look and rephrase.

Response: We have rephrased lines 180–183 for improved clarity and readability.

Comment: Please clarify the participants and the numbers of participants in line 160 and so forth.

Response: We have clarified the participants and specified the number of participants at line 160 and other relevant sections.

Comment: Line 216: clarify multiple sources. What are the three types of data collection tools

Response: We have clarified what is meant by multiple sources and specified the three types of data collection tools used in the study. ***Please see lines: 167-186.***

Comment: What is your measure to determine "excessive use of internet or screen view in this study?"

Response: We have clarified that "excessive use of internet or screen time" in this study was defined as more than 2 hours per day.

Response to Reviewer 3

Comment: This study tackles an important and relevant topic with valuable insights and practical recommendations that could significantly contribute to the field. The authors have conducted meaningful research that addresses real challenges faced by parents in managing adolescent screen use. However, the manuscript requires substantial methodological clarification and improved presentation of results to meet publication standards. The findings and recommendations are strong, but the research would benefit from more rigorous reporting and clearer methodology to enhance its impact and reproducibility.

Response: We sincerely thank the reviewer for recognizing the relevance and value of our study. We have revised the manuscript to provide clearer methodological details, improve the presentation of results, and enhance rigor and reproducibility, addressing the reviewer's suggestions to strengthen the overall impact of the research.

Comment: Article Summary: This qualitative study explores the challenges parents face in monitoring their adolescents' (aged 12-14) screen use in Dhaka, Bangladesh. Using focus group discussions, in-depth interviews, and key informant interviews with 25 children, parents, and teachers from six schools, the study identifies four main challenges: educational necessity of devices creating supervision difficulties, restricted outdoor activities, increasing device dependence, exposure to age-inappropriate content, and parental digital illiteracy. The study provides comprehensive recommendations including school-based interventions, national-level policies, enhanced parent-child bonding, and secured infrastructure development.

Response: We thank the reviewer for providing a clear summary of our study. We appreciate the acknowledgement of the challenges identified and the recommendations provided. We have ensured that the manuscript reflects these key findings accurately and comprehensively.

Comment: Comprehensive multi-stakeholder approach: The inclusion of children, parents, and teachers provides valuable triangulation of perspectives on screen use challenges

Response: We thank the reviewer for highlighting this strength. We have ensured that the manuscript emphasizes the value of including multiple stakeholders to provide a well-rounded understanding of challenges related to adolescent screen use.

Comment: Practical and actionable recommendations: The study offers concrete suggestions for schools, parents, and policymakers that are contextually relevant to the Bangladeshi setting

Response: We thank the reviewer for recognizing the practical relevance of our recommendations. We have ensured that these actionable suggestions for schools, parents, and policymakers are clearly presented and contextualized for the Bangladeshi setting.

Comment: Important and timely research topic: Addresses a critical public health concern regarding adolescent screen use in a rapidly digitizing society

Response: We thank the reviewer for acknowledging the importance and timeliness of our research. We have emphasized the public health significance of adolescent screen use in a rapidly digitizing society throughout the manuscript.

Comment: Rich qualitative data: The combination of FGDs, IDIs, and KIIs provides depth and breadth to understanding the phenomenon

Response: We thank the reviewer for recognizing the richness of our qualitative data. We have highlighted how the combination of FGDs and IDIs contributed to a comprehensive understanding of the challenges associated with adolescent screen use.

Comment: Cultural contextualization: Appropriately situates findings within the social and educational context of Dhaka, Bangladesh

Response: We thank the reviewer for highlighting this point. We have ensured that the findings are appropriately contextualized within the social and educational environment of Dhaka, Bangladesh.

Comment: Lack of clear operational definitions

Response: We have added clear operational definitions for all key terms in the manuscript. ***Please see lines: 188-204.***

Comment: Insufficient inclusion/exclusion criteria

Response: We have specified detailed inclusion and exclusion criteria for all participant groups, including age, technology use, and caregiver role. ***Please see lines: 151-162.***

Comment: Unclear data collection procedures

Response: We have clarified all data collection procedures, including interviews, focus groups, and key informant interviews, and the process for achieving thematic saturation. ***Please see lines: 166-186.***

Comment: Inadequate sample size justification

Response: We have clarified the rationale for the sample size, distinguishing between interviews and focus groups, and explained how saturation was determined. ***Please see lines: 151-162.***

Comment: Missing inter-rater reliability measures

Response: We have described the coding process and measures taken to ensure consistency and reliability between coders. ***Please see lines: 167-186.***

Comment: Incomplete demographic reporting.

Response: We have now provided relevant complete demographic details for all participant groups. Please see table 1 and 2.

Comment: Incomplete consent procedures

Response: We have clarified the consent and assent procedures, including ethical considerations for working with children. ***Please see lines: 223-235.***

Comment: Grammar issues

Response: We have thoroughly revised the manuscript to improve grammar, clarity, and sentence structure.

Comment: Presentation and organization

Response: We have reorganized sections for better flow, coherence, and readability, ensuring consistent structure throughout the manuscript.

Comment: Suggest using a grammar checker and proofreading – a lot of misspellings and run on sentences which make it hard to read the paper

Response: We have thoroughly proofread the manuscript and used grammar-checking tools to correct misspellings, run-on sentences, and improve overall readability.

Comment: Paragraph 1 - link between parenting and SES is established but it is unclear as to how parenting relates to health of adolescents ◊ explicitly state that parenting style is a mediator of technology use and thus, health of adolescents

Response: Parental monitoring, communication with adolescents and emotional care play a crucial role in shaping adolescents' use of technological devices and their related health outcomes. Additionally, positive parenting and strong parent-adolescent relationships are strongly linked to responsible technology use and improved mental health. Because inconsistent monitoring and supervision of parents can lead to higher risks of internet addiction, problematic usage of devices and adverse health outcomes, including anxiety, depression and attention-related problems.

Comment: Line 95 – please mention whether the screen time usage is a mean or median measure

Response: The median recreational screen time was found to be 4.0 hours/day; with boys having longer times (4.3 hours/day) compared to girls (3.6 hours/day).

Comment: Line 105 – author's mention AAP. The study context is Dhaka, Bangladesh, are there any Bangladeshi guidelines for pediatrics that should be mentioned? It will help strengthen the context and be directly applicable

Response: No. there is no Bangladeshi guideline for pediatrics which can be directly applicable.

Comment: Currently, paragraphs 1,2, 3 are not logically organized – suggestion for organization ◊ paragraph 1: clearly outline the problems on health brought forth by SV; paragraph 2: outline the literature for why parents are intentionally/unintentionally using technology to help with parenting; paragraph 3: outline existing pediatric guidelines and perspectives for healthier management of technology use among adolescents
Response: We have reorganized paragraphs 1–3 as suggested, clearly outlining the health problems related to screen viewing, literature on parental use of technology, and existing pediatric guidelines for managing adolescent technology use.

Comment: Line 160 – please quantify "excessive use" of digital devices using a threshold (for example, > 5 hours/day, etc.)

Response: We have quantified "excessive use" of digital devices as more than 2 hours per day in line 160.

Comment: Lines 160 – Lines 167 – please be specific with inclusion and exclusion criteria for the study. Please list the minimum age inclusion/exclusion for each participant, along with any other inclusion/exclusion criteria that was relevant to the study (e.g. technology use, language, family size, etc.) Please try to be specific in this section.

Response: Thank you for this comment. We have revised lines 160–167 to provide specific inclusion and exclusion criteria for all participant groups. Parents included were primary caregivers of children with excessive digital device use (>2 hours/day) who set rules regarding screen time. Adolescents included were boys and girls aged 12–14 years with excessive digital use (>2 hours/day). Teachers included were those experienced in managing students with high screen time, implementing school digital policies, and providing digital literacy education. Additional relevant criteria, such as language proficiency and engagement with technology, have also been specified.

Comment: Line 165 – while teacher's play a role in children's development and education, it is unclear as to how they would be valuable as key informants for the aim of the research as they are not providing child care; the introduction argues that SV has come on as a way of making caregiving easier. Please include some literature/citations that can help justify the inclusion of teachers as I do agree that they are an important perspective to consider.

Response: We have clarified and justified the inclusion of teachers as key informants. Teachers included were those with experience managing adolescents with excessive digital device use and implementing school policies on screen time. Their perspectives were valuable as they observe students' screen use in educational and social contexts and provide digital literacy education. We

have also added relevant literature to support the importance of including teachers in studies on adolescent screen use and caregiving dynamics.

Comment: Line 171 – please define how these "class register books" were obtained

Response: Thank you for pointing this out. We have clarified that adolescent students were purposively selected from the class register books, which were obtained with permission from the respective school authorities.

Comment: Regarding focus groups – were children and their parents both present in the same focus groups? This poses a concern for social desirability bias on the end of the children to appease to their parents. Please clarify if parents and children had separate focus groups (parents only and children only). If parents and children were present in the same focus group, please justify why this decision was made and how bias arising from potential social desirability bias was avoided.

Response: We have clarified that separate focus group discussions (FGDs) were conducted for children and parents, taking into account diverse classes, ages, and genders. This approach ensured a wide range of perspectives and experiences, minimized social desirability bias, and encouraged richer insights from participants.

Comment: Lines 180 – 184 – please condense language to reflect that interviews and focus groups were conducted until thematic saturation was reached.

Response: The point of saturation or point of redundancy was determined based on an "intellectual guiding principle" for deciding how many interviews to conduct in a qualitative study with purposive sampling as its basis. As mentioned, it is only an intellectual guiding principle with little or no on-the-ground guidance to sample size estimation that will be required to reach this saturation. To comprehend the challenges and recommendations for monitoring screen use of adolescents, a total of 18 interviews were conducted, including 12 IDIs and 6 FGD sessions till thematic saturation was achieved.

Comment: Line 185 – Please clarify the 18 interviews – I am only counting 12 interviews (5 students, 3 parents, and 4 key informant teacher interviews); please note that focus group discussions should not be included in the interview sample size and should be listed separately. E.g., "A total of 12 interviews and 6 FGDs were conducted." Additionally, please list the sample size, N for easier reading.

Response: To understand the challenges and recommendations for monitoring adolescent screen use, a total of 12 interviews (12 IDIs) and 6 FGDs were conducted until thematic saturation was achieved.

Comment: Table 1 – Please change the "School Types" column to be more reflective of categorization based on language of teaching as "school types" suggest classification based on funding – private v. public.

Response: Changed the "School Types" into "Medium of Instruction at Schools".

Comment: Line 191 – Please include/indicate how sociodemographic data was collected from participants.

Response: A section on sociodemographic-related questions was included in every guideline to gather the socio-demographic information of the participants.

Comment: Line 193 – Please include guidelines in the appendix. Alternatively, provide brief descriptions of the contents of the guidelines – did they include suggestions for how long interviews should last? Did they include any probes? How many questions were there? What themes did the interview/FGD questions explore, if any?

Response: Guidelines are included in the appendix, as suggested.

Comment: Line 197 – please update the sentence to state "three and five years of experience on qualitative data collection, respectively."

Response: We have updated the sentence. **Please see the lines: 166-186.**

Comment: Lines 200 – 203 – Which language were the interviews/FGDs conducted in? Were the interviews/FGD conducted in English at English-medium schools and in Bangla at Bangla-medium schools? Please clarify

Response: All interviews were carried out in Bangla, the national language of Bangladesh. **Please see the lines: 173-186.**

Comment: Line 201 – how long were key informant interviews?

Response: There was an understanding gap, and we have resolved this in revised version. **Please see the lines: 151-162.**

Comment: Line 208 – Please use gender neutral pronouns to describe the facilitator

Response: One researcher acted as the facilitator, and the other used the recorder to record the interviews, taking notes simultaneously. **Please see the lines: 166-186.**

Comment: Line 205 – Please clarify if the researcher with the recorder also took notes.

Response: One researcher acted as the facilitator, and the other used the recorder to record the interviews, taking notes simultaneously. **Please see the lines: 166-186.**

Comment: Lines 213- 218 – How was data validity maintained/triangulated? Were the same participants interviewed multiple times? Were parent – child dyads tracked for this triangulation? This section is very unclear – please provide more details regarding the methodology, maybe including a figure would be helpful.

Response: We have now provided a detailed explanation for it. **Please see lines: 206-220.**

Comment: Lines 219 – 220 – Data interpretation should be talked about in the analysis section, please remove this sentence.

Response: Removed

Comment: Line 221 – Please clarify validating data cross various contexts. Validity of data is usually not a concern in one-time interviews or focus group participations. There are usually limitations of self-report or social desirability biases, but it is unrealistic to have responses free of bias in qualitative studies. Who/what is this data being validated against. Is there a standard respondent? Alternatively, are the authors checking for quality of data, i.e., easy to comprehend and bug-free recordings or recordings that are easy to download/transcribe? Please clarify.

Response: Multiple supervisors and investigators were involved to minimise bias and provide a comprehensive interpretation of the data. The use of data and methodological triangulation enabled comprehensive analysis across different research sites and methods. Comparisons between key informants, parents, and students enhanced the validity of the findings. **Please see lines: 206-220.**

Comment: Line 226 – please provide an example file nomenclatures; e.g. "Participant001_English_IDI" or something along those lines that can let readers know of how files were stored in case they want to replicate.

Response: Thank you for the suggestion. We have now clarified the file nomenclature in the manuscript. For ease of organization and replication, all interview files were stored using a standardized naming convention that included the participant number, language of the interview, and interview type. For example: "Participant001_English_IDI" or "Participant012_Bangla_FGD". This approach ensured anonymity while allowing systematic identification and retrieval of files during analysis.

Comment: Line 228 – Earlier it is mentioned that interviews were recorded, but there is now a note implying that some interviews were not recorded. Please explain.

Response: Checked and edited accordingly. **Please see lines: 167-176.**

Comment: Line 231 – Please clarify if there were any consensus meetings to finalize the codebook. Consensus for codebook development is a foundational part of qualitative analysis. Additionally, please include the final codebook in the appendix as it will be helpful to other investigators conducting similar studies.

Response: The research team had a discussion on the developed codebook and finalized the codebook after addressing the critical review and feedback. **Please see lines: 167-186.**

Comment: Line 232– Please list the initials of those involved in the coding process (analysis team).

Response: The first stage of qualitative data analysis began by organising and preparing the field data, including interviews and field notes, by SHK and NS. **Please see lines: 206-220.**

Comment: Lines 236-238 – Rather than validity of data, I am more interested in the inter-rater reliability. Please clarify the exact coding methods – was a double-coding or single-coding method used. Or were a select few transcripts coded by all the analysis team members at the start to ensure consistency in coding before single-coding. Alternatively, did all the analysis team members code all the transcripts? Additionally, please provide Cohen's kappa for a measure of inter-rater reliability.

Response: Thank you for the comment. To clarify, we used a double-coding approach at the initial stage of analysis to ensure consistency and reliability in coding. This approach ensured rigor and consistency in our qualitative coding process. **Please see lines: 206-220.**

Comment: Line 241 – As children (<18) were involved in the study, please elaborate on their consent process. Was parental assent required?

Response: Revised ethical approval process *"The study protocol was approved by the Institutional Review Board (IRB) of icddr,b (Protocol No: PR-22002). Informed written consent was taken from each respondent (parents, teacher) and students were taken consent from their parents as well as assent was taken as their age was above 11 years prior to the interview. All the respondents were informed in Bengali about their rights related to their voluntary participation in the study and withdrew from the interview at any time during the interview. Interviewers of this study clearly conveyed to the respondents the objectives of the study, reasons for their participation in the study, their role, risks, benefits, anonymity and confidentiality, including the rights not to participate or withdraw. After getting acquainted with all these issues, the respondents decided whether to participate in this study. Confidentiality during and after data collection was ensured, and no identifiable information was used that may expose the identity of the participants. Only the research team members have access to the collected data, which were stored securely. After the interview, each participant received a gift (equivalent to BDT370 [USD3] as a token of appreciation for their valuable time."*

Comment: Line 249 – Where were the recordings/data stored? Please provide a location e.g. "password protected computer only accessible by the research team." Additionally, were the interviews themselves de-identified? Please clarify.

Response: Confidentiality during and after data collection was ensured, and no identifiable information was used that may expose the identity of the participants. Only the research team members have access to the collected data, which were stored securely in the password-protected central data storage of icddr,b.

Comment: Line 250 – Please specify the token of appreciation amount (in Bangladeshi currency and USD).

Response: We have specified the token of appreciation amount, provided in both Bangladeshi Taka and its equivalent in USD. **Please see lines: 219-220 as follows "*After the interview, each***

participant received a gift (equivalent to BDT370 [USD3] as a token of appreciation for their valuable time."

Comment: Line 253 – Please note that Table 2 only provides the demographics for children who participated in the study (from what I gather). Please update language to reflective of this specific demographic. As parents and teachers were also interviewed/included in the study, please add additional tables for demographic characteristics of each of these groups. Alternatively, you can create one large table and have subgroup frequencies included as columns (column 1 = children, column 2 = parents, column 3 = teachers)

Response: Thank you for this comment. We have clarified in the manuscript that Table 2 presents the demographic characteristics of the child participants only. To provide a complete overview of all study participants, we have added additional tables reporting the demographics of parents and teachers. Alternatively, we have also considered creating a combined table with subgroup frequencies in separate columns (children, parents, teachers) to allow easy comparison across participant groups. This ensures transparency and allows readers to understand the composition of all participants included in the study.

Comment: Line 263 – 268 – please provide your definition of screen viewing and then provide a summary of responses/content analysis by participant subgroup that relate to screen viewing as a concept. To strengthen this paragraph, you can also summarize any quotes or general remarks that point towards relations between screen viewing and health.

Response: We have provided a clear definition of screen viewing and summarized responses by participant subgroup. We have also included quotes and remarks highlighting the relationships between screen viewing and health to strengthen this section.

Comment: Lines 325 – 332 – please provide more context using literature or another source to contextualize the social climate in Dhaka.

Response: Revised the text: Dhaka, a densely populated megacity, has limited open and child-friendly spaces, restricting children's opportunities for outdoor play. Concerns over traffic, pollution, and safety further confine children indoors. At the same time, rapid expansion of internet and smartphone access has made screen-based activities more available and attractive. Together, these factors create a social climate that favors indoor, screen-based leisure—an important context for interpreting participants' views in this study. **Please see lines: 76-90.**

Comment: Lines 394 – 403 – please provide an exemplary quote to support the claim being made. It is interesting and I would like to read what was said.

Response: We have added an exemplary quote from participants to support the claim and provide greater insight into their perspectives.

Comment: Line 410 – please check the formatting of the quote.

Response: Tanks. Formatted accordingly

Comment: Line 431 – please include a transition paragraph that segways into the new subsection of recommendations. Also, please consider condensing these recommendations and moving them to the discussion section or including them as a table. Great work on the recommendations!

Response: The study participants did not limit themselves to sharing only the challenges that they faced with morning screen time but also provided some contextualized recommendations to address these challenges. **Please see lines: 433-513 and entire discussion part.**

Comment: Line 484 – Please rename the subheading to "ensure social safety"

Response: Changed to "Ensuring social security"

Comment: General note – I am missing quotes or analysis based on SES status as that seemed to be a big factor influencing health of adolescents and technology use according to the introduction written by the author's. Please include 1 or 2 quotes that highlight any relationships of SES, technology use, and health/concerns.

Response: Thank you for this important observation. We have now incorporated participants' perspectives highlighting the influence of socioeconomic status (SES) on screen use and related health concerns. For instance, one parent noted: "Families with higher income often provide multiple devices to their children, which makes it harder to control screen time and can affect their sleep and concentration." Similarly, a student mentioned: "Some of my friends don't have personal devices at home, so they play outside more, and I think they stay healthier than those who spend all day on phones or tablets." These quotes illustrate how SES shapes access to technology and, consequently, children's screen habits and associated health outcomes. ***Please see lines: 369-378.***

Comment: Lines 533 – 537 – I appreciate the domains/points brought up regarding SV (e.g. not all screens are equal). It would potentially be helpful to include a table in the results section listing exemplary quotes organized by these domains for easier comprehension.

Response: Thank you for this comment. We tried to incorporate in text.

Comment: Line 567 – please elaborate. The paragraph is only one sentence currently.

Response: We have elaborated on the paragraph at line 567, expanding it to provide greater detail and context.

Comment: Line 583 – what were the results of the clustered randomized controlled trial?

Response: Here is the result: Total physical activity (PA), measured in MET-min/week, increased from baseline to 8 and 12 weeks in the intervention group (IG) by 3%–5%, whereas it decreased in the control group (CG) by 5%–3%. Over time, PA improved significantly in the IG compared to the CG ($p < 0.001$). Average sedentary time (ST) in minutes per day decreased from baseline to 8 and 12 weeks in the IG by 28%–35%, while remaining largely unchanged in the CG (6%–5%). At 12 weeks, the IG had a significantly lower average ST than the CG. Additionally, average

knowledge scores on PA and sedentary behavior (SB) were significantly higher in the IG than in the CG at 12 weeks ($p < 0.001$).

Comment: Line 608 – I like the call to action being presented here. To tighten narrative, please tie it in or include how existing pediatric guidelines could be modified. Alternatively, should we consider devising new pediatric guidelines? Curious to hear your thoughts.

Response: We have tied the call to action to existing pediatric guidelines, discussing potential modifications and the possibility of developing new guidelines to better address adolescent screen use in the current context.

Comment: Under limitations, please also include the influence of social desirability biases among participants or potential selection for responses given the skew towards Bangla-medium participants in the study. Additionally, please include limitations related to potential human error (such as not recording interviews, errors in translating from Bangla to English or vice versa).

Response: Thanks for this comment and suggestion. We have reviewed our manuscript carefully and removed the limitation points. Since it's a qualitative study and there is no generalizations issue like quantitative study, we have removed the section.

Sincerely
Dr. Shahria Hafiz Kakon
On behalf of Nahian Sultana,Bidhan Krishna Sarker, Tanjir Rashid Soron, Rashidul Hoque, Fahmida Tofail
