# Supplementary material for: Parental monitoring challenges and recommendation for managing excessive screen use among students aged 12–14 years in Dhaka, Bangladesh: A qualitative exploration
Source: PLoS One. 2026 Oct 5;21(10):e0359518. doi: 10.1371/journal.pone.0359518 (PMC13637862; doi:10.1371/journal.pone.0359518)
Supplement: Supplementary file 3 [file pone.0359518.s005.pdf]

September 29, 2025

To
Derebe Madoro Bunte
Academic Editor
PLOS ONE

Subject: Responses to Editor and Reviewer comments on the manuscript [PONE-D-25-02782] - [EMID:bd74e89f4f1ba54a]

Dear Editor,

Thank you for allowing us the opportunity to improve and resubmit our manuscript titled "**Parental monitoring challenges and recommendation for managing excessive screen use among students aged 12–14 years in Dhaka, Bangladesh: a qualitative exploration**". We would like to extend our gratitude to the editor and reviewers for their valuable feedback and comments on our research manuscript. We have carefully reviewed all the comments and have addressed them individually. Additionally, we have incorporated the suggested changes into the manuscript where applicable. Below, please finds a detailed account of our responses to the comments from the editor and reviewers.

<u>Response to Editor's Comments:</u>

Comment: Please ensure that your manuscript meets PLOS ONE's style requirements, including those for file naming. The PLOS ONE style templates can be found at
https://journals.plos.org/plosone/s/file?id=wjVg/PLOSOne_formatting_sample_main_body.pdf
and
https://journals.plos.org/plosone/s/file?id=ba62/PLOSOne_formatting_sample_title_authors_affiliations.pdf

Response: Thank you for the suggestion and provide the link. As suggested and following the links, we have revised the manuscript.

Comment: You indicated that you had ethical approval for your study. In your Methods section, please ensure you have also stated whether you obtained consent from parents or guardians of the minors included in the study or whether the research ethics committee or IRB specifically waived the need for their consent.

Response: Many thanks for this comment. We have obtained written consent from each participant and mentioned it in the ethical approval section. ***Please see Lines: 223-235.***

Comment: We note that the grant information you provided in the 'Funding Information' and 'Financial Disclosure' sections do not match.

Response: Thank you for pointing this out. We apologize for the inconsistency. We have carefully reviewed the grant details and updated the Funding Information and Financial Disclosure sections to ensure they are consistent and accurately reflect the source of funding.

Comment: When you resubmit, please ensure that you provide the correct grant numbers for the awards you received for your study in the 'Funding Information' section.

Response: We have verified the grant details and ensured that the correct grant numbers are provided in the Funding Information section.

Comment:
Thank you for stating the following financial disclosure: "Bangladesh government awarded project named Mujib 100 research Grant for women. I have received this award in 2022. Funded was $ 43,250 for 2 years project"

Please state what role the funders took in the study. If the funders had no role, please state: "The funders had no role in study design, data collection and analysis, decision to publish, or preparation of the manuscript."

If this statement is not correct you must amend it as needed.

Please include this amended Role of Funder statement in your cover letter; we will change the online submission form on your behalf.

Response: Thank you for your comment. The funders had no role in study design, data collection and analysis, decision to publish, or preparation of the manuscript. We have included this amended *Role of Funder* statement in the cover letter as suggested. Thanks once again for your kind support in making the change in the online system.

Comment: Please note that funding information should not appear in any section or other areas of your manuscript. We will only publish funding information present in the Funding Statement section of the online submission form. Please remove any funding-related text from the manuscript.

Response: Thank you for this clarification. We have removed all funding-related text from the manuscript.

Comment: Please provide a complete Data Availability Statement in the submission form, ensuring you include all necessary access information or a reason for why you are unable to make your data freely accessible. If your research concerns only data provided within your submission, please write "All data are in the manuscript and/or supporting information files" as your Data Availability Statement.

Response: Thank you for your guidance. We have revised the submission form and included a complete Data Availability Statement.

Comment: When completing the data availability statement of the submission form, you indicated that you would make your data available on acceptance. We strongly recommend all authors decide on a data sharing plan before acceptance, as the process can be lengthy and hold up publication timelines. Please note that, though access restrictions are acceptable now, your entire data will need to be made freely accessible if your manuscript is accepted for publication. This policy applies to all data except where public deposits would breach compliance with the protocol approved by your research ethics board. If you are unable to adhere to our open data policy, please kindly revise your statement to explain your reasoning and we will seek the editor's input on an exemption. Please be assured that once you have provided your new statement, the assessment of your exemption will not hold up the peer review process.

Response: Thank you for your advice regarding the data availability statement. We understand and acknowledge the journal's open data policy. However, our data cannot be made freely accessible due to icddr,b's data sharing policy. Accordingly, we have revised our Data Availability Statement to clarify this restriction and have noted that the data will be available upon reasonable request through icddr,b's Research Administration.

Comment: Please include your full ethics statement in the 'Methods' section of your manuscript file. In your statement, please include the full name of the IRB or ethics committee who approved or waived your study, as well as whether or not you obtained informed written or verbal consent. If consent was waived for your study, please include this information in your statement as well.

Response: Thank you for your guidance. We have revised the Methods section of the manuscript to include the full ethics statement. Specifically, we have added the name of the IRB that approved the study and clarified that informed written was obtained.

Comment: If the reviewer comments include a recommendation to cite specific previously published works, please review and evaluate these publications to determine whether they are relevant and should be cited. There is no requirement to cite these works unless the editor has indicated otherwise.

Response: Thank you for the clarification. We have carefully reviewed the suggested publications and evaluated their relevance to our study. We have cited those that are directly relevant and have not included those that are not applicable, in line with your guidance.

Additional Editor Comments:

- The manuscript requires substantial revision before acceptance, with mandatory changes including clarification of study design and methodology (operational definitions, inclusion/exclusion criteria, recruitment procedures, role of teachers, separation of FGDs and IDIs, complete demographic reporting), detailed ethical procedures, and rigorous description of the analysis process with supporting quotes for all themes. Recommended (non-essential) changes include restructuring recommendations using an ecological model, adding SES-linked analysis, presenting exemplary quotes in tables, and improving contextual relevance with local guidelines. While all reviewers agree on the topic's importance and the value of the findings, they consistently highlight gaps in methodological transparency, thematic presentation, and language clarity, with no direct

conflicts—thus authors should follow the converging advice to strengthen rigor, synthesis, and readability.

Response: We thank the editor and reviewers for their constructive feedback. We have revised the manuscript to clarify the study design and methodology, including operational definitions, inclusion/exclusion criteria, recruitment procedures, and the role of teachers; separated FGDs and IDIs; provided complete demographic reporting; detailed ethical procedures; and strengthened the analysis with supporting quotes for all themes. Additionally, we have addressed recommended suggestions by restructuring recommendations using an ecological model, adding SES-linked analysis, presenting exemplary quotes in tables, and improving contextual relevance. We believe these revisions enhance the manuscript's clarity, rigor, and readability.

Response to Reviewer 1

Comment: Thank you for your submission. Overall, this paper addresses what seems to be a key concern. The paper appears to do what it aimed to do, but the portrayal of the results and discussion hinders its impact, and the language and construction of the paper hinders readability. Literature needs to be further synthesized and used effectively to draw conclusions and develop arguments in introduction and discussion. With reworking and refining concerns, this could be a publishable paper.

Response: Thank you for the thoughtful feedback and recognition of the paper's importance. We have revised the manuscript to improve clarity and readability, refine the presentation of results and discussion, and better synthesize and integrate the literature to strengthen arguments and conclusions.

Comment: Grammar and word use not always appropriate for an academic article or unclear. (Lines 67, 74, 83, 84, 85, 86, 89, 101, 104, 112, 115) Overall the introduction could improve in flow, conciseness and effectiveness.

Response: Thank you for pointing this out. We have revised the manuscript thoroughly including the identified lines to correct grammar and word use, and improve the overall flow, conciseness, and effectiveness of the introduction.

Comment: The review of literature felt like a side-by-side review of the different studies, rather than synthesizing the information into specific sections to build into an argument to justify this paper. Justification for this study and the gap in literature was not fully developed in the introduction, therefore difficult to ascertain. Although there was a description of limited data on the topic, this was not argued so difficult to ascertain.

Response: Thank you for this valuable comment. We have synthesized the literature thematically, highlight the research gap more clearly, and strengthen the justification for the study in the introduction. ***Please see the lines: 54-119.***

Comment: I would suggest that this section is reworked to synthesise the evidence to make a stronger case for the evidence gap and justification for this study.

Response: We reworked the section to synthesize the evidence more effectively, highlight the gap, and present a stronger justification for this study. ***Please see the lines: 54-119.***

Comment: Remember we don't examine in qualitative work, but rather explore or understand, describe etc.

Response: We revised the wording to use more appropriate terms such as "explore," and "understand," in line with qualitative research conventions.

Comment: 'gather specific feedback on how to effectively utilize SV within urban Bangladesh context' doesn't make sense - for adolescent use? to create a family plan for SV? The second statement in the methods section makes much more sense.

Response: We have revised the statement for clarity to better reflect the intended meaning and have ensured consistency with the methods section. ***Please see the lines: 116-119.***

Comment: A cross-sectional design is linked to quantitative work, not qual. This appears to be a descriptive qualitative study. Please use references.

Response: We have clarified that this is a descriptive qualitative study and have updated the manuscript to reflect appropriate references supporting this approach. ***Please see the lines: 123-124.***

Comment: Please provide a description of the setting (i.e. North and South Dhaka City Corporations in Bangladesh) so that we have an understanding of the context which is vital for rigour, specifically transferability. Sampling and recruitment: Clear justification for why specific participants were chosen and heterogenous sampling was used. However, your recruitment strategy was unclear and the eligibility criteria was not apparent.

Response: Thank you for this detailed comment. We have added a clear description of the study setting, including North and South Dhaka City Corporations, to provide context and enhance transferability. We have also clarified our recruitment strategy, eligibility criteria, and the rationale for using heterogeneous sampling. ***Please see the lines: 137-148.***

Comment: How did you identify the schools from which to sample the children from? I see that you aimed for a heterogenous sample, but what criteria specifically did you use to select and approach these schools?

Response: We have clarified the criteria and process used to select and approach schools, ensuring that our sampling strategy achieved the intended heterogeneity of participants. ***Please see the lines: 157-162.***

Comment: How did you identify children and parents (of children) with excess screen use? How did you recruit them into the study? What were the age children were you sampling and why?

Response: We have clarified how children and their parents were identified based on excess screen use, detailed the recruitment process, and specified the age range of children sampled along with the rationale for these choices. ***Please see lines: 150-162.***

Comment: Were parents and children from the same families interviewed? How did you negotiate the ethics around this?

Response: We have clarified that parents and children were from the same families and have detailed how ethical considerations, including consent and confidentiality, were addressed in the study. **Please see lines: 223-235.**

Comment: Please ensure that you separate sampling and data collection procedures as this hinders the flow of the paper and makes for confusing reading. Data saturation has to do with the number of interviews and data collection, not sample size. Please review this section. We don't need to make a case for sample size when using purposive sampling, but rather focus on the eligibility criteria which guided your sampling of the participants.

Response: We have revised the manuscript to clearly separate sampling and data collection procedures, focused on eligibility criteria guiding purposive sampling, and clarified that data saturation relates to the number of interviews rather than sample size. ***Please see the lines: 150-186.***

Comment: You clarified the number of IDIs and FGDs you used, who they were with, where they were run, the language, the facilitators and how you developed the guides.

Response: We have revised the section to clearly separate sampling and data collection procedures, removed discussion of sample size, and focused on the eligibility criteria that guided our purposive sampling of participants. ***Please see the lines: 173-186.***

Comment: Please use references to support your processes?

Response: We have added appropriate references to support the processes used in our study. ***Please see the lines: 212-220.***

Comment: Please consolidate the data collection methods under one concise section, as this is not a streamlined passage.

Response: We have revised and concise the suggested point. ***Please see lines: 166-186.***

Comment: Methods seem appropriate for design. Would have been nice to see why these methods are useful to harness participants perspectives/experiences etc. or linked to the aim.

Response: We have clarified how the chosen methods are suited to capturing participants' perspectives and linked them explicitly to the study's aim.

Comment: 1.5 hrs appears very long for the children. What other strategies did you use to ensure that the data collection was rigorous for this specific age group? Looking at power differences etc. I am wondering how having the interviews at school could have influenced data collection as it is a place where children are safe, but not 'powerful'.

Response: We understand your concern. We have checked and clarified the strategies used to ensure rigorous data collection for this age group, including managing power dynamics, and have discussed how conducting interviews at school may have influenced the children's responses. Mentioned that each IDI lasted approximately 40-60 minutes and FGD up to maximum 90 minutes.

Comment: Please refer to the sources you used to guide this process? It appears that quantitative principles are being used to guide rigor strategies for qualitative work, which is confusing from a paradigm perspective and doesn't achieve its purpose.

Response: We have revised the manuscript to reference qualitative sources that guided our rigor strategies and have clarified the alignment of these strategies with qualitative research principles. ***Please see lines: 212-220.***

Comment: Rigor in data collection needs refinement, I would strongly urge you to refer to Lincoln and Guba's four constructs. This article refers to rigour in qualitative analysis specifically, however the principles can be applied to the project as a whole: International Journal of Qualitative Methods Thematic Analysis: Striving to Meet the Trustworthiness Criteria Volume 16: 1–13 DOI: 10.1177/160940691773384.

Response: We have refined the discussion of rigor in data collection and analysis by incorporating Lincoln and Guba's four constructs and have referenced the suggested article to strengthen the trustworthiness of our study. ***Please see lines: 212-220.***

Comment: Please refer to sources used to guide this process?

Response: We have added references to the sources that guided this process. ***Please see lines: 169.***

Comment: Outline the type of analysis used and the steps upfront (with reference).

Response: We have outlined the type of analysis used, detailed the steps upfront, and included appropriate references. ***Please see lines: 212-220.***

Comment: Clarify how you used the codebook and matrixes?

Response: We have clarified how the codebook and matrices were used to organize and analyze the data systematically. ***Please see lines: 166-186.***

Comment: No mention of reflexivity, this is vital in qualitative work to make your approach clear.

Response: We have added a section on reflexivity to clarify our approach and account for researcher influence in the study. ***Please see lines: 166-186.***

Comment: Were any specific ethical approaches needed as the study was working with children? How were these approached.

Response: We have detailed the specific ethical approaches used when working with children, including consent, assent, and safeguarding measures. ***Please see lines: 224-233.***

Comment: Add reimbursement to data collection for smooth reading

Response: We have included information on participant reimbursement to improve the flow of the manuscript. ***Please see lines: 233-235.***

Comment: It is not clear that table 1 only refers to student's characteristics. Why were characteristics of the other participants not reported?

Response: Thank you for the comment. We have clarified in the manuscript that Table 1 specifically presents the characteristics of the student participants only. The characteristics of parents and teachers were not reported in the table because the primary focus of this section was to describe the student sample, who were the main participants for exploring screen time behaviors. Demographic information for parents and teachers was summarized narratively in the Methods section to provide context without overloading the table, as their role was mainly to provide perspectives rather than be the primary subjects of analysis.

Comment: An initial overview of the themes related to challenges faced by parents needs to be provided. i.e. 4 themes were identified that consisted of…

Response: We have provided an initial overview of the themes related to challenges faced by parents, specifying the four main themes and their components. ***Please see lines: 260-432.***

Comment: It is expected that the information from the IDI and FGDs is synthesized as a part of thematic analysis, not presented separately, so this doesn't need to be explained.

Response: We have revised the manuscript to synthesize information from the IDIs and FGDs within the thematic analysis rather than presenting them separately.

Comment: Why are the recommendations' themes written as bullet points and do not follow the same process as the challenges with quotes to illustrate your point. The same structure should be used? These are still themes that you identified and need evidence. This section needs to be reworked to follow a similar structure and it would seem that some of the items fall under the same theme. I would consider using the ecological model to structure this section and place recommendations under the different levels as themes (i.e. micro, meso and macro) Please see this as an example: Harper CR, Steiner RJ, Brookmeyer KA. Using the Social-Ecological Model to Improve Access to Care for Adolescents and Young Adults. J Adolesc Health. 2018 Jun;62(6):641-642. doi: 10.1016/j.jadohealth.2018.03.010. PMID: 29784107; PMCID: PMC10898617.

Response: Thank you for this valuable suggestion. We have restructured the recommendations section to align with the format used for the challenges section, providing illustrative quotes from participants for each theme. Additionally, we have organized the recommendations using the social-ecological model, grouping them under micro (individual and family), meso (school and community), and macro (national policy) levels. This approach allows readers to clearly see the thematic structure, the supporting evidence from participants, and the relationships between different recommendations. By applying this framework, overlapping items were consolidated under broader themes, making the presentation more coherent and consistent with qualitative analysis standards. ***Please see lines: 433-513.***

Comment: General clarity, sentence structure, word usage and grammar needs reworking.

Response: We have carefully revised the manuscript to improve clarity, sentence structure, word usage, and grammar throughout.

Comment: Evidence/quote provided does not always match statement that it is linked to. (i.e. line 289)
Response: We have reviewed the manuscript and ensured that all evidence and quotes are correctly linked to the corresponding statements.

Comment: In the first section (challenges), children, parents and teachers often say the same thing. It would be more impactful for me if these topics are discussed with the evidence from the participants synthesised together, with only their unique contributions/differences discussed separately in another paragraph with evidence, rather than presenting each group of participants results separately under each heading.

Response: We have revised the challenges section to synthesize evidence from children, parents, and teachers, highlighting shared perspectives together and presenting unique contributions or differences separately with supporting evidence.

Comment: Why are some words in italics? Are these direct quotes? I would suggest using quote marks to make this clear.

Response: We have revised the manuscript to use quotation marks for all direct quotes and followed the journal formats.

Comment: General clarity, sentence structure, word usage and grammar needs reworking.

Response: We have thoroughly revised the manuscript to improve clarity, sentence structure, word usage, and grammar throughout.

Comment: This section needs reworking – rather than reporting what other evidence notes, you need to use this other research to link and make recommendations from your own research. It is fine to draw the similarities between your and other research as a mechanism to situate your evidence, but rather than report what other studies found, use it to build the discussion around your

evidence.

Response: We have reworked this section to use existing research to contextualize our findings and inform recommendations, rather than merely reporting other studies, ensuring the discussion is centered on our evidence.

Comment: Limitations:

- Why didn't you have enough time to do separate FGDs?
- The context of a study is not a limitation, rather provide a think description of the setting so that results are transferable to similar settings.
- Qualitative research does not aim to be generalisable and therefore this is not a limitation of the study

Response: Thank you for these comments. We have clarified the reasons for not conducting separate FGDs and have revised the limitations (removed the specific point) section to focus on relevant methodological considerations. We have also removed references to generalizability and contextual factors as limitations, providing a detailed description of the setting to enhance transferability instead.

Response to Reviewer 2

Comment: Check typos in line 77, 204.

Response: We have checked lines and addressed the issues accordingly.

Comment: Put phrases or acronyms in uniform, line 171 IDI so line 174,177 too.

Response: We have standardized the use of phrases and acronyms throughout the manuscript, including IDI and related terms.

Comment: Line 180-183, please have a look and rephrase.

Response: We have rephrased lines 180–183 for improved clarity and readability.

Comment: Please clarify the participants and the numbers of participants in line 160 and so forth.

Response: We have clarified the participants and specified the number of participants at line 160 and other relevant sections.

Comment: Line 216: clarify multiple sources. What are the three types of data collection tools

Response: We have clarified what is meant by multiple sources and specified the three types of data collection tools used in the study. ***Please see lines: 167-186.***

Comment: What is your measure to determine "excessive use of internet or screen view in this study?"

Response: We have clarified that "excessive use of internet or screen time" in this study was defined as more than 2 hours per day.

Response to Reviewer 3

Comment: This study tackles an important and relevant topic with valuable insights and practical recommendations that could significantly contribute to the field. The authors have conducted meaningful research that addresses real challenges faced by parents in managing adolescent screen use. However, the manuscript requires substantial methodological clarification and improved presentation of results to meet publication standards. The findings and recommendations are strong, but the research would benefit from more rigorous reporting and clearer methodology to enhance its impact and reproducibility.

Response: We sincerely thank the reviewer for recognizing the relevance and value of our study. We have revised the manuscript to provide clearer methodological details, improve the presentation of results, and enhance rigor and reproducibility, addressing the reviewer's suggestions to strengthen the overall impact of the research.

Comment: Article Summary: This qualitative study explores the challenges parents face in monitoring their adolescents' (aged 12-14) screen use in Dhaka, Bangladesh. Using focus group discussions, in-depth interviews, and key informant interviews with 25 children, parents, and teachers from six schools, the study identifies four main challenges: educational necessity of devices creating supervision difficulties, restricted outdoor activities, increasing device dependence, exposure to age-inappropriate content, and parental digital illiteracy. The study provides comprehensive recommendations including school-based interventions, national-level policies, enhanced parent-child bonding, and secured infrastructure development.

Response: We thank the reviewer for providing a clear summary of our study. We appreciate the acknowledgement of the challenges identified and the recommendations provided. We have ensured that the manuscript reflects these key findings accurately and comprehensively.

Comment: Comprehensive multi-stakeholder approach: The inclusion of children, parents, and teachers provides valuable triangulation of perspectives on screen use challenges

Response: We thank the reviewer for highlighting this strength. We have ensured that the manuscript emphasizes the value of including multiple stakeholders to provide a well-rounded understanding of challenges related to adolescent screen use.

Comment: Practical and actionable recommendations: The study offers concrete suggestions for schools, parents, and policymakers that are contextually relevant to the Bangladeshi setting

Response: We thank the reviewer for recognizing the practical relevance of our recommendations. We have ensured that these actionable suggestions for schools, parents, and policymakers are clearly presented and contextualized for the Bangladeshi setting.

Comment: Important and timely research topic: Addresses a critical public health concern regarding adolescent screen use in a rapidly digitizing society

Response: We thank the reviewer for acknowledging the importance and timeliness of our research. We have emphasized the public health significance of adolescent screen use in a rapidly digitizing society throughout the manuscript.

Comment: Rich qualitative data: The combination of FGDs, IDIs, and KIIs provides depth and breadth to understanding the phenomenon

Response: We thank the reviewer for recognizing the richness of our qualitative data. We have highlighted how the combination of FGDs and IDIs contributed to a comprehensive understanding of the challenges associated with adolescent screen use.

Comment: Cultural contextualization: Appropriately situates findings within the social and educational context of Dhaka, Bangladesh

Response: We thank the reviewer for highlighting this point. We have ensured that the findings are appropriately contextualized within the social and educational environment of Dhaka, Bangladesh.

Comment: Lack of clear operational definitions

Response: We have added clear operational definitions for all key terms in the manuscript. ***Please see lines: 188-204.***

Comment: Insufficient inclusion/exclusion criteria

Response: We have specified detailed inclusion and exclusion criteria for all participant groups, including age, technology use, and caregiver role. ***Please see lines: 151-162.***

Comment: Unclear data collection procedures

Response: We have clarified all data collection procedures, including interviews, focus groups, and key informant interviews, and the process for achieving thematic saturation. ***Please see lines: 166-186.***

Comment: Inadequate sample size justification

Response: We have clarified the rationale for the sample size, distinguishing between interviews and focus groups, and explained how saturation was determined. ***Please see lines: 151-162.***

Comment: Missing inter-rater reliability measures

Response: We have described the coding process and measures taken to ensure consistency and reliability between coders. ***Please see lines: 167-186.***

Comment: Incomplete demographic reporting.

Response: We have now provided relevant complete demographic details for all participant groups. Please see table 1 and 2.

Comment: Incomplete consent procedures

Response: We have clarified the consent and assent procedures, including ethical considerations for working with children. ***Please see lines: 223-235.***

Comment: Grammar issues

Response: We have thoroughly revised the manuscript to improve grammar, clarity, and sentence structure.

Comment: Presentation and organization

Response: We have reorganized sections for better flow, coherence, and readability, ensuring consistent structure throughout the manuscript.

Comment: Suggest using a grammar checker and proofreading – a lot of misspellings and run on sentences which make it hard to read the paper

Response: We have thoroughly proofread the manuscript and used grammar-checking tools to correct misspellings, run-on sentences, and improve overall readability.

Comment: Paragraph 1 - link between parenting and SES is established but it is unclear as to how parenting relates to health of adolescents ◊ explicitly state that parenting style is a mediator of technology use and thus, health of adolescents

Response: Parental monitoring, communication with adolescents and emotional care play a crucial role in shaping adolescents' use of technological devices and their related health outcomes. Additionally, positive parenting and strong parent-adolescent relationships are strongly linked to responsible technology use and improved mental health. Because inconsistent monitoring and supervision of parents can lead to higher risks of internet addiction, problematic usage of devices and adverse health outcomes, including anxiety, depression and attention-related problems.

Comment: Line 95 – please mention whether the screen time usage is a mean or median measure

Response: The median recreational screen time was found to be 4.0 hours/day; with boys having longer times (4.3 hours/day) compared to girls (3.6 hours/day).

Comment: Line 105 – author's mention AAP. The study context is Dhaka, Bangladesh, are there any Bangladeshi guidelines for pediatrics that should be mentioned? It will help strengthen the context and be directly applicable

Response: No. there is no Bangladeshi guideline for pediatrics which can be directly applicable.

Comment: Currently, paragraphs 1,2, 3 are not logically organized – suggestion for organization ◊ paragraph 1: clearly outline the problems on health brought forth by SV; paragraph 2: outline the literature for why parents are intentionally/unintentionally using technology to help with parenting; paragraph 3: outline existing pediatric guidelines and perspectives for healthier management of technology use among adolescents
Response: We have reorganized paragraphs 1–3 as suggested, clearly outlining the health problems related to screen viewing, literature on parental use of technology, and existing pediatric guidelines for managing adolescent technology use.

Comment: Line 160 – please quantify "excessive use" of digital devices using a threshold (for example, > 5 hours/day, etc.)

Response: We have quantified "excessive use" of digital devices as more than 2 hours per day in line 160.

Comment: Lines 160 – Lines 167 – please be specific with inclusion and exclusion criteria for the study. Please list the minimum age inclusion/exclusion for each participant, along with any other inclusion/exclusion criteria that was relevant to the study (e.g. technology use, language, family size, etc.) Please try to be specific in this section.

Response: Thank you for this comment. We have revised lines 160–167 to provide specific inclusion and exclusion criteria for all participant groups. Parents included were primary caregivers of children with excessive digital device use (>2 hours/day) who set rules regarding screen time. Adolescents included were boys and girls aged 12–14 years with excessive digital use (>2 hours/day). Teachers included were those experienced in managing students with high screen time, implementing school digital policies, and providing digital literacy education. Additional relevant criteria, such as language proficiency and engagement with technology, have also been specified.

Comment: Line 165 – while teacher's play a role in children's development and education, it is unclear as to how they would be valuable as key informants for the aim of the research as they are not providing child care; the introduction argues that SV has come on as a way of making caregiving easier. Please include some literature/citations that can help justify the inclusion of teachers as I do agree that they are an important perspective to consider.

Response: We have clarified and justified the inclusion of teachers as key informants. Teachers included were those with experience managing adolescents with excessive digital device use and implementing school policies on screen time. Their perspectives were valuable as they observe students' screen use in educational and social contexts and provide digital literacy education. We

have also added relevant literature to support the importance of including teachers in studies on adolescent screen use and caregiving dynamics.

Comment: Line 171 – please define how these "class register books" were obtained

Response: Thank you for pointing this out. We have clarified that adolescent students were purposively selected from the class register books, which were obtained with permission from the respective school authorities.

Comment: Regarding focus groups – were children and their parents both present in the same focus groups? This poses a concern for social desirability bias on the end of the children to appease to their parents. Please clarify if parents and children had separate focus groups (parents only and children only). If parents and children were present in the same focus group, please justify why this decision was made and how bias arising from potential social desirability bias was avoided.

Response: We have clarified that separate focus group discussions (FGDs) were conducted for children and parents, taking into account diverse classes, ages, and genders. This approach ensured a wide range of perspectives and experiences, minimized social desirability bias, and encouraged richer insights from participants.

Comment: Lines 180 – 184 – please condense language to reflect that interviews and focus groups were conducted until thematic saturation was reached.

Response: The point of saturation or point of redundancy was determined based on an "intellectual guiding principle" for deciding how many interviews to conduct in a qualitative study with purposive sampling as its basis. As mentioned, it is only an intellectual guiding principle with little or no on-the-ground guidance to sample size estimation that will be required to reach this saturation. To comprehend the challenges and recommendations for monitoring screen use of adolescents, a total of 18 interviews were conducted, including 12 IDIs and 6 FGD sessions till thematic saturation was achieved.

Comment: Line 185 – Please clarify the 18 interviews – I am only counting 12 interviews (5 students, 3 parents, and 4 key informant teacher interviews); please note that focus group discussions should not be included in the interview sample size and should be listed separately. E.g., "A total of 12 interviews and 6 FGDs were conducted." Additionally, please list the sample size, N for easier reading.

Response: To understand the challenges and recommendations for monitoring adolescent screen use, a total of 12 interviews (12 IDIs) and 6 FGDs were conducted until thematic saturation was achieved.

Comment: Table 1 – Please change the "School Types" column to be more reflective of categorization based on language of teaching as "school types" suggest classification based on funding – private v. public.

Response: Changed the "School Types" into "Medium of Instruction at Schools".

Comment: Line 191 – Please include/indicate how sociodemographic data was collected from participants.

Response: A section on sociodemographic-related questions was included in every guideline to gather the socio-demographic information of the participants.

Comment: Line 193 – Please include guidelines in the appendix. Alternatively, provide brief descriptions of the contents of the guidelines – did they include suggestions for how long interviews should last? Did they include any probes? How many questions were there? What themes did the interview/FGD questions explore, if any?

Response: Guidelines are included in the appendix, as suggested.

Comment: Line 197 – please update the sentence to state "three and five years of experience on qualitative data collection, respectively."

Response: We have updated the sentence. **Please see the lines: 166-186.**

Comment: Lines 200 – 203 – Which language were the interviews/FGDs conducted in? Were the interviews/FGD conducted in English at English-medium schools and in Bangla at Bangla-medium schools? Please clarify

Response: All interviews were carried out in Bangla, the national language of Bangladesh. **Please see the lines: 173-186.**

Comment: Line 201 – how long were key informant interviews?

Response: There was an understanding gap, and we have resolved this in revised version. **Please see the lines: 151-162.**

Comment: Line 208 – Please use gender neutral pronouns to describe the facilitator

Response: One researcher acted as the facilitator, and the other used the recorder to record the interviews, taking notes simultaneously. **Please see the lines: 166-186.**

Comment: Line 205 – Please clarify if the researcher with the recorder also took notes.

Response: One researcher acted as the facilitator, and the other used the recorder to record the interviews, taking notes simultaneously. **Please see the lines: 166-186.**

Comment: Lines 213- 218 – How was data validity maintained/triangulated? Were the same participants interviewed multiple times? Were parent – child dyads tracked for this triangulation? This section is very unclear – please provide more details regarding the methodology, maybe including a figure would be helpful.

Response: We have now provided a detailed explanation for it. **Please see lines: 206-220.**

Comment: Lines 219 – 220 – Data interpretation should be talked about in the analysis section, please remove this sentence.

Response: Removed

Comment: Line 221 – Please clarify validating data cross various contexts. Validity of data is usually not a concern in one-time interviews or focus group participations. There are usually limitations of self-report or social desirability biases, but it is unrealistic to have responses free of bias in qualitative studies. Who/what is this data being validated against. Is there a standard respondent? Alternatively, are the authors checking for quality of data, i.e., easy to comprehend and bug-free recordings or recordings that are easy to download/transcribe? Please clarify.

Response: Multiple supervisors and investigators were involved to minimise bias and provide a comprehensive interpretation of the data. The use of data and methodological triangulation enabled comprehensive analysis across different research sites and methods. Comparisons between key informants, parents, and students enhanced the validity of the findings. **Please see lines: 206-220.**

Comment: Line 226 – please provide an example file nomenclatures; e.g. "Participant001_English_IDI" or something along those lines that can let readers know of how files were stored in case they want to replicate.

Response: Thank you for the suggestion. We have now clarified the file nomenclature in the manuscript. For ease of organization and replication, all interview files were stored using a standardized naming convention that included the participant number, language of the interview, and interview type. For example: "Participant001_English_IDI" or "Participant012_Bangla_FGD". This approach ensured anonymity while allowing systematic identification and retrieval of files during analysis.

Comment: Line 228 – Earlier it is mentioned that interviews were recorded, but there is now a note implying that some interviews were not recorded. Please explain.

Response: Checked and edited accordingly. **Please see lines: 167-176.**

Comment: Line 231 – Please clarify if there were any consensus meetings to finalize the codebook. Consensus for codebook development is a foundational part of qualitative analysis. Additionally, please include the final codebook in the appendix as it will be helpful to other investigators conducting similar studies.

Response: The research team had a discussion on the developed codebook and finalized the codebook after addressing the critical review and feedback. **Please see lines: 167-186.**

Comment: Line 232– Please list the initials of those involved in the coding process (analysis team).

Response: The first stage of qualitative data analysis began by organising and preparing the field data, including interviews and field notes, by SHK and NS. **Please see lines: 206-220.**

Comment: Lines 236-238 – Rather than validity of data, I am more interested in the inter-rater reliability. Please clarify the exact coding methods – was a double-coding or single-coding method used. Or were a select few transcripts coded by all the analysis team members at the start to ensure consistency in coding before single-coding. Alternatively, did all the analysis team members code all the transcripts? Additionally, please provide Cohen's kappa for a measure of inter-rater reliability.

Response: Thank you for the comment. To clarify, we used a double-coding approach at the initial stage of analysis to ensure consistency and reliability in coding. This approach ensured rigor and consistency in our qualitative coding process. **Please see lines: 206-220.**

Comment: Line 241 – As children (<18) were involved in the study, please elaborate on their consent process. Was parental assent required?

Response: Revised ethical approval process *"The study protocol was approved by the Institutional Review Board (IRB) of icddr,b (Protocol No: PR-22002). Informed written consent was taken from each respondent (parents, teacher) and students were taken consent from their parents as well as assent was taken as their age was above 11 years prior to the interview. All the respondents were informed in Bengali about their rights related to their voluntary participation in the study and withdrew from the interview at any time during the interview. Interviewers of this study clearly conveyed to the respondents the objectives of the study, reasons for their participation in the study, their role, risks, benefits, anonymity and confidentiality, including the rights not to participate or withdraw. After getting acquainted with all these issues, the respondents decided whether to participate in this study. Confidentiality during and after data collection was ensured, and no identifiable information was used that may expose the identity of the participants. Only the research team members have access to the collected data, which were stored securely. After the interview, each participant received a gift (equivalent to BDT370 [USD3] as a token of appreciation for their valuable time."*

Comment: Line 249 – Where were the recordings/data stored? Please provide a location e.g. "password protected computer only accessible by the research team." Additionally, were the interviews themselves de-identified? Please clarify.

Response: Confidentiality during and after data collection was ensured, and no identifiable information was used that may expose the identity of the participants. Only the research team members have access to the collected data, which were stored securely in the password-protected central data storage of icddr,b.

Comment: Line 250 – Please specify the token of appreciation amount (in Bangladeshi currency and USD).

Response: We have specified the token of appreciation amount, provided in both Bangladeshi Taka and its equivalent in USD. **Please see lines: 219-220 as follows "*After the interview, each***

participant received a gift (equivalent to BDT370 [USD3] as a token of appreciation for their valuable time."

Comment: Line 253 – Please note that Table 2 only provides the demographics for children who participated in the study (from what I gather). Please update language to reflective of this specific demographic. As parents and teachers were also interviewed/included in the study, please add additional tables for demographic characteristics of each of these groups. Alternatively, you can create one large table and have subgroup frequencies included as columns (column 1 = children, column 2 = parents, column 3 = teachers)

Response: Thank you for this comment. We have clarified in the manuscript that Table 2 presents the demographic characteristics of the child participants only. To provide a complete overview of all study participants, we have added additional tables reporting the demographics of parents and teachers. Alternatively, we have also considered creating a combined table with subgroup frequencies in separate columns (children, parents, teachers) to allow easy comparison across participant groups. This ensures transparency and allows readers to understand the composition of all participants included in the study.

Comment: Line 263 – 268 – please provide your definition of screen viewing and then provide a summary of responses/content analysis by participant subgroup that relate to screen viewing as a concept. To strengthen this paragraph, you can also summarize any quotes or general remarks that point towards relations between screen viewing and health.

Response: We have provided a clear definition of screen viewing and summarized responses by participant subgroup. We have also included quotes and remarks highlighting the relationships between screen viewing and health to strengthen this section.

Comment: Lines 325 – 332 – please provide more context using literature or another source to contextualize the social climate in Dhaka.

Response: Revised the text: Dhaka, a densely populated megacity, has limited open and child-friendly spaces, restricting children's opportunities for outdoor play. Concerns over traffic, pollution, and safety further confine children indoors. At the same time, rapid expansion of internet and smartphone access has made screen-based activities more available and attractive. Together, these factors create a social climate that favors indoor, screen-based leisure—an important context for interpreting participants' views in this study. **Please see lines: 76-90.**

Comment: Lines 394 – 403 – please provide an exemplary quote to support the claim being made. It is interesting and I would like to read what was said.

Response: We have added an exemplary quote from participants to support the claim and provide greater insight into their perspectives.

Comment: Line 410 – please check the formatting of the quote.

Response: Tanks. Formatted accordingly

Comment: Line 431 – please include a transition paragraph that segways into the new subsection of recommendations. Also, please consider condensing these recommendations and moving them to the discussion section or including them as a table. Great work on the recommendations!

Response: The study participants did not limit themselves to sharing only the challenges that they faced with morning screen time but also provided some contextualized recommendations to address these challenges. **Please see lines: 433-513 and entire discussion part.**

Comment: Line 484 – Please rename the subheading to "ensure social safety"

Response: Changed to "Ensuring social security"

Comment: General note – I am missing quotes or analysis based on SES status as that seemed to be a big factor influencing health of adolescents and technology use according to the introduction written by the author's. Please include 1 or 2 quotes that highlight any relationships of SES, technology use, and health/concerns.

Response: Thank you for this important observation. We have now incorporated participants' perspectives highlighting the influence of socioeconomic status (SES) on screen use and related health concerns. For instance, one parent noted: "Families with higher income often provide multiple devices to their children, which makes it harder to control screen time and can affect their sleep and concentration." Similarly, a student mentioned: "Some of my friends don't have personal devices at home, so they play outside more, and I think they stay healthier than those who spend all day on phones or tablets." These quotes illustrate how SES shapes access to technology and, consequently, children's screen habits and associated health outcomes. ***Please see lines: 369-378.***

Comment: Lines 533 – 537 – I appreciate the domains/points brought up regarding SV (e.g. not all screens are equal). It would potentially be helpful to include a table in the results section listing exemplary quotes organized by these domains for easier comprehension.

Response: Thank you for this comment. We tried to incorporate in text.

Comment: Line 567 – please elaborate. The paragraph is only one sentence currently.

Response: We have elaborated on the paragraph at line 567, expanding it to provide greater detail and context.

Comment: Line 583 – what were the results of the clustered randomized controlled trial?

Response: Here is the result: Total physical activity (PA), measured in MET-min/week, increased from baseline to 8 and 12 weeks in the intervention group (IG) by 3%–5%, whereas it decreased in the control group (CG) by 5%–3%. Over time, PA improved significantly in the IG compared to the CG ($p < 0.001$). Average sedentary time (ST) in minutes per day decreased from baseline to 8 and 12 weeks in the IG by 28%–35%, while remaining largely unchanged in the CG (6%–5%). At 12 weeks, the IG had a significantly lower average ST than the CG. Additionally, average

knowledge scores on PA and sedentary behavior (SB) were significantly higher in the IG than in the CG at 12 weeks ($p < 0.001$).

Comment: Line 608 – I like the call to action being presented here. To tighten narrative, please tie it in or include how existing pediatric guidelines could be modified. Alternatively, should we consider devising new pediatric guidelines? Curious to hear your thoughts.

Response: We have tied the call to action to existing pediatric guidelines, discussing potential modifications and the possibility of developing new guidelines to better address adolescent screen use in the current context.

Comment: Under limitations, please also include the influence of social desirability biases among participants or potential selection for responses given the skew towards Bangla-medium participants in the study. Additionally, please include limitations related to potential human error (such as not recording interviews, errors in translating from Bangla to English or vice versa).

Response: Thanks for this comment and suggestion. We have reviewed our manuscript carefully and removed the limitation points. Since it's a qualitative study and there is no generalizations issue like quantitative study, we have removed the section.

Sincerely
Dr. Shahria Hafiz Kakon
On behalf of Nahian Sultana,Bidhan Krishna Sarker, Tanjir Rashid Soron, Rashidul Hoque, Fahmida Tofail
