# Supplementary material for: Parental monitoring challenges and recommendation for managing excessive screen use among students aged 12–14 years in Dhaka, Bangladesh: A qualitative exploration
Source: PLoS One. 2026 Oct 5;21(10):e0359518. doi: 10.1371/journal.pone.0359518 (PMC13637862; doi:10.1371/journal.pone.0359518)
Supplement: Supplementary file 6 [file pone.0359518.s007.pdf]

March 17, 2026

To
Derebe Madoro Bunte
Academic Editor
PLOS ONE

Subject: Responses to Editor and Reviewer comments on the manuscript [PONE-D-25-02782R1]

Dear Editor,

Thank you for allowing us the opportunity to improve and resubmit our manuscript titled "Parental monitoring challenges and recommendation for managing excessive screen use among students aged 12–14 years in Dhaka, Bangladesh: a qualitative exploration". We sincerely thank the Academic Editor and both reviewers for their careful evaluation of our manuscript and for their constructive and valuable comments. We have carefully reviewed all the comments and have addressed them individually. Additionally, we have incorporated the suggested changes into the manuscript where applicable. Below, we provide a point-by-point response, indicating how each concern has been addressed in the revised manuscript. Page and line numbers refer to the revised version.

<u>Response to Academic Editor's Comments:</u>

Comment 1: Major comments highlight the need for a clear definition of "excessive use," a conclusion that fully answers the study aim with a future research suggestion, and clarification of unclear statements and missing citations. Extensive grammar and language corrections are required throughout.

Response: Thank you for this comment. We have addressed each point mentioned. The definition of "excessive screen use" is clearly stated in the introduction and methods section as more than 2 hours per day, consistent with international guidelines. Please see pages 4, 8, and lines 72-74, and 177-179.

Further, we have rewritten the conclusion in order to directly answer the study's aim and now include a clear recommendation for future research. The manuscript has undergone extensive language editing for grammar, clarity, and academic tone. Please see pages 6, 28-29, and lines 120-123, 629-643.

Comment 2: The results section needs major restructuring, with the current 14 subthemes consolidated into 3–5 main themes supported by a clear thematic overview. The study's aim must align with the perspectives presented, and the recommendations should be written narratively and explained through the chosen framework.

Response: Thanks for this suggestion. As suggested, the results section has been fully restructured, consolidating the original 14 sub-themes into four major challenge-based themes, each supported by sub-themes and illustrative quotes. The study aim has been refined to reflect the inclusion of

parents', teachers', and adolescents' perspectives. Recommendations are now presented narratively, explicitly linked to the guiding framework, which is described in both the Methods and Discussion sections. Please see pages 10-11, 13-24, and lines 228-235, 281-547.

<u>Response to 1st Reviewer's Comments:</u>

Comment-1: "This study aimed to explore the challenges parents face when their students engage in excessive use of electronic digital screens. Additionally, it sought to capture participants' recommendations for the best use of digital devices among students." Please make sure the conclusion has answered this accordingly, and you could give one suggestion for further research recommendations.

Response: Many thanks for pointing out this and for your guidance. As suggested, the conclusion section has been revised to explicitly address both components of the study aim, parental monitoring challenges, and participant recommendations. We have also added a clear future research suggestion focusing on longitudinal and intervention-based studies to evaluate effective screen-use management strategies. Please see pages 28-29 and lines 629-643.

Comments-2: Please specify where you describe the definition of "excessive use" since you responded "We have quantified "excessive use" of digital devices as more than 2 hours per day in line 160. I have not seen the statement in the revised manuscript. Please recheck.

Response: We thank the reviewer for noting this omission. The definition of "excessive screen use" has now been explicitly included in the Methods section (Study Participants and sampling), specifying use of digital devices for more than 2 hours per day, with appropriate citation. Please see pages 4, 8, and lines 72-74, and 177-179.

Comments-3: check line 124 = what is the significance of mentioning this sentence here? "We conducted qualitative interviews from the same schools where previous quantitative study was performed."

Response: Thanks for pointing this out. We agree that clarification is required. The sentence has been revised to explain that the qualitative study was designed as a follow-up exploration to contextualize findings from the earlier quantitative work. We have added a citation of previous quantitative work. This clarification has been added to the Study Design section. Please see page 6 and lines 128-131.

Comment-4: check line 133, please provide citation here.

Response: An appropriate citation has now been added to support the respective statement.

Comment-5: I understand writing an academic manuscript is a bit tricky for non native English. Please have a thorough and detailed check on the manuscript since I found many need to be proofread, such as:
check line 33, 37= "who attending" is this grammatically correct?
Check line 59

Check line 109
check line 111= You meant "in the UK."
check line 147, 160, 173, 208, 214, 219, 225, 261 etc.

Response: We fully acknowledge this concern. The manuscript has undergone thorough language editing, correcting all highlighted issues and many additional grammatical, punctuation, and stylistic errors throughout the text. We believe the manuscript now meets the standards of clear and correct academic English. Thank you.

<u>Response to 2nd Reviewer's Comments:</u>

Comment-1 Despite the improvement, there are significant grammar and punctuation errors, which, for me, are key in academic writing in English.

Response: Many thanks for the feedback. We have again thoroughly checked the grammar and punctuation issues and addressed them throughout the paper. We appreciate this feedback and have conducted a comprehensive proofreading and language revision of the entire manuscript, including professional-level editing for grammar, punctuation, and clarity.

Comment-2: I am concerned about the manner in which the results are presented, which hinders your ability to make good points in the discussion. The overview of how the themes will be presented is not yet sufficient. Please provide an overview of the number of themes that will be shown, name them, and describe how they will be presented and why? The overview of themes in the Results section is insufficient.

Response: Thank you for this valuable comment. We have now restructured the result sections and presented the results considering the socio-ecological models framework pattern. We have added a clear thematic overview at the data analysis sub-section under the method section, and structured the challenge sub-section under the result accordingly. Please see pages 11, 13-19, and lines 234-235, 280-427.

Comment-3: There are 14 themes (shown below), which appear to be subthemes and are not consolidated in order to answer your aim of provide the results of "the challenges parents face in supervising and restricting children's SV in urban Dhaka, Bangladesh, and." feedback on strategies that may help achieve a healthier balance between digital engagement and overall child well-being." These need to be consolidated into 3-5 major themes related to your aim. Please review thematic analysis with Braun and Clarkes methods for further information on this.
Themes include
1. Students' use of digital devices for education
2. Student's reliance on the internet for learning support
3. Parents' perspectives on digital learning and supervision
4. Teachers' perspectives on digital learning and supervision
5. Safety concerns and limited peer interaction
6. Limited family time and communication
7. Scarcity of outdoor play and recreational opportunities
8. Unwanted exposure to age-inappropriate contents

9. Socioeconomic status and screen time
- remember in this subtheme your sample not randomised so you did not find an association?
10. Student's perspective on unwanted content
11. Teachers' perspective on supervision
12. Students' perspective on supervision
13. Lack of parental digital literacy
14. Recommendations

Response: We agree with this important suggestion. As suggested, we have revised the theme based on the socio-ecological framework and restructured the finding accordingly. Please see pages 13-24 and lines 280-547.

Comment-4: The sub-theme on socioeconomic status should not imply association due to non-random sampling.

Response: Thank you for identifying this issue. As the findings conflict with the methods, we have removed the specific findings to avoid any inconsistencies.

Comment-5: The aim focuses on parental challenges, yet perspectives from teachers and students are also presented. Not only parents' perspectives are provided, but also teachers and students, nor is it related only to parental challenges is managing SV? Please adapt your aim to represent the evidence you are presenting or adapt the evidence you are presenting?

Response: Thank you for pointing this out. We have now revised the aims, taking into account the perspectives of both teachers and students. Please see page 6 and lines 120-123.

Comment 6: Recommendations are still under a bulleted format, although I do appreciate them being put within a framework. Please describe how you will be presenting these recommendations according to this framework in your methods section and provide a brief review of the framework.

Response: Thanks for this comment and concern. The recommendations section has been rewritten in narrative form, organized according to the socio-ecological framework used. We have added a brief description of the framework in the data analysis sub-section under the methods section and linked the recommendations explicitly to its components in the Results and Discussion. Please see pages: 10-11, 19-24, line: 228-235, 430-547.

Response to comments on the PDF file, which was attached to the email body:
Comments for the Introduction section

Comment: Where are the references for this? (line no 58-60)

Response: We have now added appropriate references. Thank you.

Comment: Add some further references here? (line no 65-67)

Response: Thank you. We have added further reference according to your suggestion.

Comment: putting it this way makes it seem like high SES is an unhealthy behaviour. I would move this to the first one you mention, then add in unhealthy behaviours (line-80)

Response: We agree with your thoughts and rearranged the sentences accordingly. Please see page 4 and line no 78-85.

Comment: I would remove this section and replace it with the current (line no-80)

Response: Thank you, and we have made the suggested change accordingly.

Comment: The grammar here is incorrect - engaged on devices? or engaged in using devices? (line no 81-82)

Response: Thanks for this concern. We have checked the grammar and identified that 'engaged in using a device' is correct.

Comment: combining... with use of internet...? (line no 83-85)

Response: We have paraphrased this sentence. Please see page 4 and line no 86-87.

Comment: This is colloquial language (line no 94)

Response: We have paraphrased this sentence.

Comment: Grammar (line no 98)

Response: We have corrected grammar.

Comment: Is there nothing from a more similar context to Bangladesh? (line no 99-105)

Response: Thank you for this suggestion. We have added the literature from Bangladesh and a similar context, including the literature from the developed country context.

Comment: Capital letter (line no 109)

Response: We addressed the issue.

Comment: This whole sentence doesn't make complete sense. (line no 109-111)

Response: We paraphrased this sentence now.

Comment: Please clarify whether you mean formal guidance on device use? (line no 115)

Response: Thank you for the comment. We have revised the respective lines to clear the meaning of formal guidance. Please see pages 24 and lines 531-556.

Response to the comments for the Materials and Methods section

Comment: Please reference this here? (line n0 125)

Response: We have added the appropriate reference.

Comment: to provide? (line no 126)

Response: We have paraphrased this sentence.

Comment: Reference (line no 131-133)

Response: We have provided an appropriate reference for this sentence.

Response to the comments for the Study setting

Comment: There is no information here about these settings, so that we would be able to know if our setting is similar or not to allow for transferability. All we know if that it is a high-seas area? (line no- 136-140)

Response: Thank you for this important comment. We have now provided a brief information about the setting under the method section. And as per the comment, we have made the necessary change throughout the paper.

Comment: this needs to go under participant sampling - not study setting (line no 143-148)

Response: Thanks for your suggestions. We have removed this part from the study setting section and added it to the study participants and sampling section. Please see pages 8 and lines 160-165.

Response to the comments for Study participants and sampling

Comment: Were these parents of the same children, i.e., a dyad, or different parents and children? (line no 151-153)

Response: Thank you for this comment. Parents and students were interviewed independently and were not recruited as parent-child dyads. Please see pages 7 and lines 151-159.

Comment: Also at the school (line no- 175,180)

Response: Thank you for this query. The focus group discussions were conducted on school premises in a quiet, private teacher's common room with the permission of the school authorities. Participants were seated in a circle to facilitate open discussion and interaction. Only the participants and the research team were present during the FGDs, and all sessions were audio recorded with prior informed consent. This information has been clarified in the methods section under the study participation and sampling. Please see page 8.

Comment: observed the interviews or team, doing what? (line no 182,183)

Response: Thank you for this clarification request. The Mental Health Expert did not observe participants; rather, they observed the interview process and the performance of the interview team. The expert provided methodological feedback on interviewing techniques, probing, neutrality, and ethical conduct during data collection when necessary. This clarification has been incorporated into the Methods section.

Response to the comments for the Operational definition

Comment: This doesn't seem like the right place for this? (line no 188)

Response: Thank you for your guidance. We have moved the operational definition subsection to the right place, following the standard guideline just before the data collection subsection. Please see pages 10.

Response to the comments for the Result section:

Comment: This doesn't add much to the analysis - I would take this out. Please add in here an overview of the themes. I.e., note how many themes were identified, the names of the themes and whose perspectives are represented and why... (line no-249-250)

Response: Thank you for this comment. We have used the socio-ecological framework, which has been mentioned in the data analysis sub-section, and following the framework, we presented the findings under challenges and recommendations. In challenges, we identified four themes under the different levels of the framework, and findings under the recommendation have been presented under the framework components.

Comment: A more appropriate title for this seems to be Lack of guidance on device use for education? (line no 261)

Response: The sub-theme has been renamed to "Educational reliance on digital devices without structured guidance," which more accurately reflects the identified challenge.

Comment: only one quote per sub-theme (line no 281-289)

Response: We have reduced the number of quotes and retained one representative quote per sub-theme to improve readability and conciseness.

Comment: this is not a challenge though? (line no 290)

Response: We have removed that subtheme, and only four board themes have been kept. The sub-theme has been renamed to "Educational reliance on digital devices without structured guidance," which more accurately reflects the identified challenge. Please see pages 16 and lines 360-365.

Comment: how, when this sample is not randomised? The association between SES and screen time cannot be inferred, given the non-random sample. (line no 370-379)

Response: Thank you for highlighting this issue. We have now removed the particular findings to avoid methodological inconsistency.

Comment: Why is this section described with bullet points? If you want to include bullet points rather put this in a text box in a shortened manner and describe these recommendations here. (line no- 433)

Response: We have rewritten this section in narrative form and integrated participant recommendations into thematic paragraphs, while retaining clarity and coherence. Please see pages 19-24 and lines 431-540.

Response to the comments for the Discussion section

Comment: My problem is that the results haven't been presented as challenges, but as perspectives, influencing factors, and concepts. These subthemes need to be reworked into 3-4 major overarching themes that are consistent and do not have any overlapping concepts with other (line no 516)

Response: We have revised the result section with highlighted specific 4 subthemes into challenges. All discussion points now directly correspond to findings presented earlier, avoiding the introduction of new or unsupported themes. Please see pages 13-19 and lines 281-422.

Comment: But where have you presented these two themes in the evidence above? (line no 529-534)

Response: Thanks for your suggestion. We have rewritten this sentence to remove the inconsistency from the discussion part. We have mentioned this topic in the challenges section as well. Please see pages 16-17 and lines 359-384.

Comment: This appears to be an overarching theme - barriers to engaging in non-SV-based activities? (line no- 535-536)

Response: Thank you for this insightful observation. We agree that parents' concerns about social safety, particularly for girls, reflect a broader barrier to children's engagement in non-screen-based activities. This captures parental safety concerns, limited outdoor opportunities, and social restrictions that influence children's activity patterns. This ensures consistency with the other themes and highlights the contextual factors affecting screen use in the challenges section. Please see pages 18-19 and lines 406-428.

Comment: How is this a challenge to managing SV use, though? Concerns over SV, yes, but not experienced challenges in managing SV. (line no 546,547)

Response: Thank you for this important comment. We acknowledge that concern about exposure to inappropriate content reflects perceived risks of screen viewing rather than a direct challenge in

managing screen time. To address this, we have clarified in the manuscript that these concerns were reported as part of the broader contextual factors influencing parents' monitoring strategies, rather than as operational difficulties in enforcing screen time limits. This distinction helps to differentiate between challenges in managing screen use and the perceived risks associated with content exposure.

Kind regards,
Dr. Shahria Hafiz Kakon
On behalf of Nahian Sultana, Bidhan Krishna Sarker, Tanjir Rashid Soron, Rashidul Haque, Fahmida Tofail
