# Supplementary material for: Parental monitoring challenges and recommendation for managing excessive screen use among students aged 12–14 years in Dhaka, Bangladesh: A qualitative exploration
Source: PLoS One. 2026 Oct 5;21(10):e0359518. doi: 10.1371/journal.pone.0359518 (PMC13637862; doi:10.1371/journal.pone.0359518)
Supplement: Supplementary file 4 [file pone.0359518.s002.docx]

**Specific Reviewer Notes:**

**Article Title: Parental monitoring challenges and recommendation for managing excessive screen use among adolescents aged 12–14 years in Dhaka, Bangladesh: a qualitative exploration**

This study tackles an important and relevant topic with valuable insights and practical recommendations that could significantly contribute to the field. The authors have conducted meaningful research that addresses real challenges faced by parents in managing adolescent screen use. However, the manuscript requires substantial methodological clarification and improved presentation of results to meet publication standards. The findings and recommendations are strong, but the research would benefit from more rigorous reporting and clearer methodology to enhance its impact and reproducibility.

**Article Summary:** This qualitative study explores the challenges parents face in monitoring their adolescents' (aged 12-14) screen use in Dhaka, Bangladesh. Using focus group discussions, in-depth interviews, and key informant interviews with 25 children, parents, and teachers from six schools, the study identifies four main challenges: educational necessity of devices creating supervision difficulties, restricted outdoor activities increasing device dependence, exposure to age-inappropriate content, and parental digital illiteracy. The study provides comprehensive recommendations including school-based interventions, national-level policies, enhanced parent-child bonding, and secured infrastructure development.

**Strengths:**

- **Comprehensive multi-stakeholder approach:** The inclusion of children, parents, and teachers provides valuable triangulation of perspectives on screen use challenges
- **Practical and actionable recommendations:** The study offers concrete suggestions for schools, parents, and policymakers that are contextually relevant to the Bangladeshi setting
- **Important and timely research topic:** Addresses a critical public health concern regarding adolescent screen use in a rapidly digitizing society
- **Rich qualitative data:** The combination of FGDs, IDIs, and KIIs provides depth and breadth to understanding the phenomenon
- **Cultural contextualization:** Appropriately situates findings within the social and educational context of Dhaka, Bangladesh

**Weaknesses:**

***Major Concerns***

- Lack of clear operational definitions
- Insufficient inclusion/exclusion criteria
- Unclear data collection procedures
- Inadequate sample size justification
- Missing inter-rater reliability measures
- Incomplete demographic reporting
- Incomplete consent procedures

***Minor Concerns***

- Grammar issues
- Presentation and organization

**Suggestions for Improvement:**

- Suggest using a grammar checker and proofreading – a lot of misspellings and run on sentences which make it hard to read the paper
- Introduction –
  - Paragraph 1 - link between parenting and SES is established but it is unclear as to how parenting relates to health of adolescents 🡪 explicitly state that parenting style is a mediator of technology use and thus, health of adolescents
  - line 95 – please mention whether the screen time usage is a mean or median measure
  - line 105 – author’s mention AAP. The study context is Dhaka, Bangladesh, are there any Bangladeshi guidelines for pediatrics that should be mentioned? It will help strengthen the context and be directly applicable
  - Currently, paragraphs 1,2, 3 are not logically organized – suggestion for organization 🡪 paragraph 1: clearly outline the problems on health brought forth by SV; paragraph 2: outline the literature for why parents are intentionally/unintentionally using technology to help with parenting; paragraph 3: outline existing pediatric guidelines and perspectives for healthier management of technology use among adolescents
- Materials and Methods
  - Line 160 – please quantify “excessive use” of digital devices using a threshold (for example, > 5 hours/day, etc.)
  - Lines 160 – Lines 167 – please be specific with inclusion and exclusion criteria for the study. Please list the minimum age inclusion/exclusion for each participant, along with any other inclusion/exclusion criteria that was relevant to the study (e.g. technology use, language, family size, etc.) Please try to be specific in this section.
  - Line 165 – while teacher’s play a role in children’s development and education, it is unclear as to how they would be valuable as key informants for the aim of the research as they are not providing child care; the introduction argues that SV has come on as a way of making caregiving easier. Please include some literature/citations that can help justify the inclusion of teachers as I do agree that they are an important perspective to consider
  - Line 171 – please define how these “class register books” were obtained
  - Regarding focus groups – were children and their parents both present in the same focus groups? This poses a concern for social desirability bias on the end of the children to appease to their parents. Please clarify if parents and children had separate focus groups (parents only and children only). If parents and children were present in the same focus group, please justify why this decision was made and how bias arising from potential social desirability bias was avoided.
  - Lines 180 – 184 – please condense language to reflect that interviews and focus groups were conducted until thematic saturation was reached.
  - Line 185 – Please clarify the 18 interviews – I am only counting 12 interviews (5 students, 3 parents, and 4 key informant teacher interviews); please note that focus group discussions should not be included in the interview sample size and should be listed separately. E.g., “A total of 12 interviews and 6 FGDs were conducted.” Additionally, please list the sample size, N for easier reading.
  - Table 1 – Please change the “School Types” column to be more reflective of categorization based on language of teaching as “school types” suggest classification based on funding – private v. public.
  - Line 191 – Please include/indicate how sociodemographic data was collected from participants.
  - Line 193 – Please include guidelines in the appendix. Alternatively, provide brief descriptions of the contents of the guidelines – did they include suggestions for how long interviews should last? Did they include any probes? How many questions were there? What themes did the interview/FGD questions explore, if any?
  - Line 197 – please update the sentence to state “three and five years of experience on qualitative data collection, respectively.”
  - Lines 200 – 203 – Which language were the interviews/FGDs conducted in? Were the interviews/FGD conducted in English at English-medium schools and in Bangla at Bangla-medium schools? Please clarify
  - Line 201 – how long were key informant interviews?
  - Line 208 – Please use gender neutral pronouns to describe the facilitator
  - Line 205 – Please clarify if the researcher with the recorder also took notes.
  - Lines 213- 218 – How was data validity maintained/triangulated? Were the same participants interviewed multiple times? Were parent – child dyads tracked for this triangulation? This section is very unclear – please provide more details regarding the methodology, maybe including a figure would be helpful.
  - Lines 219 – 220 – Data interpretation should be talked about in the analysis section, please remove this sentence.
  - Line 221 – Please clarify validating data cross various contexts. Validity of data is usually not a concern in one-time interviews or focus group participations. There are usually limitations of self-report or social desirability biases, but it is unrealistic to have responses free of bias in qualitative studies. Who/what is this data being validated against. Is there a standard respondent? Alternatively, are the authors checking for quality of data, i.e., easy to comprehend and bug-free recordings or recordings that are easy to download/transcribe? Please clarify.
  - Line 226 – please provide an example file nomenclatures; e.g. “Participant001_English_IDI” or something along those lines that can let readers know of how files were stored in case they want to replicate.
  - Line 228 – Earlier it is mentioned that interviews were recorded, but there is now a note implying that some interviews were not recorded. Please explain.
  - Line 231 – Please clarify if there were any consensus meetings to finalize the codebook. Consensus for codebook development is a foundational part of qualitative analysis. Additionally, please include the final codebook in the appendix as it will be helpful to other investigators conducting similar studies.
  - Line 232– Please list the initials of those involved in the coding process (analysis team).
  - Lines 236-238 – Rather than validity of data, I am more interested in the inter-rater reliability. Please clarify the exact coding methods – was a double-coding or single-coding method used. Or were a select few transcripts coded by all the analysis team members at the start to ensure consistency in coding before single-coding. Alternatively, did all the analysis team members code all the transcripts? Additionally, please provide Cohen’s kappa for a measure of inter-rater reliability.
  - Line 241 – As children (<18) were involved in the study, please elaborate on their consent process. Was parental assent required?
  - Line 249 – Where were the recordings/data stored? Please provide a location e.g. “password protected computer only accessible by the research team.” Additionally, were the interviews themselves de-identified? Please clarify.
  - Line 250 – Please specify the token of appreciation amount (in Bangladeshi currency and USD).
- Results –
  - Line 253 – Please note that Table 2 only provides the demographics for children who participated in the study (from what I gather). Please update language to reflective of this specific demographic. As parents and teachers were also interviewed/included in the study, please add additional tables for demographic characteristics of each of these groups. Alternatively, you can create one large table and have subgroup frequencies included as columns (column 1 = children, column 2 = parents, column 3 = teachers)
  - Line 263 – 268 – please provide your definition of screen viewing and then provide a summary of responses/content analysis by participant subgroup that relate to screen viewing as a concept. To strengthen this paragraph, you can also summarize any quotes or general remarks that point towards relations between screen viewing and health.
  - Lines 325 – 332 – please provide more context using literature or another source to contextualize the social climate in Dhaka.
  - Lines 394 – 403 – please provide an exemplary quote to support the claim being made. It is interesting and I would like to read what was said.
  - Line 410 – please check the formatting of the quote.
  - Line 431 – please include a transition paragraph that segways into the new subsection of recommendations. Also, please consider condensing these recommendations and moving them to the discussion section or including them as a table. Great work on the recommendations!
  - Line 484 – Please rename the subheading to “ensure social safety”
  - General note – I am missing quotes or analysis based on SES status as that seemed to be a big factor influencing health of adolescents and technology use according to the introduction written by the author’s. Please include 1 or 2 quotes that highlight any relationships of SES, technology use, and health/concerns.
- Discussion –
  - Lines 533 – 537 – I appreciate the domains/points brought up regarding SV (e.g. not all screens are equal). It would potentially be helpful to include a table in the results section listing exemplary quotes organized by these domains for easier comprehension.
  - Line 567 – please elaborate. The paragraph is only one sentence currently.
  - Line 583 – what were the results of the clustered randomized controlled trial?
  - Line 608 – I like the call to action being presented here. To tighten narrative, please tie it in or include how existing pediatric guidelines could be modified. Alternatively, should we consider devising new pediatric guidelines? Curious to hear your thoughts.
  - Under limitations, please also include the influence of social desirability biases among participants or potential selection for responses given the skew towards Bangla-medium participants in the study. Additionally, please include limitations related to potential human error (such as not recording interviews, errors in translating from Bangla to English or vice versa).

**Overall Recommendation:**

**MAJOR REVISION** - The study addresses an important and timely research question with valuable findings and practical recommendations. However, significant methodological concerns regarding data collection procedures, analysis transparency, and reporting standards must be addressed before publication. The authors should focus on clarifying their methodology, improving data presentation, and ensuring reproducibility of their analytical approach. With these revisions, this could be a valuable contribution to the literature on adolescent screen use and parental monitoring challenges.

**Alignment w/ *PLOS One* Criteria (Ranked 1 = Strongly Disagree, 3 = Neutral, 5 = Strongly Agree)**

1. The study presents the results of original research. **5**
2. Results reported have not been published elsewhere. **5**
3. Experiments, statistics, and other analyses are performed to a high technical standard and are described in sufficient detail. **2**
4. Conclusions are presented in an appropriate fashion and are supported by the data. **3**
5. The article is presented in an intelligible fashion and is written in standard English. **3**
6. The research meets all applicable standards for the ethics of experimentation and research integrity. **3**
7. The article adheres to appropriate reporting guidelines and community standards for data availability. **5**
