# Supplementary material for: Parental monitoring challenges and recommendation for managing excessive screen use among students aged 12–14 years in Dhaka, Bangladesh: A qualitative exploration
Source: PLoS One. 2026 Oct 5;21(10):e0359518. doi: 10.1371/journal.pone.0359518 (PMC13637862; doi:10.1371/journal.pone.0359518)
Supplement: Supplementary file 5 [file pone.0359518.s006.docx]

| Line | Comment |
| --- | --- |
| 27 | Consider replacing overindulgence with a more appropriate word |
| 29 and 48 | Specify whose specific recommendations are you getting rather than saying participants or population, you have three different types of participants in your study or three populations |
| 36 | What different types of in-depth interviews?  “A thematic analysis was performed using an inductive approach based on the socio-ecological model framework” – an inductive approach cannot be base specifically on a model or framework, this is a deductive approach as a framework is being applied? |
| 47 | Parental challenges – please be more specific |
| 55 | Do you mean inescapable? |
| 60 | Four factors related to SES were noted but then only one described? |
| 65 | In adolescents? |
| 70 | In addition to what? I would remove this |
| 72 | I would continue using the term “screen viewing” that screen time |
| 75 -77 | Does this not belong better in the first paragraph where you are talking about what influences technology habits? This second paragraph is about the outcomes of high SV so it doesn’t really align with the argument |
| 81 | Increased compared to what? Historical use? The WHO recommendations? |
| 84 | SV acronym described again? |
| 87-91 | Surely the AAP guidelines should be discussed together with the WHO guidelines in the paragraph above? Or bring the WHO guideline here? |
| 93-108 | This section seems to be about the challenges parents face in managing SV in adolescents if I am right – please look at the evidence presented in this section and synthesize fully? For example evidence from Canda is presented, then Bangladesh, then HIC contexts – with Canada also being a HIC context, that literature should be synthesised with the latter. |
| 96 | SV restrictions **can** limit opportunities and development. This only really talks to part of what the paper says – HICs are described as more restrictive, and restrictions can reduce opportunity but also increase risk |
| 96 | In what circumstances? LMIC’s? |
| 100 | The sole challenge? A key challenge maybe |
| 108-111 | The Indian study is not referenced? |
| 108 | The ongoing section of this paragraph is a new argument and should be a new paragraph I think this seems to be about strategies to manage SV, no longer the challenges that parents face in trying to manage SV in their adolescents? |
| 122 | From the perspectives of parents, teachers and 12-14 year old adolescents (not only teacher and adolescents?) |
| 133 | A combination of multiple **data collection methods** (not tools)  Lines 131-136 are data collection methods, not study design and therefore should not be discussed under this section. Please rather describe the qualitative study here to provide further background to the study design and how the qualitative work fits into this |
| 138-144 | There are no references for this section? |
| 145 | Start off this section with precisely who your different samples were.  This section is on the whole very ‘wordy’ and this makes it a little difficult to follow. |
| 167-171 | Remove data collection methods from this section, as this related to data collection not sampling |
| 208-209  212-213 | Check sentences for clarity/grammar |
| 217-220 | This information relates to data analysis and sampling, please rather place in the correct sections, rather than data collection methods? |
| 227 | You can described thematic analysis here already, as familiarization is a part of the process so you effectively already started thematic analysis with familiarization? |
| 237 – 241 | Still limited discussion of reflexivity, specifically important when using an inductive approach, and how this relates to rigour. |
| 255 | The term ‘gift’ doesn’t sit well in terms of possible coercion and potential for social desirability bias – it would be better to talk about compensation for their time. |
| 281 | The description of the themes do not relate to your actual themes?  i.e. theme 1 is not patterns and purposes…. But challenges faced by parents in monitoring.. |
| 298 | The quote is more about a lack of opportunities for family engagement that adolescent preferences for engaging with friends than family? |
| 316 | What is eve-teasing? |
| 318 | The quote is not about a lack of availability of peers/cousins, but lack of engagement when they are together? |
| 331 | This quote is about digital device usage – not unsupervised access, so I’m not sure if it is good evidence? |
| 338 | Is this not a recommendation? |
| 386 | This is the impact of excessive SV and not related to theme? |
| 440 onwards | Recommendations are still presented as bullet points with limited quotes attached as evidence? |
| 462, 509 | Choose one of the quotes and cut them shorter or put under the appropriate section above |
| 558-560 | This does not link to the preceding or proceeding paragraph? |
| 585 | “Many parents reported feeling unprepared to discuss sexual development, which compounded the challenges of supervision.”  I don’t think that this was presented in the results? |
