# Supplementary material for: Parental monitoring challenges and recommendation for managing excessive screen use among students aged 12–14 years in Dhaka, Bangladesh: A qualitative exploration
Source: PLoS One. 2026 Oct 5;21(10):e0359518. doi: 10.1371/journal.pone.0359518 (PMC13637862; doi:10.1371/journal.pone.0359518)
Supplement: Supplementary file 6 [file pone.0359518.s008.docx]

August 5, 2026

To

Derebe Madoro Bunte

Academic Editor

PLOS ONE

Subject: Responses to Editor and Reviewer comments on the manuscript [PONE-D-25-02782R2]

Dear Editor,

Thank you for allowing us the opportunity to improve and resubmit our manuscript titled “Parental monitoring challenges and recommendation for managing excessive screen use among students aged 12–14 years in Dhaka, Bangladesh: a qualitative exploration”. We sincerely thank the Editor and the Reviewer for their careful evaluation of our manuscript and for providing thoughtful and constructive comments. We greatly appreciate the time and effort devoted to reviewing our work. The comments and suggestions have been invaluable in improving the quality, clarity, and scientific rigor of the manuscript. We have carefully considered each comment and revised the manuscript accordingly. A detailed point-by-point response is provided below, and all corresponding changes have been incorporated into the revised manuscript. For ease of review, the revisions have been highlighted in the manuscript.

**Reviewer Comment 1 (Line 27)**: Consider replacing "overindulgence" with a more appropriate word.

**Response:** Thank you for this helpful suggestion. We agree that the term "overindulgence" was not the most appropriate in this context. We have replaced it with "excessive use" throughout the manuscript to improve clarity and ensure consistency with the terminology used in the literature. [See the line no. 24]

**Reviewer Comment 2 (Lines 29 and 48)**: Specify whose specific recommendations you are getting rather than saying participants or population; you have three different types of participants in your study or three populations

**Response**: Thank you for this valuable comment. We have revised the manuscript to clearly specify that the recommendations were provided by parents, teachers, and students, rather than using the general terms "participants" or "population." This improves the precision and clarity of the findings. [See the line no. 26]

**Reviewer Comment 3 (Line 36)**: What different types of in-depth interviews?

**Response**: Thank you for the suggestion. We have clarified that separate in-depth interviews (IDIs) were conducted with parents, teachers, and students to capture the perspectives of each stakeholder group. [See the line no. 32-34]

**Reviewer Comment 4 (Line 36)**: “A thematic analysis was performed using an inductive approach based on the socio-ecological model framework” – an inductive approach cannot be base specifically on a model or framework, this is a deductive approach as a framework is being applied?

**Response**: Thank you for this valuable comment. We agree that applying the socio-ecological model represents a deductive framework. We have revised the manuscript to clarify that **themes were first generated inductively from the data and were subsequently organized and interpreted using the socio-ecological model as an analytical framework**, rather than stating that the inductive approach was based on the framework. [See the line no. 35-37]

**Reviewer Comment 5 (Line 47)**: Parental challenges – please be more specific.

**Response**: Thank you. We have revised the sentence to specify the key parental challenges identified in this study, including monitoring children's screen viewing, supervising age-specific online content, balancing educational and recreational device use, promoting alternative offline activities, and lack of digital literacy among parents. [See the line no. 47]

**Reviewer Comment 6 (Line 55)**: Do you mean inescapable?

**Response:** Thank you for pointing this out. We have replaced the original wording with "inescapable" to improve the accuracy of the sentence. [See the line no. 53]

**Reviewer Comment 7 (Line 60)**: Four factors related to SES were noted but only one described.

**Response**: Thank you for your observation. We have revised this section to clearly describe the four socioeconomic factors identified in the literature, including parenting practices, digital skill expectations, access to digital resources, and opportunities for alternative recreational activities. [See the line no. 58-60]

**Reviewer Comment 8 (Line 65)**: In adolescents?

**Response**: Yes, thank you; we have revised the sentence to specify that the evidence refers to adolescents. [See the line no. 69]

**Reviewer Comment 9 (Line 70)**: In addition to what?

**Response**: We agree with the reviewer and have removed the phrase to improve readability. [See the line no. 72-74]

**Reviewer Comment 10 (Line 72)**: Continue using the term "screen viewing" rather than screen time.

**Response**: Thank you for this suggestion. We have standardized the terminology throughout the manuscript and consistently use screen viewing (SV) except where referring directly to established international guidelines that specifically use the term "screen time."

**Reviewer Comment 11 (Lines 75–77)**: Does this not belong better in the first paragraph where you are talking about what influences technology habits? This second paragraph is about the outcomes of high SV so it doesn’t really align with the argument

**Response**: We agree. The relevant information has been relocated to the preceding paragraph discussing determinants of technology use, resulting in a more logical flow of the Introduction. [See the line no. 64-66]

**Reviewer Comment 12 (Line 81)**: Increased compared to what? Historical use? The WHO recommendations?

**Response**: Thank you. We have revised the sentence with “ A study found that approximately 79% of adolescents exceeded the WHO recommended recreational screen viewing.” [See the line no. 79-81]

**Reviewer Comment 13 (Line 84)**: SV acronym described again.

**Response**: Thank you for pointing this out. We have removed the repeated definition of the acronym "SV" and retained its definition only at its first appearance in the manuscript.

**Reviewer Comment 14 (Lines 87–91)**: Surely the AAP guidelines should be discussed together with the WHO guidelines in the paragraph above? Or bring the WHO guideline here?

**Response**: We appreciate this suggestion. We have reorganized the Introduction to discuss the WHO and American Academy of Pediatrics recommendations together, providing a clearer comparison of international guidance. [See the line no. 81-85]

**Reviewer Comment 15 (Lines 93–108)**: This section seems to be about the challenge’s parents face in managing SV in adolescents if I am right – please look at the evidence presented in this section and synthesize fully? For example, evidence from Canda is presented, then Bangladesh, then HIC contexts – with Canada also being a HIC context, that literature should be synthesised with the latter.

**Response**: Thank you for this valuable recommendation. We have substantially revised this section by synthesizing evidence from high-income and low- and middle-income countries rather than presenting studies sequentially. This revision provides a more coherent overview of parental challenges in managing adolescents' screen viewing. [See the line no. 94-115]

**Reviewer Comment 16 (Line 96)**: SV restrictions can limit opportunities and development. This only really talks to part of what the paper says – HICs are described as more restrictive, and restrictions can reduce opportunity but also increase risk.

In what circumstances? LMIC’s?

**Response**: Thank you for this valuable comment. We have revised the text to provide a more balanced interpretation, clarifying that while overly restrictive screen-viewing practices may limit opportunities for digital learning and development, insufficient restrictions may increase the risk of excessive screen use and its associated adverse outcomes.

We have clarified that the statement refers to low- and middle-income countries experiencing rapid expansion of digital technology, including Bangladesh. [See the line no. 98-103]

**Reviewer Comment 17 (Line 100)**: "The sole challenge" should be "a key challenge."

**Response**: We agree and have revised the wording accordingly. [See the line no. 106]

**Reviewer Comment 18 (Line 108)**: The Indian study is not referenced.

**Response**: Thank you for identifying this omission. The appropriate reference has now been added to both the text and the reference list and the sentence has been paraphrased for better understanding for the reader. [See the line no. 119]

**Reviewer Comment 19 (Line 108 onwards)**: The ongoing section of this paragraph is a new argument and should be a new paragraph I think this seems to be about strategies to manage SV, no longer the challenges that parents face in trying to manage SV in their adolescents?

**Response**: We agree. The paragraph has been divided into two separate paragraphs to distinguish between parental challenges and potential management strategies, thereby improving readability and logical progression. [See the line no. 120-124]

**Reviewer Comment 20 (Line 122)**: From the perspectives of parents, teachers, and 12-14 years old adolescents (not only teachers and adolescents?)

**Response**: Thank you. We have revised the sentence to specify that the study explored the perspectives of parents, teachers, and students aged 12–14 years. [See the line no. 130]

**Reviewer Comment 21 (Line 133)**: A combination of multiple data collection methods (not tools). Data collection methods, not study design, and therefore should not be discussed under this section. Please rather describe the qualitative study here to provide further background to the study design and how the qualitative work fits into this.

**Response**: Thank you for this valuable comment. We have revised this section accordingly by removing the description of the data collection methods from the **Study Design** section. Instead, we now provide additional background on the qualitative study, explaining its purpose, rationale, and how it complements the previous quantitative study. The description of the in-depth interviews (IDIs) and focus group discussions (FGDs) has been moved to the **Data Collection Methods** section, and the term **"data collection methods"** has replaced **"tools"** throughout the manuscript. [See the line no. 134-155]

**Reviewer Comment 22 (Line 138-144)**: There are no references for this section?

**Responses**: Thank you for this comment. We have carefully reviewed this section and added the appropriate references to support the statements and methodological approach described. This revision ensures that all relevant information is adequately referenced in the revised manuscript. The following reference has been added as Reference 22. [See the line no. 138]

**Reviewer Comment 23 (Line 145)**: Start off this section with precisely who your different samples were. This section is on the whole very ‘wordy’ and this makes it a little difficult to follow.

**Response**: Thank you for this helpful suggestion. We have revised the Study participants and sampling section to begin with a clear description of the three participant groups (parents, students, and teachers), their eligibility criteria, and recruitment strategy. We also edited the section to improve clarity and reduce unnecessary detail, making it more concise and easier to follow. [See the line no. 156-183]

**Reviewer Comment 24 (Line 167-171)**: Remove data collection methods from this section, as this related to data collection not sampling.

**Response**: Thank you. We have removed descriptions related to data collection procedures from the sampling section and relocated them to the Data collection and quality checking section to improve the organization of the Methods. [See the line no. 206-231]

**Reviewer Comment 25 (Line 208–209, 212–213)**: Check sentences for clarity/grammar.

**Response**: Thank you. We carefully revised these sentences to improve grammar, readability, and overall clarity while preserving the intended meaning. [See the line no. 180-181 and 226-228]

**Reviewer Comment 26 (Line 217–220)**: This information relates to data analysis and sampling; please rather place in the correct sections, rather than data collection methods.

**Response**: Thank you for pointing this out. The relevant text has been moved from the data collection method to the Study participants and sampling and data analysis section, where it more appropriately belongs. [See the line no. 158 and Data analysis section 232-257]

**Reviewer Comment 27 (Line 227)**: You can describe thematic analysis here already, as familiarization is a part of the process so you effectively already started thematic analysis with familiarization?

**Response**: Thank you. We have revised the Data analysis section to clearly state that thematic analysis began during data familiarization, following Braun and Clarke's approach, and have described the subsequent coding and theme development process accordingly. [See the line no. 238-245, reference no 24]

**Reviewer Comment 28 (Line 237-241)**: Still limited discussion of reflexivity, specifically important when using an inductive approach, and how this relates to rigour.

**Response**: Thank you for this important suggestion. We have expanded the Data analysis section by describing measures taken to enhance rigor, including collaborative coding, triangulation, and acknowledgment of the research team's role during analysis. We have also strengthened the discussion on reflexivity and its contribution to trustworthiness in the inductive analytical process. [See the line no. 246-250]

**Reviewer Comment 29 (Line 255)**: The term ‘gift’ doesn’t sit well in terms of possible coercion and potential for social desirability bias – it would be better to talk about compensation for their time.

**Response**: Thank you. We agree and have replaced the term "gift" with "compensation for their time and transportation expenses" to better reflect the ethical purpose of the reimbursement and avoid any implication of coercion. [See the line no. 270]

**Reviewer Comment 30 (Line 281)**: The description of the themes do not relate to your actual themes.

**Response**: Thank you for identifying this inconsistency. We have revised the introductory paragraph of the Results section so that the descriptions accurately correspond to the final themes presented in the manuscript, including "Challenges faced by parents in monitoring children's screen use." [See the line no. 282-296]

**Reviewer Comment 31(Line 298)**: Is the quote more about a lack of opportunities for family engagement than adolescent preferences for engaging with friends than family?

**Response**: Thank you. We agree and have revised the accompanying narrative to accurately reflect that the quotation illustrates limited family engagement and parental availability rather than adolescents' preference for peers. [now the line no is 313-321]

**Reviewer Comment 32 (Line 316)**: What is eve-teasing?

**Response**: Thank you. We have clarified the term by replacing "eve-teasing" with "sexual harassment in public spaces" to improve clarity for an international readership. [See the line no. 330-333]

**Reviewer Comment 33 (Line 318)**: The quote is not about a lack of availability of peers/cousins, but lack of engagement when they are together?

**Response**: Thank you. We have revised the interpretation to better reflect that the quotation illustrates reduced engagement among peers and cousins due to device use, rather than their absence. [See the line no. 329-333]

**Reviewer Comment 34 (Line 331):** This quote is about digital device usage – not unsupervised access, so I’m not sure if it is good evidence?

**Response**: Thank you. We have replaced the interpretation so that it accurately reflects the content of the quotation. We also selected a more appropriate supporting quotation where necessary to better align with the theme of parental monitoring and supervision. [See the line no. 341-344]

**Reviewer Comment 35 (Line 338)**: Is this not a recommendation?

**Response**: Thank you for this insightful comment. We agree that the original wording was presented as a recommendation rather than a finding. We have revised the text to describe teachers' observations regarding children's exposure to age-inappropriate online content and the challenges related to parental digital literacy and monitoring capacity. The revised narrative now accurately reflects the supporting quotation and is presented as a study finding rather than a recommendation. [See the line no. 341-344]

**Reviewer Comment 36 (Line 386):** This is the impact of excessive SV and not related to the theme?

**Response**: Thank you. We have revised the text to ensure that only findings directly related to the theme are presented. The discussion of the impacts of excessive screen viewing has been relocated to a more appropriate section. [See the line no. 382-389]

**Reviewer Comment 37 (Line 440 onwards)**: Recommendations are still presented as bullet points with limited quotes attached as evidence?

**Response**: Thank you. We have substantially revised the Recommendations section by presenting the findings in narrative form under the socio-ecological framework and integrating illustrative participant quotations to support each recommendation. [See the line no. 451-456, 464-669, 472-475, 477-478]

**Reviewer Comment 38 (Line 462, 509)**: Choose one of the quotes and cut them shorter or put under the appropriate section above.

**Response**: Thank you. We have cut and shortened the quotations by retaining only the most relevant excerpts and relocated them to the most appropriate thematic sections to improve readability and avoid repetition.

**Reviewer Comment 39 (Line 558-560)**: This does not link to the preceding or proceeding paragraph?

**Response**: Thank you. We revised this paragraph to improve its logical flow and ensure that it clearly connects with the surrounding discussion. [See the line no. 579-583]

**Reviewer Comment 40 (Line 585)**: “Many parents reported feeling unprepared to discuss sexual development, which compounded the challenges of supervision.” I don’t think that this was presented in the results?

**Response**: Thank you for your careful observation. We agree that this statement was insufficiently supported by the qualitative findings presented. Therefore, we have removed this statement from the Discussion to ensure that all interpretations are directly grounded in the study results. [See the line no. 608-609]

**Kind regards,**

Dr. Shahria Hafiz Kakon

On behalf of Nahian Sultana, Bidhan Krishna Sarker, Tanjir Rashid Soron, Rashidul Haque, Fahmida Tofail
