# Supplementary material for: Parental monitoring challenges and recommendation for managing excessive screen use among students aged 12–14 years in Dhaka, Bangladesh: A qualitative exploration
Source: PLoS One. 2026 Oct 5;21(10):e0359518. doi: 10.1371/journal.pone.0359518 (PMC13637862; doi:10.1371/journal.pone.0359518)
Supplement: Peer review history

# Peer review: Decision Letter 0

**DOI:** 10.1371/journal.pone.0359518.r001

**Authors:** Derebe Madoro Bunte

9 Aug 2025

Parental monitoring challenges and recommendation for managing excessive screen use among adolescents aged 12–14 years in Dhaka, Bangladesh: a qualitative exploration

Dear Dr.  Kakon,

plosone@plos.org. When you're ready to submit your revision, log on to https://www.editorialmanager.com/pone/ and select the 'Submissions Needing Revision' folder to locate your manuscript file.

- A rebuttal letter that responds to each point raised by the academic editor and reviewer(s). You should upload this letter as a separate file labeled 'Response to Reviewers'.
- A marked-up copy of your manuscript that highlights changes made to the original version. You should upload this as a separate file labeled 'Revised Manuscript with Track Changes'.
- An unmarked version of your revised paper without tracked changes. You should upload this as a separate file labeled 'Manuscript'.

If you would like to make changes to your financial disclosure, please include your updated statement in your cover letter. Guidelines for resubmitting your figure files are available below the reviewer comments at the end of this letter.

If applicable, we recommend that you deposit your laboratory protocols in protocols.io to enhance the reproducibility of your results. Protocols.io assigns your protocol its own identifier (DOI) so that it can be cited independently in the future. For instructions see: https://journals.plos.org/plosone/s/submission-guidelines#loc-laboratory-protocols. Additionally, PLOS ONE offers an option for publishing peer-reviewed Lab Protocol articles, which describe protocols hosted on protocols.io. Read more information on sharing protocols at https://plos.org/protocols?utm_medium=editorial-email&utm_source=authorletters&utm_campaign=protocols.

We look forward to receiving your revised manuscript.

Kind regards,

Derebe Madoro Bunte,

Academic Editor

PLOS ONE

Journal Requirements:

When submitting your revision, we need you to address these additional requirements.

1.Please ensure that your manuscript meets PLOS ONE's style requirements, including those for file naming. The PLOS ONE style templates can be found at

https://journals.plos.org/plosone/s/file?id=wjVg/PLOSOne_formatting_sample_main_body.pdf and

https://journals.plos.org/plosone/s/file?id=ba62/PLOSOne_formatting_sample_title_authors_affiliations.pdf

2. You indicated that you had ethical approval for your study. In your Methods section, please ensure you have also stated whether you obtained consent from parents or guardians of the minors included in the study or whether the research ethics committee or IRB specifically waived the need for their consent.

3. We note that the grant information you provided in the ‘Funding Information’ and ‘Financial Disclosure’ sections do not match.

When you resubmit, please ensure that you provide the correct grant numbers for the awards you received for your study in the ‘Funding Information’ section.

4. Thank you for stating the following financial disclosure:

“Bangladesh government awarded project named Mujib 100 research Grant for women. I have received this award on 2022. Funded was $ 43,250 for 2 years project”

Please state what role the funders took in the study.  If the funders had no role, please state: "The funders had no role in study design, data collection and analysis, decision to publish, or preparation of the manuscript."

If this statement is not correct you must amend it as needed.

Please include this amended Role of Funder statement in your cover letter; we will change the online submission form on your behalf.

5. Please note that funding information should not appear in any section or other areas of your manuscript. We will only publish funding information present in the Funding Statement section of the online submission form. Please remove any funding-related text from the manuscript.

6. Please provide a complete Data Availability Statement in the submission form, ensuring you include all necessary access information or a reason for why you are unable to make your data freely accessible. If your research concerns only data provided within your submission, please write "All data are in the manuscript and/or supporting information files" as your Data Availability Statement.

7. When completing the data availability statement of the submission form, you indicated that you will make your data available on acceptance. We strongly recommend all authors decide on a data sharing plan before acceptance, as the process can be lengthy and hold up publication timelines. Please note that, though access restrictions are acceptable now, your entire data will need to be made freely accessible if your manuscript is accepted for publication. This policy applies to all data except where public deposition would breach compliance with the protocol approved by your research ethics board. If you are unable to adhere to our open data policy, please kindly revise your statement to explain your reasoning and we will seek the editor's input on an exemption. Please be assured that, once you have provided your new statement, the assessment of your exemption will not hold up the peer review process.

8. Please include your full ethics statement in the ‘Methods’ section of your manuscript file. In your statement, please include the full name of the IRB or ethics committee who approved or waived your study, as well as whether or not you obtained informed written or verbal consent. If consent was waived for your study, please include this information in your statement as well.

9. If the reviewer comments include a recommendation to cite specific previously published works, please review and evaluate these publications to determine whether they are relevant and should be cited. There is no requirement to cite these works unless the editor has indicated otherwise.

Additional Editor Comments:

- The manuscript requires substantial revision before acceptance, with mandatory changes including clarification of study design and methodology (operational definitions, inclusion/exclusion criteria, recruitment procedures, role of teachers, separation of FGDs and IDIs, complete demographic reporting), detailed ethical procedures, and rigorous description of the analysis process with supporting quotes for all themes. Recommended (non-essential) changes include restructuring recommendations using an ecological model, adding SES-linked analysis, presenting exemplary quotes in tables, and improving contextual relevance with local guidelines. While all reviewers agree on the topic’s importance and the value of the findings, they consistently highlight gaps in methodological transparency, thematic presentation, and language clarity, with no direct conflicts—thus authors should follow the converging advice to strengthen rigor, synthesis, and readability.

Reviewer's comments

Comments to the Author

1. Is the manuscript technically sounds, and do the data support the conclusions?

The manuscript must describe a technically sound piece of scientific research with data that supports the conclusions. Experiments must have been conducted rigorously, with appropriate controls, replication, and sample sizes. The conclusions must be drawn appropriately based on the data presented.

#Reviewer-1 Partly

#Reviewer-2 Yes

##Reviewer-3 Partly

2. Has the statistical analysis been performed appropriately and rigorously?

#Reviewer-1 N/A

#Reviewer-2 N/A

##Reviewer-3 No

3. Have the authors made all data underlying the findings in their manuscript fully available?

The PLOS Data policy requires authors to make all data underlying the findings described in their manuscript fully available without restriction, with rare exception (please refer to the Data Availability Statement in the manuscript PDF file). The data should be provided as part of the manuscript or its supporting information, or deposited to a public repository. For example, in addition to summary statistics, the data points behind means, medians and variance measures should be available. If there are restrictions on publicly sharing data—e.g. participant privacy or use of data from a third party—those must be specified. Yes No

#Reviewer-1 Yes

#Reviewer-2 No

##Reviewer-3 Yes

4. Is the manuscript presented in an intelligible fashion and written in standard English?

PLOS ONE does not copyedit accepted manuscripts, so the language in submitted articles must be clear, correct, and unambiguous. Any typographical or grammatical errors should be corrected at revision, so please note any specific errors here.

#Reviewer-1 No

#Reviewer-2 No

##Reviewer-3 No

5. Review Comments to the Author

Please use the space provided to explain your answers to the questions above. You may also include additional comments for the author, including concerns about dual publication, research ethics, or publication ethics. (Please upload your review as an attachment if it exceeds 20,000 characters)

#Reviewer-1

Thank you for your submission.

Overall, this paper addresses what seems to be a key concern. The paper appears to do what it aimed to do, but the portrayal of the results and discussion hinders its impact, and the language and construction of the paper hinders readability. Literature needs to be further synthesized and used effectively to draw conclusions and develop arguments in introduction and discussion. With reworking and refining concerns, this could be a publishable paper.

Introduction:

- Grammar and word use not always appropriate for an academic article or unclear. (Lines 67, 74, 83, 84, 85, 86, 89, 101, 104, 112, 115) Overall the introduction could improve in flow, conciseness and effectiveness.

- The review of literature felt like a side-by-side review of the different studies, rather than synthesising the information into specific sections to build into an argument to justify this paper. Justification for this study and the gap in literature was not fully developed in the introduction, therefore difficult to ascertain. Although there was a description of limited data on the topic, this was not argued so difficult to ascertain.

- I would suggest that this section is reworked to synthesise the evidence to make a stronger case for the evidence gap and justification for this study.

Aim:

- Remember we don't examine in qualitative work, but rather explore or understand, describe etc.

- 'gather specific feedback on how to effectively utilize SV within urban Bangladesh context' doesn't make sense - for adolescent use? to create a family plan for SV? The second aim statement in the methods section makes much more sense.

Design:

- A cross sectional design is linked to quantitative work, not qual. This appears to be a descriptive qualitative study. Please use references.

Setting:

- Please provide a description of the setting (i.e. North and South Dhaka City Corporations in Bangladesh) so that we have an understanding of the context which is vital for rigour, specifically transferability.

Sampling and recruitment:

Clear justification for why specific participants were chosen and heterogenous sampling was used. However, your recruitment strategy was unclear and the eligibility criteria was not apparent.

- How did you identify the schools from which to sample the children from? I see that you aimed for a heterogenous sample, but what criteria specifically did you use to select and approach these schools?

- How did you identify children and parents (of children) with excess screen use? How did you recruit them into the study? What were the age children were you sampling and why?

- Were parents and children from the same families interviewed? How did you negotiate the ethics around this?

- Please ensure that you separate sampling and data collection procedures as this hinders the flow of the paper and makes for confusing reading. Data saturation has to do with the number of interviews and data collection, not sample size. Please review this section. We don’t need to make a case for sample size when using purposive sampling, but rather focus on the eligibility criteria which guided your sampling of the participants.

Data collection:

- You clarified the number of IDIs and FGDs you used, who they were with, where they were run, the language, the facilitators and how you developed the guides.

- Please use references to support your processes?

- Please consolidate the data collection methods under one concise section, as this is not a streamlined passage.

- Methods seem appropriate for design. Would have been nice to see why these methods are useful to harness participants perspectives/experiences etc. or linked to the aim.

- 1.5 hrs appears very long for the children. What other strategies did you use to ensure that the data collection was rigorous for this specific age group? Looking at power differences etc. I am wondering how having the interviews at school could have influenced data collection as it is a place where children are safe, but not ‘powerful’.

Rigour:

- Please refer to the sources you used to guide this process? It appears that quantitative principles are being used to guide rigour strategies for qualitative work, which is confusing from a paradigm perspective and doesn’t achieve its purpose.

- Rigour in data collection needs refinement, I would strongly urge you to refer to Lincoln and Guba's four constructs. This article refers to rigour in qualitative analysis specifically, however the principles can be applied to the project as a whole:

International Journal of Qualitative Methods Thematic Analysis: Striving to Meet the Trustworthiness Criteria Volume 16: 1–13 DOI: 10.1177/160940691773384.

Analysis:

- Please refer to sources used to guide this process?

- Outline the type of analysis used and the steps upfront (with reference).

- Clarify how you used the codebook and matrixes?

- No mention of reflexivity, this is vital in qualitative work to make your approach clear.

Ethics:

- Were any specific ethical approaches needed as the study was working with children? How were these approached?

- Add reimbursement to data collection for smooth reading

Results:

- It is not clear that table 1 only refers to student’s characteristics. Why were characteristics of the other participants not reported?

- Lacking clarity

- An initial overview of the themes related to challenges faced by parents needs to be provided. i.e. 4 themes were identified that consisted of…

- It is expected that the information from the IDI and FGDs is synthesized as a part of thematic analysis, not presented separately, so this doesn’t need to be explained.

- Why are the recommendations’ themes written as bullet points and do not follow the same process as the challenges with quotes to illustrate your point. The same structure should be used? These are still themes that you identified and need evidence. This section needs to be reworked to follow a similar structure and it would seem that some of the items fall under the same theme. I would consider using the ecological model to structure this section and place recommendations under the different levels as themes (i.e. micro, meso and macro) Please see this as an example:

Harper CR, Steiner RJ, Brookmeyer KA. Using the Social-Ecological Model to Improve Access to Care for Adolescents and Young Adults. J Adolesc Health. 2018 Jun;62(6):641-642. doi: 10.1016/j.jadohealth.2018.03.010. PMID: 29784107; PMCID: PMC10898617.

- General clarity, sentence structure, word usage and grammar needs reworking.

- Evidence/quote provided does not always match statement that it is linked to. (i.e. line 289)

- In the first section (challenges), children, parents and teachers often say the same thing. It would be more impactful for me if these topics are discussed with the evidence from the participants synthesised together, with only their unique contributions/differences discussed separately in another paragraph with evidence, rather than presenting each group of participants results separately under each heading.

- Why are some words in italics? Are these direct quotes? I would suggest using quote marks to make this clear.

Discussion:

- General clarity, sentence structure, word usage and grammar needs reworking.

- This section needs reworking – rather than reporting what other evidence notes, you need to use this other research to link and make recommendations from your own research. It is fine to draw the similarities between your and other research as a mechanism to situate your evidence, but rather than report what other studies found, use it to build the discussion around your evidence.

- Limitations

o why didn’t you have enough time to do separate FGDs?

o The context of a study is not a limitation, rather provide a think description of the setting so that results are transferable to similar settings.

o Qualitative research does not aim to be generalisable and therefore this is not a limitation of the study

#Reviewer-2.

1. check typos in line 77, 204

2. put phrases or acronyms in uniform, line 171 IDI so line 174,177 too.

3. line 180-183, please have a look and rephrase

4. please clarify the participants and the numbers of participants in the line 160 and so forth. 5. line 216: clarify multiple sources. what are the three types of data collection tools

6. What is your measure to determine "excessive use of internet or screen view in this study?"

#Reviewer-3

This study tackles an important and relevant topic with valuable insights and practical recommendations that could significantly contribute to the field. The authors have conducted meaningful research that addresses real challenges faced by parents in managing adolescent screen use. However, the manuscript requires substantial methodological clarification and improved presentation of results to meet publication standards. The findings and recommendations are strong, but the research would benefit from more rigorous reporting and clearer methodology to enhance its impact and reproducibility.

Article Summary: This qualitative study explores the challenges parents face in monitoring their adolescents' (aged 12-14) screen use in Dhaka, Bangladesh. Using focus group discussions, in-depth interviews, and key informant interviews with 25 children, parents, and teachers from six schools, the study identifies four main challenges: educational necessity of devices creating supervision difficulties, restricted outdoor activities increasing device dependence, exposure to age-inappropriate content, and parental digital illiteracy. The study provides comprehensive recommendations including school-based interventions, national-level policies, enhanced parent-child bonding, and secured infrastructure development.

Strengths:

• Comprehensive multi-stakeholder approach: The inclusion of children, parents, and teachers provides valuable triangulation of perspectives on screen use challenges

• Practical and actionable recommendations: The study offers concrete suggestions for schools, parents, and policymakers that are contextually relevant to the Bangladeshi setting

• Important and timely research topic: Addresses a critical public health concern regarding adolescent screen use in a rapidly digitizing society

• Rich qualitative data: The combination of FGDs, IDIs, and KIIs provides depth and breadth to understanding the phenomenon

• Cultural contextualization: Appropriately situates findings within the social and educational context of Dhaka, Bangladesh

Weaknesses:

Major Concerns

• Lack of clear operational definitions

• Insufficient inclusion/exclusion criteria

• Unclear data collection procedures

• Inadequate sample size justification

• Missing inter-rater reliability measures

• Incomplete demographic reporting

• Incomplete consent procedures

Minor Concerns

• Grammar issues

• Presentation and organization

Suggestions for Improvement:

• Suggest using a grammar checker and proofreading – a lot of misspellings and run on sentences which make it hard to read the paper

• Introduction –

o Paragraph 1 - link between parenting and SES is established but it is unclear as to how parenting relates to health of adolescents � explicitly state that parenting style is a mediator of technology use and thus, health of adolescents

o line 95 – please mention whether the screen time usage is a mean or median measure

o line 105 – author’s mention AAP. The study context is Dhaka, Bangladesh, are there any Bangladeshi guidelines for pediatrics that should be mentioned? It will help strengthen the context and be directly applicable

o Currently, paragraphs 1,2, 3 are not logically organized – suggestion for organization � paragraph 1: clearly outline the problems on health brought forth by SV; paragraph 2: outline the literature for why parents are intentionally/unintentionally using technology to help with parenting; paragraph 3: outline existing pediatric guidelines and perspectives for healthier management of technology use among adolescents

• Materials and Methods

o Line 160 – please quantify “excessive use” of digital devices using a threshold (for example, > 5 hours/day, etc.)

o Lines 160 – Lines 167 – please be specific with inclusion and exclusion criteria for the study. Please list the minimum age inclusion/exclusion for each participant, along with any other inclusion/exclusion criteria that was relevant to the study (e.g. technology use, language, family size, etc.) Please try to be specific in this section.

o Line 165 – while teacher’s play a role in children’s development and education, it is unclear as to how they would be valuable as key informants for the aim of the research as they are not providing child care; the introduction argues that SV has come on as a way of making caregiving easier. Please include some literature/citations that can help justify the inclusion of teachers as I do agree that they are an important perspective to consider

o Line 171 – please define how these “class register books” were obtained

o Regarding focus groups – were children and their parents both present in the same focus groups? This poses a concern for social desirability bias on the end of the children to appease to their parents. Please clarify if parents and children had separate focus groups (parents only and children only). If parents and children were present in the same focus group, please justify why this decision was made and how bias arising from potential social desirability bias was avoided.

o Lines 180 – 184 – please condense language to reflect that interviews and focus groups were conducted until thematic saturation was reached.

o Line 185 – Please clarify the 18 interviews – I am only counting 12 interviews (5 students, 3 parents, and 4 key informant teacher interviews); please note that focus group discussions should not be included in the interview sample size and should be listed separately. E.g., “A total of 12 interviews and 6 FGDs were conducted.” Additionally, please list the sample size, N for easier reading.

o Table 1 – Please change the “School Types” column to be more reflective of categorization based on language of teaching as “school types” suggest classification based on funding – private v. public.

o Line 191 – Please include/indicate how sociodemographic data was collected from participants.

o Line 193 – Please include guidelines in the appendix. Alternatively, provide brief descriptions of the contents of the guidelines – did they include suggestions for how long interviews should last? Did they include any probes? How many questions were there? What themes did the interview/FGD questions explore, if any?

o Line 197 – please update the sentence to state “three and five years of experience on qualitative data collection, respectively.”

o Lines 200 – 203 – Which language were the interviews/FGDs conducted in? Were the interviews/FGD conducted in English at English-medium schools and in Bangla at Bangla-medium schools? Please clarify

o Line 201 – how long were key informant interviews?

o Line 208 – Please use gender neutral pronouns to describe the facilitator

o Line 205 – Please clarify if the researcher with the recorder also took notes.

o Lines 213- 218 – How was data validity maintained/triangulated? Were the same participants interviewed multiple times? Were parent – child dyads tracked for this triangulation? This section is very unclear – please provide more details regarding the methodology, maybe including a figure would be helpful.

o Lines 219 – 220 – Data interpretation should be talked about in the analysis section, please remove this sentence.

o Line 221 – Please clarify validating data cross various contexts. Validity of data is usually not a concern in one-time interviews or focus group participations. There are usually limitations of self-report or social desirability biases, but it is unrealistic to have responses free of bias in qualitative studies. Who/what is this data being validated against. Is there a standard respondent? Alternatively, are the authors checking for quality of data, i.e., easy to comprehend and bug-free recordings or recordings that are easy to download/transcribe? Please clarify.

o Line 226 – please provide an example file nomenclatures; e.g. “Participant001_English_IDI” or something along those lines that can let readers know of how files were stored in case they want to replicate.

o Line 228 – Earlier it is mentioned that interviews were recorded, but there is now a note implying that some interviews were not recorded. Please explain.

o Line 231 – Please clarify if there were any consensus meetings to finalize the codebook. Consensus for codebook development is a foundational part of qualitative analysis. Additionally, please include the final codebook in the appendix as it will be helpful to other investigators conducting similar studies.

o Line 232– Please list the initials of those involved in the coding process (analysis team).

o Lines 236-238 – Rather than validity of data, I am more interested in the inter-rater reliability. Please clarify the exact coding methods – was a double-coding or single-coding method used. Or were a select few transcripts coded by all the analysis team members at the start to ensure consistency in coding before single-coding. Alternatively, did all the analysis team members code all the transcripts? Additionally, please provide Cohen’s kappa for a measure of inter-rater reliability.

o Line 241 – As children (<18) were involved in the study, please elaborate on their consent process. Was parental assent required?

o Line 249 – Where were the recordings/data stored? Please provide a location e.g. “password protected computer only accessible by the research team.” Additionally, were the interviews themselves de-identified? Please clarify.

o Line 250 – Please specify the token of appreciation amount (in Bangladeshi currency and USD).

• Results –

o Line 253 – Please note that Table 2 only provides the demographics for children who participated in the study (from what I gather). Please update language to reflective of this specific demographic. As parents and teachers were also interviewed/included in the study, please add additional tables for demographic characteristics of each of these groups. Alternatively, you can create one large table and have subgroup frequencies included as columns (column 1 = children, column 2 = parents, column 3 = teachers)

o Line 263 – 268 – please provide your definition of screen viewing and then provide a summary of responses/content analysis by participant subgroup that relate to screen viewing as a concept. To strengthen this paragraph, you can also summarize any quotes or general remarks that point towards relations between screen viewing and health.

o Lines 325 – 332 – please provide more context using literature or another source to contextualize the social climate in Dhaka.

o Lines 394 – 403 – please provide an exemplary quote to support the claim being made. It is interesting and I would like to read what was said.

o Line 410 – please check the formatting of the quote.

o Line 431 – please include a transition paragraph that segways into the new subsection of recommendations. Also, please consider condensing these recommendations and moving them to the discussion section or including them as a table. Great work on the recommendations!

o Line 484 – Please rename the subheading to “ensure social safety”

o General note – I am missing quotes or analysis based on SES status as that seemed to be a big factor influencing health of adolescents and technology use according to the introduction written by the author’s. Please include 1 or 2 quotes that highlight any relationships of SES, technology use, and health/concerns.

• Discussion –

o Lines 533 – 537 – I appreciate the domains/points brought up regarding SV (e.g. not all screens are equal). It would potentially be helpful to include a table in the results section listing exemplary quotes organized by these domains for easier comprehension.

o Line 567 – please elaborate. The paragraph is only one sentence currently.

o Line 583 – what were the results of the clustered randomized controlled trial?

o Line 608 – I like the call to action being presented here. To tighten narrative, please tie it in or include how existing pediatric guidelines could be modified. Alternatively, should we consider devising new pediatric guidelines? Curious to hear your thoughts.

o Under limitations, please also include the influence of social desirability biases among participants or potential selection for responses given the skew towards Bangla-medium participants in the study. Additionally, please include limitations related to potential human error (such as not recording interviews, errors in translating from Bangla to English or vice versa).

6. PLOS authors have the option to publish the peer review history of their article (what does this mean?). If published, this will include your full peer review and any attached files.

If you choose “no”, your identity will remain anonymous but your review may still be made public.

Do you want your identity to be public for this peer review? For information about this choice, including consent withdrawal, please see our Privacy Policy.

#Reviewer-1 No

#Reviewer-2 No

#Reviewer-3 No

[Note: HTML markup is below. Please do not edit.]

Reviewer's Responses to Questions

**Comments to the Author**

1. Is the manuscript technically sound, and do the data support the conclusions?

Reviewer #1: Partly

Reviewer #2: Yes

Reviewer #3: Partly

**********

2. Has the statistical analysis been performed appropriately and rigorously?

Reviewer #1: N/A

Reviewer #2: N/A

Reviewer #3: No

**********

3. Have the authors made all data underlying the findings in their manuscript fully available?

The PLOS Data policy

Reviewer #1: Yes

Reviewer #2: No

Reviewer #3: Yes

**********

4. Is the manuscript presented in an intelligible fashion and written in standard English?

Reviewer #1: No

Reviewer #2: No

Reviewer #3: No

**********

Reviewer #1: Thank you for your submission.

Overall, this paper addresses what seems to be a key concern. The paper appears to do what it aimed to do, but the portrayal of the results and discussion hinders its impact, and the language and construction of the paper hinders readability. Literature needs to be further synthesized and used effectively to draw conclusions and develop arguments in introduction and discussion. With reworking and refining concerns, this could be a publishable paper.

Introduction:

- Grammar and word use not always appropriate for an academic article or unclear. (Lines 67, 74, 83, 84, 85, 86, 89, 101, 104, 112, 115) Overall the introduction could improve in flow, conciseness and effectiveness.

- The review of literature felt like a side-by-side review of the different studies, rather than synthesising the information into specific sections to build into an argument to justify this paper. Justification for this study and the gap in literature was not fully developed in the introduction, therefore difficult to ascertain. Although there was a description of limited data on the topic, this was not argued so difficult to ascertain.

- I would suggest that this section is reworked to synthesise the evidence to make a stronger case for the evidence gap and justification for this study.

Aim:

- Remember we don't examine in qualitative work, but rather explore or understand, describe etc.

- 'gather specific feedback on how to effectively utilize SV within urban Bangladesh context' doesn't make sense - for adolescent use? to create a family plan for SV? The second aim statement in the methods section makes much more sense.

Design:

- A cross sectional design is linked to quantitative work, not qual. This appears to be a descriptive qualitative study. Please use references.

Setting:

- Please provide a description of the setting (i.e. North and South Dhaka City Corporations in Bangladesh) so that we have an understanding of the context which is vital for rigour, specifically transferability.

Sampling and recruitment:

- Clear justification for why specific participants were chosen and heterogenous sampling was used. However, your recruitment strategy was unclear and the eligibility criteria was not apparent.

- How did you identify the schools from which to sample the children from? I see that you aimed for a heterogenous sample, but what criteria specifically did you use to select and approach these schools?

- How did you identify children and parents (of children) with excess screen use? How did you recruit them into the study? What were the age children were you sampling and why?

- Were parents and children from the same families interviewed? How did you negotiate the ethics around this?

- Please ensure that you separate sampling and data collection procedures as this hinders the flow of the paper and makes for confusing reading. Data saturation has to do with the number of interviews and data collection, not sample size. Please review this section. We don’t need to make a case for sample size when using purposive sampling, but rather focus on the eligibility criteria which guided your sampling of the participants.

Data collection:

- You clarified the number of IDIs and FGDs you used, who they were with, where they were run, the language, the facilitators and how you developed the guides.

- Please use references to support your processes?

- Please consolidate the data collection methods under one concise section, as this is not a streamlined passage.

- Methods seem appropriate for design. Would have ben nice to see why these methods are useful to harness participants perspectives/experiences etc. or linked to the aim.

- 1.5 hrs appears very long for the children. What other strategies did you use to ensure that the data collection was rigorous for this specific age group? Looking at power differences etc. I am wondering how having the interviews at school could have influenced data collection as it is a place where children are safe, but not ‘powerful’.

Rigour:

- Please refer to the sources you used to guide this process? It appears that quantitative principles are being used to guide rigour strategies for qualitative work, which is confusing from a paradigm perspective and doesn’t achieve its purpose.

- Rigour in data collection needs refinement, I would strongly urge you to refer to Lincoln and Guba's four constructs. This article refers to rigour in qualitative analysis specifically, however the principles can be applied to the project as a whole:

International Journal of Qualitative Methods Thematic Analysis: Striving to Meet the Trustworthiness Criteria Volume 16: 1–13 DOI: 10.1177/160940691773384

Analysis:

- Please refer to sources used to guide this process?

- Outline the type of analysis used and the steps upfront (with reference).

- Clarify how you used the codebook and matrixes?

- No mention of reflexivity, this is vital in qualitative work to make your approach clear.

Ethics:

- Were any specific ethical approaches needed as the study was working with children? How were these approached?

- Add reimbursement to data collection for smooth reading

Results:

- It is not clear that table 1 only refers to student’s characteristics. Why were characteristics of the other participants not reported?

- Lacking clarity

- An initial overview of the themes related to challenges faced by parents needs to be provided. i.e. 4 themes were identified that consisted of…

- It is expected that the information from the IDI and FGDs is synthesized as a part of thematic analysis, not presented separately, so this doesn’t need to be explained.

- Why are the recommendations’ themes written as bullet points and do not follow the same process as the challenges with quotes to illustrate your point. The same structure should be used? These are still themes that you identified and need evidence. This section needs to be reworked to follow a similar structure and it would seem that some of the items fall under the same theme. I would consider using the ecological model to structure this section and place recommendations under the different levels as themes (i.e. micro, meso and macro) Please see this as an example:

Harper CR, Steiner RJ, Brookmeyer KA. Using the Social-Ecological Model to Improve Access to Care for Adolescents and Young Adults. J Adolesc Health. 2018 Jun;62(6):641-642. doi: 10.1016/j.jadohealth.2018.03.010. PMID: 29784107; PMCID: PMC10898617.

- General clarity, sentence structure, word usage and grammar needs reworking.

- Evidence/quote provided does not always match statement that it is linked to. (i.e. line 289)

- In the first section (challenges), children, parents and teachers often say the same thing. It would be more impactful for me if these topics are discussed with the evidence from the participants synthesised together, with only their unique contributions/differences discussed separately in another paragraph with evidence, rather than presenting each group of participants results separately under each heading.

- Why are some words in italics? Are these direct quotes? I would suggest using quote marks to make this clear.

Discussion:

- General clarity, sentence structure, word usage and grammar needs reworking.

- This section needs reworking – rather than reporting what other evidence notes, you need to use this other research to link and make recommendations from your own research. It is fine to draw the similarities between your and other research as a mechanism to situate your evidence, but rather than report what other studies found, use it to build the discussion around your evidence.

- Limitations

o why didn’t you have enough time to do separate FGDs?

o The context of a study is not a limitation, rather provide a think description of the setting so that results are transferable to similar settings.

o Qualitative research does not aim to be generalisable and therefore this is not a limitation of the study

Reviewer #2: 1. check typos in line 77, 204

2. put phrases or acronyms in uniform, line 171 IDI so line 174,177 too.

3. line 180-183, please have a look and rephrase

4. please clarify the participants and the numbers of participants in the line 160 and so forth.

5. line 216: clarify multiple sources. what are the three types of data collection tools

6. What is your measure to determine "excessive use of internet or screen view in this study?"

Reviewer #3: This study tackles an important and relevant topic with valuable insights and practical recommendations that could significantly contribute to the field. The authors have conducted meaningful research that addresses real challenges faced by parents in managing adolescent screen use. However, the manuscript requires substantial methodological clarification and improved presentation of results to meet publication standards. The findings and recommendations are strong, but the research would benefit from more rigorous reporting and clearer methodology to enhance its impact and reproducibility.

Article Summary: This qualitative study explores the challenges parents face in monitoring their adolescents' (aged 12-14) screen use in Dhaka, Bangladesh. Using focus group discussions, in-depth interviews, and key informant interviews with 25 children, parents, and teachers from six schools, the study identifies four main challenges: educational necessity of devices creating supervision difficulties, restricted outdoor activities increasing device dependence, exposure to age-inappropriate content, and parental digital illiteracy. The study provides comprehensive recommendations including school-based interventions, national-level policies, enhanced parent-child bonding, and secured infrastructure development.

Strengths:

• Comprehensive multi-stakeholder approach: The inclusion of children, parents, and teachers provides valuable triangulation of perspectives on screen use challenges

• Practical and actionable recommendations: The study offers concrete suggestions for schools, parents, and policymakers that are contextually relevant to the Bangladeshi setting

• Important and timely research topic: Addresses a critical public health concern regarding adolescent screen use in a rapidly digitizing society

• Rich qualitative data: The combination of FGDs, IDIs, and KIIs provides depth and breadth to understanding the phenomenon

• Cultural contextualization: Appropriately situates findings within the social and educational context of Dhaka, Bangladesh

Weaknesses:

Major Concerns

• Lack of clear operational definitions

• Insufficient inclusion/exclusion criteria

• Unclear data collection procedures

• Inadequate sample size justification

• Missing inter-rater reliability measures

• Incomplete demographic reporting

• Incomplete consent procedures

Minor Concerns

• Grammar issues

• Presentation and organization

Suggestions for Improvement:

• Suggest using a grammar checker and proofreading – a lot of misspellings and run on sentences which make it hard to read the paper

• Introduction –

o Paragraph 1 - link between parenting and SES is established but it is unclear as to how parenting relates to health of adolescents � explicitly state that parenting style is a mediator of technology use and thus, health of adolescents

o line 95 – please mention whether the screen time usage is a mean or median measure

o line 105 – author’s mention AAP. The study context is Dhaka, Bangladesh, are there any Bangladeshi guidelines for pediatrics that should be mentioned? It will help strengthen the context and be directly applicable

o Currently, paragraphs 1,2, 3 are not logically organized – suggestion for organization � paragraph 1: clearly outline the problems on health brought forth by SV; paragraph 2: outline the literature for why parents are intentionally/unintentionally using technology to help with parenting; paragraph 3: outline existing pediatric guidelines and perspectives for healthier management of technology use among adolescents

• Materials and Methods

o Line 160 – please quantify “excessive use” of digital devices using a threshold (for example, > 5 hours/day, etc.)

o Lines 160 – Lines 167 – please be specific with inclusion and exclusion criteria for the study. Please list the minimum age inclusion/exclusion for each participant, along with any other inclusion/exclusion criteria that was relevant to the study (e.g. technology use, language, family size, etc.) Please try to be specific in this section.

o Line 165 – while teacher’s play a role in children’s development and education, it is unclear as to how they would be valuable as key informants for the aim of the research as they are not providing child care; the introduction argues that SV has come on as a way of making caregiving easier. Please include some literature/citations that can help justify the inclusion of teachers as I do agree that they are an important perspective to consider

o Line 171 – please define how these “class register books” were obtained

o Regarding focus groups – were children and their parents both present in the same focus groups? This poses a concern for social desirability bias on the end of the children to appease to their parents. Please clarify if parents and children had separate focus groups (parents only and children only). If parents and children were present in the same focus group, please justify why this decision was made and how bias arising from potential social desirability bias was avoided.

o Lines 180 – 184 – please condense language to reflect that interviews and focus groups were conducted until thematic saturation was reached.

o Line 185 – Please clarify the 18 interviews – I am only counting 12 interviews (5 students, 3 parents, and 4 key informant teacher interviews); please note that focus group discussions should not be included in the interview sample size and should be listed separately. E.g., “A total of 12 interviews and 6 FGDs were conducted.” Additionally, please list the sample size, N for easier reading.

o Table 1 – Please change the “School Types” column to be more reflective of categorization based on language of teaching as “school types” suggest classification based on funding – private v. public.

o Line 191 – Please include/indicate how sociodemographic data was collected from participants.

o Line 193 – Please include guidelines in the appendix. Alternatively, provide brief descriptions of the contents of the guidelines – did they include suggestions for how long interviews should last? Did they include any probes? How many questions were there? What themes did the interview/FGD questions explore, if any?

o Line 197 – please update the sentence to state “three and five years of experience on qualitative data collection, respectively.”

o Lines 200 – 203 – Which language were the interviews/FGDs conducted in? Were the interviews/FGD conducted in English at English-medium schools and in Bangla at Bangla-medium schools? Please clarify

o Line 201 – how long were key informant interviews?

o Line 208 – Please use gender neutral pronouns to describe the facilitator

o Line 205 – Please clarify if the researcher with the recorder also took notes.

o Lines 213- 218 – How was data validity maintained/triangulated? Were the same participants interviewed multiple times? Were parent – child dyads tracked for this triangulation? This section is very unclear – please provide more details regarding the methodology, maybe including a figure would be helpful.

o Lines 219 – 220 – Data interpretation should be talked about in the analysis section, please remove this sentence.

o Line 221 – Please clarify validating data cross various contexts. Validity of data is usually not a concern in one-time interviews or focus group participations. There are usually limitations of self-report or social desirability biases, but it is unrealistic to have responses free of bias in qualitative studies. Who/what is this data being validated against. Is there a standard respondent? Alternatively, are the authors checking for quality of data, i.e., easy to comprehend and bug-free recordings or recordings that are easy to download/transcribe? Please clarify.

o Line 226 – please provide an example file nomenclatures; e.g. “Participant001_English_IDI” or something along those lines that can let readers know of how files were stored in case they want to replicate.

o Line 228 – Earlier it is mentioned that interviews were recorded, but there is now a note implying that some interviews were not recorded. Please explain.

o Line 231 – Please clarify if there were any consensus meetings to finalize the codebook. Consensus for codebook development is a foundational part of qualitative analysis. Additionally, please include the final codebook in the appendix as it will be helpful to other investigators conducting similar studies.

o Line 232– Please list the initials of those involved in the coding process (analysis team).

o Lines 236-238 – Rather than validity of data, I am more interested in the inter-rater reliability. Please clarify the exact coding methods – was a double-coding or single-coding method used. Or were a select few transcripts coded by all the analysis team members at the start to ensure consistency in coding before single-coding. Alternatively, did all the analysis team members code all the transcripts? Additionally, please provide Cohen’s kappa for a measure of inter-rater reliability.

o Line 241 – As children (<18) were involved in the study, please elaborate on their consent process. Was parental assent required?

o Line 249 – Where were the recordings/data stored? Please provide a location e.g. “password protected computer only accessible by the research team.” Additionally, were the interviews themselves de-identified? Please clarify.

o Line 250 – Please specify the token of appreciation amount (in Bangladeshi currency and USD).

• Results –

o Line 253 – Please note that Table 2 only provides the demographics for children who participated in the study (from what I gather). Please update language to reflective of this specific demographic. As parents and teachers were also interviewed/included in the study, please add additional tables for demographic characteristics of each of these groups. Alternatively, you can create one large table and have subgroup frequencies included as columns (column 1 = children, column 2 = parents, column 3 = teachers)

o Line 263 – 268 – please provide your definition of screen viewing and then provide a summary of responses/content analysis by participant subgroup that relate to screen viewing as a concept. To strengthen this paragraph, you can also summarize any quotes or general remarks that point towards relations between screen viewing and health.

o Lines 325 – 332 – please provide more context using literature or another source to contextualize the social climate in Dhaka.

o Lines 394 – 403 – please provide an exemplary quote to support the claim being made. It is interesting and I would like to read what was said.

o Line 410 – please check the formatting of the quote.

o Line 431 – please include a transition paragraph that segways into the new subsection of recommendations. Also, please consider condensing these recommendations and moving them to the discussion section or including them as a table. Great work on the recommendations!

o Line 484 – Please rename the subheading to “ensure social safety”

o General note – I am missing quotes or analysis based on SES status as that seemed to be a big factor influencing health of adolescents and technology use according to the introduction written by the author’s. Please include 1 or 2 quotes that highlight any relationships of SES, technology use, and health/concerns.

• Discussion –

o Lines 533 – 537 – I appreciate the domains/points brought up regarding SV (e.g. not all screens are equal). It would potentially be helpful to include a table in the results section listing exemplary quotes organized by these domains for easier comprehension.

o Line 567 – please elaborate. The paragraph is only one sentence currently.

o Line 583 – what were the results of the clustered randomized controlled trial?

o Line 608 – I like the call to action being presented here. To tighten narrative, please tie it in or include how existing pediatric guidelines could be modified. Alternatively, should we consider devising new pediatric guidelines? Curious to hear your thoughts.

o Under limitations, please also include the influence of social desirability biases among participants or potential selection for responses given the skew towards Bangla-medium participants in the study. Additionally, please include limitations related to potential human error (such as not recording interviews, errors in translating from Bangla to English or vice versa).

**********

what does this mean?). If published, this will include your full peer review and any attached files.

If you choose “no”, your identity will remain anonymous but your review may still be made public.

**Do you want your identity to be public for this peer review?**  For information about this choice, including consent withdrawal, please see our Privacy Policy

Reviewer #1: No

Reviewer #2: No

Reviewer #3: No

**********

[NOTE: If reviewer comments were submitted as an attachment file, they will be attached to this email and accessible via the submission site. Please log into your account, locate the manuscript record, and check for the action link "View Attachments". If this link does not appear, there are no attachment files.]

While revising your submission, please upload your figure files to the Preflight Analysis and Conversion Engine (PACE) digital diagnostic tool, https://pacev2.apexcovantage.com/. PACE helps ensure that figures meet PLOS requirements. To use PACE, you must first register as a user. Registration is free. Then, login and navigate to the UPLOAD tab, where you will find detailed instructions on how to use the tool. If you encounter any issues or have any questions when using PACE, please email PLOS at figures@plos.org. Please note that Supporting Information files do not need this step.

Attachment Submitted filename: PONE-D-25-02782_reviewer notes.pdf

Attachment Submitted filename: PONE-D-25-02782_reviewer_comments_SS.docx

# Author comment: Author response to Decision Letter 1

**DOI:** 10.1371/journal.pone.0359518.r002

1 Oct 2025

NA

Attachment Submitted filename: Point by point response for editor and reviewers.pdf

# Peer review: Decision Letter 1

**DOI:** 10.1371/journal.pone.0359518.r003

**Authors:** Derebe Madoro Bunte

24 Nov 2025

Dear Dr. Kakon,

Thank you for submitting your manuscript to PLOS ONE. After careful consideration, we feel that it has merit but does not fully meet PLOS ONE’s publication criteria as it currently stands. Therefore, we invite you to submit a revised version of the manuscript that addresses the points raised during the review process.

==============================

**ACADEMIC EDITOR:**

==============================

Please submit your revised manuscript by Jan 08 2026 11:59PM. If you will need more time than this to complete your revisions, please reply to this message or contact the journal office at plosone@plos.org. When you're ready to submit your revision, log on to https://www.editorialmanager.com/pone/ and select the 'Submissions Needing Revision' folder to locate your manuscript file.

- A rebuttal letter that responds to each point raised by the academic editor and reviewer(s). You should upload this letter as a separate file labeled 'Response to Reviewers'.
- A marked-up copy of your manuscript that highlights changes made to the original version. You should upload this as a separate file labeled 'Revised Manuscript with Track Changes'.
- An unmarked version of your revised paper without tracked changes. You should upload this as a separate file labeled 'Manuscript'.

If you would like to make changes to your financial disclosure, please include your updated statement in your cover letter. Guidelines for resubmitting your figure files are available below the reviewer comments at the end of this letter.

If applicable, we recommend that you deposit your laboratory protocols in protocols.io to enhance the reproducibility of your results. Protocols.io assigns your protocol its own identifier (DOI) so that it can be cited independently in the future. For instructions see: https://journals.plos.org/plosone/s/submission-guidelines#loc-laboratory-protocols. Additionally, PLOS ONE offers an option for publishing peer-reviewed Lab Protocol articles, which describe protocols hosted on protocols.io. Read more information on sharing protocols at https://plos.org/protocols?utm_medium=editorial-email&utm_source=authorletters&utm_campaign=protocols.

We look forward to receiving your revised manuscript.

Kind regards,

Derebe Madoro Bunte,

Academic Editor

PLOS ONE

Journal Requirements:

If the reviewer comments include a recommendation to cite specific previously published works, please review and evaluate these publications to determine whether they are relevant and should be cited. There is no requirement to cite these works unless the editor has indicated otherwise.

Additional Editor Comments (if provided):

Reviewer #1

‎Thanks for your hardworking in refining the manuscript. Please see some comments that you may touch on:

‎1. "This study aimed to explore the challenges parents face when their students engage in excessive use of electronic digital screens. Additionally, it sought to capture participants’ recommendations for the

‎best use of digital devices among students."

‎Please make sure the conclusion has answered this accordingly and you could give one suggestion for further research recommendation.

‎2. Please specify where you describe the definition of "excessive use" since you reponded "We have quantified “excessive use” of digital devices as more than 2 hours per day in line 160. I have not seen the statement in the revised manuscript. Please recheck.

‎3. check line 124 = what is the significance of mentioning this sentence here? “We conducted qualitative interview from the same schools where previously quantitative study was performed.”

‎4. check line 133, please provide citation here.

‎5. I understand writing an academic manuscript is a bit tricky for non native English. Please have a thorugh and detailed check on the manucript since I found many need to be proofread such as:

‎check line 33, 37= “who attending” is this grammatically correct?

‎check line 59

‎check line 109

‎check line 111= you meant "in the UK"

‎check line 147, 160, 173, 208, 214, 219, 225, 261 etc.

‎Thank you and have a great one!

‎ Reviewer#2

‎Thanks for the revisions and I have read through the responses to the reviewers. I can see that a lot of further work has gone into this revision, however I am still concerned about a number of aspects.

‎Firstly, despite the improvement, there are significant grammar and punctuation errors, which for me is key in academic writing in English.

‎Secondly, I am concerned over the manner in which the results are presented, which hinders your ability to make good points in the discussion:

‎- The overview of how the themes will be presented is not yet sufficient. Please provide an overview of the number of themes that will be shown, name them and describe how they will be presented and why?

‎- There are 14 themes (shown below), which appear to be subthemes and are not consolidated in order to answer your aim of provide the results of “the challenges parents face in supervising and restricting children’s SV in urban Dhaka, Bangladesh, and”…” feedback on strategies that may help achieve a healthier balance between digital engagement and overall child well-being.”

‎Themes include

‎1. Students’ use digital device for education

‎2. Student’s reliance on the internet for learning support

‎3. Parents’ perspectives on digital learning and supervision

‎4. Teachers’ perspectives on digital learning and supervision

‎5. Safety concerns and limited peer interaction

‎6. Limited family time and communication

‎7. Scarcity of outdoor play and recreational opportunities

‎8. Unwanted exposure to age-inappropriate contents

‎9. Socioeconomic status and screen time

‎- remember in this subtheme your sample not randomised so you did not find an association?

‎10. Student’s perspective on unwanted content

‎11. Teachers’ perspective on supervision

‎12. Students’ perspective on supervision

‎13. Lack of parental digital literacy

‎14. Recommendations

‎These need to be consolidated into 3-5 major themes related to your aim. Please review thematic analysis with Braun and Clarkes methods for further information on this.

‎- Not only parents’ perspectives are provided, but also teachers and students, nor is it related only to parental challenges is managing SV? Please adapt your aim to represent the evidence you are presenting or adapt the evidence you are presenting?

‎- Recommendations are still under bulleted format, although I do appreciate them being put within a framework. Please describe how you will be presenting these recommendations according to this framework in your methods section and provide a brief review of the framework.

[Note: HTML markup is below. Please do not edit.]

Reviewers' comments:

Reviewer's Responses to Questions

**Comments to the Author**

Reviewer #1: (No Response)

Reviewer #2: (No Response)

**********

2. Is the manuscript technically sound, and do the data support the conclusions?

Reviewer #1: Partly

Reviewer #2: Yes

**********

3. Has the statistical analysis been performed appropriately and rigorously?

Reviewer #1: N/A

Reviewer #2: N/A

**********

4. Have the authors made all data underlying the findings in their manuscript fully available?

The PLOS Data policy

Reviewer #1: Yes

Reviewer #2: No

**********

5. Is the manuscript presented in an intelligible fashion and written in standard English?

Reviewer #1: No

Reviewer #2: No

**********

Reviewer #1: Thanks for the revisions and I have read through the responses to the reviewers. I can see that a lot of further work has gone into this revision, however I am still concerned about a number of aspects.

Firstly, despite the improvement, there are significant grammar and punctuation errors, which for me is key in academic writing in English.

Secondly, I am concerned over the manner in which the results are presented, which hinders your ability to make good points in the discussion:

- The overview of how the themes will be presented is not yet sufficient. Please provide an overview of the number of themes that will be shown, name them and describe how they will be presented and why?

- There are 14 themes (shown below), which appear to be subthemes and are not consolidated in order to answer your aim of provide the results of “the challenges parents face in supervising and restricting children’s SV in urban Dhaka, Bangladesh, and”…” feedback on strategies that may help achieve a healthier balance between digital engagement and overall child well-being.”

Themes include

1. Students’ use digital device for education

2. Student’s reliance on the internet for learning support

3. Parents’ perspectives on digital learning and supervision

4. Teachers’ perspectives on digital learning and supervision

5. Safety concerns and limited peer interaction

6. Limited family time and communication

7. Scarcity of outdoor play and recreational opportunities

8. Unwanted exposure to age-inappropriate contents

9. Socioeconomic status and screen time

- remember in this subtheme your sample not randomised so you did not find an association?

10. Student’s perspective on unwanted content

11. Teachers’ perspective on supervision

12. Students’ perspective on supervision

13. Lack of parental digital literacy

14. Recommendations

These need to be consolidated into 3-5 major themes related to your aim. Please review thematic analysis with Braun and Clarkes methods for further information on this.

- Not only parents’ perspectives are provided, but also teachers and students, nor is it related only to parental challenges is managing SV? Please adapt your aim to represent the evidence you are presenting or adapt the evidence you are presenting?

- Recommendations are still under bulleted format, although I do appreciate them being put within a framework. Please describe how you will be presenting these recommendations according to this framework in your methods section and provide a brief review of the framework.

Reviewer #2: Thanks for your hardworking in refining the manuscript. Please see some comments that you may touch on:

1. "This study aimed to explore the challenges parents face when their students engage in excessive use of electronic digital screens. Additionally, it sought to capture participants’ recommendations for the

best use of digital devices among students."

Please make sure the conclusion has answered this accordingly and you could give one suggestion for further research recommendation.

2. Please specify where you describe the definition of "excessive use" since you reponded "We have quantified “excessive use” of digital devices as more than 2 hours per day in line 160. I have not seen the statement in the revised manuscript. Please recheck.

3. check line 124 = what is the significance of mentioning this sentence here? “We conducted qualitative interview from the same schools where previously quantitative study was performed.”

4. check line 133, please provide citation here.

5. I understand writing an academic manuscript is a bit tricky for non native English. Please have a thorugh and detailed check on the manucript since I found many need to be proofread such as:

check line 33, 37= “who attending” is this grammatically correct?

check line 59

check line 109

check line 111= you meant "in the UK"

check line 147, 160, 173, 208, 214, 219, 225, 261 etc.

Thank you and have a great one!

**********

what does this mean?). If published, this will include your full peer review and any attached files.

If you choose “no”, your identity will remain anonymous but your review may still be made public.

**Do you want your identity to be public for this peer review?**  For information about this choice, including consent withdrawal, please see our Privacy Policy

Reviewer #1: No

Reviewer #2: No

**********

[NOTE: If reviewer comments were submitted as an attachment file, they will be attached to this email and accessible via the submission site. Please log into your account, locate the manuscript record, and check for the action link "View Attachments". If this link does not appear, there are no attachment files.]

To ensure your figures meet our technical requirements, please review our figure guidelines: https://journals.plos.org/plosone/s/figures

You may also use PLOS’s free figure tool, NAAS, to help you prepare publication quality figures: https://journals.plos.org/plosone/s/figures#loc-tools-for-figure-preparation.

NAAS will assess whether your figures meet our technical requirements by comparing each figure against our figure specifications.

Attachment Submitted filename: PONE-D-25-02782_R1_Reviewed.pdf

# Author comment: Author response to Decision Letter 2

**DOI:** 10.1371/journal.pone.0359518.r004

29 Mar 2026

March 17, 2026

To

Derebe Madoro Bunte

Academic Editor

PLOS ONE

Subject: Responses to Editor and Reviewer comments on the manuscript [PONE-D-25-02782R1]

Dear Editor,

Thank you for allowing us the opportunity to improve and resubmit our manuscript titled “Parental monitoring challenges and recommendation for managing excessive screen use among students aged 12–14 years in Dhaka, Bangladesh: a qualitative exploration”. We sincerely thank the Academic Editor and both reviewers for their careful evaluation of our manuscript and for their constructive and valuable comments. We have carefully reviewed all the comments and have addressed them individually. Additionally, we have incorporated the suggested changes into the manuscript where applicable. Below, we provide a point-by-point response, indicating how each concern has been addressed in the revised manuscript. Page and line numbers refer to the revised version.

Response to Academic Editor’s Comments:

Comment 1: Major comments highlight the need for a clear definition of “excessive use,” a conclusion that fully answers the study aim with a future research suggestion, and clarification of unclear statements and missing citations. Extensive grammar and language corrections are required throughout.

Response: Thank you for this comment. We have addressed each point mentioned. The definition of “excessive screen use” is clearly stated in the introduction and methods section as more than 2 hours per day, consistent with international guidelines. Please see pages 4, 8, and lines 72-74, and 177-179.

Further, we have rewritten the conclusion in order to directly answer the study's aim and now include a clear recommendation for future research. The manuscript has undergone extensive language editing for grammar, clarity, and academic tone. Please see pages 6, 28-29, and lines 120-123, 629-643.

Comment 2: The results section needs major restructuring, with the current 14 subthemes consolidated into 3–5 main themes supported by a clear thematic overview. The study's aim must align with the perspectives presented, and the recommendations should be written narratively and explained through the chosen framework.

Response: Thanks for this suggestion. As suggested, the results section has been fully restructured, consolidating the original 14 sub-themes into four major challenge-based themes, each supported by sub-themes and illustrative quotes. The study aim has been refined to reflect the inclusion of parents’, teachers’, and adolescents’ perspectives. Recommendations are now presented narratively, explicitly linked to the guiding framework, which is described in both the Methods and Discussion sections. Please see pages 10-11, 13-24, and lines 228-235, 281-547.

Response to 1st Reviewer’s Comments:

Comment-1: "This study aimed to explore the challenges parents face when their students engage in excessive use of electronic digital screens. Additionally, it sought to capture participants’ recommendations for the best use of digital devices among students." Please make sure the conclusion has answered this accordingly, and you could give one suggestion for further research recommendations.

Response: Many thanks for pointing out this and for your guidance. As suggested, the conclusion section has been revised to explicitly address both components of the study aim, parental monitoring challenges, and participant recommendations. We have also added a clear future research suggestion focusing on longitudinal and intervention-based studies to evaluate effective screen-use management strategies. Please see pages 28-29 and lines 629-643.

Comments-2: Please specify where you describe the definition of "excessive use" since you responded "We have quantified “excessive use” of digital devices as more than 2 hours per day in line 160. I have not seen the statement in the revised manuscript. Please recheck.

Response: We thank the reviewer for noting this omission. The definition of “excessive screen use” has now been explicitly included in the Methods section (Study Participants and sampling), specifying use of digital devices for more than 2 hours per day, with appropriate citation. Please see pages 4, 8, and lines 72-74, and 177-179.

Comments-3: check line 124 = what is the significance of mentioning this sentence here? “We conducted qualitative interviews from the same schools where previous quantitative study was performed.”

Response: Thanks for pointing this out. We agree that clarification is required. The sentence has been revised to explain that the qualitative study was designed as a follow-up exploration to contextualize findings from the earlier quantitative work. We have added a citation of previous quantitative work. This clarification has been added to the Study Design section. Please see page 6 and lines 128-131.

Comment-4: check line 133, please provide citation here.

Response: An appropriate citation has now been added to support the respective statement.

Comment-5: I understand writing an academic manuscript is a bit tricky for non native English. Please have a thorough and detailed check on the manuscript since I found many need to be proofread, such as:

‎check line 33, 37= “who attending” is this grammatically correct?

‎Check line 59

‎Check line 109

‎check line 111= You meant "in the UK."

‎check line 147, 160, 173, 208, 214, 219, 225, 261 etc.

Response: We fully acknowledge this concern. The manuscript has undergone thorough language editing, correcting all highlighted issues and many additional grammatical, punctuation, and stylistic errors throughout the text. We believe the manuscript now meets the standards of clear and correct academic English. Thank you.

Response to 2nd Reviewer’s Comments:

Comment-1 Despite the improvement, there are significant grammar and punctuation errors, which, for me, are key in academic writing in English.

Response: Many thanks for the feedback. We have again thoroughly checked the grammar and punctuation issues and addressed them throughout the paper. We appreciate this feedback and have conducted a comprehensive proofreading and language revision of the entire manuscript, including professional-level editing for grammar, punctuation, and clarity.

Comment-2: I am concerned about the manner in which the results are presented, which hinders your ability to make good points in the discussion. The overview of how the themes will be presented is not yet sufficient. Please provide an overview of the number of themes that will be shown, name them, and describe how they will be presented and why? The overview of themes in the Results section is insufficient.

Response: Thank you for this valuable comment. We have now restructured the result sections and presented the results considering the socio-ecological models framework pattern. We have added a clear thematic overview at the data analysis sub-section under the method section, and structured the challenge sub-section under the result accordingly. Please see pages 11, 13-19, and lines 234-235, 280-427.

Comment-3: There are 14 themes (shown below), which appear to be subthemes and are not consolidated in order to answer your aim of provide the results of “the challenges parents face in supervising and restricting children’s SV in urban Dhaka, Bangladesh, and.” feedback on strategies that may help achieve a healthier balance between digital engagement and overall child well-being.” These need to be consolidated into 3-5 major themes related to your aim. Please review thematic analysis with Braun and Clarkes methods for further information on this.

‎Themes include

‎1. Students’ use of digital devices for education

‎2. Student’s reliance on the internet for learning support

‎3. Parents’ perspectives on digital learning and supervision

‎4. Teachers’ perspectives on digital learning and supervision

‎5. Safety concerns and limited peer interaction

‎6. Limited family time and communication

‎7. Scarcity of outdoor play and recreational opportunities

‎8. Unwanted exposure to age-inappropriate contents

‎9. Socioeconomic status and screen time

‎- remember in this subtheme your sample not randomised so you did not find an association?

‎10. Student’s perspective on unwanted content

‎11. Teachers’ perspective on supervision

‎12. Students’ perspective on supervision

‎13. Lack of parental digital literacy

‎14. Recommendations

Response: We agree with this important suggestion. As suggested, we have revised the theme based on the socio-ecological framework and restructured the finding accordingly. Please see pages 13-24 and lines 280-547.

Comment-4: The sub-theme on socioeconomic status should not imply association due to non-random sampling.

Response: Thank you for identifying this issue. As the findings conflict with the methods, we have removed the specific findings to avoid any inconsistencies.

Comment-5: The aim focuses on parental challenges, yet perspectives from teachers and students are also presented. Not only parents’ perspectives are provided, but also teachers and students, nor is it related only to parental challenges is managing SV? Please adapt your aim to represent the evidence you are presenting or adapt the evidence you are presenting?

Response: Thank you for pointing this out. We have now revised the aims, taking into account the perspectives of both teachers and students. Please see page 6 and lines 120-123.

Comment 6: Recommendations are still under a bulleted format, although I do appreciate them being put within a framework. Please describe how you will be presenting these recommendations according to this framework in your methods section and provide a brief review of the framework.

Response: Thanks for this comment and concern. The recommendations section has been rewritten in narrative form, organized according to the socio-ecological framework used. We have added a brief description of the framework in the data analysis sub-section under the methods section and linked the recommendations explicitly to its components in the Results and Discussion. Please see pages: 10-11, 19-24, line: 228-235, 430-547.

Response to comments on the PDF file, which was attached to the email body:

Comments for the Introduction section

Comment: Where are the references for this? (line no 58-60)

Response: We have now added appropriate references. Thank you.

Comment: Add some further references here? (line no 65-67)

Response: Thank you. We have added further reference according to your suggestion.

Comment: putting it this way makes it seem like high SES is an unhealthy behaviour. I would move this to the first one you mention, then add in unhealthy behaviours (line-80)

Response: We agree with your thoughts and rearranged the sentences accordingly. Please see page 4 and line no 78-85.

Comment: I would remove this section and replace it with the current (line no-80)

Response: Thank you, and we have made the suggested change accordingly.

Comment: The grammar here is incorrect - engaged on devices? or engaged in using devices? (line no 81-82)

Response: Thanks for this concern. We have checked the grammar and identified that ‘engaged in using a device’ is correct.

Comment: combining... with use of internet...? (line no 83-85)

Response: We have paraphrased this sentence. Please see page 4 and line no 86-87.

Comment: This is colloquial language (line no 94)

Response: We have paraphrased this sentence.

Comment: Grammar (line no 98)

Response: We have corrected grammar.

Comment: Is there nothing from a more similar context to Bangladesh? (line no 99-105)

Response: Thank you for this suggestion. We have added the literature from Bangladesh and a similar context, including the literature from the developed country context.

Comment: Capital letter (line no 109)

Response: We addressed the issue.

Comment: This whole sentence doesn't make complete sense. (line no 109-111)

Response: We paraphrased this sentence now.

Comment: Please clarify whether you mean formal guidance on device use? (line no 115)

Response: Thank you for the comment. We have revised the respective lines to clear the meaning of formal guidance. Please see pages 24 and lines 531-556.

Response to the comments for the Materials and Methods section

Comment: Please reference this here? (line n0 125)

Response: We have added the appropriate reference.

Comment: to provide? (line no 126)

Response: We have paraphrased this sentence.

Comment: Reference (line no 131-133)

Response: We have provided an appropriate reference for this sentence.

Response to the comments for the Study setting

Comment: There is no information here about these settings, so that we would be able to know if our setting is similar or not to allow for transferability. All we know if that it is a high-seas area? (line no- 136-140)

Response: Thank you for this important comment. We have now provided a brief information about the setting under the method section. And as per the comment, we have made the necessary change throughout the paper.

Comment: this needs to go under participant sampling - not study setting (line no 143-148)

Response: Thanks for your suggestions. We have removed this part from the study setting section and added it to the study participants and sampling section. Please see pages 8 and lines 160-165.

Response to the comments for Study participants and sampling

Comment: Were these parents of the same children, i.e., a dyad, or different parents and children? (line no 151-153)

Response: Thank you for this comment. Parents and students were interviewed independently and were not recruited as parent-child dyads. Please see pages 7 and lines 151-159.

Comment: Also at the school (line no- 175,180)

Response: Thank you for this query. The focus group discussions were conducted on school premises in a quiet, private teacher’s common room with the permission of the school authorities. Participants were seated in a circle to facilitate open discussion and interaction. Only the participants and the research team were present during the FGDs, and all sessions were audio recorded with prior informed consent. This information has been clarified in the methods section under the study participation and sampling. Please see page 8.

Comment: observed the interviews or team, doing what? (line no 182,183)

Response: Thank you for this clarification request. The Mental Health Expert did not observe participants; rather, they observed the interview process and the performance of the interview team. The expert provided methodological feedback on interviewing techniques, probing, neutrality, and ethical conduct during data collection when necessary. This clarification has been incorporated into the Methods section.

Response to the comments for the Operational definition

Comment: This doesn't seem like the right place for this? (line no 188)

Response: Thank you for your guidance. We have moved the operational definition subsection to the right place, following the standard guideline just before the data collection subsection. Please see pages 10.

Response to the comments for the Result section:

Comment: This doesn't add much to the analysis - I would take this out. Please add in here an overview of the themes. I.e., note how many themes were identified, the names of the themes and whose perspectives are represented and why... (line no-249-250)

Response: Thank you for this comment. We have used the socio-ecological framework, which has been mentioned in the data analysis sub-section, and following the framework, we presented the findings under challenges and recommendations. In challenges, we identified four themes under the different levels of the framework, and findings under the recommendation have been presented under the framework components.

Comment: A more appropriate title for this seems to be Lack of guidance on device use for education? (line no 261)

Response: The sub-theme has been renamed to “Educational reliance on digital devices without structured guidance,” which more accurately reflects the identified challenge.

Comment: only one quote per sub-theme (line no 281-289)

Response: We have reduced the number of quotes and retained one representative quote per sub-theme to improve readability and conciseness.

Comment: this is not a challenge though? (line no 290)

Response: We have removed that subtheme, and only four board themes have been kept. The sub-theme has been renamed to “Educational reliance on digital devices without structured guidance,” which more accurately reflects the identified challenge. Please see pages 16 and lines 360-365.

Comment: how, when this sample is not randomised? The association between SES and screen time cannot be inferred, given the non-random sample. (line no 370-379)

Response: Thank you for highlighting this issue. We have now removed the particular findings to avoid methodological inconsistency.

Comment: Why is this section described with bullet points? If you want to include bullet points rather put this in a text box in a shortened manner and describe these recommendations here. (line no- 433)

Response: We have rewritten this section in narrative form and integrated participant recommendations into thematic paragraphs, while retaining clarity and coherence. Please see pages 19-24 and lines 431-540.

Response to the comments for the Discussion section

Comment: My problem is that the results haven't been presented as challenges, but as perspectives, influencing factors, and concepts. These subthemes need to be reworked into 3-4 major overarching themes that are consistent and do not have any overlapping concepts with other (line no 516)

Response: We have revised the result section with highlighted specific 4 subthemes into challenges. All discussion points now directly correspond to findings presented earlier, avoiding the introduction of new or unsupported themes. Please see pages 13-19 and lines 281-422.

Comment: But where have you presented these two themes in the evidence above? (line no 529-534)

Response: Thanks for your suggestion. We have rewritten this sentence to remove the inconsistency from the discussion part. We have mentioned this topic in the challenges section as well. Please see pages 16-17 and lines 359-384.

Comment: This appears to be an overarching theme - barriers to engaging in non-SV-based activities? (line no- 535-536)

Response: Thank you for this insightful observation. We agree that parents’ concerns about social safety, particularly for girls, reflect a broader barrier to children’s engagement in non-screen-based activities. This captures parental safety concerns, limited outdoor opportunities, and social restrictions that influence children’s activity patterns. This ensures consistency with the other themes and highlights the contextual factors affecting screen use in the challenges section. Please see pages 18-19 and lines 406-428.

Comment: How is this a challenge to managing SV use, though? Concerns over SV, yes, but not experienced challenges in managing SV. (line no 546,547)

Response: Thank you for this important comment. We acknowledge that concern about exposure to inappropriate content reflects perceived risks of screen viewing rather than a direct challenge in managing screen time. To address this, we have clarified in the manuscript that these concerns were reported as part of the broader contextual factors influencing parents’ monitoring strategies, rather than as operational difficulties in enforcing screen time limits. This distinction helps to differentiate between challenges in managing screen use and the perceived risks associated with content exposure.

Kind regards,

Dr. Shahria Hafiz Kakon

On behalf of Nahian Sultana, Bidhan Krishna Sarker, Tanjir Rashid Soron, Rashidul Haque, Fahmida Tofail

Attachment Submitted filename: Response to Reviewers.pdf

# Peer review: Decision Letter 2

**DOI:** 10.1371/journal.pone.0359518.r005

**Authors:** Derebe Madoro Bunte

14 Jul 2026

PLOS One

Dear Dr.  Kakon,

Thank you for submitting your manuscript to PLOS ONE. After careful consideration, we feel that it has merit but does not fully meet PLOS ONE’s publication criteria as it currently stands. Therefore, we invite you to submit a revised version of the manuscript that addresses the points raised during the review process.

Please submit your revised manuscript by Aug 28 2026 11:59PM. If you will need more time than this to complete your revisions, please reply to this message or contact the journal office at plosone@plos.org. When you're ready to submit your revision, log on to https://www.editorialmanager.com/pone/ and select the 'Submissions Needing Revision' folder to locate your manuscript file.

Please include the following items when submitting your revised manuscript:

- A letter that responds to each point raised by the academic editor and reviewer(s). You should upload this letter as a separate file labeled 'Response to Reviewers'.
- A marked-up copy of your manuscript that highlights changes made to the original version. You should upload this as a separate file labeled 'Revised Manuscript with Track Changes'.
- An unmarked version of your revised paper without tracked changes. You should upload this as a separate file labeled 'Manuscript'.

If you would like to make changes to your financial disclosure, please include your updated statement in your cover letter. Guidelines for resubmitting your figure files are available below the reviewer comments at the end of this letter.

If applicable, we recommend that you deposit your laboratory protocols in protocols.io to enhance the reproducibility of your results. Protocols.io assigns your protocol its own identifier (DOI) so that it can be cited independently in the future. For instructions see: https://journals.plos.org/plosone/s/submission-guidelines#loc-laboratory-protocols. Additionally, PLOS ONE offers an option for publishing peer-reviewed Lab Protocol articles, which describe protocols hosted on protocols.io. Read more information on sharing protocols at https://plos.org/protocols?utm_medium=editorial-email&utm_source=authorletters&utm_campaign=protocols.

As the corresponding author, your ORCID iD is verified in the submission system and will appear in the published article. PLOS supports the use of ORCID, and we encourage all coauthors to register for an ORCID iD and use it as well. Please encourage your coauthors to verify their ORCID iD within the submission system before final acceptance, as unverified ORCID iDs will not appear in the published article. *Only*  the individual author can complete the verification step; PLOS staff *cannot*  verify ORCID iDs on behalf of authors.

We look forward to receiving your revised manuscript.

Kind regards,

Derebe Madoro Bunte,

Academic Editor

PLOS One

Journal Requirements:

If the reviewer comments include a recommendation to cite specific previously published works, please review and evaluate these publications to determine whether they are relevant and should be cited. There is no requirement to cite these works unless the editor has indicated otherwise.

Please review your reference list to ensure that it is complete and correct. If you have cited papers that have been retracted, please include the rationale for doing so in the manuscript text, or remove these references and replace them with relevant current references. Any changes to the reference list should be mentioned in the rebuttal letter that accompanies your revised manuscript. If you need to cite a retracted article, indicate the article’s retracted status in the References list and also include a citation and full reference for the retraction notice.

Additional Editor Comments:

Thank you for revising the manuscript. The reviewers acknowledge improvements; however, further revision is required before acceptance.

**Required for acceptance:**  Ensure a consistent study aim throughout the manuscript; clarify the qualitative methods and thematic analysis; align the results and discussion with the stated aim; address the data availability concern; and revise the manuscript for grammar, tense, formatting, acronym consistency, citations, and academic language, including a discussion of the study's strengths and limitations.

**Recommended:**  Improve the organization of the discussion to better reflect the results and strengthen the integration of findings with the literature. Where reviewer comments differ, prioritize the required revisions above and provide a detailed point-by-point response to all comments.

[Note: HTML markup is below. Please do not edit.]

Reviewers' comments:

Reviewer's Responses to Questions

**Comments to the Author**

Reviewer #1: (No Response)

Reviewer #2: All comments have been addressed

**********

2. Is the manuscript technically sound, and do the data support the conclusions?

Reviewer #1: Partly

Reviewer #2: Partly

**********

3. Has the statistical analysis been performed appropriately and rigorously?

Reviewer #1: N/A

Reviewer #2: N/A

**********

4. Have the authors made all data underlying the findings in their manuscript fully available?

The PLOS Data policy

Reviewer #1: Yes

Reviewer #2: Yes

**********

5. Is the manuscript presented in an intelligible fashion and written in standard English?

Reviewer #1: No

Reviewer #2: No

**********

Reviewer #1: Thank you for the responses to comments and adaptions made to the article which have increased clarity in terms of representation of the results. Although many of the comments were addressed, some have not yet been fully or only partly addressed namely grammatical, acronymization and formatting challenges; the overview of the themes; and the link between the aim and the data presented in the results section. Please see general comments below, as well as the file uploaded referring to specific lines in the article.

Overall comments:

- I am in general concerned with the way that the qualitative methods and analysis are discussed and described, these need to be clearly described according to commonly used mechanisms and constructs (i.e. not tools, but data collection methods)

- Check SV acronym use and make sure it is consistent over the document and only

- Please in general review grammar and choice of specific words (i.e. overindulgence vs excessive SV; sole vs key factor)

- Sections are written in a mixture on current and past tense at times, please review

- Ensure that the information presented matches the heading (i.e. data collection methods are only discussed under data collection, not design or sampling)

Aim

The aim is not consistent within the document and presents a lack of clarity in terms of what exactly the study is aiming to explore, here are the four versions with the differences bolded. There needs to be clarity in your aim and the reporting of your results if you are presenting challenges with regulating adolescent SV or parental challenges specifically:

• “the challenges parents face when their students engage in excessive use of electronic digital screens. Additionally, it sought to capture participants’ recommendations for the best use of digital devices among students”

• “to understand parental experience in regulating screen use and strategies adopted to manage its duration and quality”

• “to explore the challenges parents face in supervising and restricting children’s screen viewing (SV), including the perspective of teachers and students in urban Dhaka, Bangladesh, and to gather their feedback on strategies that may help achieve a healthier balance between digital engagement and overall child well-being.”

• This study aimed to understand the challenges parents face when their children use electronic screens excessively and to gather parents’ suggestions on how digital devices can be used more healthily and effectively for students.

Data analysis:

• This section describing thematic analysis in general is concerning for me as I feel like it the description of the process could represent the need for further engagement with the analysis method and its underlying principles.

• This section requires further clarity and linking to thematic analysis principles. It seems that you have used thematic analysis using both an inductive and deductive approach. A codebook was created inductively. Then were those codes and subthemes created inductively or using the SES framework? Or were these themes then arranged under the SES framework?

Results

- The paragraphs remain bulleted

- Some evidence presented does not match the description preceeding the quote and there are instances where evidence referred to in the discussion is not presented in the results section.

Discussion

• It might be easier to follow the discussion if the results/challenges parents face are discussed in the same order that they are presented? And in separated paragraphs?

• Other evidence also needs to be synthesised and clearly linked to this study’s findings rather than just reported in the discussion, as it doesn’t always feel clear.

Reviewer #2:

1. I believe the last two sentences in the conclusion (lines 641-644) should be in the present tense. Line 85 should omit the extraneous word “even” to avoid sentiment. Line 86 should be “It is popular…”. Line 115 should be “the UK, parents….”. Line 121 should be “…aims to…”.

2. Please provide the necessary citations for lines 161-165 to support the statement.

3. Please proofread line 208 to follow the previous tense and check this “…and maintains

confidentiality” because it seems to be incoherent in the sentence.

4. Please proofread lines 558-560 to make it more readable and sound academic.

5. Please provide a citation for the sentence at the end of line 618.

6. You might want to discuss the strengths and limitations of this study in the discussion section.

**********

what does this mean?). If published, this will include your full peer review and any attached files.

If you choose “no”, your identity will remain anonymous but your review may still be made public.

**Do you want your identity to be public for this peer review?**  For information about this choice, including consent withdrawal, please see our Privacy Policy

Reviewer #1: No

Reviewer #2: No

**********

[NOTE: If reviewer comments were submitted as an attachment file, they will be attached to this email and accessible via the submission site. Please log into your account, locate the manuscript record, and check for the action link "View Attachments". If this link does not appear, there are no attachment files.]

To ensure your figures meet our technical requirements, please review our figure guidelines: https://journals.plos.org/plosone/s/figures

You may also use PLOS’s free figure tool, NAAS, to help you prepare publication quality figures: https://journals.plos.org/plosone/s/figures#loc-tools-for-figure-preparation.

NAAS will assess whether your figures meet our technical requirements by comparing each figure against our figure specifications.

Attachment Submitted filename: Reviewer comments.docx

# Author comment: Author response to Decision Letter 3

**DOI:** 10.1371/journal.pone.0359518.r006

10 Aug 2026

August 5, 2026

To

Derebe Madoro Bunte

Academic Editor

PLOS ONE

Subject: Responses to Editor and Reviewer comments on the manuscript [PONE-D-25-02782R2]

Dear Editor,

Thank you for allowing us the opportunity to improve and resubmit our manuscript titled “Parental monitoring challenges and recommendation for managing excessive screen use among students aged 12–14 years in Dhaka, Bangladesh: a qualitative exploration”. We sincerely thank the Editor and the Reviewer for their careful evaluation of our manuscript and for providing thoughtful and constructive comments. We greatly appreciate the time and effort devoted to reviewing our work. The comments and suggestions have been invaluable in improving the quality, clarity, and scientific rigor of the manuscript. We have carefully considered each comment and revised the manuscript accordingly. A detailed point-by-point response is provided below, and all corresponding changes have been incorporated into the revised manuscript. For ease of review, the revisions have been highlighted in the manuscript.

Reviewer Comment 1 (Line 27): Consider replacing "overindulgence" with a more appropriate word.

Response: Thank you for this helpful suggestion. We agree that the term "overindulgence" was not the most appropriate in this context. We have replaced it with "excessive use" throughout the manuscript to improve clarity and ensure consistency with the terminology used in the literature. [See the line no. 24]

Reviewer Comment 2 (Lines 29 and 48): Specify whose specific recommendations you are getting rather than saying participants or population; you have three different types of participants in your study or three populations

Response: Thank you for this valuable comment. We have revised the manuscript to clearly specify that the recommendations were provided by parents, teachers, and students, rather than using the general terms "participants" or "population." This improves the precision and clarity of the findings. [See the line no. 26]

Reviewer Comment 3 (Line 36): What different types of in-depth interviews?

Response: Thank you for the suggestion. We have clarified that separate in-depth interviews (IDIs) were conducted with parents, teachers, and students to capture the perspectives of each stakeholder group. [See the line no. 32-34]

Reviewer Comment 4 (Line 36): “A thematic analysis was performed using an inductive approach based on the socio-ecological model framework” – an inductive approach cannot be base specifically on a model or framework, this is a deductive approach as a framework is being applied?

Response: Thank you for this valuable comment. We agree that applying the socio-ecological model represents a deductive framework. We have revised the manuscript to clarify that themes were first generated inductively from the data and were subsequently organized and interpreted using the socio-ecological model as an analytical framework, rather than stating that the inductive approach was based on the framework. [See the line no. 35-37]

Reviewer Comment 5 (Line 47): Parental challenges – please be more specific.

Response: Thank you. We have revised the sentence to specify the key parental challenges identified in this study, including monitoring children's screen viewing, supervising age-specific online content, balancing educational and recreational device use, promoting alternative offline activities, and lack of digital literacy among parents. [See the line no. 47]

Reviewer Comment 6 (Line 55): Do you mean inescapable?

Response: Thank you for pointing this out. We have replaced the original wording with "inescapable" to improve the accuracy of the sentence. [See the line no. 53]

Reviewer Comment 7 (Line 60): Four factors related to SES were noted but only one described.

Response: Thank you for your observation. We have revised this section to clearly describe the four socioeconomic factors identified in the literature, including parenting practices, digital skill expectations, access to digital resources, and opportunities for alternative recreational activities. [See the line no. 58-60]

Reviewer Comment 8 (Line 65): In adolescents?

Response: Yes, thank you; we have revised the sentence to specify that the evidence refers to adolescents. [See the line no. 69]

Reviewer Comment 9 (Line 70): In addition to what?

Response: We agree with the reviewer and have removed the phrase to improve readability. [See the line no. 72-74]

Reviewer Comment 10 (Line 72): Continue using the term "screen viewing" rather than screen time.

Response: Thank you for this suggestion. We have standardized the terminology throughout the manuscript and consistently use screen viewing (SV) except where referring directly to established international guidelines that specifically use the term "screen time."

Reviewer Comment 11 (Lines 75–77): Does this not belong better in the first paragraph where you are talking about what influences technology habits? This second paragraph is about the outcomes of high SV so it doesn’t really align with the argument

Response: We agree. The relevant information has been relocated to the preceding paragraph discussing determinants of technology use, resulting in a more logical flow of the Introduction. [See the line no. 64-66]

Reviewer Comment 12 (Line 81): Increased compared to what? Historical use? The WHO recommendations?

Response: Thank you. We have revised the sentence with “ A study found that approximately 79% of adolescents exceeded the WHO recommended recreational screen viewing.” [See the line no. 79-81]

Reviewer Comment 13 (Line 84): SV acronym described again.

Response: Thank you for pointing this out. We have removed the repeated definition of the acronym "SV" and retained its definition only at its first appearance in the manuscript.

Reviewer Comment 14 (Lines 87–91): Surely the AAP guidelines should be discussed together with the WHO guidelines in the paragraph above? Or bring the WHO guideline here?

Response: We appreciate this suggestion. We have reorganized the Introduction to discuss the WHO and American Academy of Pediatrics recommendations together, providing a clearer comparison of international guidance. [See the line no. 81-85]

Reviewer Comment 15 (Lines 93–108): This section seems to be about the challenge’s parents face in managing SV in adolescents if I am right – please look at the evidence presented in this section and synthesize fully? For example, evidence from Canda is presented, then Bangladesh, then HIC contexts – with Canada also being a HIC context, that literature should be synthesised with the latter.

Response: Thank you for this valuable recommendation. We have substantially revised this section by synthesizing evidence from high-income and low- and middle-income countries rather than presenting studies sequentially. This revision provides a more coherent overview of parental challenges in managing adolescents' screen viewing. [See the line no. 94-115]

Reviewer Comment 16 (Line 96): SV restrictions can limit opportunities and development. This only really talks to part of what the paper says – HICs are described as more restrictive, and restrictions can reduce opportunity but also increase risk.

In what circumstances? LMIC’s?

Response: Thank you for this valuable comment. We have revised the text to provide a more balanced interpretation, clarifying that while overly restrictive screen-viewing practices may limit opportunities for digital learning and development, insufficient restrictions may increase the risk of excessive screen use and its associated adverse outcomes.

We have clarified that the statement refers to low- and middle-income countries experiencing rapid expansion of digital technology, including Bangladesh. [See the line no. 98-103]

Reviewer Comment 17 (Line 100): "The sole challenge" should be "a key challenge."

Response: We agree and have revised the wording accordingly. [See the line no. 106]

Reviewer Comment 18 (Line 108): The Indian study is not referenced.

Response: Thank you for identifying this omission. The appropriate reference has now been added to both the text and the reference list and the sentence has been paraphrased for better understanding for the reader. [See the line no. 119]

Reviewer Comment 19 (Line 108 onwards): The ongoing section of this paragraph is a new argument and should be a new paragraph I think this seems to be about strategies to manage SV, no longer the challenges that parents face in trying to manage SV in their adolescents?

Response: We agree. The paragraph has been divided into two separate paragraphs to distinguish between parental challenges and potential management strategies, thereby improving readability and logical progression. [See the line no. 120-124]

Reviewer Comment 20 (Line 122): From the perspectives of parents, teachers, and 12-14 years old adolescents (not only teachers and adolescents?)

Response: Thank you. We have revised the sentence to specify that the study explored the perspectives of parents, teachers, and students aged 12–14 years. [See the line no. 130]

Reviewer Comment 21 (Line 133): A combination of multiple data collection methods (not tools). Data collection methods, not study design, and therefore should not be discussed under this section. Please rather describe the qualitative study here to provide further background to the study design and how the qualitative work fits into this.

Response: Thank you for this valuable comment. We have revised this section accordingly by removing the description of the data collection methods from the Study Design section. Instead, we now provide additional background on the qualitative study, explaining its purpose, rationale, and how it complements the previous quantitative study. The description of the in-depth interviews (IDIs) and focus group discussions (FGDs) has been moved to the Data Collection Methods section, and the term "data collection methods" has replaced "tools" throughout the manuscript. [See the line no. 134-155]

Reviewer Comment 22 (Line 138-144): There are no references for this section?

Responses: Thank you for this comment. We have carefully reviewed this section and added the appropriate references to support the statements and methodological approach described. This revision ensures that all relevant information is adequately referenced in the revised manuscript. The following reference has been added as Reference 22. [See the line no. 138]

Reviewer Comment 23 (Line 145): Start off this section with precisely who your different samples were. This section is on the whole very ‘wordy’ and this makes it a little difficult to follow.

Response: Thank you for this helpful suggestion. We have revised the Study participants and sampling section to begin with a clear description of the three participant groups (parents, students, and teachers), their eligibility criteria, and recruitment strategy. We also edited the section to improve clarity and reduce unnecessary detail, making it more concise and easier to follow. [See the line no. 156-183]

Reviewer Comment 24 (Line 167-171): Remove data collection methods from this section, as this related to data collection not sampling.

Response: Thank you. We have removed descriptions related to data collection procedures from the sampling section and relocated them to the Data collection and quality checking section to improve the organization of the Methods. [See the line no. 206-231]

Reviewer Comment 25 (Line 208–209, 212–213): Check sentences for clarity/grammar.

Response: Thank you. We carefully revised these sentences to improve grammar, readability, and overall clarity while preserving the intended meaning. [See the line no. 180-181 and 226-228]

Reviewer Comment 26 (Line 217–220): This information relates to data analysis and sampling; please rather place in the correct sections, rather than data collection methods.

Response: Thank you for pointing this out. The relevant text has been moved from the data collection method to the Study participants and sampling and data analysis section, where it more appropriately belongs. [See the line no. 158 and Data analysis section 232-257]

Reviewer Comment 27 (Line 227): You can describe thematic analysis here already, as familiarization is a part of the process so you effectively already started thematic analysis with familiarization?

Response: Thank you. We have revised the Data analysis section to clearly state that thematic analysis began during data familiarization, following Braun and Clarke's approach, and have described the subsequent coding and theme development process accordingly. [See the line no. 238-245, reference no 24]

Reviewer Comment 28 (Line 237-241): Still limited discussion of reflexivity, specifically important when using an inductive approach, and how this relates to rigour.

Response: Thank you for this important suggestion. We have expanded the Data analysis section by describing measures taken to enhance rigor, including collaborative coding, triangulation, and acknowledgment of the research team's role during analysis. We have also strengthened the discussion on reflexivity and its contribution to trustworthiness in the inductive analytical process. [See the line no. 246-250]

Reviewer Comment 29 (Line 255): The term ‘gift’ doesn’t sit well in terms of possible coercion and potential for social desirability bias – it would be better to talk about compensation for their time.

Response: Thank you. We agree and have replaced the term "gift" with "compensation for their time and transportation expenses" to better reflect the ethical purpose of the reimbursement and avoid any implication of coercion. [See the line no. 270]

Reviewer Comment 30 (Line 281): The description of the themes do not relate to your actual themes.

Response: Thank you for identifying this inconsistency. We have revised the introductory paragraph of the Results section so that the descriptions accurately correspond to the final themes presented in the manuscript, including "Challenges faced by parents in monitoring children's screen use." [See the line no. 282-296]

Reviewer Comment 31(Line 298): Is the quote more about a lack of opportunities for family engagement than adolescent preferences for engaging with friends than family?

Response: Thank you. We agree and have revised the accompanying narrative to accurately reflect that the quotation illustrates limited family engagement and parental availability rather than adolescents' preference for peers. [now the line no is 313-321]

Reviewer Comment 32 (Line 316): What is eve-teasing?

Response: Thank you. We have clarified the term by replacing "eve-teasing" with "sexual harassment in public spaces" to improve clarity for an international readership. [See the line no. 330-333]

Reviewer Comment 33 (Line 318): The quote is not about a lack of availability of peers/cousins, but lack of engagement when they are together?

Response: Thank you. We have revised the interpretation to better reflect that the quotation illustrates reduced engagement among peers and cousins due to device use, rather than their absence. [See the line no. 329-333]

Reviewer Comment 34 (Line 331): This quote is about digital device usage – not unsupervised access, so I’m not sure if it is good evidence?

Response: Thank you. We have replaced the interpretation so that it accurately reflects the content of the quotation. We also selected a more appropriate supporting quotation where necessary to better align with the theme of parental monitoring and supervision. [See the line no. 341-344]

Reviewer Comment 35 (Line 338): Is this not a recommendation?

Response: Thank you for this insightful comment. We agree that the original wording was presented as a recommendation rather than a finding. We have revised the text to describe teachers' observations regarding children's exposure to age-inappropriate online content and the challenges related to parental digital literacy and monitoring capacity. The revised narrative now accurately reflects the supporting quotation and is presented as a study finding rather than a recommendation. [See the line no. 341-344]

Reviewer Comment 36 (Line 386): This is the impact of excessive SV and not related to the theme?

Response: Thank you. We have revised the text to ensure that only findings directly related to the theme are presented. The discussion of the impacts of excessive screen viewing has been relocated to a more appropriate section. [See the line no. 382-389]

Reviewer Comment 37 (Line 440 onwards): Recommendations are still presented as bullet points with limited quotes attached as evidence?

Response: Thank you. We have substantially revised the Recommendations section by presenting the findings in narrative form under the socio-ecological framework and integrating illustrative participant quotations to support each recommendation. [See the line no. 451-456, 464-669, 472-475, 477-478]

Reviewer Comment 38 (Line 462, 509): Choose one of the quotes and cut them shorter or put under the appropriate section above.

Response: Thank you. We have cut and shortened the quotations by retaining only the most relevant excerpts and relocated them to the most appropriate thematic sections to improve readability and avoid repetition.

Reviewer Comment 39 (Line 558-560): This does not link to the preceding or proceeding paragraph?

Response: Thank you. We revised this paragraph to improve its logical flow and ensure that it clearly connects with the surrounding discussion. [See the line no. 579-583]

Reviewer Comment 40 (Line 585): “Many parents reported feeling unprepared to discuss sexual development, which compounded the challenges of supervision.” I don’t think that this was presented in the results?

Response: Thank you for your careful observation. We agree that this statement was insufficiently supported by the qualitative findings presented. Therefore, we have removed this statement from the Discussion to ensure that all interpretations are directly grounded in the study results. [See the line no. 608-609]

Kind regards,

Dr. Shahria Hafiz Kakon

On behalf of Nahian Sultana, Bidhan Krishna Sarker, Tanjir Rashid Soron, Rashidul Haque, Fahmida Tofail

Attachment Submitted filename: Response to Reviewers Comments 05.08.2026.docx

# Editor report: Decision Letter 3

**DOI:** 10.1371/journal.pone.0359518.r007

**Authors:** Derebe Madoro Bunte

16 Sep 2026

Parental monitoring challenges and recommendation for managing excessive screen use among students aged 12–14 years in Dhaka, Bangladesh: a qualitative exploration

PONE-D-25-02782R3

Dear Dr. Shahria Hafiz Kakon,

We’re pleased to inform you that your manuscript has been judged scientifically suitable for publication and will be formally accepted for publication once it meets all outstanding technical requirements.

Within one week, you’ll receive an e-mail detailing the required amendments. When these have been addressed, you’ll receive a formal acceptance letter and your manuscript will be scheduled for publication.

An invoice will be generated when your article is formally accepted. Please note, if your institution has a publishing partnership with PLOS and your article meets the relevant criteria, all or part of your publication costs will be covered. Please make sure your user information is up-to-date by logging into Editorial Manager at Editorial Manager® and clicking the ‘Update My Information' link at the top of the page. For questions related to billing, please contact billing support.

If your institution or institutions have a press office, please notify them about your upcoming paper to help maximize its impact. If they’ll be preparing press materials, please inform our press team as soon as possible -- no later than 48 hours after receiving the formal acceptance. Your manuscript will remain under strict press embargo until 2 pm Eastern Time on the date of publication. For more information, please contact onepress@plos.org.

Kind regards,

Derebe Madoro Bunte

Academic Editor

PLOS One

Additional Editor Comments (optional):

Reviewers' comments:

# Editor report: Acceptance letter

**DOI:** 10.1371/journal.pone.0359518.r008

**Authors:** Derebe Madoro Bunte

PONE-D-25-02782R3

PLOS One

Dear Dr. Kakon,

I'm pleased to inform you that your manuscript has been deemed suitable for publication in PLOS One. Congratulations! Your manuscript is now being handed over to our production team.

At this stage, our production department will prepare your paper for publication. This includes ensuring the following:

* All references, tables, and figures are properly cited

* All relevant supporting information is included in the manuscript submission,

* There are no issues that prevent the paper from being properly typeset

You will receive further instructions from the production team, including instructions on how to review your proof when it is ready. Please keep in mind that we are working through a large volume of accepted articles, so please give us a few days to review your paper and let you know the next and final steps.

Lastly, if your institution or institutions have a press office, please let them know about your upcoming paper now to help maximize its impact. If they'll be preparing press materials, please inform our press team within the next 48 hours. Your manuscript will remain under strict press embargo until 2 pm Eastern Time on the date of publication. For more information, please contact onepress@plos.org.

You will receive an invoice from PLOS for your publication fee after your manuscript has reached the completed accept phase. If you receive an email requesting payment before acceptance or for any other service, this may be a phishing scheme. Learn how to identify phishing emails and protect your accounts at https://explore.plos.org/phishing.

If we can help with anything else, please email us at customercare@plos.org.

Thank you for submitting your work to PLOS One and supporting open access.

Kind regards,

PLOS One Editorial Office Staff

on behalf of

Dr. Derebe Madoro Bunte

Academic Editor

PLOS One
